# Supplementary material for: Non-chelation control in allylations of α-oxy ketones using group-14 allylatranes
Source: Nat Commun. 2026 Mar 3;17:2019. doi: 10.1038/s41467-026-69732-2 (PMC12957295; doi:10.1038/s41467-026-69732-2)
Supplement: Supplementary file 2 — Supplementary Information [file 41467_2026_69732_MOESM2_ESM.pdf]

Supplementary information

Non-Chelation Control in Allylations of α-Oxy Ketones Using Group-14 Allylatranes

Yuya Tsutsui,[1] Kokoro Shiga,[1] Akihito Konishi,*[1,2] and Makoto Yasuda*[1,2]

[1] Department of Applied Chemistry, Graduate School of Engineering, The University of Osaka, 2-1 Yamadaoka, Suita, Osaka 565-0871, Japan
a-koni@chem.eng.osaka-u.ac.jp, yasuda@chem.eng.osaka-u.ac.jp
[2] Innovative Catalysis Science Division, Institute for Open and Transdisciplinary Research Initiatives (ICS-OTRI), The University of Osaka, Suita, Osaka 565-0871, Japan

Table of Contents

1. General 3

2. Materials 3

3. Preparation of 1E(allyl) and its derivatives 4

4. Synthetic procedures 4

5. X-ray crystallographic data 70

5-1. Allylsilatrane 1Si(allyl) 70

5-2. Allylgermatrane 1Ge(allyl) 71

5-3. Allylstannatrane 1Sn(allyl) 72

5-4. Methallylsilatrane 1Si(methallyl) 73

5-5. 2-Phenylallylsilatrane 1Si(2-phenylallyl) 74

5-6. *syn*-3a 76

5-7. *anti*-3a′ 77

5-8. *anti*-3d 79

5-9. Summary of structural data of allylatranes 81

6. Diastereoselective allylation reactions 82

6-1. Screening reaction conditions 82

6-1-1. Screening allyl nucleophiles 82

6-1-2. Screening activators and solvents with 1Si(allyl) 83

6-2. *anti*-Selective allylation using 1Si(allyl) 84

6-3. *syn*-Selective allylation using Sn(II) salts 84

6-4. Screening allyl nucleophiles in allylation of 2q 85

6-5. Stereochemical determination of cyclobutane product 3q 85

7. Mechanistic study 90

7-1. Stability of allylsilatranes 90

7-2. Evaluation of nucleophilicity 91

7-3. In-situ observation of allylation 92

8. Computational method 94

8-1. General 94

8-2. HOMO energies of 1E(allyl) 94

8-3. Second order perturbation analysis of allylic moieties of 1E(allyl) 95

9. Computational estimation of the reaction profile 97

9-1. General 97

9-2. Summary for the reaction profiles for the allylations of 2a with 1Si(allyl) and BF_3 98

10. References 100

1. General

NMR spectra were recorded on JEOL-ECS400 (400 MHz for ^{1}H, 100 MHz for ^{13}C, 127 MHz for ^{11}B, 78.7 MHz for ^{29}Si NMR and 147.6 MHz for ^{119}Sn NMR). Chemical shifts were reported in ppm on the δ scale relative to tetramethylsilane (δ = 0 for ^{1}H NMR) and $CDCl_3$ (δ = 77.0 for ^{13}C NMR) as an internal reference. Chemical shifts were reported in ppm on the δ scale relative to $BF_3{\cdot}OEt_2$ (δ = 0 for ^{11}B NMR), tetramethylsilane (δ = 0 for ^{29}Si NMR) and tetramethylstannane (δ = 0 for ^{119}Sn NMR) as an external reference. ^{1}H NMR spectroscopy splitting patterns were designated as singlet (s), doublet (d), doublet of doublets (dd), doublet of doublet of doublets (ddd), triplet (t), doublet of triplets (dt), quartet (q), quintet (quint), sextet (sext), and septet (sep). Splitting patterns that could not be interpreted or easily visualized were designated as multiplet (m) or broad (br). ^{1}H and ^{13}C NMR signals of compounds were assigned using HMQC, HSQC, HMBC, COSY, and ^{13}C off-resonance techniques. Silica-gel column chromatography was carried out using Silica Gel 60 N (Kanto Chemical Co., spherical, neutral, 0.040-0.050 mm) at medium pressure. Purification by recycle GPC was performed on LaboACE LC-5060P (Japan Analytical Industry Co., Ltd.). High-resolution mass spectra were obtained by a Q Exactive Orbitrap (Thermo Fisher Scientific), a JEOL JMS-700, a Shimadzu GCMS-QP2010 Ultra, a JEOL JMS-S3000, and JEOL JMS-T100LP. IR spectra were recorded as thin films or as solids in KBr pellets on a JASCO FT/IR 6200 spectrophotometer. Data collection for X-ray crystal analysis was performed on Rigaku/XtaLAB Synergy-S/Cu (CuK$_{\alpha}$ λ = 1.54187 Å) diffractometers. All non-hydrogen atoms were refined with anisotropic displacement parameters and hydrogen atoms were placed at calculated positions and refined "riding" on their corresponding carbon atoms by Olex2[1] program. Unless otherwise noted, all reactions were performed with dry solvents under an atmosphere of N_2 gas in dried glassware using standard vacuum-line techniques.

2. Materials

Unless otherwise noted, all materials including dry solvents were obtained from commercial suppliers and used as received. The compounds **2a**, **2b**, and **2n** are commercially available and were used without further purification. The known compounds **1**SiCl,[2] **1**GeCl,[2] **1**SnCl,[2] **2c**,[3] **2d**,[4] **2e**,[5] **2f**,[6] **2j**,[7] **2k**,[8] **2l**,[9] **2m**,[10] **2o**,[11] **2p**,[11] and **2q**[12] were synthesized according to procedures reported in the literature. The compounds **1**Si(allyl), **1**Ge(allyl), **1**Sn(allyl), **1**Si(methallyl), **1**Si(2-phenylallyl), **2g**, **2h**, and **2i** were prepared by the methods described in the supporting information. The products *syn*/*anti*-**3j** were also found in the literature.[7]

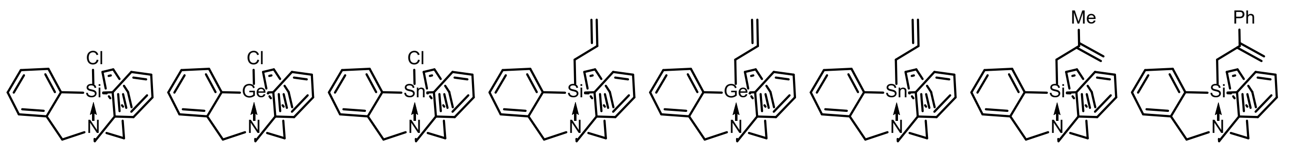

1SiCl **1**GeCl **1**SnCl **1**Si(allyl) **1**Ge(allyl) **1**Sn(allyl) **1**Si(methallyl) **1**Si(2-phenylallyl)

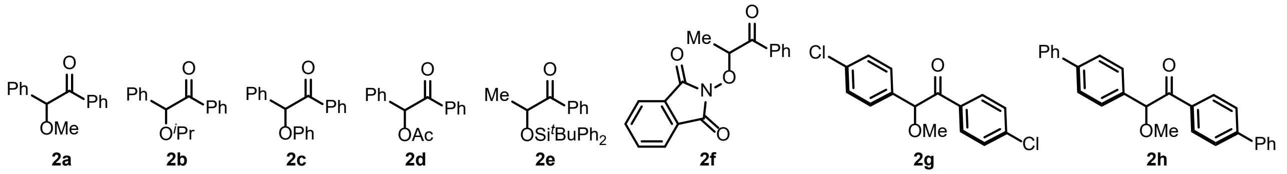

2a **2b** **2c** **2d** **2e** **2f** **2g** **2h**

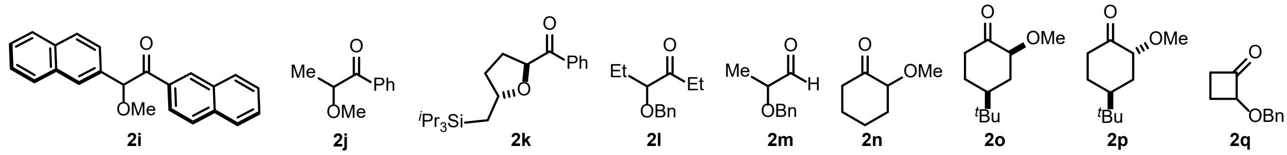

2i **2j** **2k** **2l** **2m** **2n** **2o** **2p** **2q**

3. Preparation of 1E(allyl) and its derivatives

The synthesis of the atrane-type allylmetals **1**E(allyl) (E = Si, Ge, Sn) is summarized. According to our previous study,[2] **1**ECl were prepared. Subsequently, the treatment of the corresponding atrane **1**ECl with allylmagnesium bromide afforded **1**E(allyl) in moderate to high yields. The resulting **1**E(allyl) can be isolated by silica gel column chromatography under ambient conditions. Furthermore, **1**Si(metallyl) and **1**Si(2-phenylallyl) were synthesized. The chlorosilatrane **1**SiCl was converted to the silyllithium **1**SiLi, followed by the reaction with 3-chloro-2-methyl-1-propene or *α*-(bromomethyl)styrene.

The ^{29}Si NMR signal for **1**Si(allyl) (−50.2 ppm in $CDCl_3$) is shifted to a higher field than that of allyltriphenylsilane (−13.6 ppm), indicating a strong electron donation from the nitrogen atom via the transannular N–Si bond. A similar correlation was also observed in ^{119}Sn NMR (**1**Sn(allyl), −159.9 ppm; allyltriphenylstannane, −122.0 ppm in $CDCl_3$).

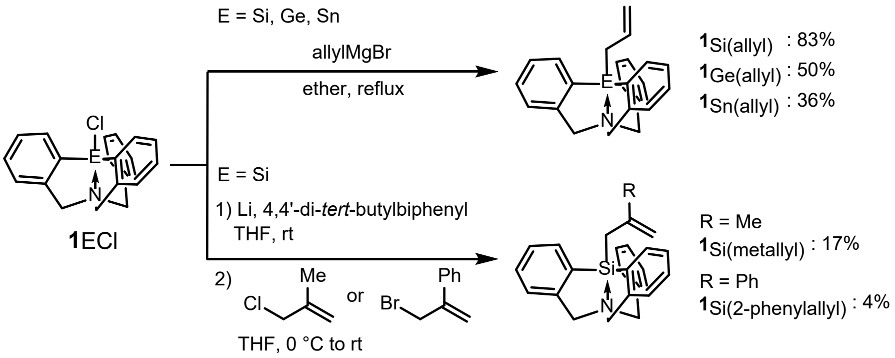

Synthetic routes for allylatranes **1**E(allyl) and its derivatives

4. Synthetic procedures

General procedure for the synthesis of atrane-type derivatives 1ECl (E = Si or Sn)

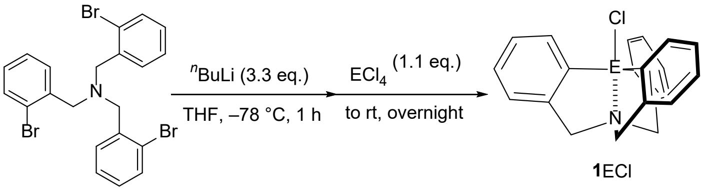

Under N_2 atmosphere, ^{n}BuLi (1.6 M in hexane, 41.3 mL, 66.0 mmol) was titrated to a solution of tris(2-bromobenzyl)amine (10.5 g, 20.0 mmol) in THF (120 mL) at –78 °C and stirred for 1 hour. After dropwisely adding ECl_4 (22.0 mmol) into the reaction mixture, the reaction temperature was allowed to warm up to room temperature. The reaction mixture was quenched by methanol and the solvents were removed under vacuum to give the crude product. Washing the product with methanol afforded the pure product **1**ECl as a colorless solid.

12-chloro-7,12-dihydro-5*H*-12,6-([1,2]benzenomethano)dibenzo[*c,f*][1,5]azasilocine 1SiCl

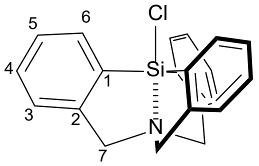

yield quant.; mp >300 °C; IR (KBr) *ν* = 3060 (m), 2922 (m), 1592 (m), 1441 (s), 1353 (s), 1234 (s), 1127 (s), 1067

(s), 968 (s), 828 (s), 726 (s) cm^{-1}; ^{1}H NMR (400 MHz, $CDCl_3$) 8.39 (dd, J = 7.4, 1.6 Hz, 3H, 6-H), 7.37 (td, J = 7.4, 1.2 Hz, 3H, 5-H), 7.33 (td, J = 7.8, 1.6 Hz, 3H, 4-H), 7.11 (d, J = 6.8 Hz, 3H, 3-H), 4.02 (s, 6H, 7-H); ^{13}C NMR (100 MHz, $CDCl_3$) 143.29 (s, C-2), 138.13 (d, C-6), 134.25 (s, C-1), 130.13 (d, C-4), 128.22 (d, C-5), 124.18 (d, C-3), 57.88 (t, C-7); ^{29}Si{^{1}H} NMR (78.5 MHz, $CDCl_3$, Me_4Si in $CDCl_3$ as an external standard) –52.3; MS (EI$^+$, 70 eV) m/z 349 ([M+2]$^+$, 10), 347 (M$^+$, 27), 312 (17), 256 (100), 229 (11), 165 (17); HRMS (EI$^+$, 70 eV) Calculated ($C_{21}H_{18}ClNSi$): 347.0897 (M$^+$), Found: 347.0890 (M$^+$); Analysis $C_{21}H_{18}ClNSi$ (347.9170), Calculated: C, 72.50; H, 5.22; Cl, 10.19; N, 4.03; Si, 8.07, Found: C, 72.59; H, 5.17; N 4.11.

^{1}H NMR: (400 MHz, $CDCl_3$)

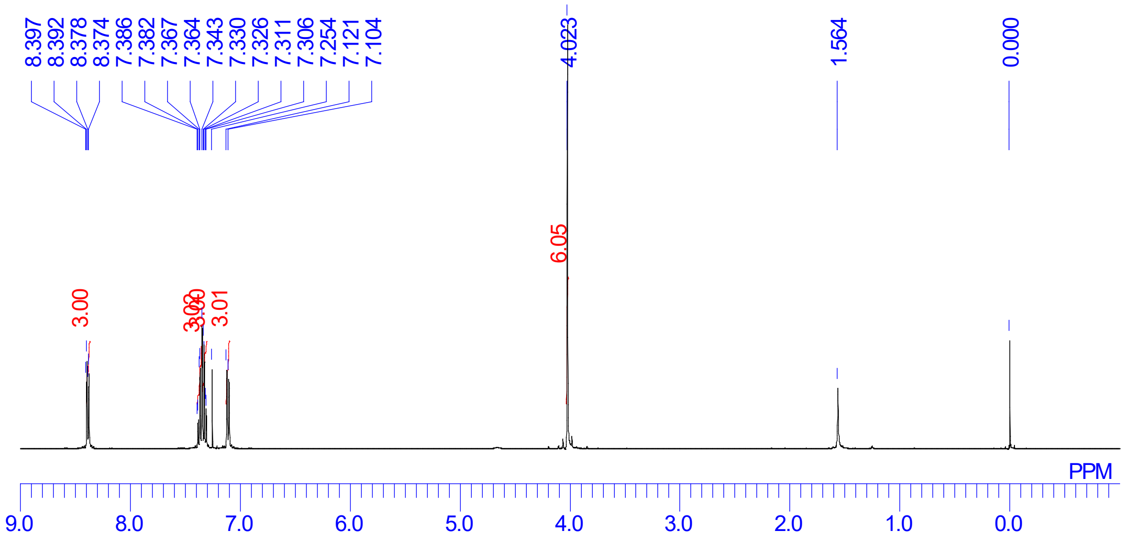

^{13}C NMR: (100 MHz, $CDCl_3$)

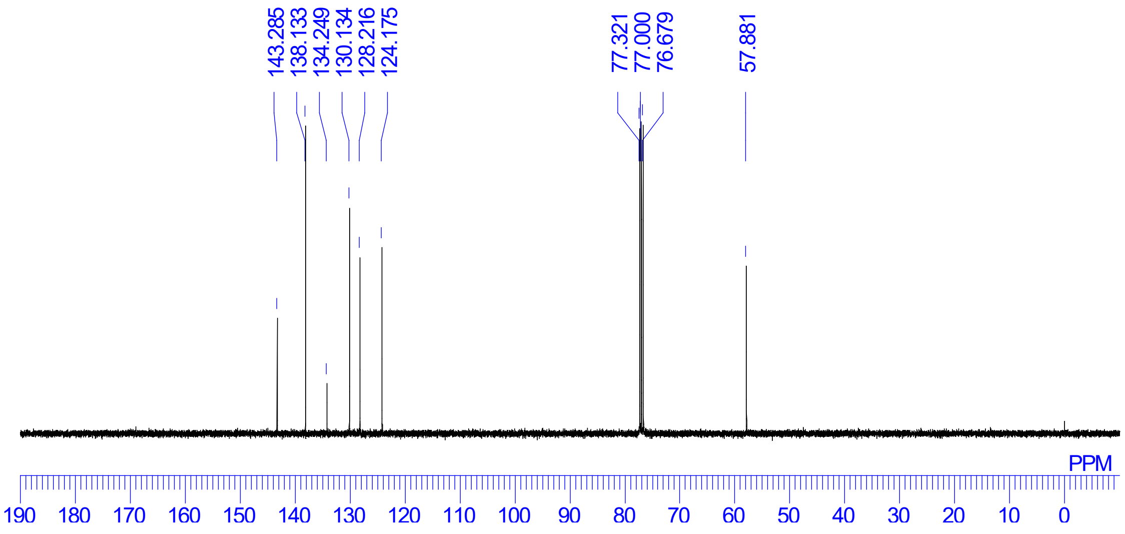

$^{29}Si\{^1H\}$ NMR: (78.7 MHz, $CDCl_3$, Me_4Si in $CDCl_3$ as an external standard)

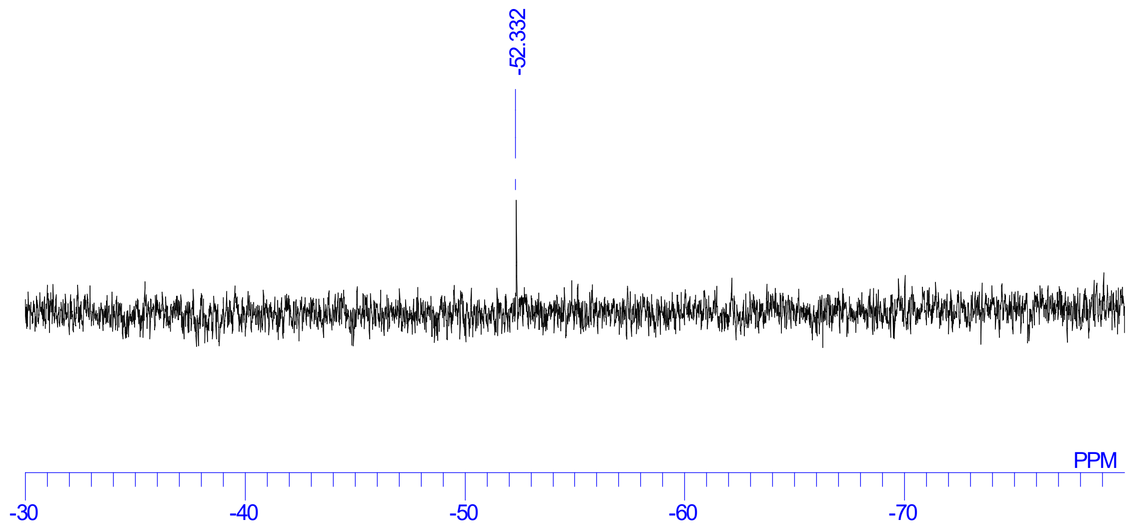

12-chloro-7,12-dihydro-5*H*-12,6-([1,2]benzenomethano)dibenzo[*c*,*f*][1,5]azastannocine 1SnCl

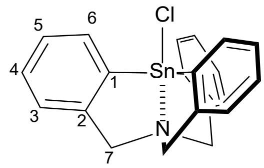

yield 76%; mp 226.0–226.2 °C; IR (KBr) v = 3058 (m), 2909 (m), 1436 (s), 1354 (w), 1261 (w), 1193 (m), 1099 (m), 964 (m), 819 (w), 753 (s) cm^{-1}; ^{1}H NMR (400 MHz, $CDCl_3$) 7.98 (dd, J = 7.2, 1.6 Hz, 3H, 6-H), 7.37 (td, J = 7.4, 1.2 Hz, 3H, 5-H), 7.30 (td, J = 7.4, 1.6 Hz, 3H, 4-H), 7.14 (d, J = 8.0 Hz, 3H, 3-H), 4.03 (s, 6H, 7-H); ^{13}C NMR (100 MHz, $CDCl_3$) 142.61 (s, C-2), 137.07 (s, C-1), 136.17 (d, C-6), 129.58 (d, C-4), 128.64 (d, C-5), 126.08 (d, C-3), 58.24 (t, C-7); $^{119}Sn\{^1H\}$ NMR (147.5 MHz, $CDCl_3$, Me_4Sn in $CDCl_3$ as an external standard) –81.7; MS (EI$^+$, 70 eV) m/z 441 ($[M+2]^+$, 6), 439 (M^+, 16), 437 ($[M–2]^+$, 14), 404 (18), 348 (100), 284 (85), 178 (68); HRMS (EI$^+$, 70 eV) Calculated ($C_{21}H_{18}ClNSn$): 439.0150 (M^+), Found: 439.0145 (M^+).

^{1}H NMR: (400 MHz, $CDCl_3$)

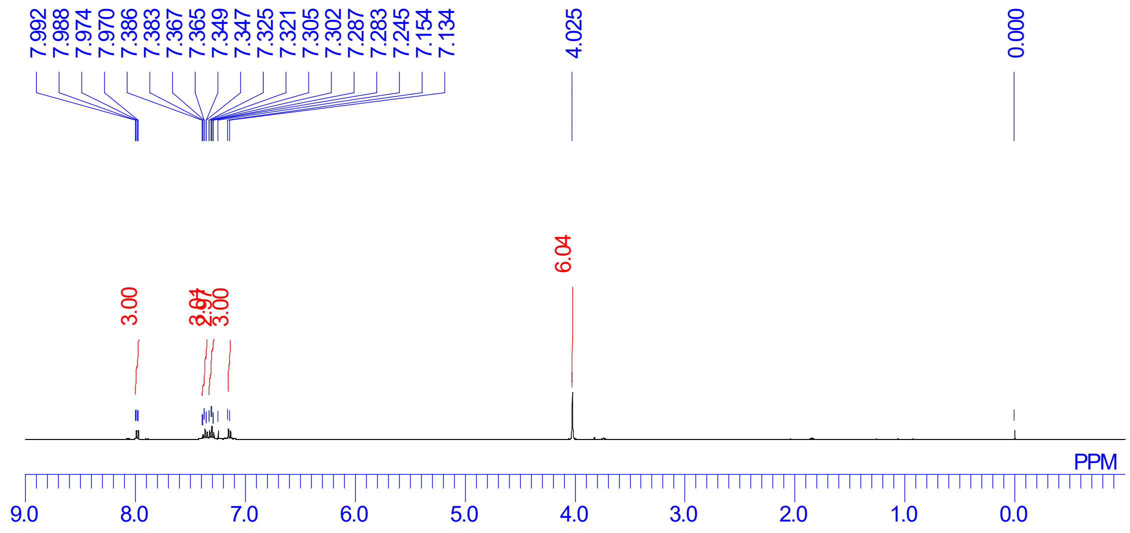

^{13}C NMR: (100 MHz, $CDCl_3$)

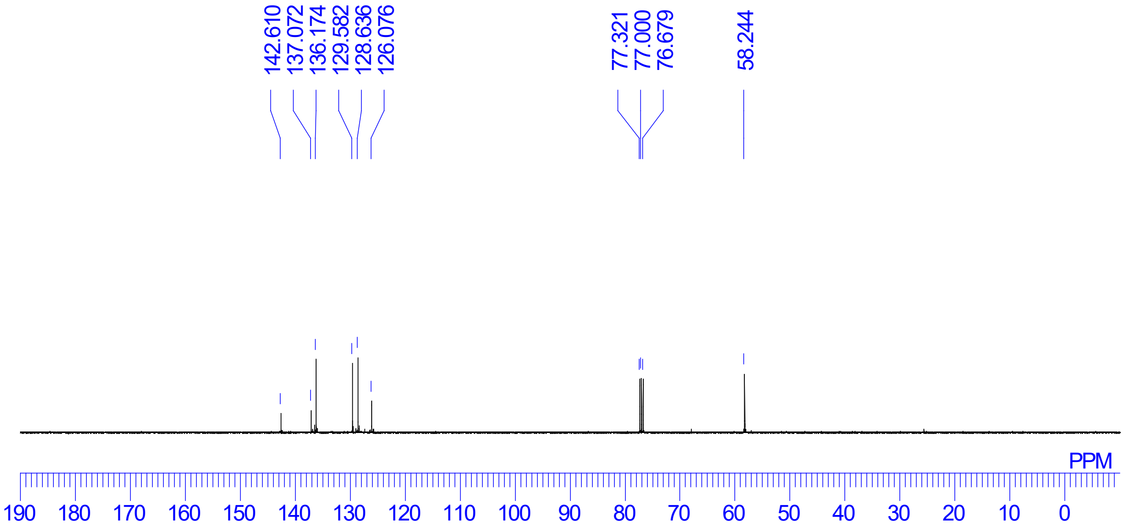

^{119}Sn{^{1}H} NMR: (147.5 MHz, $CDCl_3$, Me_4Sn in $CDCl_3$ as an external standard)

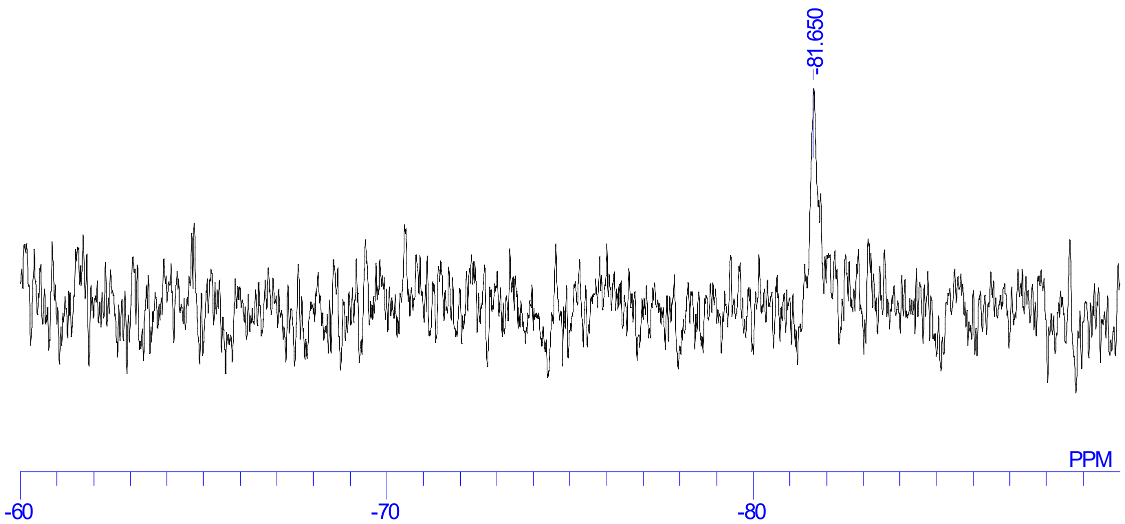

12-chloro-7,12-dihydro-5*H*-12,6-([1,2]benzenomethano)dibenzo[*c*,*f*][1,5]azagermocine 1GeCl

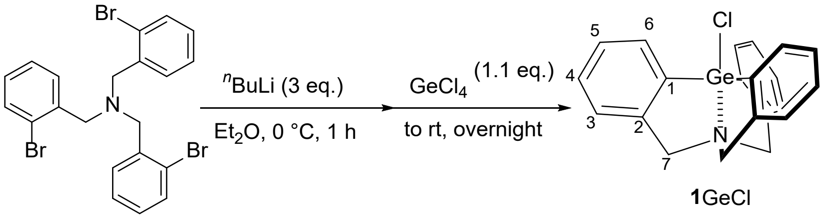

Under N_2 atmosphere, ^{n}BuLi (1.6 M in hexane, 3.75 mL, 6.0 mmol) was titrated to a solution of tris(2-bromobenzyl)amine (1.05 g, 2.0 mmol) in Et_2O (35 mL) at 0 °C and stirred for 1 hour. After dropwisely adding tetrachlorogermane (0.43 g, 2.0 mmol) into the reaction mixture, the reaction temperature was allowed to warm up to room temperature. The reaction mixture was quenched by methanol and the solvents were removed under vacuum to give the crude product. Washing the product with methanol afforded the pure product **1**GeCl as a colorless solid quantitatively (0.79 g, quant.).

mp 239.2–239.8 °C; IR (KBr) *v* = 3056 (w), 2913 (w), 1440 (s), 1353 (w), 1259 (w), 1107 (w), 969 (w), 823 (w), 754 (s) cm^{-1}; ^{1}H NMR (400 MHz, $CDCl_3$) 8.31 (d, *J* = 7.2 Hz, 3H, 6-H), 7.39 (t, *J* = 7.4 Hz, 3H, 5-H), 7.33 (td, *J* = 7.4, 1.6 Hz, 3H, 4-H), 7.14 (d, *J* = 7.6 Hz, 3H, 3-H), 4.04 (s, 6H, 7-H); ^{13}C NMR (100 MHz, $CDCl_3$) 141.69 (s, C-2), 135.30 (d, C-6), 135.26 (s, C-1), 129.86 (d, C-4), 128.55 (d, C-5), 125.12 (d, C-3), 57.20 (t, C-7); MS (EI^{+}, 70 eV) *m*/*z* 395 ([M+2]$^{+}$, 11), 393 (M^{+}, 25), 358 (33), 302 (100), 265 (10), 178 (15), 165 (25); HRMS (EI^{+}, 70 eV) Calculated ($C_{21}H_{18}ClNGe$): 393.0340 (M^{+}), Found: 393.0337 (M^{+}).

^{1}H NMR: (400 MHz, $CDCl_3$)

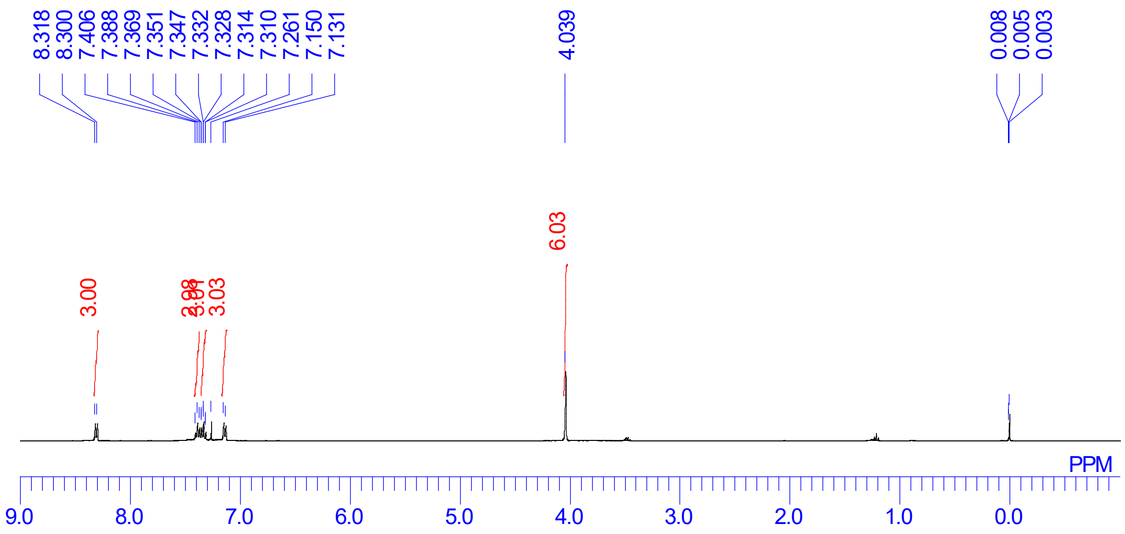

^{13}C NMR: (100 MHz, $CDCl_3$)

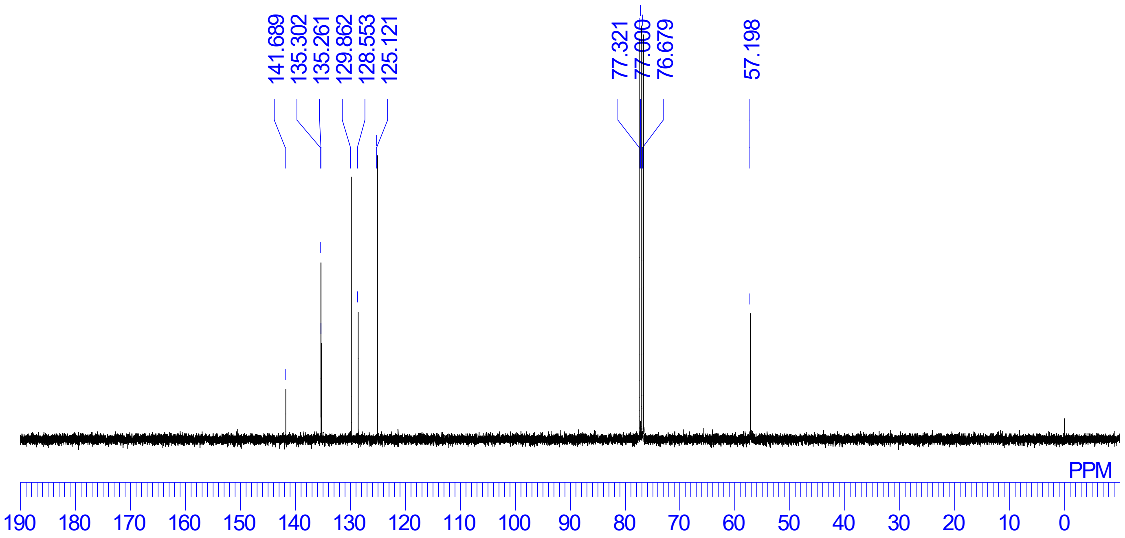

Allylsilatrane 1Si(allyl)

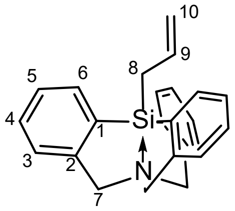

The mixture of a solution of allylmagnesium bromide in Et_2O (0.7 M, 12.3 mL, 8.62 mmol) and **1**SiCl[2] (1.50 g, 4.31 mmol) was heated at 50 °C for 6 h. After the reaction mixture was cooled to room temperature, water (10 mL) was added to quench the reaction and the mixture was extracted with chloroform (3×20 mL). The obtained organic layer was dried over Na_2SO_4 and the solvent was removed in vacuum. The obtained residue was purified by column chromatography (hexane/ethyl acetate = 50/50) on silicagel to give the product as a colorless solid (1.26 g, 83%). mp 110.0–110.8 °C; IR (KBr) v = 3052 (m), 2917 (m), 2847 (m), 1618 (w), 1590 (w), 1439 (s), 1355 (m), 1244 (m), 1117 (s), 975 (s), 932 (w), 894 (s), 730 (m) cm^{-1}; ^1H NMR (400 MHz, $CDCl_3$) 7.97–7.94 (m, 3H, 6-H), 7.27–7.25 (m, 6H, 5-H, 4-H), 7.11–7.08 (m, 3H, 3-H), 6.56–6.45 (m, 1H, 9-H), 5.33 (dd, J = 17.2, 2.4 Hz, 1H, 10-H), 5.11 (dd, J = 10.0, 2.0 Hz, 1H, 10-H), 3.83 (s, 6H, 7-H), 2.72 (d, J = 6.8 Hz, 2H, 8-H); $^{13}C\{^1H\}$ NMR (100 MHz, $CDCl_3$) 145.5 (s, C-2), 140.0 (d, C-9), 136.9 (d, C-6), 136.2 (s, C-1), 129.0 (d, C-4), 127.1 (d, C-5), 125.2 (d, C-3), 114.7 (t, C-10), 56.0 (t, C-7), 24.3 (t, C-8); $^{29}Si\{^1H\}$ NMR (78.7 MHz, $CDCl_3$, Me_4Si in $CDCl_3$ as an external standard) −50.2; HRMS (DART$^+$) Calculated ($C_{24}H_{24}NSi$): 354.1673 ($[M+H]^+$), Found: 354.1663.

^1H NMR: (400 MHz, $CDCl_3$)

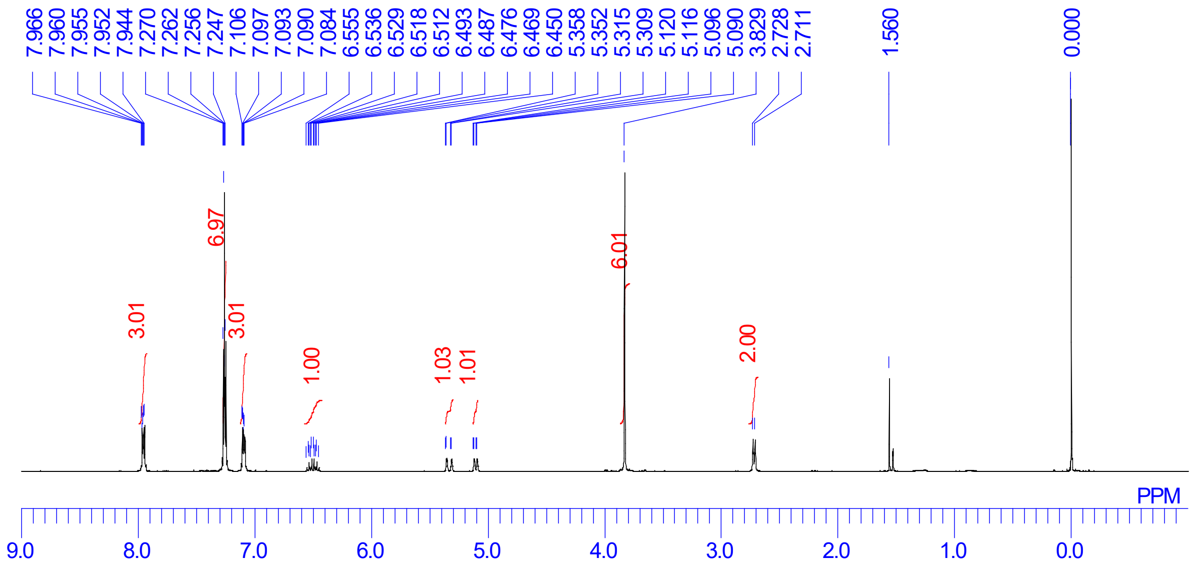

$^{13}C\{^1H\}$ NMR: (100 MHz, $CDCl_3$)

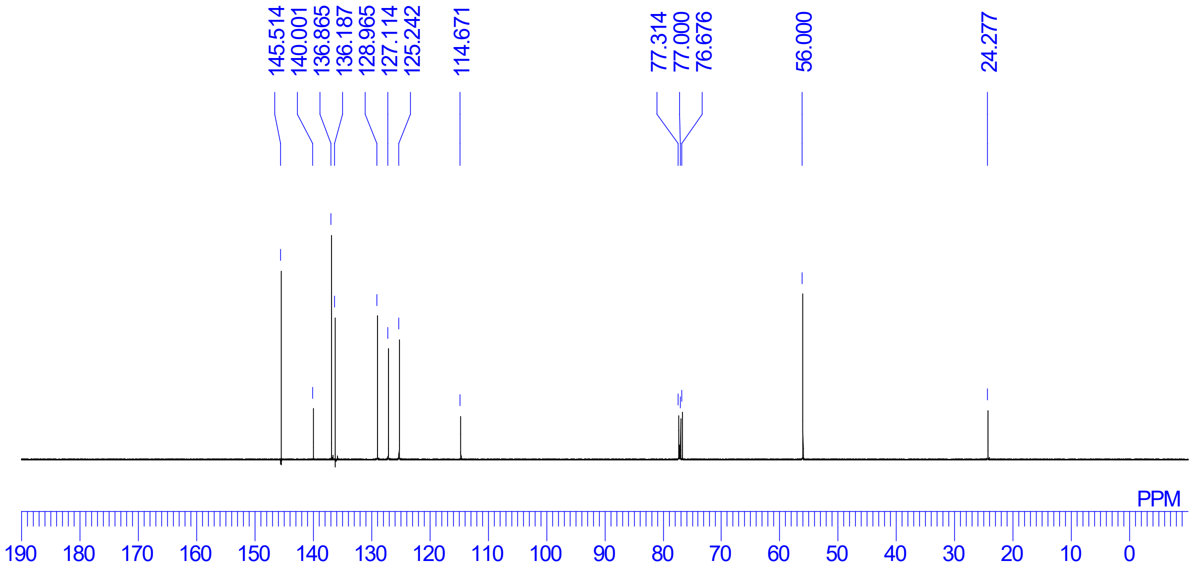
145.514
140.001
136.865
136.187
128.965
127.114
125.242
114.671
77.314
77.000
76.676
56.000
24.277
PPM
190 180 170 160 150 140 130 120 110 100 90 80 70 60 50 40 30 20 10 0

$^{29}Si\{^1H\}$ NMR: (78.7 MHz, $CDCl_3$, Me_4Si in $CDCl_3$ as an external standard)

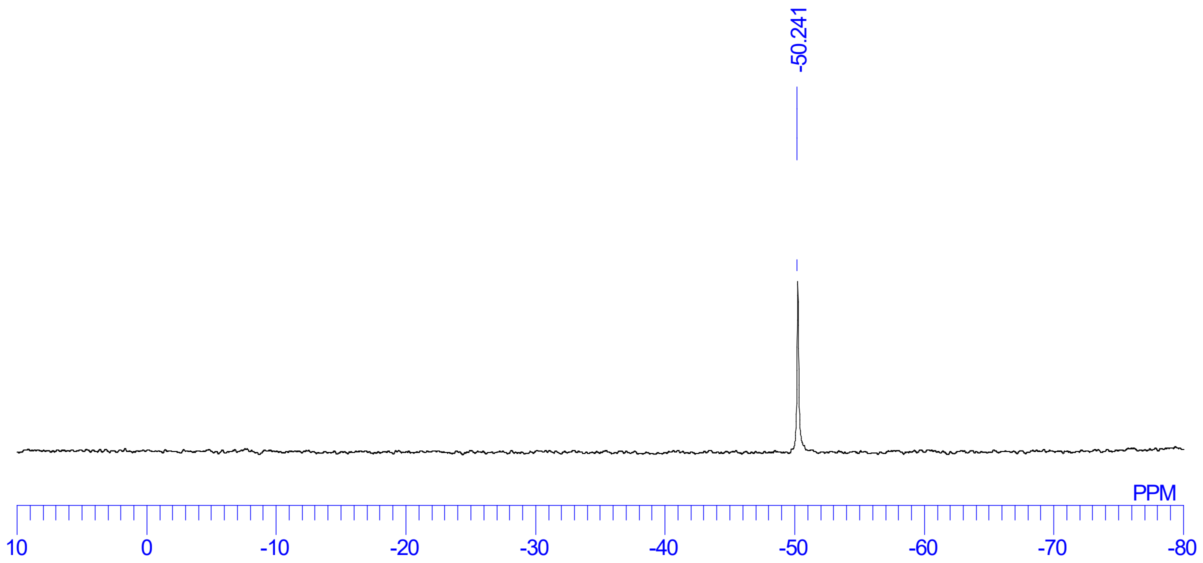

Allylgermatrane 1Ge(allyl)

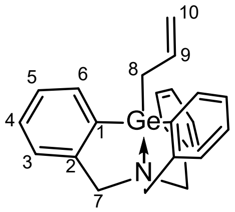

The mixture of a solution of allylmagnesium bromide in Et_2O (0.7 M, 7.9 mL, 5.54 mmol) and **1**GeCl[2] (0.96 g, 2.77 mmol) was heated at 50 °C for 9 h. After the reaction mixture was cooled to room temperature, water (10 mL) was added to quench the reaction and the mixture was extracted with chloroform (3×20 mL). The obtained organic layer was dried over Na_2SO_4 and the solvent was removed in vacuum. The obtained residue was purified by column chromatography (hexane/ethyl acetate = 50/50) on silicagel to give the product as a colorless solid (0.49 g, 50%).

mp 115.2–116.0 °C; IR (KBr) ν = 3049 (w), 2878 (w), 2836 (w), 1622 (m), 1438 (s), 1358 (m), 1308 (m), 1197 (w), 1114 (m), 976 (s), 887 (s), 744 (s) cm^{-1}; ^1H NMR (400 MHz, $CDCl_3$) 7.84–7.82 (m, 3H, 6-H), 7.26–7.21 (m, 6H, 5-H, 4-H), 7.10–7.08 (m, 3H, 3-H), 6.59–6.49 (m, 1H, 9-H), 5.36 (dd, J = 17.0, 2.2 Hz, 1H, 10-H), 5.11 (dd, J = 10.0, 2.0 Hz, 1H, 10-H), 3.84 (s, 6H, 7-H), 2.80 (d, J = 7.2 Hz, 2H, 8-H); $^{13}C\{^1H\}$ NMR (100 MHz, $CDCl_3$) 144.3 (s, C-2), 138.9 (d, C-9), 137.5 (s, C-1), 135.2 (d, C-6), 128.6 (d, C-4), 127.2 (d, C-5), 126.2 (d, C-3), 114.4 (t, C-10), 55.6 (t, C-7), 21.4 (t, C-8); HRMS (DART$^+$) Calculated ($C_{24}H_{24}NGe$): 400.1115 ($[M+H]^+$), Found: 400.1112; Analysis Calculated ($C_{24}H_{23}NGe$): C, 72.41; H, 5.82; N, 3.52; Ge, 18.24, Found: C, 72.34; H, 6.00; N, 3.44.

^{1}H NMR: (400 MHz, $CDCl_3$)

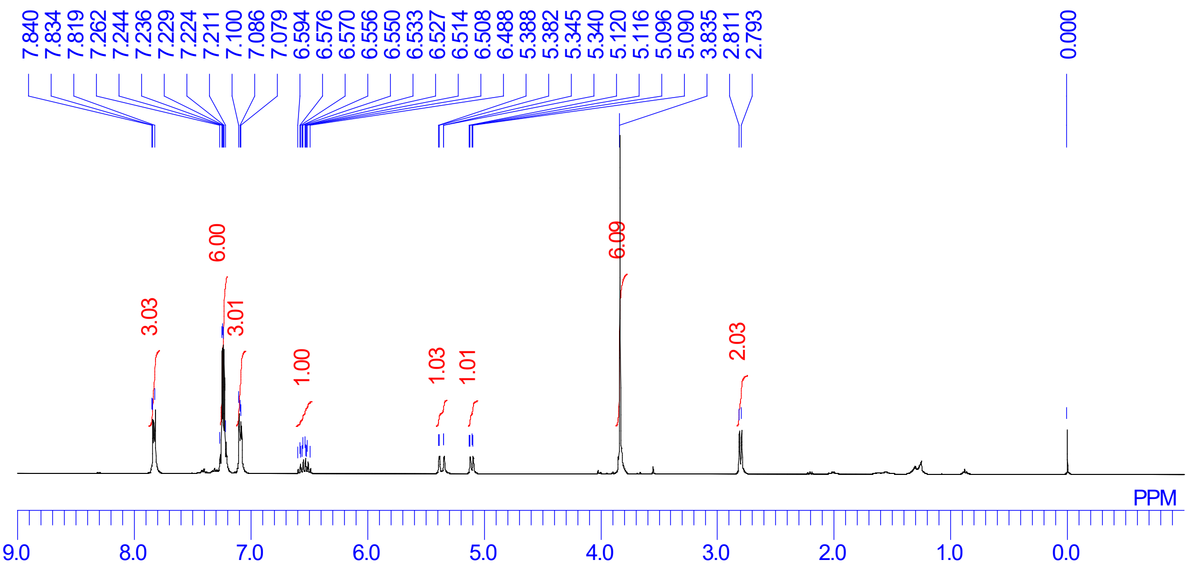

$^{13}C\{^{1}H\}$ NMR: (100 MHz, $CDCl_3$)

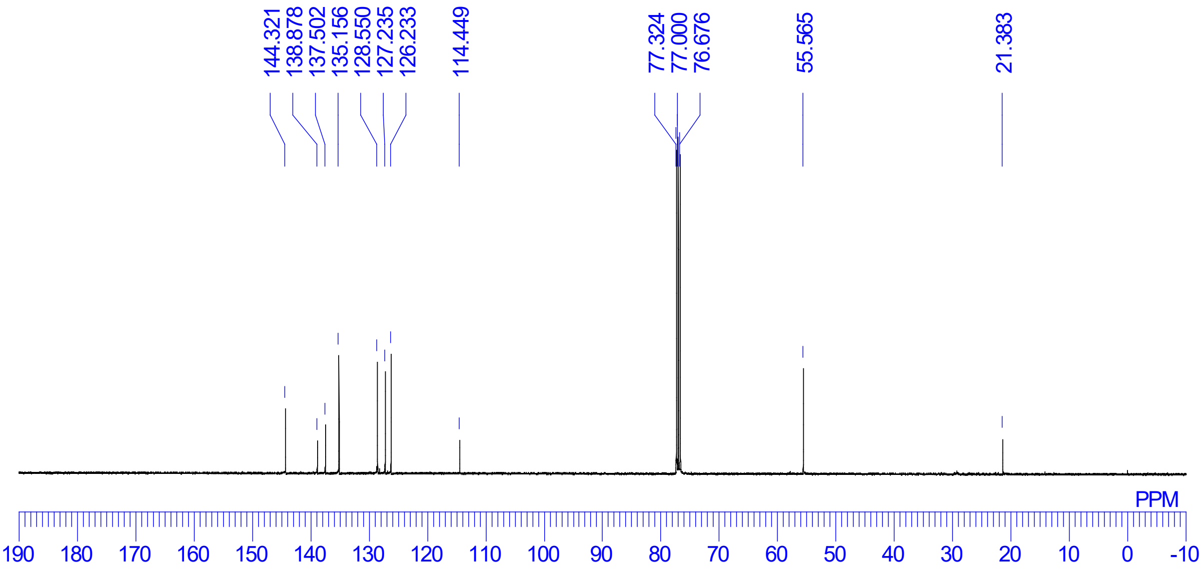

Allylstannatrane 1Sn(allyl)

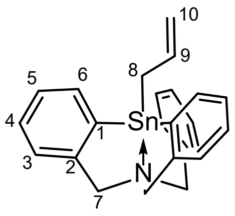

The mixture of a solution of allylmagnesium bromide in Et_2O (0.7 M, 5.7 mL, 4.0 mmol) and **1**SnCl[2] (0.88 g, 2.0 mmol) was heated at 50 °C for 6 h. After the reaction mixture was cooled to room temperature, the mixture was extracted with chloroform (3×20 mL). The obtained organic layer was dried over Na_2SO_4 and the solvent was

removed in vacuum. The obtained residue was purified by column chromatography (hexane/ethyl acetate = 60:40) on silicagel to give the product as a colorless solid (0.32 g, 36%).

mp 125.1–125.7 °C; IR (KBr) v = 3049 (m), 2910 (w), 2843 (m), 1618 (s), 1434 (s), 1397 (w), 1357 (m), 1302 (m), 1193 (m), 1106 (s), 1035 (m), 969 (s), 867 (s), 819 (m), 748 (s) cm^{-1}; ^{1}H NMR (400 MHz, $CDCl_3$) 7.77 (dd, $J_{H\text{–}H}$ = 6.6, 1.8 Hz, d, $^{3}J_{Sn\text{–}H}$ = 49.8 Hz, 3H, 6-H), 7.28–7.21 (m, 6H, 5-H, 4-H), 7.09 (d, $J_{H\text{–}H}$ = 6.8 Hz, d, $^{4}J_{Sn\text{–}H}$ = 22.4 Hz, 3H, 3-H), 6.65–6.53 (m, 1H, 9-H), 5.30–5.21 (m, 1H, 10-H), 4.97–4.90 (m, 1H, 10-H), 3.84 (s, 6H, 7-H), 2.67 (dd, $J_{H\text{–}H}$ = 8.4, 1.2 Hz, d, $^{2}J_{Sn\text{–}H}$ = 62.0 Hz, 2H, 8-H); $^{13}C\{^{1}H\}$ NMR (100 MHz, $CDCl_3$) 144.1 (s, d, $^{2}J_{Sn\text{–}C}$ = 29.3 Hz, C-2), 139.23 (s, d, $^{1}J_{119Sn\text{–}C}$ = 563.5 Hz, d, $^{1}J_{117Sn\text{–}C}$ = 538.2 Hz, C-1), 139.21 (d, d, $^{2}J_{Sn\text{–}C}$ = 40.4 Hz, C-9), 137.0 (d, d, $^{2}J_{Sn\text{–}C}$ = 44.5 Hz, C-6), 128.5 (d, d, $^{4}J_{Sn\text{–}C}$ = 10.0 Hz, C-4), 127.5 (d, d, $^{3}J_{Sn\text{–}C}$ = 54.6 Hz, C-5), 126.5 (d, d, $^{3}J_{Sn\text{–}C}$ = 42.5 Hz, C-3), 110.9 (t, d, $^{3}J_{Sn\text{–}C}$ = 47.5 Hz, C-10), 57.1 (t, d, $^{3}J_{Sn\text{–}C}$ = 30.4 Hz, C-7), 17.7 (t, d, $^{1}J_{119Sn\text{–}C}$ = 271.1 Hz, d, $^{1}J_{117Sn\text{–}C}$ = 260.0 Hz, C-8); $^{119}Sn\{^{1}H\}$ NMR (147.5 MHz, $CDCl_3$, Me_4Sn in $CDCl_3$ as an external standard) −159.9; HRMS (DART^{+}) Calculated ($C_{24}H_{24}NSn$): 446.0925 ($[M+H]^{+}$), Found: 446.0924; Analysis Calculated ($C_{24}H_{23}NSn$): C, 64.90; H, 5.22; N, 3.15; Sn, 26.73, Found: C, 64.98; H, 5.29; N, 3.12.

^{1}H NMR: (400 MHz, $CDCl_3$)

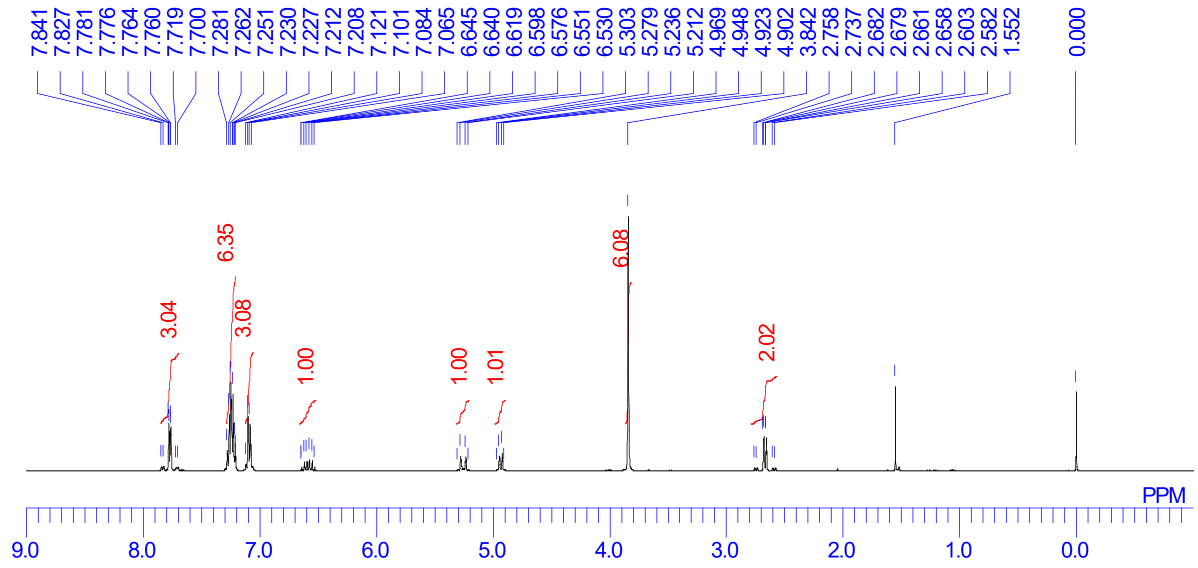

$^{13}C\{^1H\}$ NMR: (100 MHz, $CDCl_3$)

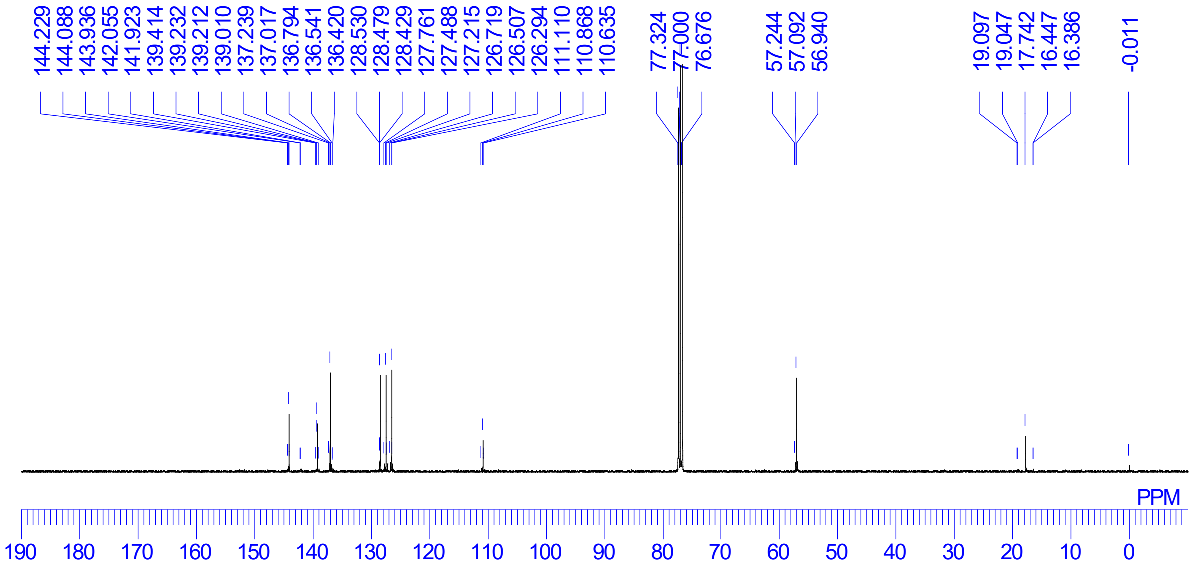

$^{119}Sn\{^1H\}$ NMR: (147.5 MHz, $CDCl_3$, Me_4Sn in $CDCl_3$ as an external standard)

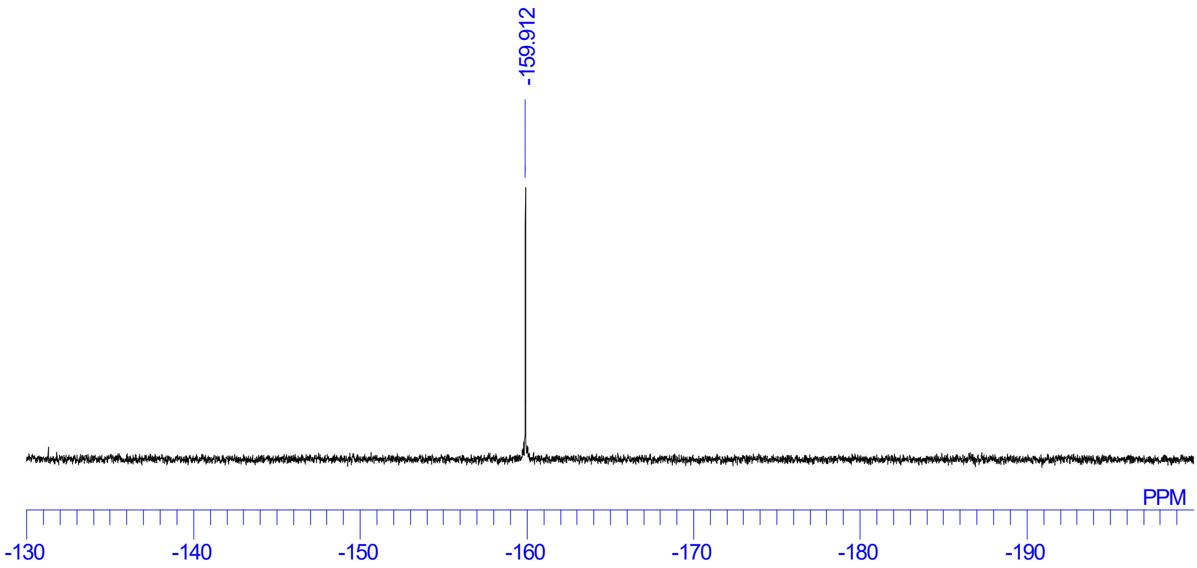

Methallylsilatrane 1Si(methallyl)

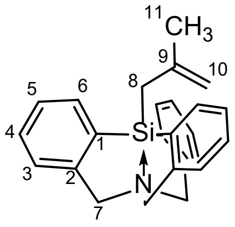

To a solution of lithium wires (69 mg, 10.0 mmol) and 4,4'-di-*tert*-butylbiphenyl (0.53 g, 2.0 mmol) in THF (10 mL) were added **1**SiCl[2] (0.88 g, 2.0 mmol). The reaction mixture was stirred at room temperature for 15 h until the color of solution turned dark green. The resulting mixture was added dropwise to a solution of 3-chloro-2-methyl-

1-propene (0.60 mL, 6.0 mmol) in THF (10 mL) at 0 °C. The reaction mixture was stirred to room temperature for 3 h. Water (10 mL) was added to quench the reaction and the mixture was extracted with chloroform (3×20 mL). The obtained organic layer was dried over Na_2SO_4 and the solvent was removed in vacuum. The obtained residue was purified by column chromatography (hexane/ethyl acetate = 80/20) on silicagel. Further purification was conducted by a recycle GPC to give the product as a colorless solid (0.12 g, 17%).

mp 105.8–106.5 °C; IR (KBr) v = 3051 (w), 2912 (w), 2845 (m), 1439 (s), 1356 (w), 1244 (w), 1117 (s), 1069 (m), 975 (m), 884 (s), 826 (w), 752 (s) cm^{-1}; ^{1}H NMR (400 MHz, $CDCl_3$) 7.89–7.87 (m, 3H, 6-H), 7.26–7.21 (m, 6H, 5-H, 4-H), 7.08–7.06 (m, 3H, 3-H), 4.91 (s, 1H, 10-H), 4.53 (s, 1H, 10-H), 3.83 (s, 6H, 7-H), 2.41 (s, 2H, 8-H), 2.08 (s, 3H, 11-H); $^{13}C\{^{1}H\}$ NMR (100 MHz, $CDCl_3$) 145.6 (s, C-9), 145.5 (s, C-2), 137.3 (d, C-6), 136.1 (s, C-1), 128.9 (d, C-4), 126.9 (d, C-5), 125.1 (d, C-3), 115.7 (t, C-10), 56.2 (t, C-7), 29.2 (q, C-11), 25.8 (t, C-8); $^{29}Si\{^{1}H\}$ NMR: (78.7 MHz, $CDCl_3$, Me_4Si in $CDCl_3$ as an external standard) −45.1; HRMS (DART$^+$); Calculated ($C_{25}H_{26}NSi$): 368.1829 ($[M+H]^+$), Found: 368.1824.

^{1}H NMR: (400 MHz, $CDCl_3$)

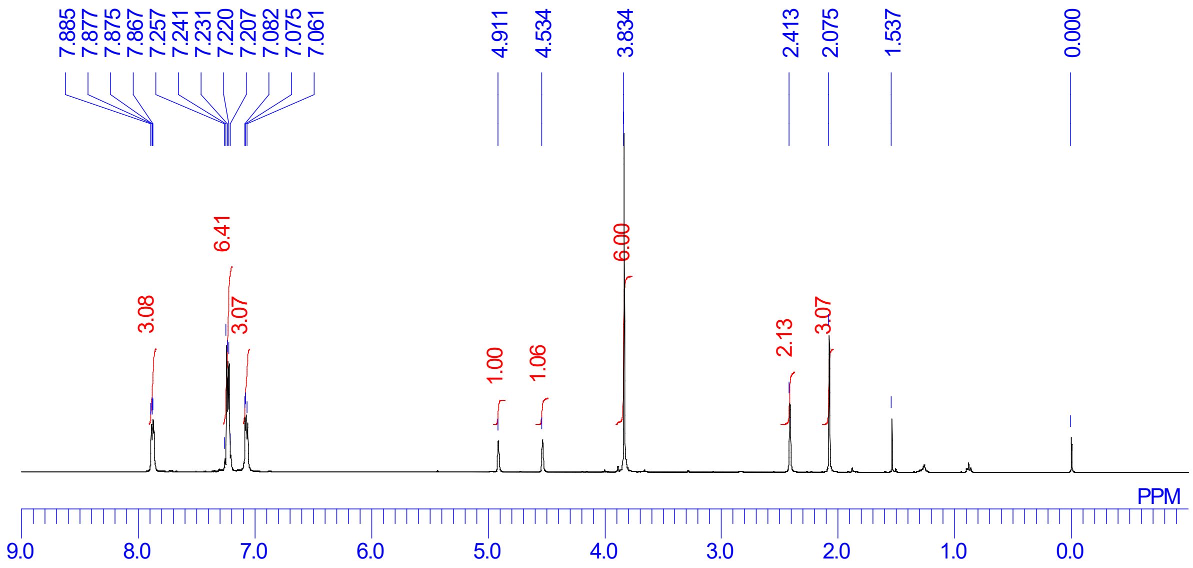

$^{13}C\{^1H\}$ NMR: (100 MHz, $CDCl_3$)

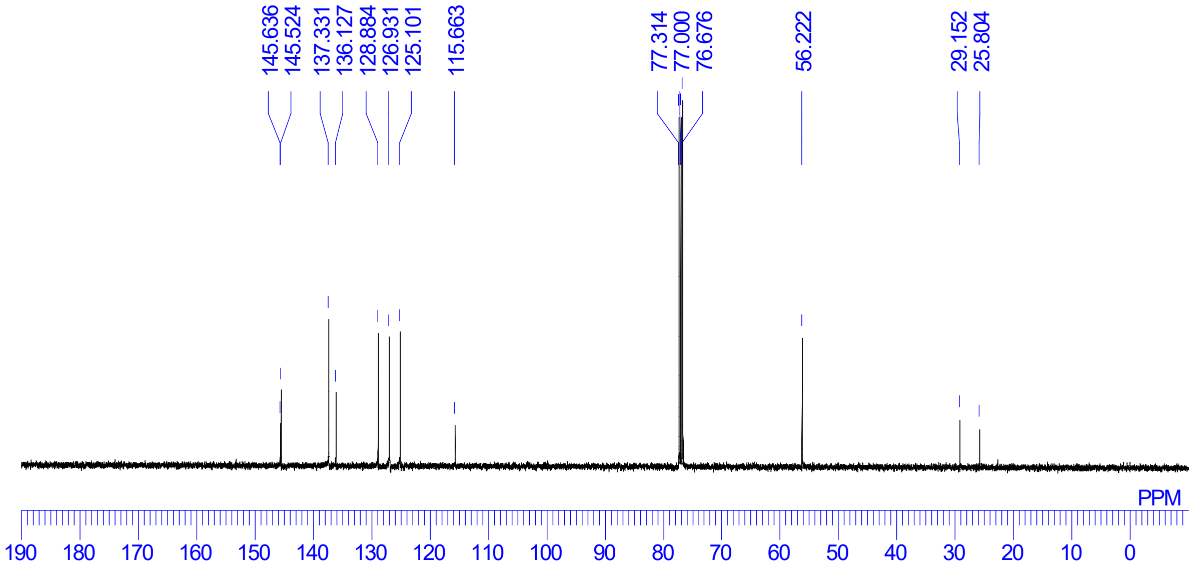

$^{29}Si\{^1H\}$ NMR: (78.7 MHz, $CDCl_3$, Me_4Si in $CDCl_3$ as an external standard)

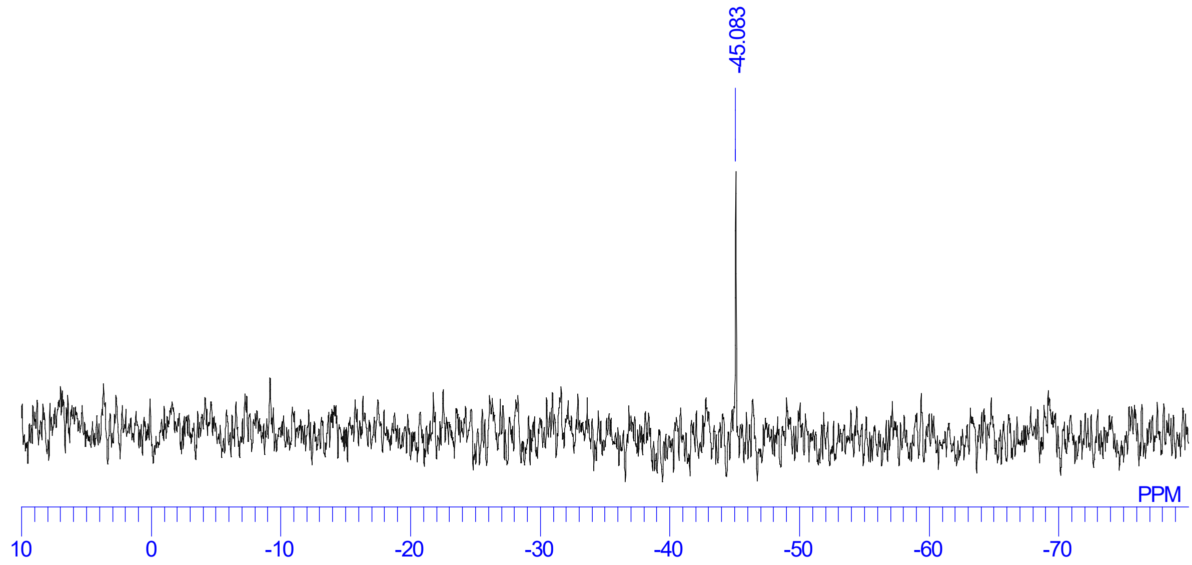

2-Phenylallylsilatrane 1Si(2-phenylallyl)

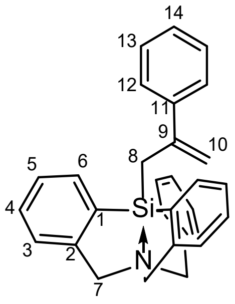

To a solution of lithium wires (69 mg, 10.0 mmol) and 4,4'-di-*tert*-butylbiphenyl (1.07 g, 4.0 mmol) in THF (10

mL) were added **1**SiCl^2 (0.88 g, 2.0 mmol). The reaction mixture was stirred at room temperature for 15 h until the color of solution turned dark green. The resulting solution was added dropwise to a solution of *α*-(bromomethyl)styrene (0.86 mL, 6.0 mmol) in THF (10 mL) at 0 °C. The reaction mixture was stirred to room temperature for 3 h. Water (10 mL) was added to quench the reaction and the mixture was extracted with chloroform (3×20 mL). The obtained organic layer was dried over Na_2SO_4 and the solvent was removed in vacuum. The obtained residue was purified by column chromatography (hexane/ethyl acetate = 80/20) on silicagel. Further purification was conducted by a recycle GPC to give the product as a colorless solid (0.035 g, 4%).

mp 96.8–97.2 °C; IR (KBr) ν = 3052 (w), 2917 (w), 2848 (w), 1735 (w), 1591 (w), 1492 (m), 1439 (s), 1357 (m), 1304 (m), 1244 (m), 1157 (w), 1117 (s), 1068 (s), 975 (m), 908 (m), 825 (m), 745 (s) cm^{-1}; ^{1}H NMR (400 MHz, $CDCl_3$) 7.92 (d, *J* = 7.6 Hz, 3H, 6-H), 7.77 (dd, *J* = 7.0, 1.4 Hz, 2H, 12-H), 7.45 (td, *J* = 8.0, 2.1 Hz, 2H, 13-H), 7.34 (td, *J* = 6.9, 1.6 Hz, 1H, 14-H), 7.28–7.21 (m, 6H, 4-H, 5-H), 7.11 (d, *J* = 6.8 Hz, 3H, 3-H), 5.52 (s, 1H, 10-H), 4.96 (s, 1H, 10-H), 3.89 (s, 6H, 7-H), 2.87 (s, 2H, 8-H); ^{13}C{^{1}H} NMR (100 MHz, $CDCl_3$) 147.3 (s, C-9), 145.9 (s, C-11), 145.5 (s, C-2), 137.4 (d, C-6), 135.9 (s, C-1), 129.0 (d, C-4), 128.2 (d, C-13), 127.1 (d, C-5), 127.0 (d, C-14), 126.1 (d, C-12), 125.1 (d, C-3), 118.6 (t, C-10), 56.4 (t, C-7), 23.0 (t, C-8); ^{29}Si{^{1}H} NMR: (78.7 MHz, $CDCl_3$, Me_4Si in $CDCl_3$ as an external standard) −45.3; HRMS (DART$^+$) Calculated ($C_{30}H_{28}NSi$): 430.1986 ([M+H]$^+$), Found: 430.1995.

^{1}H NMR: (400 MHz, $CDCl_3$)

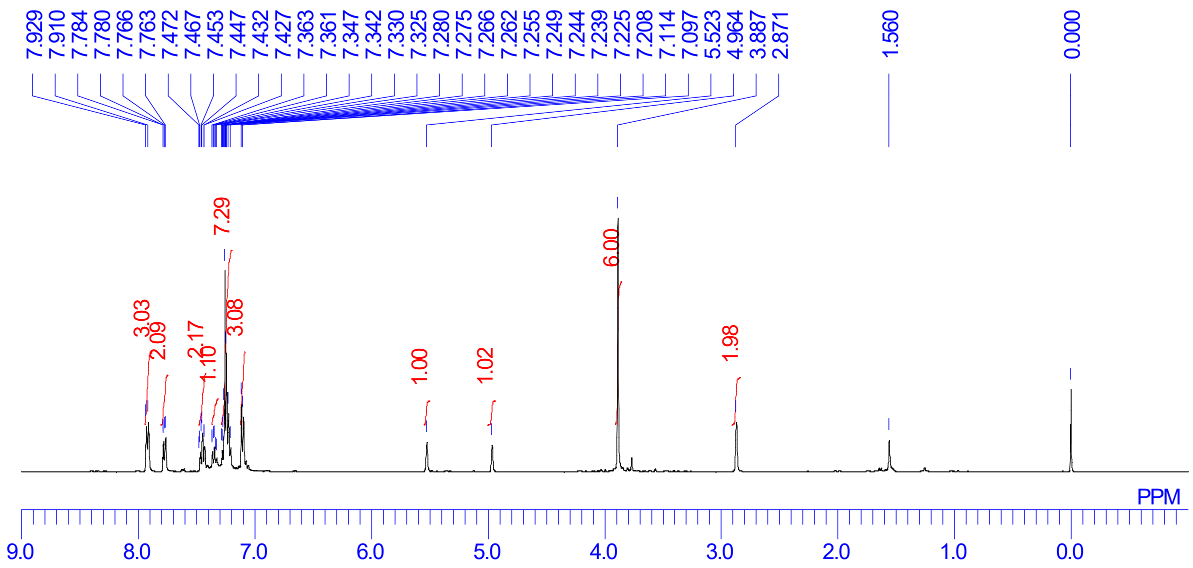

$^{13}C\{^1H\}$ NMR: (100 MHz, $CDCl_3$)

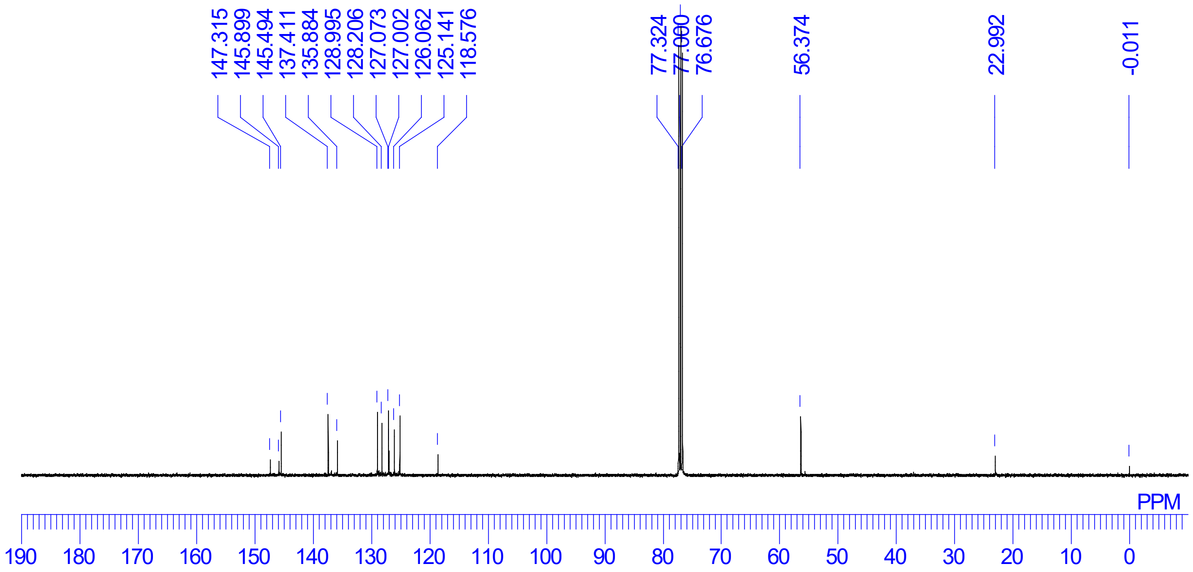

$^{29}Si\{^1H\}$ NMR: (78.7 MHz, $CDCl_3$, Me_4Si in $CDCl_3$ as an external standard)

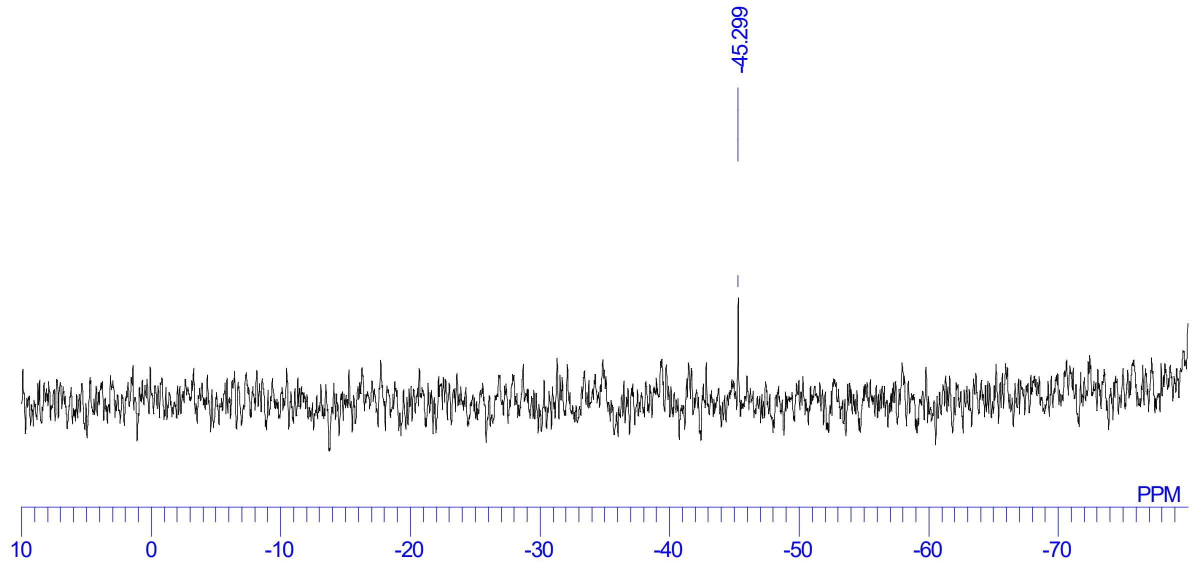

(1*S,2*R**)-1-Methoxy-1,2-diphenylpent-4-en-2-ol *syn*-3a**

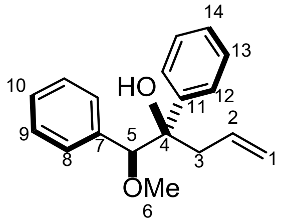

To a mixture of allyl bromide (0.59 mL, 6.8 mmol) and indium (0.78 g, 6.8 mmol) in THF (10 mL) was added benzoin methyl ether **2a** (1.28 g, 5.6 mmol). After the reaction mixture was stirred for 1 h at room temperature, methanol (5 mL) was added to the mixture. The residue was filtrated with a celite-pad and the solvent was removed

in vacuum. The obtained residue was purified by column chromatography (hexane/ethyl acetate = 80/20) on silica gel to give product as a colorless solid (1.51 g, quant.). The NMR data were consistent with the data previously reported.[13]

^{1}H NMR (400 MHz, $CDCl_3$) 7.21–7.12 (m, 8H), 6.96 (dd, J = 7.8, 1.8 Hz, 2H), 5.68–5.58 (m, 1H), 5.11 (dd, J = 17.4, 1.8 Hz, 1H), 5.03 (dd, J = 10.4, 2.0 Hz, 1H), 4.30 (s, 1H), 3.26 (s, 3H), 2.87 (d, J = 8.0 Hz, 1H), 2.84 (s, 1H); $^{13}C\{^{1}H\}$ NMR (100 MHz, $CDCl_3$) 142.0 (s), 136.7 (s), 133.7 (d), 128.6 (d), 127.6 (d), 127.38 (d), 127.37 (d), 126.55 (d), 126.47 (d), 118.6 (t), 89.6 (d), 78.1 (s), 57.4 (q), 42.7 (t).

^{1}H NMR: (400 MHz, $CDCl_3$)

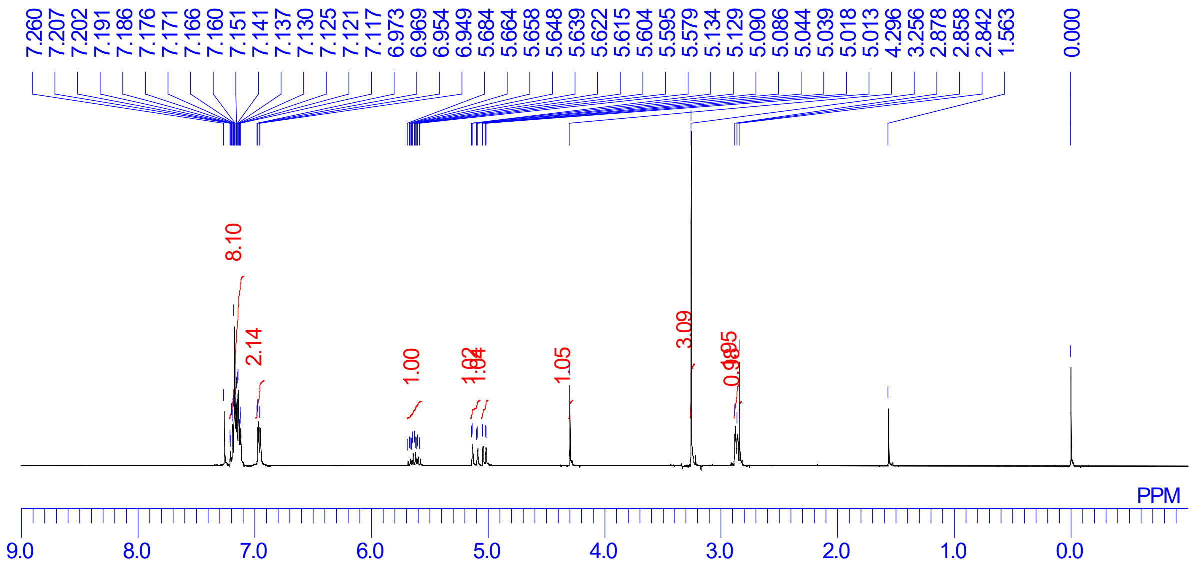

$^{13}C\{^{1}H\}$ NMR: (100 MHz, $CDCl_3$)

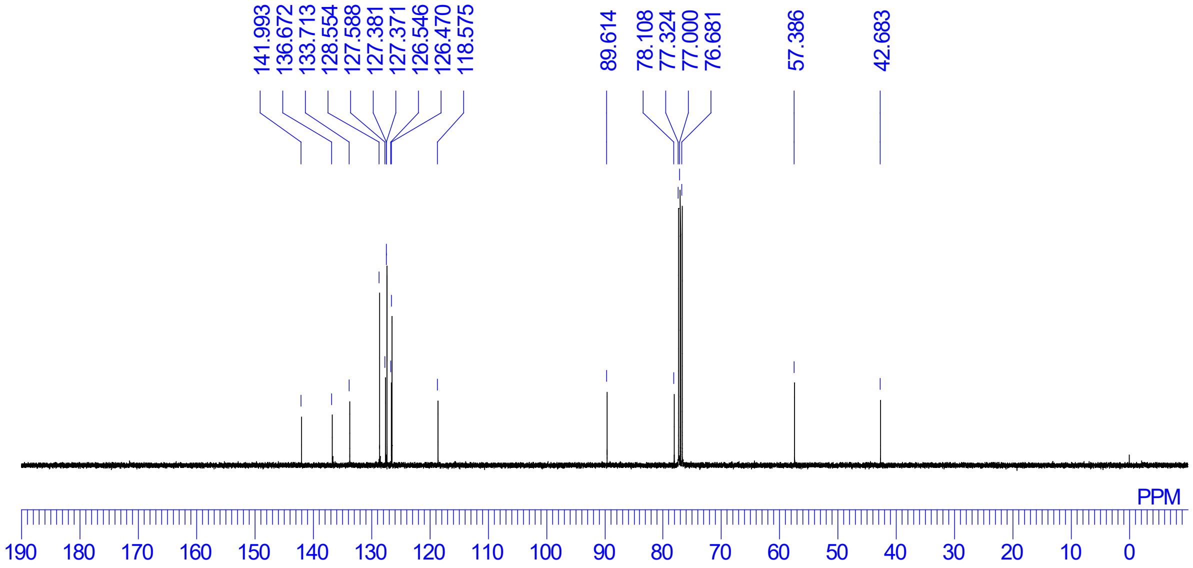

(1*S,2*S**)-1-Methoxy-1,2-diphenylpent-4-en-2-ol *anti*-3a**

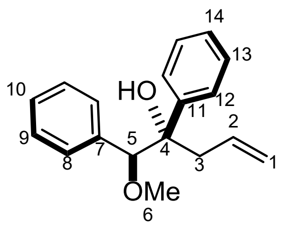

In a nitrogen-filled glovebox, to a mixture of $BF_3 \cdot Et_2O$ (142 mg, 1.0 mmol) and benzoin methyl ether **2a** (226 mg, 1.0 mmol) in dichloromethane (10 mL) was added **1**Si(allyl) (354 mg, 1.0 mmol). After the reaction mixture was stirred for 3 h at room temperature, methanol (10 mL) was added to the mixture. The residue was evaporated to give a crude mixture, which was analyzed by ^{1}H NMR to obtain the yield and diastereoselectivity using 1,1,2,2-tetrachloroethane as an internal standard (NMR yield: 97%, *syn*/*anti* = 6/94). The obtained residue was purified by column chromatography (hexane/ethyl acetate = 30/70) on silica gel to give product as a colorless oil (237 mg, 88%). The NMR data were consistent with the data previously reported.[13]

^{1}H NMR (400 MHz, $CDCl_3$) 7.29–7.17 (m, 8H), 7.00 (d, J = 8.0 Hz, 2H), 5.63–5.52 (m, 1H), 5.04–4.97 (m, 2H), 4.28 (s, 1H), 3.22 (s, 3H), 2.92 (s, 1H), 2.68 (dd, J = 14.8, 6.4 Hz, 1H), 2.56 (dd, J = 14.4, 7.6 Hz, 1H); ^{13}C{^{1}H} NMR (100 MHz, $CDCl_3$) 142.5 (s), 136.6 (s), 133.6 (d), 128.7 (d), 127.9 (d), 127.6 (d), 127.5 (d), 126.8 (d), 126.7 (d), 118.5 (t), 90.4 (d), 78.2 (s), 57.4 (q), 40.8 (t).

^{1}H NMR: (400 MHz, $CDCl_3$)

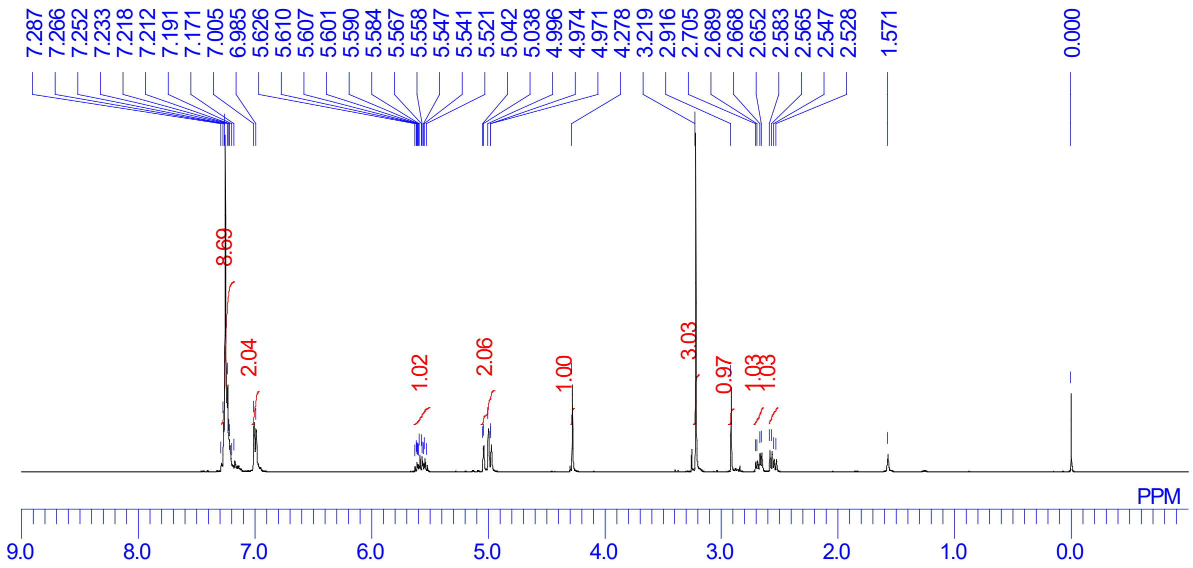

$^{13}C\{^{1}H\}$ NMR: (100 MHz, $CDCl_3$)

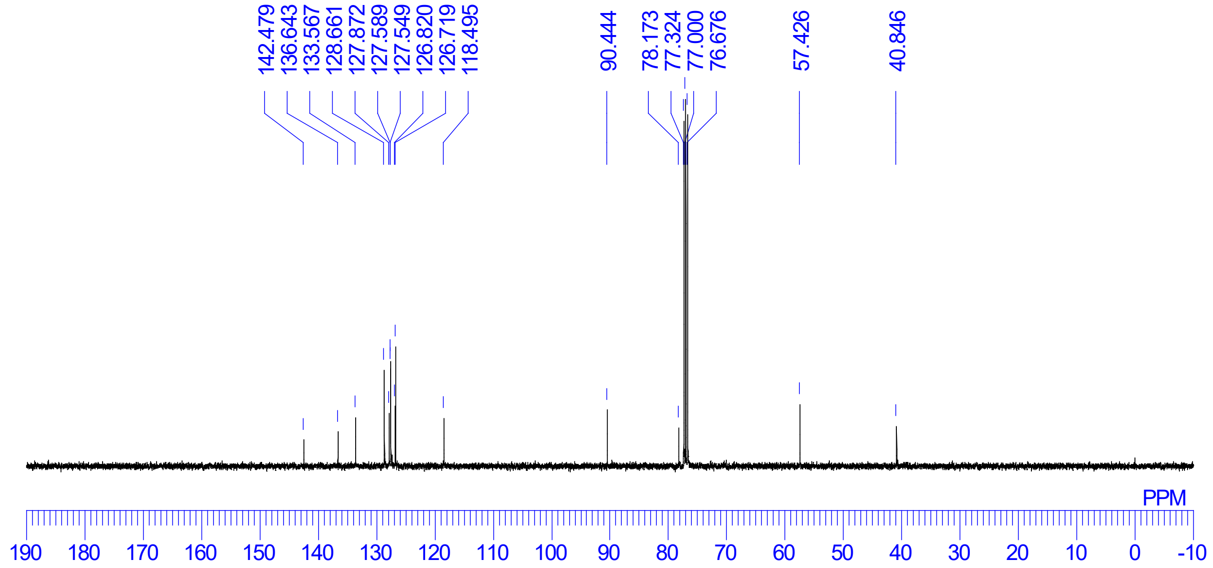

[{(1*S,2S*)-1-Methoxy-1,2-diphenylpent-4-en-2-yl}oxy]triphenylsilane *anti*-3a′**

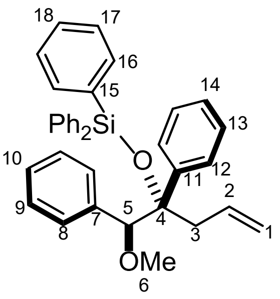

To a stirred solution of (1*S**,2*S**)-1-methoxy-1,2-diphenylpent-4-en-2-ol *anti*-**3a** (254 mg, 0.95 mmol) in THF (5 mL) was added ^{n}BuLi in hexane (2.6 M, 0.40 mL, 1.04 mmol) at −78 °C. After the reaction mixture was stirred for 30 min to room temperature, chlorotriphenylsilane (558 mg, 1.89 mmol) was added to the mixture. The resulting mixture was heated to reflux for 9 h. The reaction mixture was cooled to room temperature, NH_4Cl aq. (10 mL) was added to quench the reaction and the mixture was extracted with ethyl acetate (3×10 mL). The organic layer was dried over $MgSO_4$ and the solvent was removed in vacuum. The obtained residue was purified by column chromatography (hexane/ethyl acetate = 80/20) on silicagel. Further purification was conducted by a recycle GPC to give the product as a colorless solid (239 mg, 48%).

mp 143.5–144.0 °C; IR (KBr) v = 3068 (w), 2950 (w), 2871 (m), 1492 (w), 1428 (m), 1266 (w), 1211 (w), 1112 (s), 1064 (m), 998 (m), 959 (m), 914 (m), 727 (s), 700 (s) cm^{-1}; ^{1}H NMR (400 MHz, $CDCl_3$) 7.70 (dd, J = 8.0, 1.6 Hz, 6H), 7.42–7.33 (m, 9H), 7.23–7.14 (m, 4H), 7.11–7.08 (m, 4H), 6.71 (d, J = 6.8 Hz, 2H), 5.62–5.52 (m, 1H), 4.74 (d, J = 10.4 Hz, 1H), 4.41 (d, J = 17.2 Hz, 1H), 4.23 (s, 1H), 2.89 (dd, J = 15.2, 6.8 Hz, 1H), 2.57 (dd, J = 15.2, 6.4 Hz, 1H), 2.45 (s, 3H); $^{13}C\{^{1}H\}$ NMR (100 MHz, $CDCl_3$) 142.4 (s), 137.1 (s), 136.7 (s), 135.7 (d, Two signals were overlapped.), 133.5 (d), 129.3 (d), 129.0 (d), 127.8 (d), 127.50 (d), 127.48 (d), 127.2 (d), 126.8 (d), 118.1 (t), 90.6 (d), 84.6 (s), 55.2 (q), 35.6 (t); $^{29}Si\{^{1}H\}$ NMR (78.7 MHz, $CDCl_3$, Me_4Si in $CDCl_3$ as an external standard) −20.3; HRMS (MALDI-TOF MS) Calculated ($C_{36}H_{34}O_2NaSi$): 549.2220 ($[M+Na]^+$), Found: 549.2227.

^{1}H NMR: (400 MHz, $CDCl_3$)

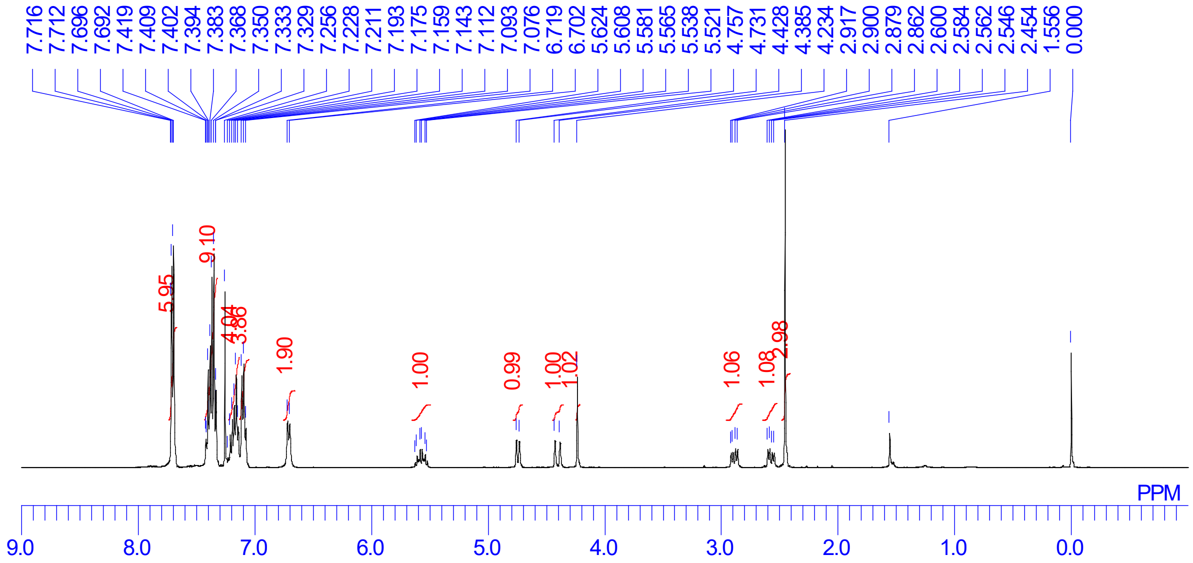

$^{13}C\{^1H\}$ NMR: (100 MHz, $CDCl_3$)

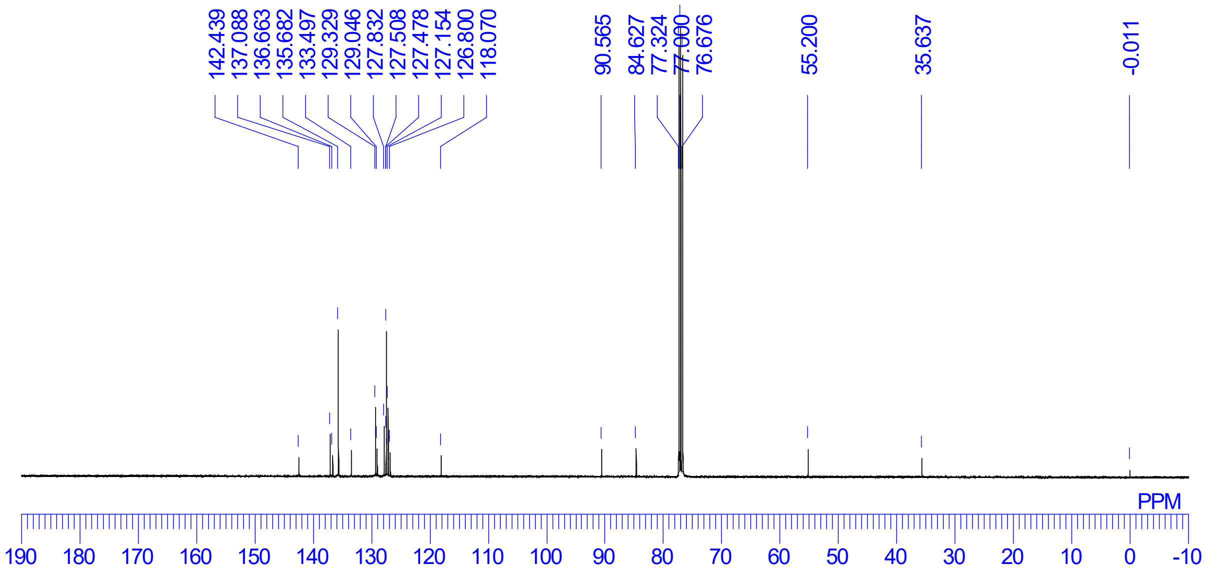

(1*S,2*R**)-1-Isopropoxy-1,2-diphenylpent-4-en-2-ol *syn*-3b**

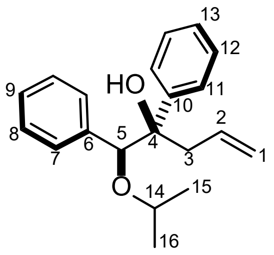

To a mixture of $SnCl_2$ (114 mg, 0.60 mmol) and benzoin isopropyl ether **2b** (127 mg, 0.50 mmol) in acetonitrile (5 mL) was added tributylallylstannane (199 mg, 0.60 mmol). After the reaction mixture was stirred for 21 h at room

temperature, methanol (5 mL) was added to the mixture. The residue was evaporated to give a crude mixture, which was analyzed by ^{1}H NMR to obtain the yield and diastereoselectivity using 1,1,2,2-tetrachloroethane as an internal standard (NMR yield: 91%, *syn*/*anti* = >99/1). The obtained residue was purified by column chromatography (10% w/w anhydrous K_2CO_3-silica, hexane/ethyl acetate = 70/30) to give the product as a colorless oil (117 mg, 79%). IR (neat) ν = 3571 (m), 3085 (w), 2973 (s), 2928 (m), 1641 (w), 1495 (m), 1449 (s), 1373 (s), 1304 (w), 1219 (m), 1120 (s), 1057 (s), 924 (m), 885 (m), 708 (s) cm^{-1}; ^{1}H NMR (400 MHz, $CDCl_3$) 7.20–7.11 (m, 8H), 6.98 (dd, J = 7.6, 1.2 Hz, 2H), 5.69–5.59 (m, 1H), 5.08 (dd, J = 17.0, 1.8 Hz, 1H), 5.00 (dd, J = 10.4, 2.4 Hz, 1H), 4.46 (s, 1H), 3.45 (sep, J = 6.3 Hz, 1H), 2.92–2.81 (m, 3H), 1.12 (d, J = 6.0 Hz, 3H), 1.08 (d, J = 6.4 Hz, 3H); ^{13}C{^{1}H} NMR (100 MHz, $CDCl_3$) 142.2 (s), 138.2 (s), 134.0 (d), 128.5 (d), 127.31 (d), 127.28 (d), 127.2 (d), 126.5 (d), 126.4 (d), 118.2 (t), 84.9 (d), 78.1 (s), 69.8 (d), 42.5 (t), 23.3 (q), 20.9 (q); HRMS (DART$^+$) Calculated ($C_{20}H_{24}O_2$): 296.1771 (M$^+$), Found: 296.1778.

^{1}H NMR: (400 MHz, $CDCl_3$)

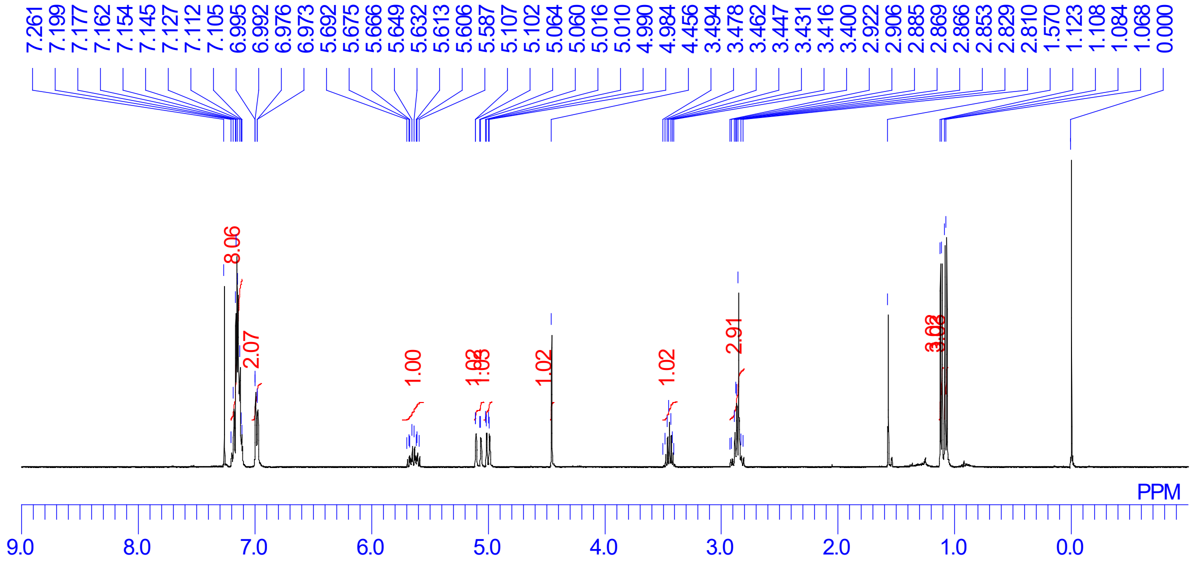

$^{13}C\{^1H\}$ NMR: (100 MHz, $CDCl_3$)

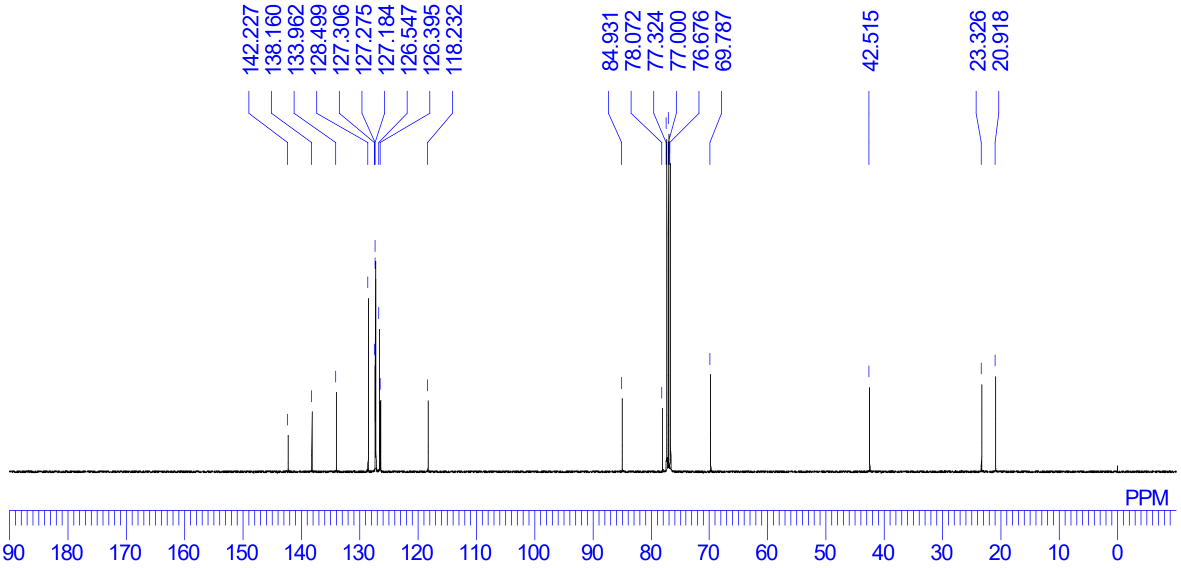

(1*S,2*S**)-1-Isopropoxy-1,2-diphenylpent-4-en-2-ol *anti*-3b**

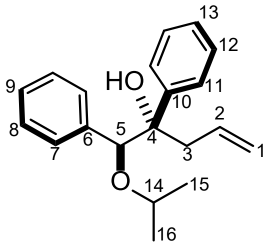

In a nitrogen-filled glovebox, to a mixture of BF_3·Et_2O (42.6 mg, 0.3 mmol) and benzoin isopropyl ether **2b** (76.3 mg, 0.3 mmol) in dichloromethane (3 mL) was added **1**Si(allyl) (106 mg, 0.3 mmol) at room temperature. After the reaction mixture was stirred for 12 h, methanol (3 mL) was added to the mixture. The residue was evaporated to give a crude mixture, which was analyzed by ^{1}H NMR to obtain the yield and diastereoselectivity using 1,1,2,2-tetrachloroethane as an internal standard (NMR yield: 71%, *syn*/*anti* = 1/>99). The obtained residue was purified by column chromatography (10% w/w anhydrous K_2CO_3-silica, hexane/ethyl acetate = 80/20). Further purification was conducted by a recycle GPC to give the product as a colorless oil (50.3 mg, 57%).

IR (neat) v = 3552 (m), 3062 (m), 2973 (s), 2930 (m), 2889 (m), 1639 (w), 1495 (m), 1448 (m), 1374 (m), 1177 (w), 1123 (m), 1059 (m), 917 (m), 702 (m) cm^{-1}; ^{1}H NMR (400 MHz, $CDCl_3$) 7.26–7.19 (m, 8H), 7.01 (dd, J = 7.8, 1.4 Hz, 2H, 7-H), 5.65–5.54 (m, 1H, 2-H), 5.02–4.94 (m, 2H, 1-H), 4.43 (s, 1H, 5-H), 3.43 (sep, J = 6.3 Hz, 1H, 14-H), 3.05 (s, 1H, OH), 2.67 (dd, J = 14.8, 6.8 Hz, 1H, 3-H), 2.52 (dd, J = 14.8, 7.6 Hz, 1H, 3-H), 1.05 (d, J = 6.4 Hz, 3H), 1.03 (d, J = 5.6 Hz, 3H); $^{13}C\{^1H\}$ NMR (100 MHz, $CDCl_3$) 142.7 (s, C-10), 138.1 (s, C-6), 133.9 (d, C-2), 128.6 (d), 127.6 (d), 127.4 (d, Two signals were overlapped.), 126.8 (d), 126.7 (d), 118.1 (t, C-1), 86.1 (d, C-5), 78.0 (s, C-4), 69.9 (d, C-14), 40.7 (t, C-3), 23.3 (q), 21.0 (q); HRMS (ESI$^+$) Calculated ($C_{20}H_{28}NO_2$): 314.2115 ($[M+NH_4]^+$), Found: 314.2114.

^{1}H NMR: (400 MHz, $CDCl_3$)

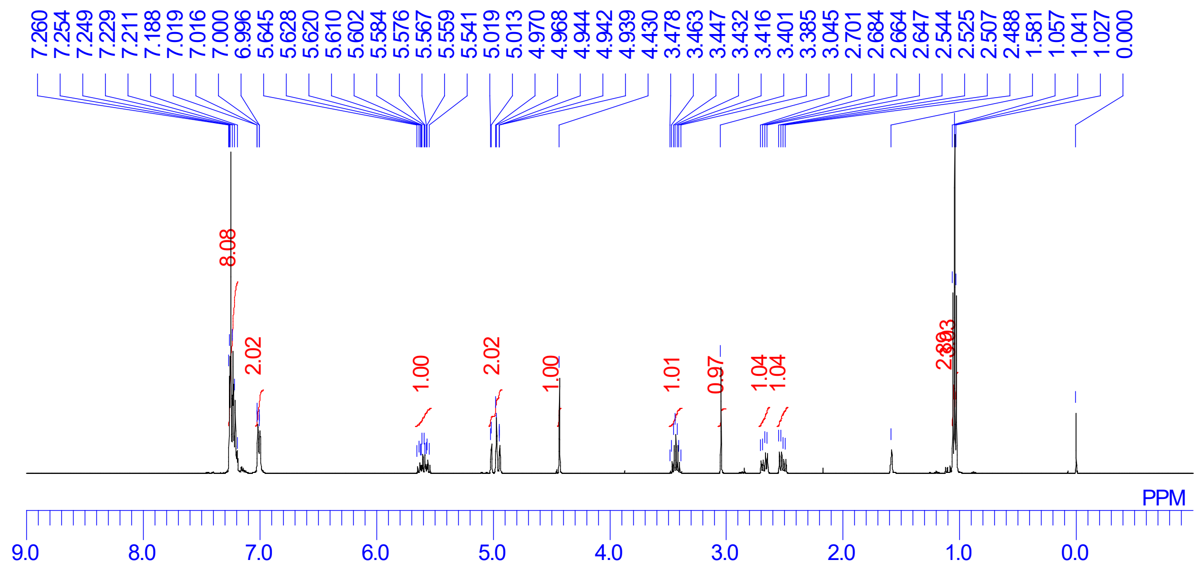

$^{13}C\{^1H\}$ NMR: (100 MHz, $CDCl_3$)

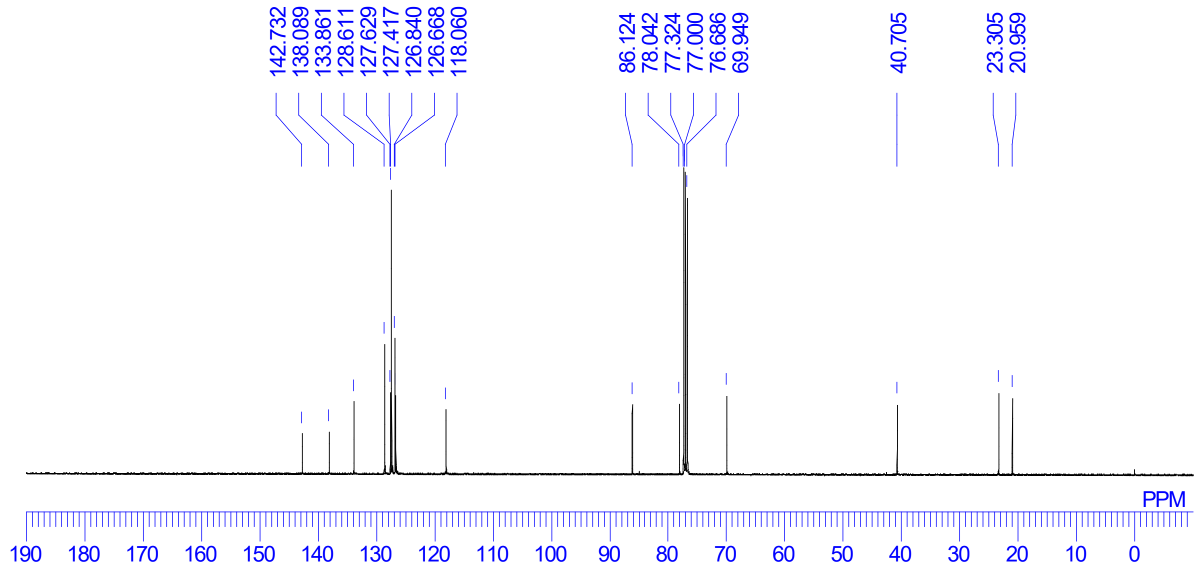

(1*S,2*R**)-1-Phenoxy-1,2-diphenylpent-4-en-2-ol *syn*-3c**

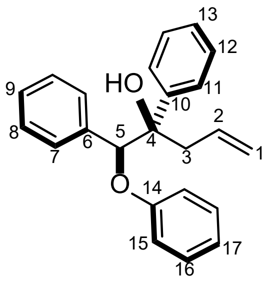

To a mixture of $SnCl_2$ (114 mg, 0.60 mmol) and 2-phenoxy-1,2-diphenylethan-1-one **2c** (144 mg, 0.50 mmol) in acetonitrile (5 mL) was added tributylallylstannane (199 mg, 0.60 mmol). After the reaction mixture was stirred for

13 h at room temperature, methanol (5 mL) was added to the mixture. The residue was evaporated to give a crude mixture, which was analyzed by ^{1}H NMR to obtain the yield and diastereoselectivity using 1,1,2,2-tetrachloroethane as an internal standard (NMR yield: 93%, *syn*/*anti* = >99/1). The obtained residue was purified by column chromatography (10% w/w anhydrous K_2CO_3-silica, hexane/ethyl acetate = 80/20) to give the product as a colorless oil (142 mg, 86%).

IR (neat) v = 3566 (br), 3062 (m), 3030 (m), 2910 (w), 1713 (m), 1638 (w), 1598 (s), 1493 (s), 1453 (s), 1362 (m), 1289 (m), 1236 (s), 1173 (m), 1077 (m), 998 (m), 973 (w), 888 (w), 860 (m), 753 (s) cm^{-1}; ^{1}H NMR (400 MHz, $CDCl_3$) 7.29–7.19 (m, 5H), 7.15–7.10 (m, 5H), 7.03 (dd, J = 7.6, 1.6 Hz, 2H), 6.84 (t, J = 7.4 Hz, 1H), 6.80 (d, J = 8.0 Hz, 2H), 5.68–5.58 (m, 1H), 5.20 (s, 1H), 5.12 (dd, J = 17.2, 2.0 Hz, 1H), 5.03 (dd, J = 10.0, 2.0 Hz, 1H), 3.04 (dd, J = 14.4, 6.0 Hz, 1H) , 2.94 (dd, J = 14.4, 8.0 Hz, 1H), 2.62 (s, 1H); ^{13}C{^{1}H} NMR (100 MHz, acetone-d_6) 158.9 (s), 143.3 (s), 138.1 (s), 135.1 (d), 129.9 (d), 129.3 (d), 128.0 (d), 127.9 (d), 127.8 (d), 127.5 (d), 127.1 (d), 121.4 (d), 117.8 (t), 116.6 (d), 86.3 (d), 79.0 (s), 43.4 (t); HRMS (MALDI-TOF MS) Calculated ($C_{23}H_{22}O_2Na$): 353.1512 ([M+Na]$^{+}$), Found: 353.1509.

^{1}H NMR: (400 MHz, $CDCl_3$)

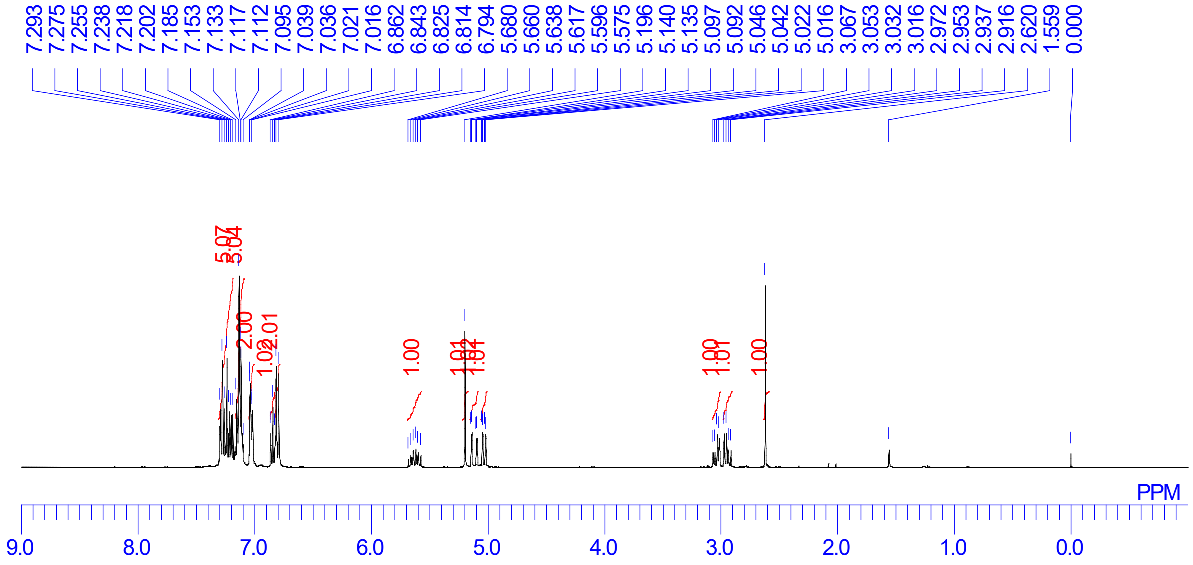

$^{13}C\{^{1}H\}$ NMR: (100 MHz, acetone-d_6)

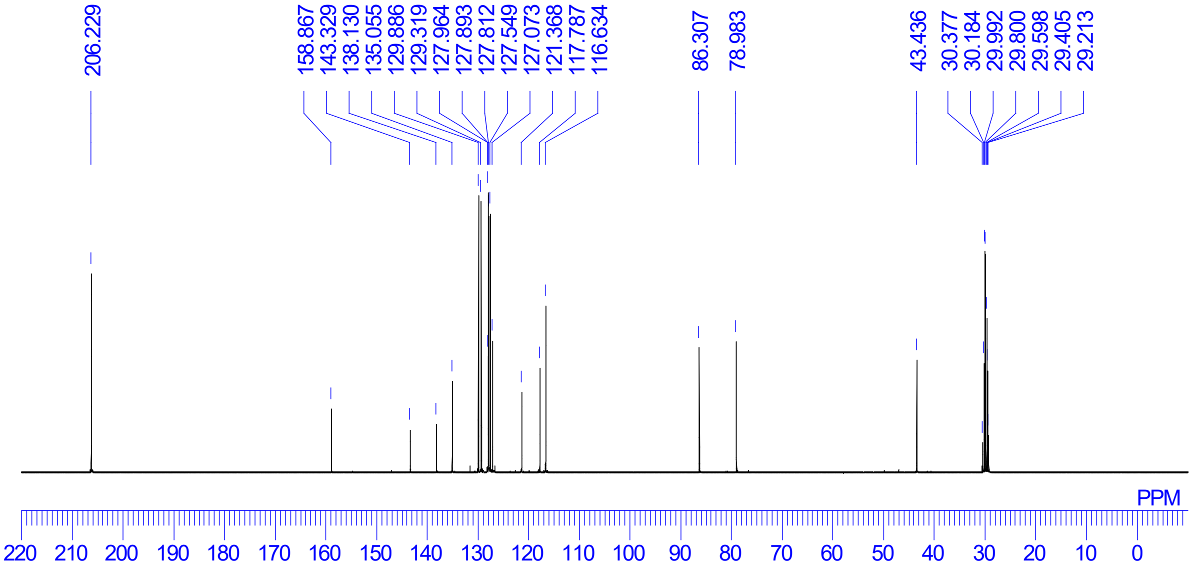

(1*S,2*S**)-1-Phenoxy-1,2-diphenylpent-4-en-2-ol *anti*-3c**

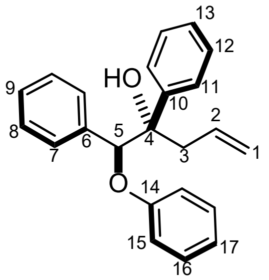

In a nitrogen-filled glovebox, to a mixture of $BF_3{\cdot}Et_2O$ (28.3 mg, 0.2 mmol) and 2-phenoxy-1,2-diphenylethan-1-one **2c** (57.7 mg, 0.2 mmol) in dichloromethane (2 mL) was added **1**Si(allyl) (70.7 mg, 0.2 mmol). After the reaction mixture was stirred for 6 h at room temperature, methanol (2 mL) was added to the mixture. The residue was evaporated to give a crude mixture, which was analyzed by ^{1}H NMR to obtain the yield and diastereoselectivity using 1,1,2,2-tetrachloroethane as an internal standard (NMR yield: 92%, *syn*/*anti* = 1/>99). The obtained residue was purified by column chromatography (hexane/ethyl acetate = 80/20) on silica gel. Further purification was conducted by a recycle GPC to give product as a colorless oil (64.0 mg, 91%).

IR (neat) v = 3564 (br), 3062 (m), 3031 (m), 2917 (w), 1639 (m), 1598 (s), 1494 (s), 1449 (s), 1347 (m), 1301 (m), 1235 (s), 1173 (m), 1156 (w), 1078 (m), 1040 (m), 919 (m), 752 (s) cm^{-1}; ^{1}H NMR (400 MHz, $CDCl_3$) 7.33–7.25 (m, 5H), 7.22–7.12 (m, 5H), 7.01 (d, J = 8.0 Hz, 2H), 6.86 (t, J = 7.4 Hz, 1H), 6.78 (d, J = 8.8 Hz, 2H), 5.72–5.61 (m, 1H, 2-H), 5.21 (s, 1H, 5-H), 5.13–5.06 (m, 2H, 1-H), 2.93 (dd, J = 14.0, 5.2 Hz, 1H, 3-H), 2.81–2.75 (m, 2H, 3-H, OH); $^{13}C\{^{1}H\}$ NMR (100 MHz, $CDCl_3$) 157.7 (s), 141.6 (s, C-10), 136.4 (s), 133.2 (d, C-2), 129.3 (d), 128.2 (d), 127.9 (d), 127.7 (d), 127.6 (d), 127.1 (d), 126.9 (d), 121.1 (d), 119.3 (t, C-1), 116.0 (d), 86.4 (d, C-5), 78.1 (s, C-4), 41.4 (t, C-3); HRMS (MALDI-TOF MS) Calculated ($C_{23}H_{22}O_2Na$): 353.1512 ([M+Na]$^+$), Found: 353.1499.

^{1}H NMR: (400 MHz, $CDCl_3$)

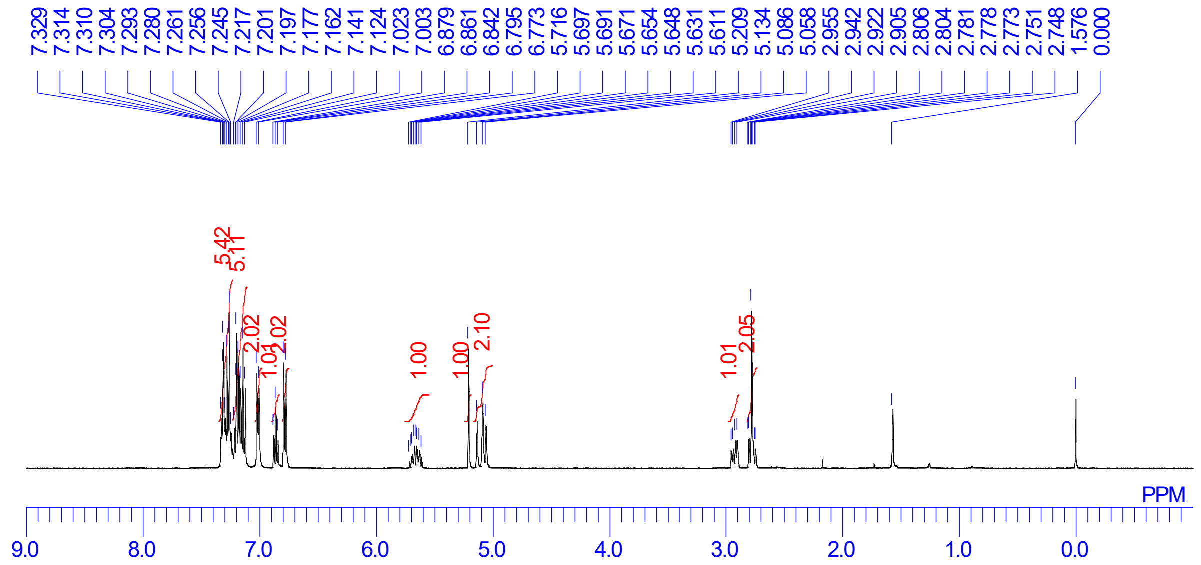

^{13}C{^{1}H} NMR: (100 MHz, $CDCl_3$)

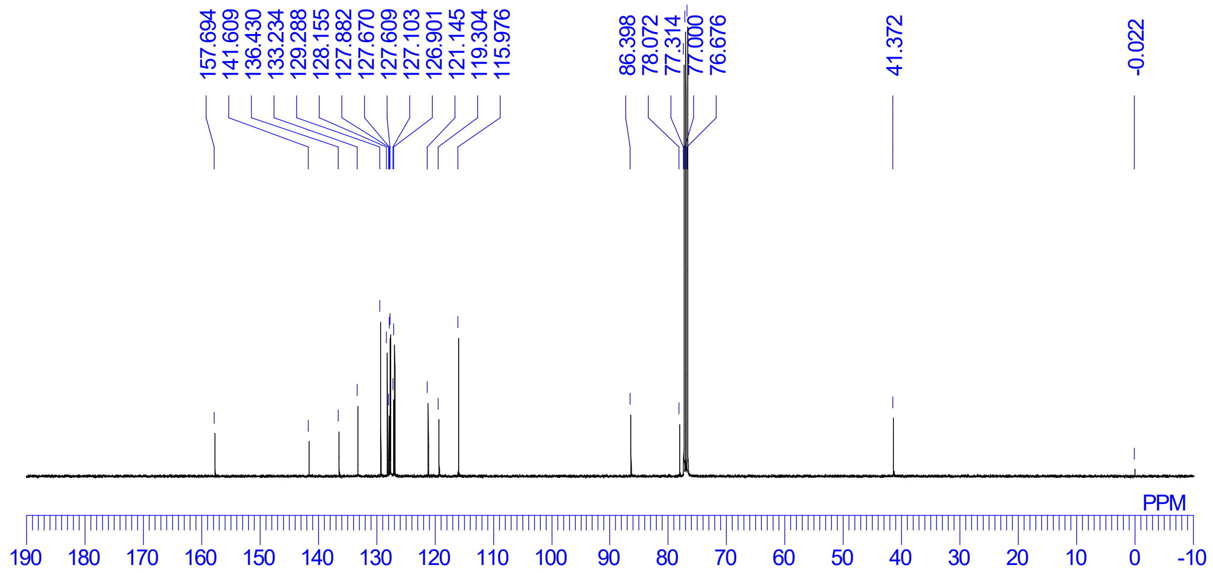

(1*S,2*R**)-2-Hydroxy-1,2-diphenylpent-4-en-1-yl acetate *syn*-3d**

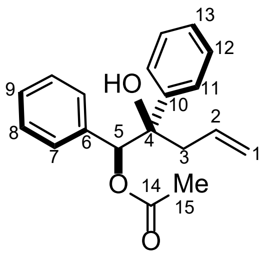

To a mixture of $SnCl_2$ (114 mg, 0.60 mmol) and 2-oxo-1,2-diphenylethyl acetate **2d** (127 mg, 0.50 mmol) in acetonitrile (5 mL) was added tributylallylstannane (199 mg, 0.60 mmol). After the reaction mixture was stirred for

13 h at room temperature, methanol (5 mL) was added to the mixture. The residue was evaporated to give a crude mixture, which was analyzed by ^{1}H NMR to obtain the yield and diastereoselectivity using 1,1,2,2-tetrachloroethane as an internal standard (NMR yield: 97%, *syn*/*anti* = >99/1). The obtained residue was purified by column chromatography (10% w/w anhydrous K_2CO_3-silica, hexane/ethyl acetate = 20/80) to give the product as a colorless solid (136 mg, 92%).

mp 106.8–107.6 °C; IR (KBr) ν = 3535 (br), 3073 (w), 2935 (w), 1731 (s), 1641 (w), 1448 (w), 1372 (m), 1245 (s), 1175 (s), 1027 (s), 967 (m), 926 (m), 886 (m), 759 (m), 742 (m) cm^{-1}; ^{1}H NMR (400 MHz, $CDCl_3$) 7.26–7.25 (m, 3H), 7.23–7.21 (m, 2H), 7.19–7.13 (m, 3H), 7.01 (dd, *J* = 8.2, 1.0 Hz, 2H), 5.99 (s, 1H), 5.58–5.48 (m, 1H), 5.14 (dd, *J* = 17.2, 1.2 Hz, 1H), 5.07 (dd, *J* = 10.0, 2.4 Hz, 1H), 2.94 (dd, *J* = 14.4, 6.0 Hz, 1H), 2.68 (dd, *J* = 14.4, 8.8 Hz, 1H), 2.28 (s, 1H), 2.16 (s, 3H); ^{13}C{^{1}H} NMR (100 MHz, $CDCl_3$) 170.0 (s), 141.4 (s), 135.9 (s), 132.7 (d), 128.1 (d), 127.84 (d), 127.79 (d), 127.5 (d), 127.1 (d), 126.3 (d), 119.8 (t), 80.6 (d), 77.5 (s), 42.5 (t), 21.2 (q); HRMS (MALDI-TOF MS) Calculated ($C_{19}H_{20}O_3Na$): 319.1305 ([M+Na]$^+$), Found: 319.1297.

^{1}H NMR: (400 MHz, $CDCl_3$)

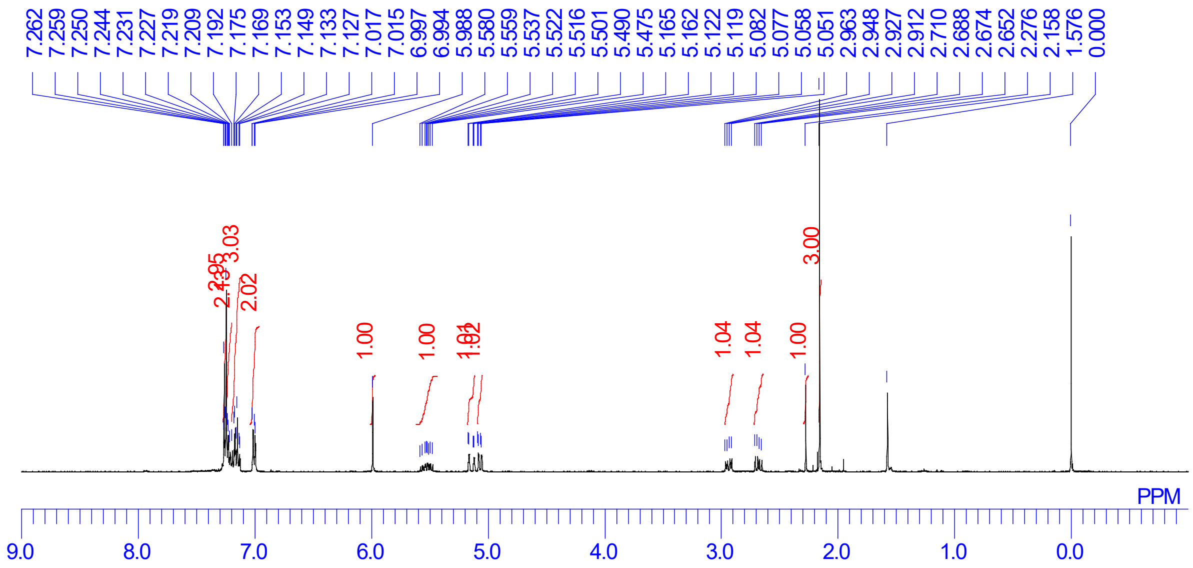

$^{13}C\{^1H\}$ NMR: (100 MHz, $CDCl_3$)

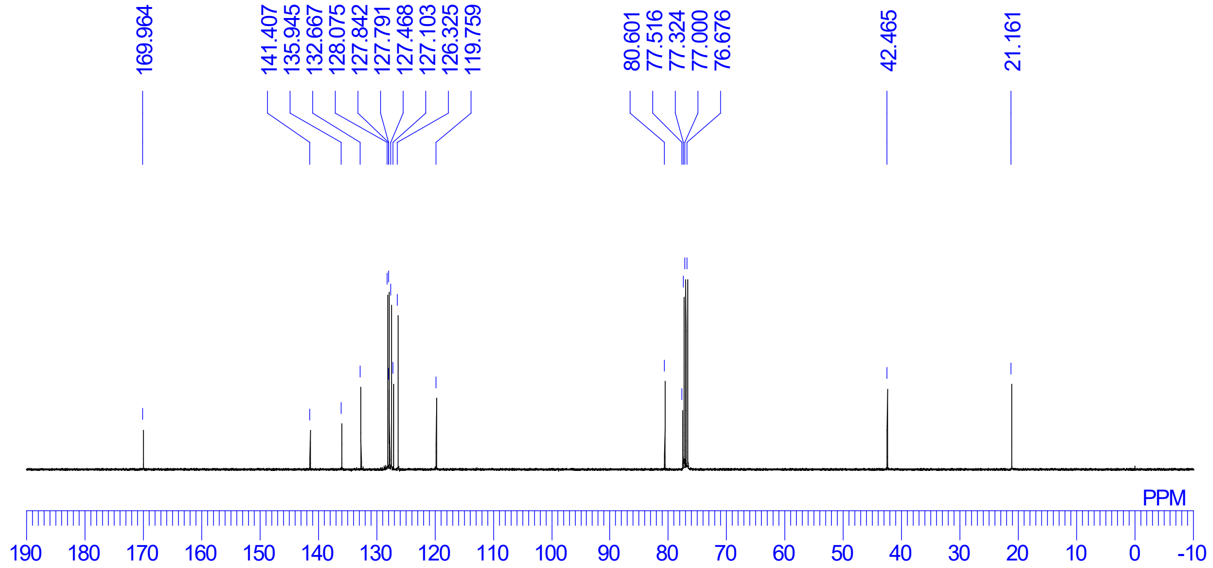

(1*S,2*S**)-2-Hydroxy-1,2-diphenylpent-4-en-1-yl acetate *anti*-3d**

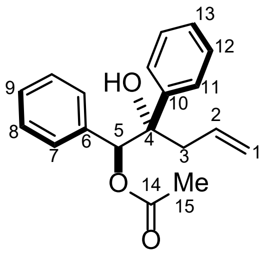

In a nitrogen-filled glovebox, to a mixture of $BF_3 \cdot Et_2O$ (14.2 mg, 0.1 mmol) and 2-oxo-1,2-diphenylethyl acetate **2d** (25.4 mg, 0.1 mmol) in dichloromethane (2 mL) was added **1**Si(allyl) (35.4 mg, 0.1 mmol) at 0 °C. After the reaction mixture was stirred for 12 h, methanol (1 mL) was added to the mixture. The residue was evaporated to give a crude mixture, which was analyzed by ^1H NMR to obtain the yield and diastereoselectivity using 1,1,2,2-tetrachloroethane as an internal standard (NMR yield: 92%, *syn*/*anti* = 1/>99). The obtained residue was purified by a recycle GPC to give product as a colorless solid (23.7 mg, 80%).

mp 112.1–112.8 °C; IR (KBr) ν = 3520 (br), 3061 (m), 2979 (m), 2913 (w), 1730 (s), 1642 (m), 1602 (w), 1496 (m), 1436 (s), 1375 (s), 1236 (s), 1135 (s), 1078 (m), 917 (s), 844 (m), 806 (w), 708 (s) cm^{-1}; ^1H NMR (400 MHz, $CDCl_3$) 7.37–7.33 (m, 3H), 7.31–7.29 (m, 3H), 7.28–7.24 (m, 2H), 7.22–7.19 (m, 2H), 5.99 (s, 1H, 5-H), 5.53–5.42 (m, 1H, 2-H), 5.06–5.02 (m, 2H, 1-H), 2.80 (dd, J = 13.8, 6.2 Hz, 1H, 3-H), 2.42 (dd, J = 13.8, 8.2 Hz, 1H, 3-H), 2.34 (s, 1H, OH), 1.95 (s, 3H, 15-H); $^{13}C\{^1H\}$ NMR (100 MHz, $CDCl_3$) 169.5 (s, C-14), 142.0 (s, C-10), 136.2 (s, C-6), 132.3 (d, C-2), 128.5 (d), 128.2 (d), 127.9 (d), 127.8 (d), 127.1 (d), 126.2 (d), 120.0 (t, C-1), 80.6 (d, C-5), 77.4 (s, C-4), 42.6 (t, C-3), 20.9 (q, C-15); HRMS (MALDI-TOF MS) Calculated ($C_{19}H_{20}O_3Na$): 319.1305 ($[M+Na]^+$), Found: 319.1319.

^{1}H NMR: (400 MHz, $CDCl_3$)

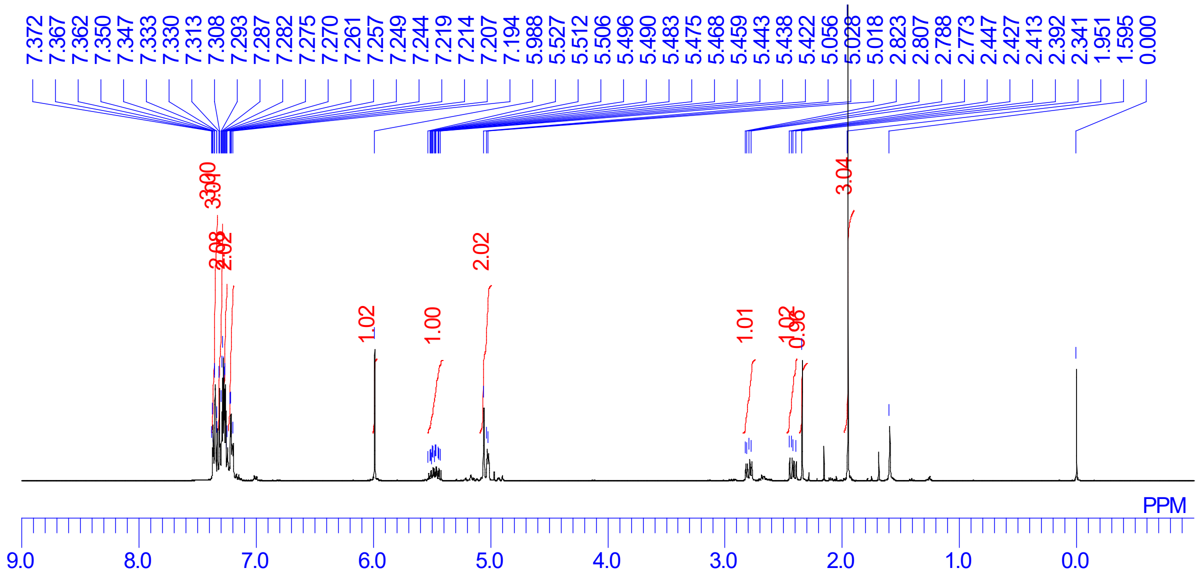

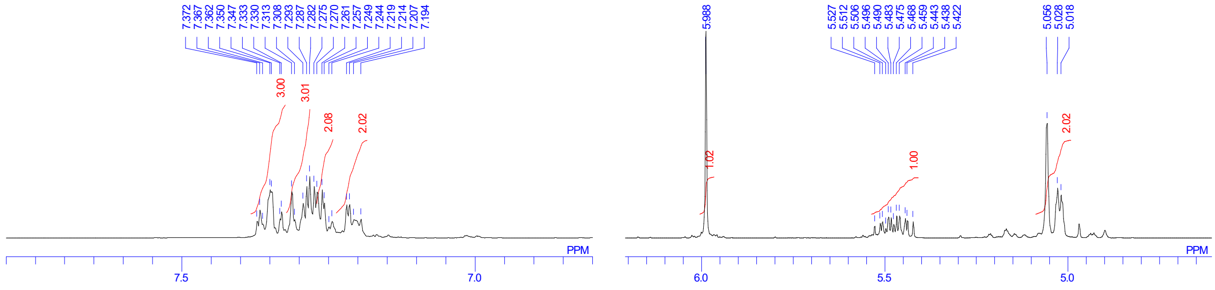

$^{13}C\{^{1}H\}$ NMR: (100 MHz, $CDCl_3$)

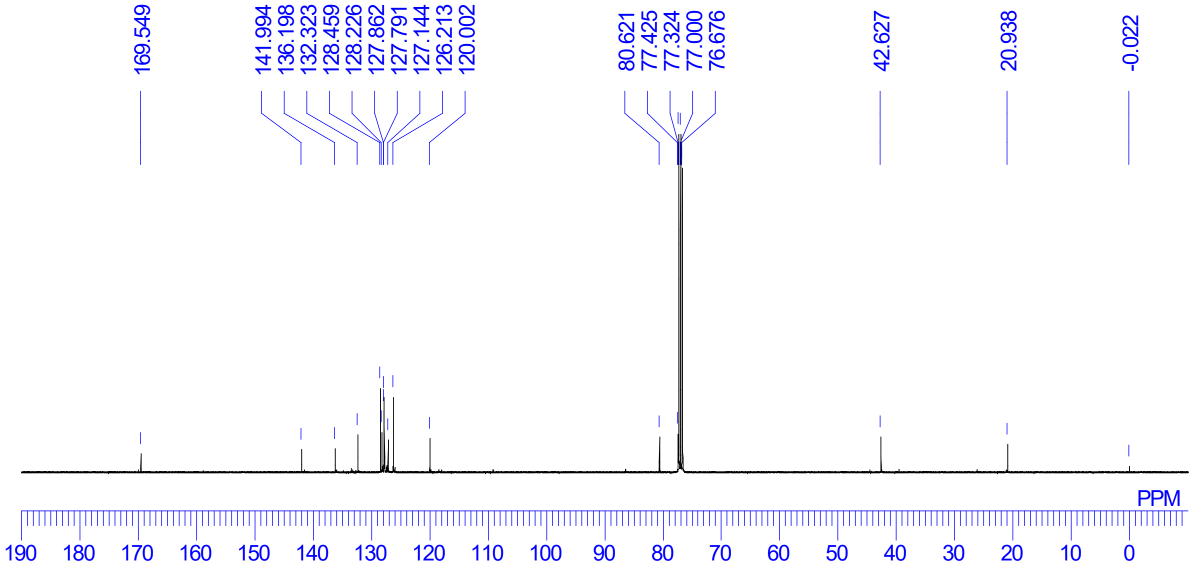

(2S*,3S*)-2-{(*tert*-butyldiphenylsilyl)oxy}-3-phenylhex-5-en-3-ol *anti*-3e

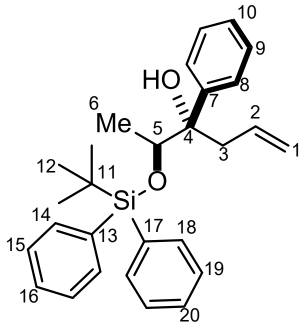

In a nitrogen-filled glovebox, to a mixture of BF_3·Et_2O (14.2 mg, 0.10 mmol) and 2-{(*tert*-butyldiphenylsilyl)oxy}-1-phenylpropan-1-one **2e** (38.9 mg, 0.10 mmol) in dichloromethane (1 mL) was added **1**Si(allyl) (53.0 mg, 0.15 mmol). After the reaction mixture was stirred for 6 h at room temperature, methanol (1 mL) was added to the mixture. The residue was evaporated to give a crude mixture, which was analyzed by ^{1}H NMR to obtain the yield and diastereoselectivity using 1,1,2,2-tetrachloroethane as an internal standard (NMR yield: 95%, *syn*/*anti* = 4/96). The obtained residue was purified by column chromatography (hexane/ethyl acetate = 80/20) on silica gel. Further purification was conducted by a recycle GPC to give product as a colorless oil (32.8 mg, 76%). The NMR data were consistent with the data previously reported.[5]

^{1}H NMR (400 MHz, $CDCl_3$) 7.59 (d, J = 6.4 Hz, 2H), 7.53 (d, J = 6.8 Hz, 2H), 7.43–7.39 (m, 4H), 7.36–7.30 (m, 6H), 7.25 (t, J = 7.4 Hz, 1H), 5.61–5.51 (m, 1H), 5.07–4.98 (m, 2H), 3.99 (q, J = 6.4 Hz, 1H), 2.81 (s, 1H), 2.73 (dd, J = 14.2, 6.6 Hz, 1H), 2.59 (dd, J = 14.0, 7.6 Hz, 1H), 0.94 (d, J = 6.4 Hz, 3H), 0.92 (s, 9H); ^{13}C{^{1}H} NMR (100 MHz, $CDCl_3$) 143.8 (s), 135.92 (d), 135.90 (d), 134.3 (s), 133.7 (d), 132.8 (s), 129.8 (d), 129.5 (d), 127.8 (d), 127.6 (d), 127.4 (d), 126.6 (d), 126.5 (d), 118.5 (t), 78.2 (s), 76.2 (d), 41.8 (t), 26.8 (q), 19.2 (s), 17.8 (q); ^{29}Si{^{1}H} NMR (78.7 MHz, $CDCl_3$, Me_4Si in $CDCl_3$ as an external standard) −4.77.

^{1}H NMR: (400 MHz, $CDCl_3$)

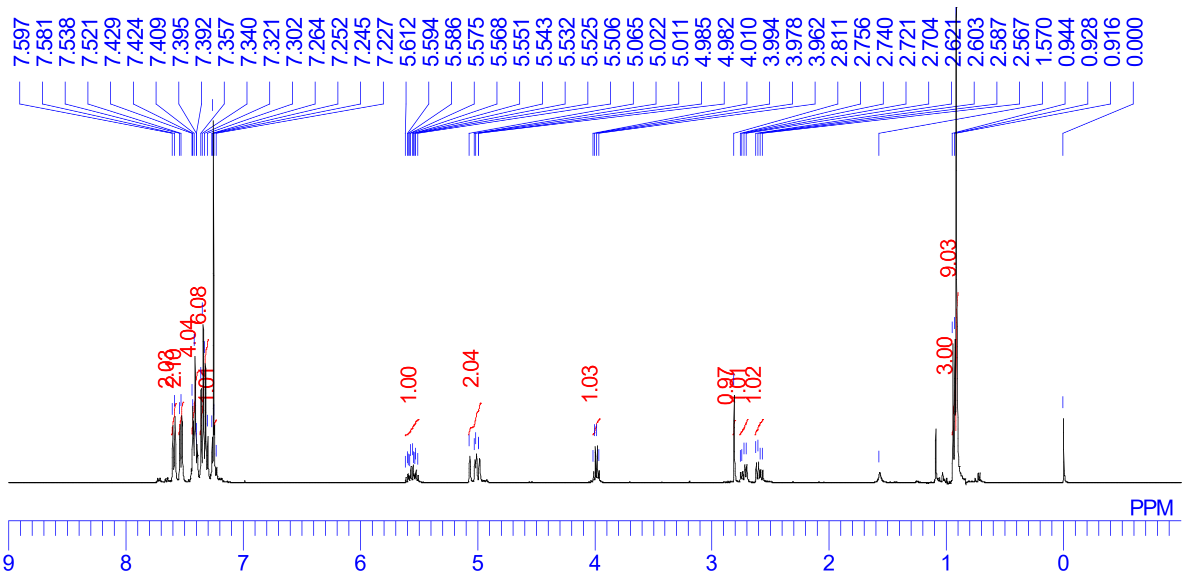

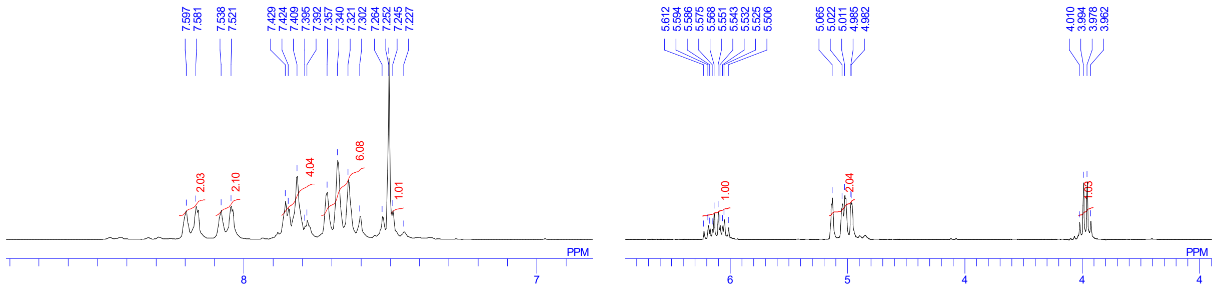

$^{13}C\{^1H\}$ NMR: (100 MHz, $CDCl_3$)

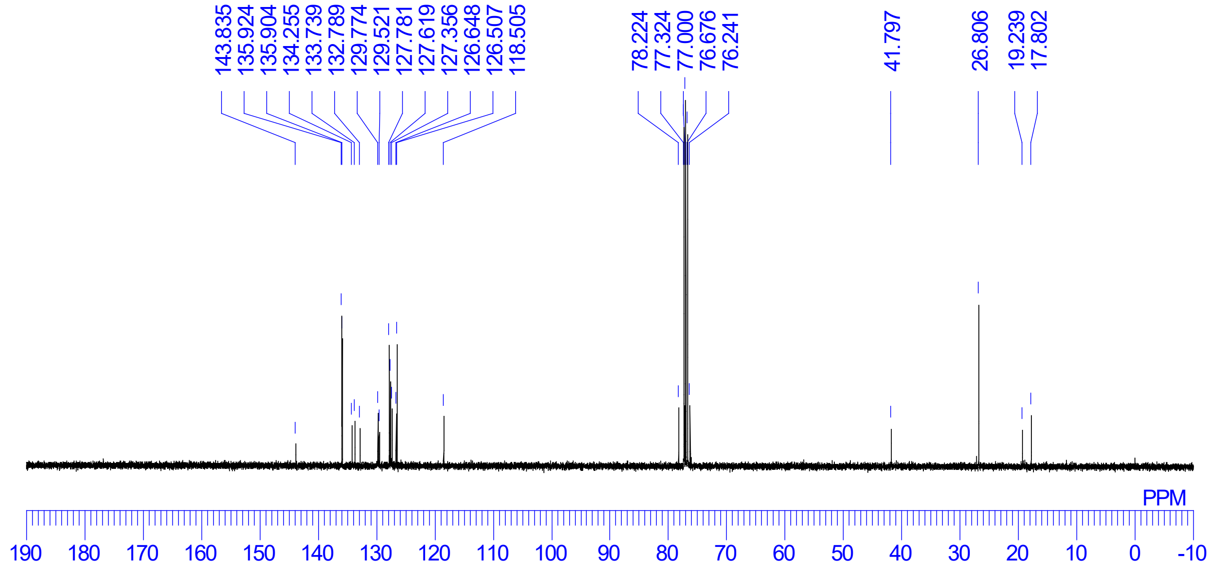

$^{29}Si\{^1H\}$ NMR: (78.7 MHz, $CDCl_3$, Me_4Si in $CDCl_3$ as an external standard)

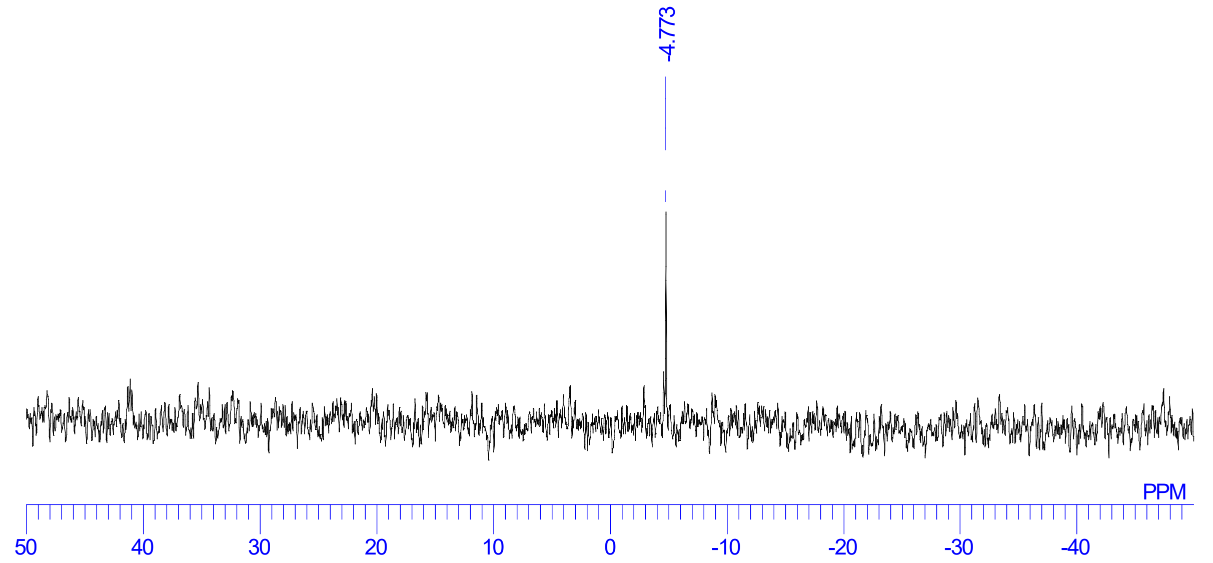

2-[{(2*S,3*R**)-3-Hydroxy-3-phenylhex-5-en-2-yl}oxy]isoindoline-1,3-dione *syn*-3f**

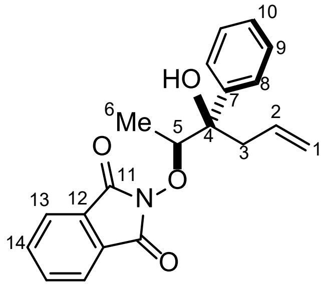

To a mixture of $SnCl_2$ (68.3 mg, 0.36 mmol) and 2-{(1-oxo-1-phenylpropan-2-yl)oxy}isoindoline-1,3-dione **2f** (88.6 mg, 0.30 mmol) in acetonitrile (3 mL) was added tributylallylstannane (119.2 mg, 0.36 mmol). After the reaction mixture was stirred for 12 h at room temperature, methanol (3 mL) was added to the mixture. The residue was evaporated to give a crude mixture, which was analyzed by ^{1}H NMR to obtain the yield and diastereoselectivity using 1,1,2,2-tetrachloroethane as an internal standard (NMR yield: 89%, *syn*/*anti* = >99/1). The obtained residue was purified by column chromatography (10% w/w anhydrous K_2CO_3-silica, hexane/ethyl acetate = 20/80) to give the product as a colorless oil (78.8 mg, 78%).

IR (neat) v = 3555 (br), 3091 (w), 3063 (w), 2986 (m), 2939 (m), 1788 (m), 1731 (s), 1642 (m), 1496 (m), 1447 (m), 1374 (s), 1323 (m), 1223 (m), 1187 (s), 1079 (m), 1031 (m), 981 (s), 849 (m), 768 (s) cm^{-1}; ^{1}H NMR (400 MHz, $CDCl_3$) 7.87–7.85 (m, 2H), 7.78–7.76 (m, 2H), 7.47 (d, J = 8.0 Hz, 2H), 7.36 (t, J = 7.6 Hz, 2H), 7.26 (t, J = 7.2 Hz, 1H), 5.73–5.62 (m, 1H), 5.13 (d, J = 18.0 Hz, 1H), 5.05 (d, J = 9.6 Hz, 1H), 4.57 (q, J = 6.5 Hz, 1H), 3.70 (s, 1H), 3.06 (dd, J = 14.6, 8.2 Hz, 1H), 2.92 (dd, J = 14.8, 6.0 Hz, 1H), 1.18 (d, J = 6.4 Hz, 3H); ^{13}C{^{1}H} NMR (100 MHz, $CDCl_3$) 164.6 (s), 141.8 (s), 134.7 (d), 133.3 (d), 128.7 (s), 128.1 (d), 127.0 (d), 125.9 (d), 123.7 (d), 118.8 (t), 90.6 (d), 77.4 (s), 43.5 (t), 13.6 (q); HRMS (MALDI-TOF MS) Calculated ($C_{20}H_{19}NO_4Na$): 360.1206 ([M+Na]$^{+}$), Found: 360.1202.

^{1}H NMR: (400 MHz, $CDCl_3$)

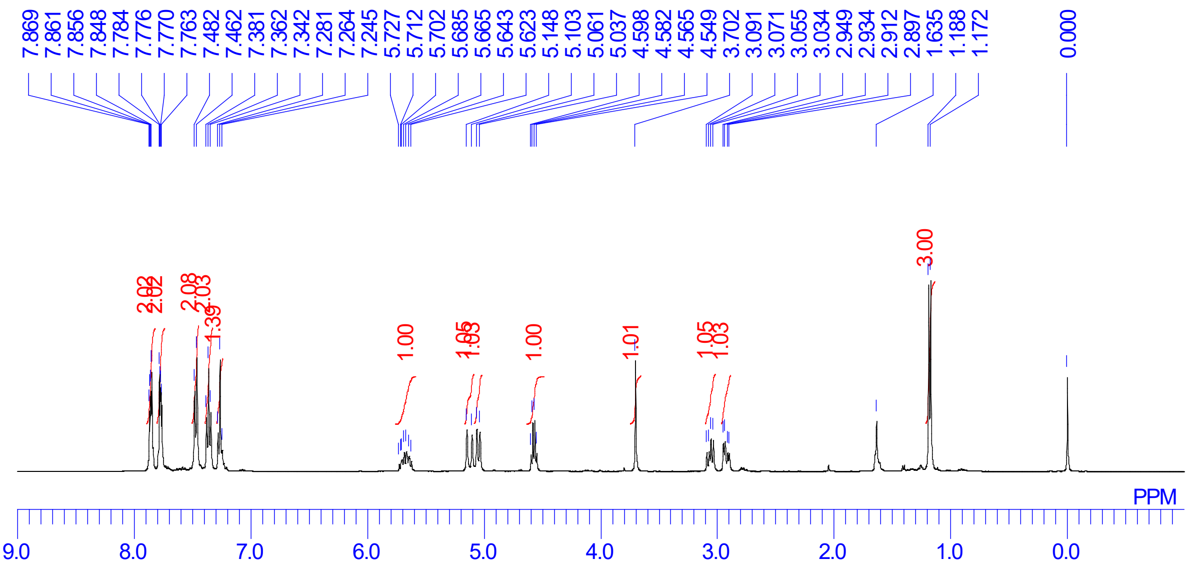

$^{13}C\{^1H\}$ NMR: (100 MHz, $CDCl_3$)

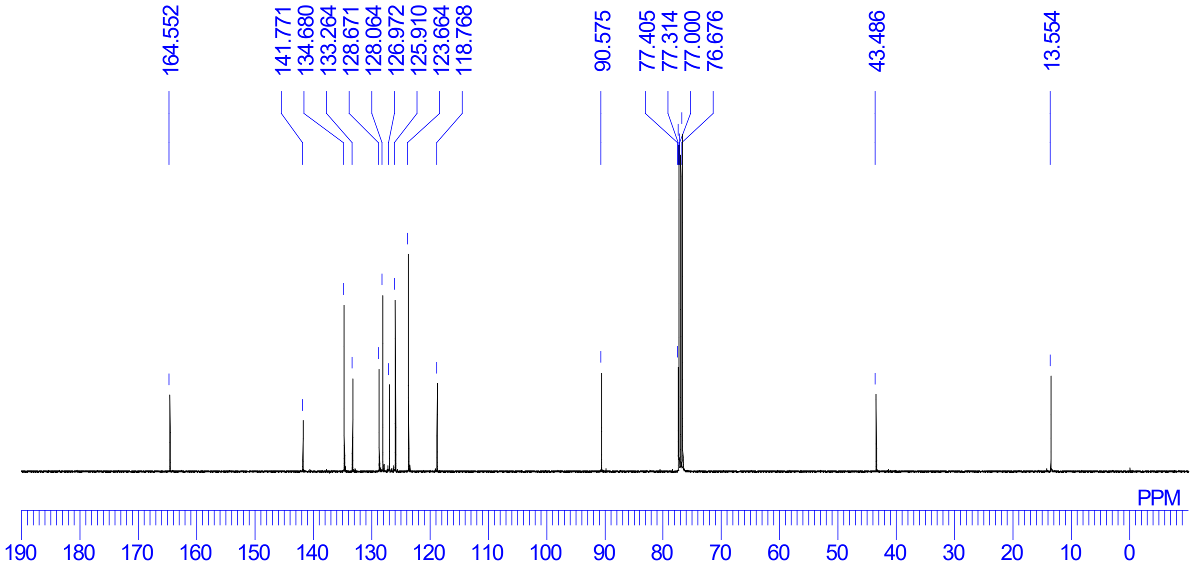

2-[{(2*S,3*S**)-3-hydroxy-3-phenylhex-5-en-2-yl}oxy]isoindoline-1,3-dione *anti*-3f**

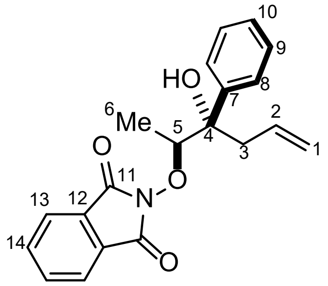

In a nitrogen-filled glovebox, to a mixture of $BF_3 \cdot Et_2O$ (28.4 mg, 0.2 mmol) and 2-{(1-oxo-1-phenylpropan-2-yl)oxy}isoindoline-1,3-dione **2f** (59.1 mg, 0.2 mmol) in dichloromethane (2 mL) was added **1**Si(allyl) (70.8 mg, 0.2 mmol). After the reaction mixture was stirred for 3 h at room temperature, methanol (2 mL) was added to the mixture. The residue was evaporated to give a crude mixture, which was analyzed by ^1H NMR to obtain the yield and diastereoselectivity using 1,1,2,2-tetrachloroethane as an internal standard (NMR yield: 98%, *syn*/*anti* = 1/>99). The obtained residue was purified by column chromatography (hexane/ethyl acetate = 25/75) on silica gel. Further purification was conducted by a recycle GPC to give product as a colorless oil (60.6 mg, 90%).

IR (neat) v = 3490 (br), 3068 (m), 3027 (w), 2984 (m), 2942 (w), 1793 (s), 1736 (s), 1719 (s), 1686 (m), 1492 (m), 1467 (m), 1376 (m), 1188 (m), 1080 (m), 983 (s), 879 (m), 764 (s) cm^{-1}; ^1H NMR (400 MHz, $CDCl_3$) 7.75–7.69 (m, 4H, 13-H, 14-H), 7.49 (dd, J = 8.4, 1.2 Hz, 2H, 8-H), 7.23 (t, J = 7.8 Hz, 2H, 9-H), 7.08 (t, J = 7.4 Hz, 1H, 10-H), 5.71–5.61 (m, 1H, 2-H), 5.11 (dd, J = 17.4, 1.8 Hz, 1H, 1-H), 5.06 (dd, J = 10.4, 2.0 Hz, 1H, 1-H), 4.69 (q, J = 6.5 Hz, 1H, 5-H), 2.79 (dd, J = 7.4, 1.0 Hz, 1H, 3-H), 1.41 (d, J = 6.8 Hz, 3H, 6-H); $^{13}C\{^1H\}$ NMR (100 MHz, $CDCl_3$) 164.1 (s, C-11), 142.2 (s, C-7), 134.4 (d), 132.8 (d, C-2), 128.5 (s, C-12), 127.9 (d, C-9), 126.8 (d, C-10), 126.2 (d, C-8), 123.4 (d), 119.1 (t, C-1), 89.8 (d, C-5), 76.9 (s, C-4), 41.5 (t, C-3), 14.3 (q, C-6); HRMS (MALDI-TOF MS) Calculated ($C_{20}H_{19}NO_4Na$): 360.1206 ($[M+Na]^+$), Found: 360.1210.

^{1}H NMR: (400 MHz, $CDCl_3$)

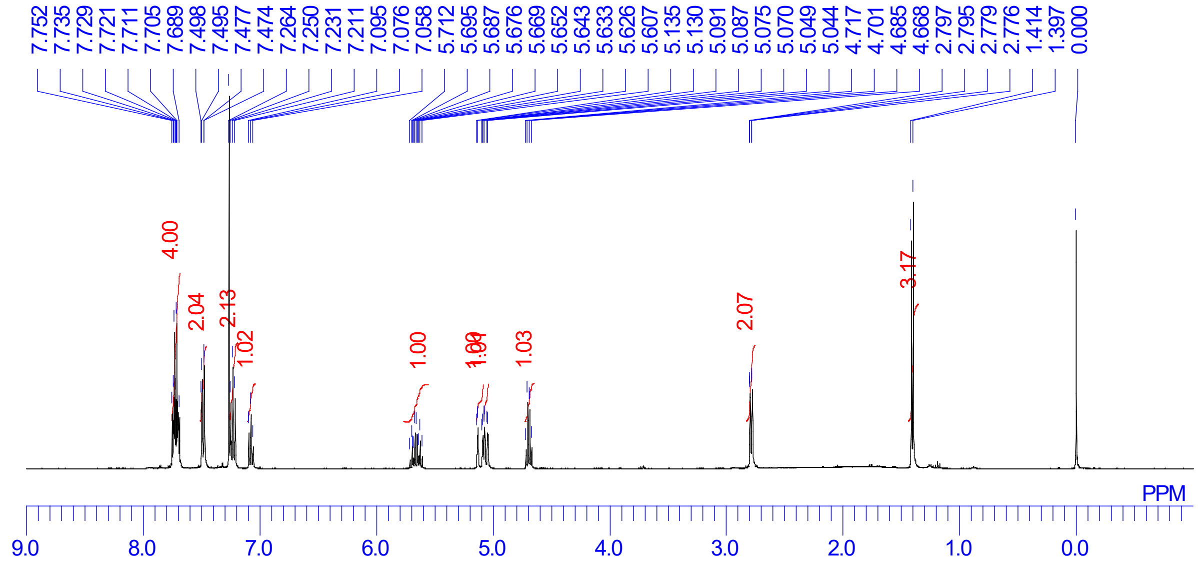

^{13}C{^{1}H} NMR: (100 MHz, $CDCl_3$)

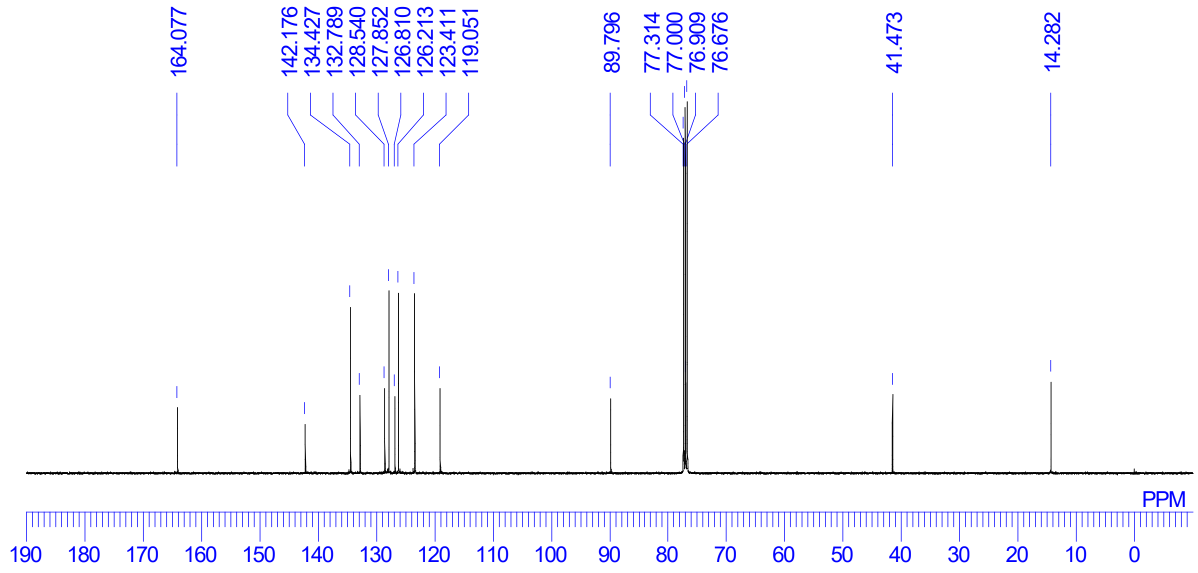

1,2-Bis(4-chlorophenyl)-2-methoxyethan-1-one 2g

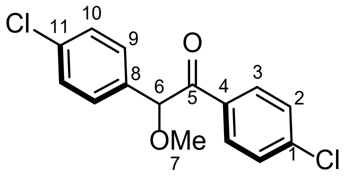

Methyl iodide (1.46 mL, 23.5 mmol) was added to a suspension of 1,2-bis(4-chlorophenyl)-2-hydroxyethan-1-one (1.10 g, 3.92 mmol) and silver(I) oxide (1.81 g, 7.83 mmol) in chloroform (10 mL). The reaction mixture was heated to reflux for 20 h. After the reaction mixture was cooled to room temperature, the resulting suspension was filtered on celite. The filtrate was dried over Na_2SO_4 and the solvent was removed in vacuum. The obtained residue was

purified by column chromatography (hexane/ethyl acetate = 40/60) on silicagel to give the product as a colorless oil (0.80 g, 69%).

IR (neat) ν = 3091 (w), 3069 (w), 2934 (m), 2827 (m), 1723 (s), 1695 (s), 1589 (s), 1489 (s), 1462 (m), 1401 (s), 1282 (s), 1092 (s), 1014 (s), 945 (m), 817 (s) cm^{-1}; ^{1}H NMR (400 MHz, $CDCl_3$) 7.93 (d, J = 8.8 Hz, 2H), 7.379 (d, J = 8.8 Hz, 2H), 7.376 (d, J = 8.8 Hz, 2H), 7.33 (d, J = 8.8 Hz, 2H), 5.38 (s, 1H), 3.45 (s, 3H); $^{13}C\{^{1}H\}$ NMR (100 MHz, $CDCl_3$) 195.8 (s), 139.9 (s), 134.6 (s), 134.3 (s), 132.7 (s), 130.5 (d), 129.1 (d), 128.9 (d), 128.5 (d), 86.3 (d), 57.6 (q); HRMS (DART$^+$) Calculated ($C_{15}H_{16}NO_2Cl_2$): 312.0553 ($[M+NH_4]^+$), Found: 312.0549.

^{1}H NMR: (400 MHz, $CDCl_3$)

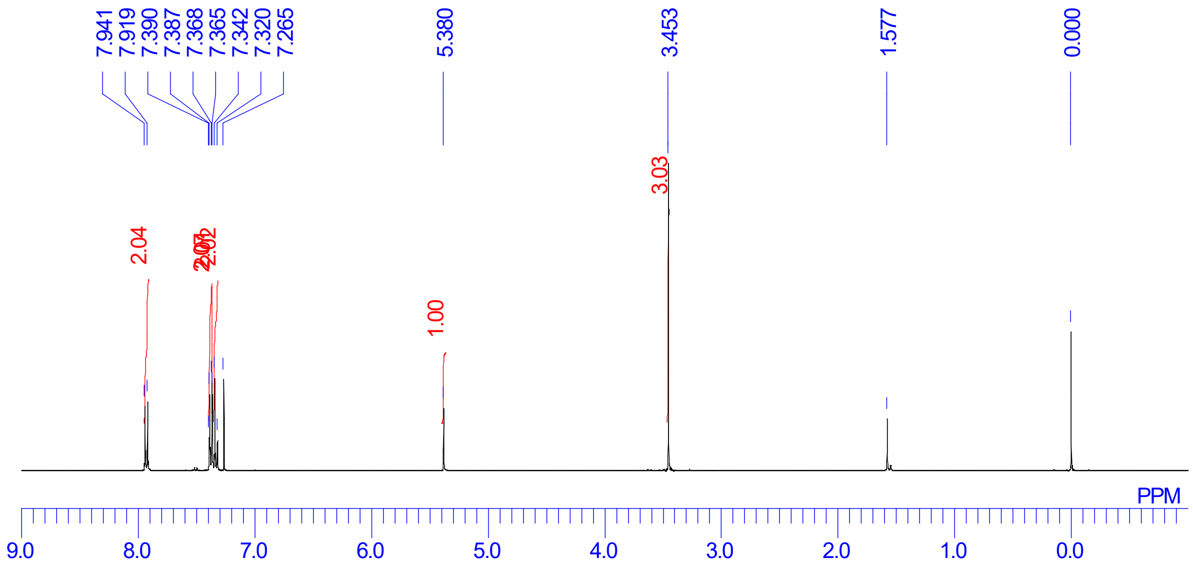

$^{13}C\{^{1}H\}$ NMR: (100 MHz, $CDCl_3$)

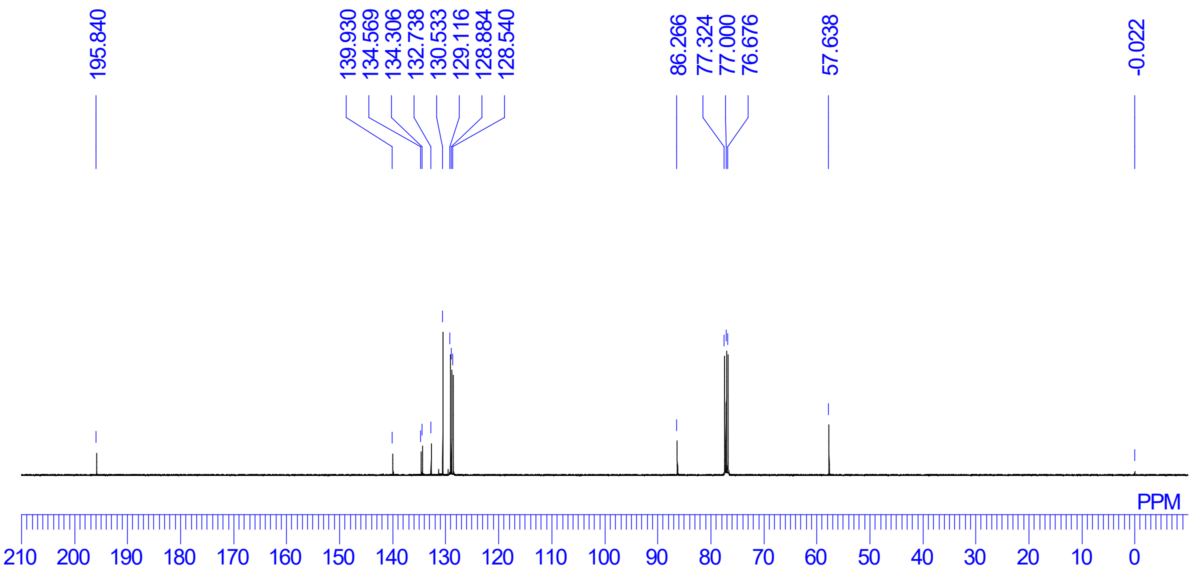

(1*S,2*R**)-1,2-Bis(4-chlorophenyl)-1-methoxypent-4-en-2-ol *syn*-3g**

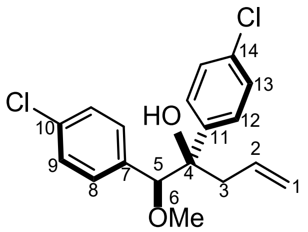

To a mixture of $SnCl_2$ (114 mg, 0.60 mmol) and 1,2-bis(4-chlorophenyl)-2-methoxyethan-1-one **2g** (148 mg, 0.50 mmol) in acetonitrile (2 mL) was added tributylallylstannane (199 mg, 0.60 mmol). After the reaction mixture was stirred for 12 h at room temperature, methanol (5 mL) was added to the mixture. The residue was evaporated to give a crude mixture, which was analyzed by ^{1}H NMR to obtain the yield and diastereoselectivity using 1,1,2,2-tetrachloroethane as an internal standard (NMR yield: 91%, *syn*/*anti* = >99/1). The obtained residue was purified by column chromatography (10% w/w anhydrous K_2CO_3-silica, hexane/ethyl acetate = 75/25) to give the product as a colorless oil (149 mg, 88%).

IR (neat) ν = 3545 (br), 3076 (w), 2980 (w), 2933 (m), 2826 (w), 1639 (w), 1597 (m), 1491 (s), 1405 (m), 1339 (m), 1177 (m), 1091 (s), 1014 (s), 929 (m), 889 (w), 783 (s), 749 (m) cm^{-1}; ^{1}H NMR (400 MHz, $CDCl_3$) 7.17 (d, J = 8.4 Hz, 2H), 7.15 (d, J = 8.0 Hz, 2H), 7.07 (d, J = 8.8 Hz, 2H), 6.91 (d, J = 8.8 Hz, 2H), 5.63–5.53 (m, 1H), 5.12–5.03 (m, 2H), 4.22 (s, 1H), 3.23 (s, 3H), 2.82–2.80 (m, 3H); $^{13}C\{^1H\}$ NMR (100 MHz, $CDCl_3$) 140.3 (s), 135.0 (s), 133.5 (s), 133.0 (d), 132.5 (s), 129.8 (d), 127.9 (d), 127.7 (d), 127.6 (d), 119.1 (t), 88.6 (d), 77.7 (s), 57.4 (q), 42.6 (t); HRMS (DART$^+$) Calculated ($C_{18}H_{17}O_2Cl_2$): 335.0600 ([M−H]$^+$), Found: 335.0597.

^{1}H NMR: (400 MHz, $CDCl_3$)

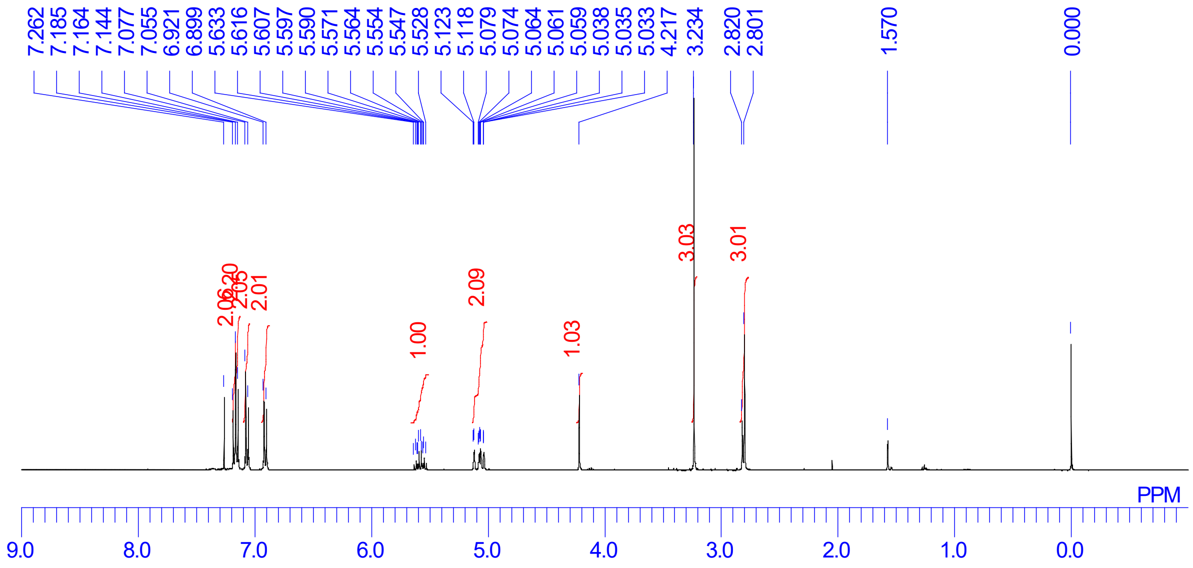

$^{13}C\{^1H\}$ NMR: (100 MHz, $CDCl_3$)

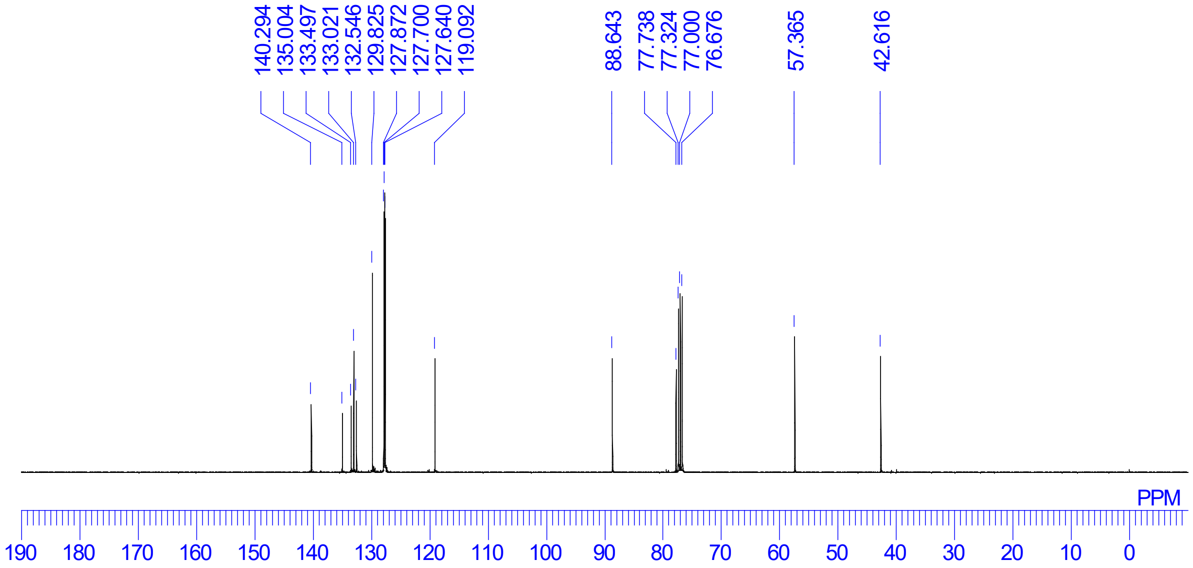

(1*S,2*S**)-1,2-Bis(4-chlorophenyl)-1-methoxypent-4-en-2-ol *anti*-3g**

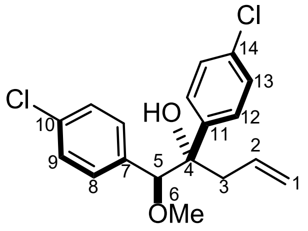

In a nitrogen-filled glovebox, to a mixture of $BF_3 \cdot Et_2O$ (28.4 mg, 0.2 mmol) and 1,2-bis(4-chlorophenyl)-2-methoxyethan-1-one **2g** (59.0 mg, 0.2 mmol) in dichloromethane (2 mL) was added **1**Si(allyl) (70.7 mg, 0.2 mmol). After the reaction mixture was stirred for 6 h at room temperature, methanol (2 mL) was added to the mixture. The residue was evaporated to give a crude mixture, which was analyzed by ^1H NMR to obtain the yield and diastereoselectivity using 1,1,2,2-tetrachloroethane as an internal standard (NMR yield: 84%, *syn*/*anti* = 3/97). The obtained residue was purified by column chromatography (hexane/ethyl acetate = 20/80) on silica gel to give product as a colorless oil (45.0 mg, 67%).

IR (neat) v = 3546 (br), 3076 (m), 2933 (s), 2826 (m), 1639 (m), 1597 (m), 1492 (s), 1405 (s), 1340 (m), 1304 (w), 1177 (m), 1091 (s), 1014 (s), 918 (m), 889 (w), 783 (s) cm^{-1}; ^1H NMR (400 MHz, $CDCl_3$) 7.23 (d, J = 8.4 Hz, 2H, 13-H), 7.21 (d, J = 8.4 Hz, 2H, 9-H), 7.16 (d, J = 8.4 Hz, 2H, 12-H), 6.90 (d, J = 8.4 Hz, 2H, 8-H), 5.60–5.49 (m, 1H, 2-H), 5.08–5.03 (m, 2H, 1-H), 4.20 (s, 1H, 5-H), 3.21 (s, 3H, 6-H), 2.79 (s, 1H, OH), 2.69 (dd, J = 14.4, 6.4 Hz, 1H, 3-H), 2.57 (d, J = 14.4, 8.0 Hz, 1H, 3-H); $^{13}C\{^1H\}$ NMR (100 MHz, $CDCl_3$) 140.5 (s, C-11), 134.9 (s, C-7), 133.8 (s, C-10), 132.88 (d, C-2), 132.86 (s, C-14), 129.9 (d, C-8), 128.3 (d, C-9), 127.9 (d, C-12), 127.7 (d, C-13), 119.3 (t, C-1), 89.5 (d, C-5), 77.7 (s, C-4), 57.5 (q, C-6), 41.1 (t, C-3); HRMS (DART$^+$) Calculated ($C_{18}H_{17}O_2Cl_2$): 335.0600 ($[M-H]^+$), Found: 335.0604.

^{1}H NMR: (400 MHz, $CDCl_3$)

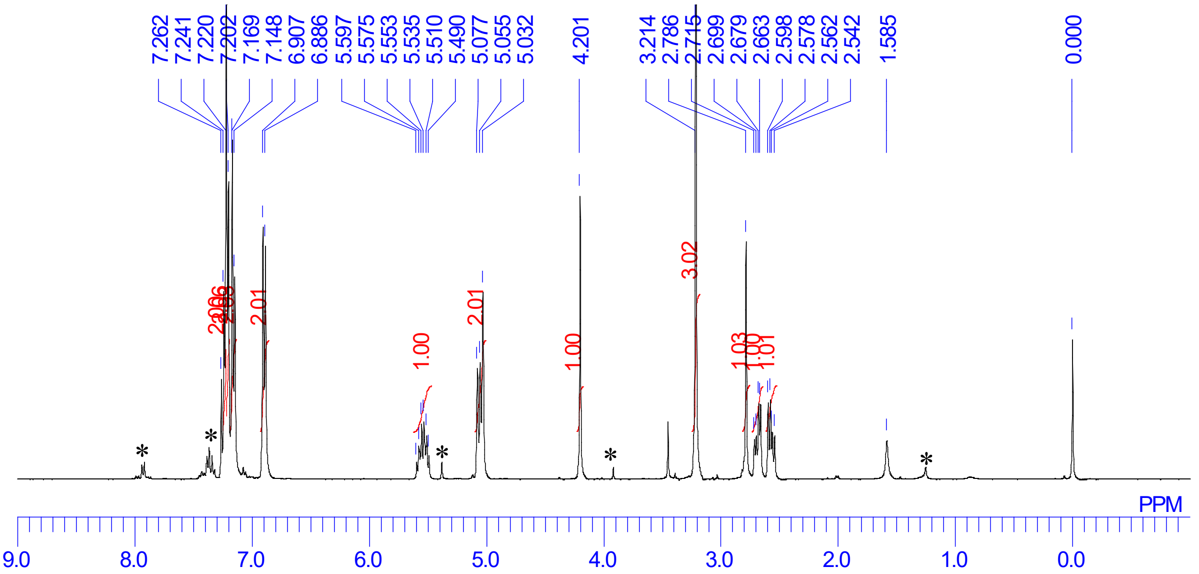

Asterisks represent inseparable impurities and residual solvents.

$^{13}C\{^{1}H\}$ NMR: (100 MHz, $CDCl_3$)

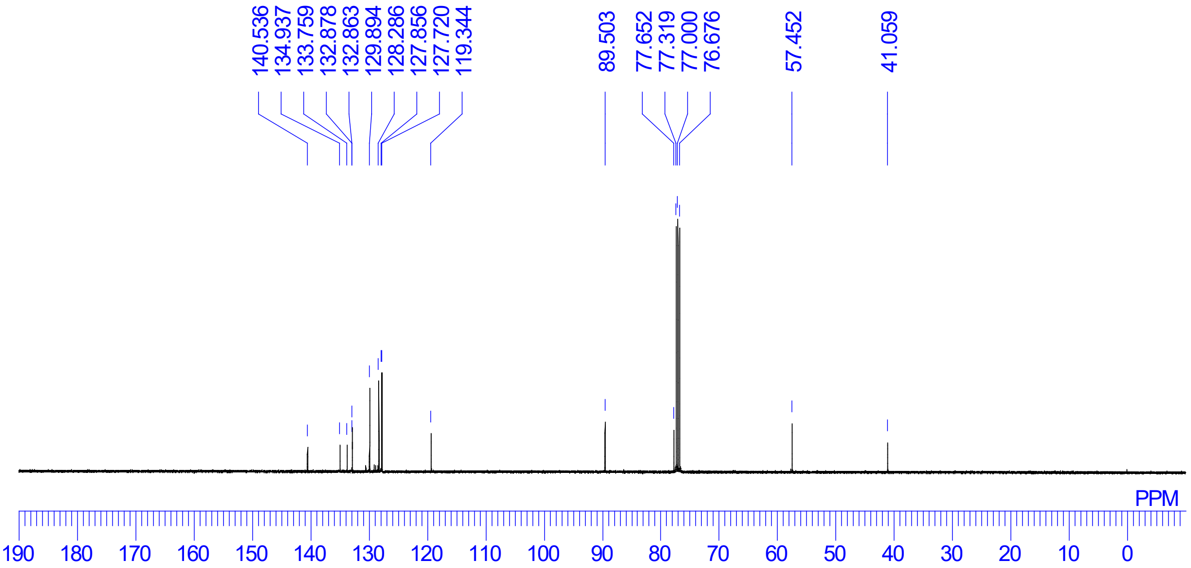

1,2-Di{(1,1'-biphenyl)-4-yl}-2-methoxyethan-1-one 2h

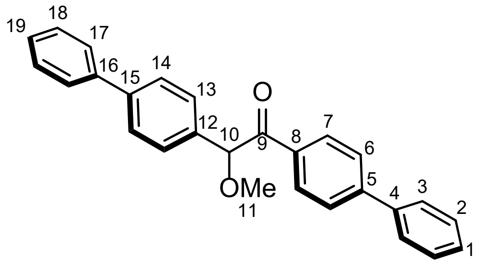

Methyl iodide (0.24 mL, 3.79 mmol) was added to a suspension of 1,2-di{(1,1'-biphenyl)-4-yl}-2-hydroxyethan-1-

one (0.23 g, 0.63 mmol) and silver(I) oxide (0.29 g, 1.26 mmol) in chloroform (10 mL). The reaction mixture was heated to reflux for 20 h. After the reaction mixture was cooled to room temperature, the resulting suspension was filtered on celite. The filtrate was dried over Na_2SO_4 and the solvent was removed in vacuum. The obtained residue was purified by column chromatography (hexane/ethyl acetate = 10/90) on silicagel to give the product as a colorless solid (0.21 g, 88%).

mp 94.8–95.2 °C; IR (KBr) ν = 3055 (w), 2924 (w), 2818 (w), 1687 (s), 1602 (s), 1486 (m), 1404 (m), 1227 (m), 1196 (s), 1105 (s), 1084 (s), 1005 (m), 944 (m), 819 (m), 764 (s) cm^{-1}; ^1H NMR (400 MHz, $CDCl_3$) 8.11 (d, J = 8.4 Hz, 2H), 7.61 (d, J = 8.4 Hz, 2H), 7.57–7.53 (m, 8H), 7.44–7.38 (m, 3H), 7.35 (d, J = 7.2 Hz, 2H), 7.31 (t, J = 7.4 Hz, 1H), 5.59 (s, 1H,), 3.50 (s, 3H); $^{13}C\{^1H\}$ NMR (100 MHz, $CDCl_3$) 196.5 (s), 145.9 (s), 141.4 (s), 140.3 (s), 139.6 (s), 135.0 (s), 133.4 (s), 129.6 (d), 128.9 (d), 128.7 (d), 128.2 (d), 128.0 (d), 127.6 (d), 127.4 (d), 127.14 (d), 127.08 (d), 126.99 (d), 86.3 (d), 57.5 (q); HRMS (MALDI-TOF MS) Calculated ($C_{27}H_{22}O_2Na$): 401.1512 ($[M+Na]^+$), Found: 401.1503.

^1H NMR: (400 MHz, $CDCl_3$)

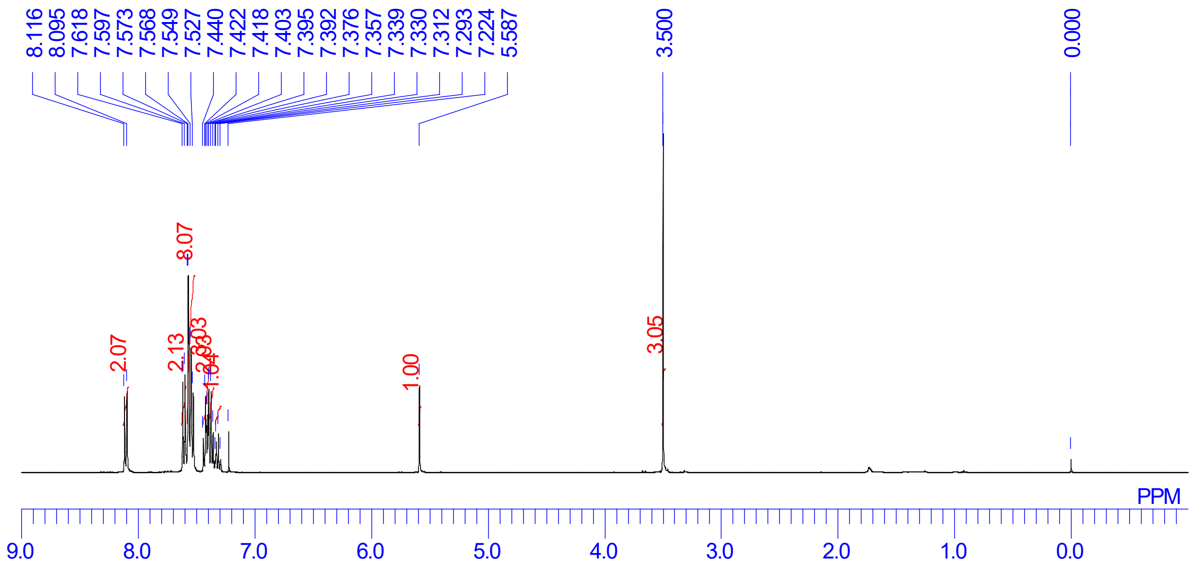

$^{13}C\{^1H\}$ NMR: (100 MHz, $CDCl_3$)

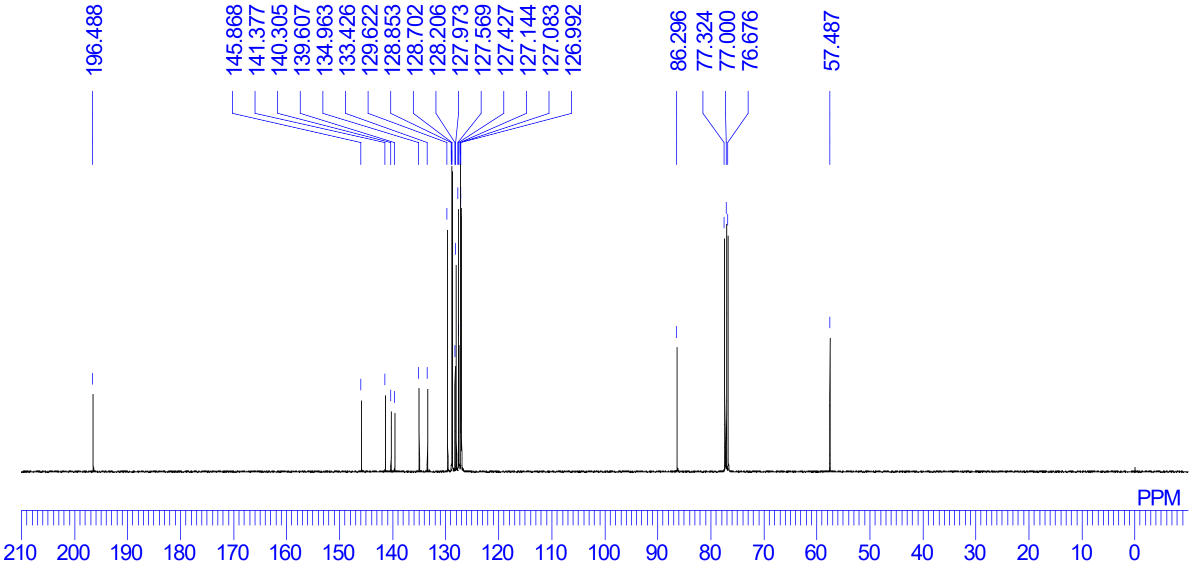

(1*S,2*R**)-1,2-Di{(1,1'-biphenyl)-4-yl}-1-methoxypent-4-en-2-ol *syn*-3h**

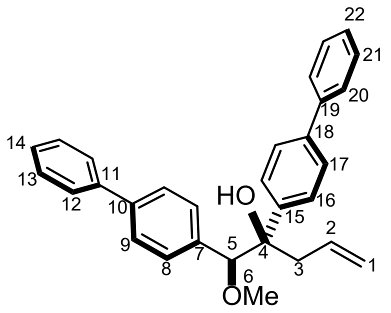

To a mixture of $SnCl_2$ (45.5 mg, 0.24 mmol) and 1,2-di{(1,1'-biphenyl)-4-yl}-2-methoxyethan-1-one **2h** (75.7 mg, 0.2 mmol) in acetonitrile (2 mL) was added tributylallylstannane (79.5 mg, 0.24 mmol). After the reaction mixture was stirred for 20 h at room temperature, methanol (2 mL) was added to the mixture. The residue was evaporated to give a crude mixture, which was analyzed by ^{1}H NMR to obtain the yield and diastereoselectivity using 1,1,2,2-tetrachloroethane as an internal standard (NMR yield: 97%, *syn*/*anti* = >99/1). The obtained residue was purified by column chromatography (10% w/w anhydrous K_2CO_3-silica, hexane/ethyl acetate = 80/20) to give the product as a colorless solid (76.2 mg, 91%).

mp 129.2–130.0 °C; IR (KBr) v = 3567 (br), 3077 (w), 3030 (m), 2955 (w), 2826 (w), 1487 (s), 1443 (m), 1403 (m), 1254 (w), 1092 (s), 974 (m), 917 (m), 849 (w), 741 (s) cm^{-1}; ^{1}H NMR (400 MHz, $CDCl_3$) 7.58 (d, J = 8.4 Hz, 2H), 7.56 (d, J = 8.4 Hz, 2H), 7.46 (d, J = 8.8 Hz, 2H), 7.43–7.39 (m, 6H), 7.32 (t, J = 7.4 Hz, 2H), 7.24 (d, J = 8.4 Hz, 2H), 7.08 (d, J = 7.6 Hz, 2H), 5.75–5.64 (m, 1H), 5.16 (dd, J = 17.4, 1.4 Hz, 1H), 5.07 (d, J = 10.4, 2.0 Hz, 1H), 4.38 (s, 1H), 3.30 (s, 3H), 2.97–2.93 (m, 2H), 2.87 (dd, J = 14.4, 8.0 Hz, 1H); $^{13}C\{^1H\}$ NMR (100 MHz, $CDCl_3$) 141.1 (s), 140.64 (s), 140.60 (s), 140.3 (s), 139.2 (s), 135.7 (s), 133.6 (d), 129.0 (d), 128.67 (d), 128.65 (d), 127.2 (d), 127.1 (d), 127.0 (d), 126.92 (d), 126.90 (d), 126.1 (d), 126.0 (d), 118.8 (t), 89.4 (d), 78.1 (s), 57.5 (q), 42.5 (t); HRMS (MALDI-TOF MS) Calculated ($C_{30}H_{28}O_2Na$): 443.1982 ([M+Na]$^+$), Found: 443.2001.

^{1}H NMR: (400 MHz, $CDCl_3$)

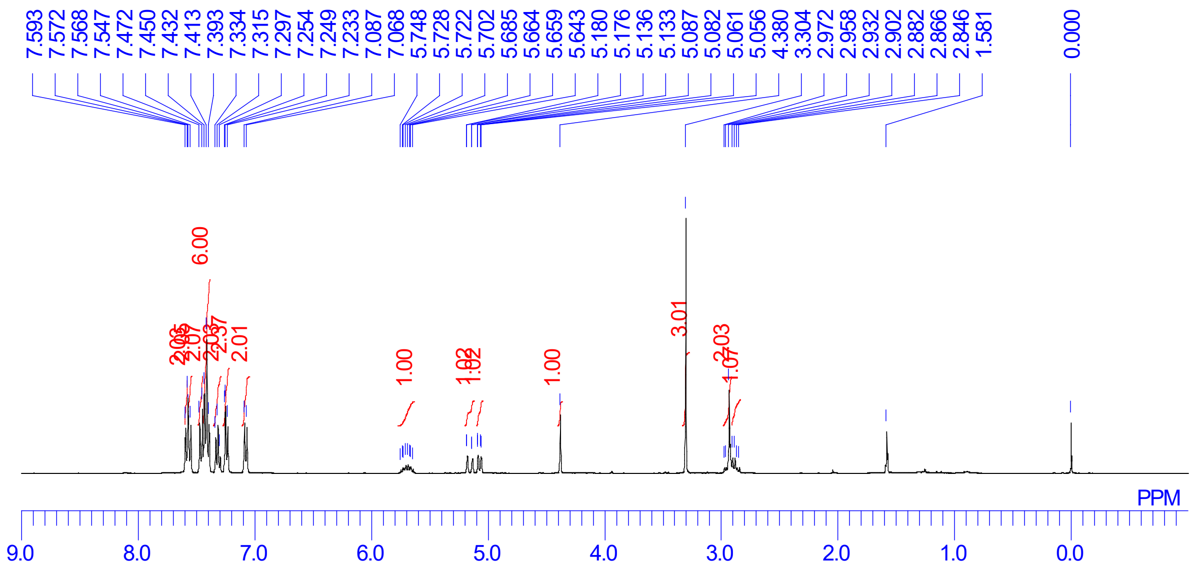

$^{13}C\{^{1}H\}$ NMR: (100 MHz, $CDCl_3$)

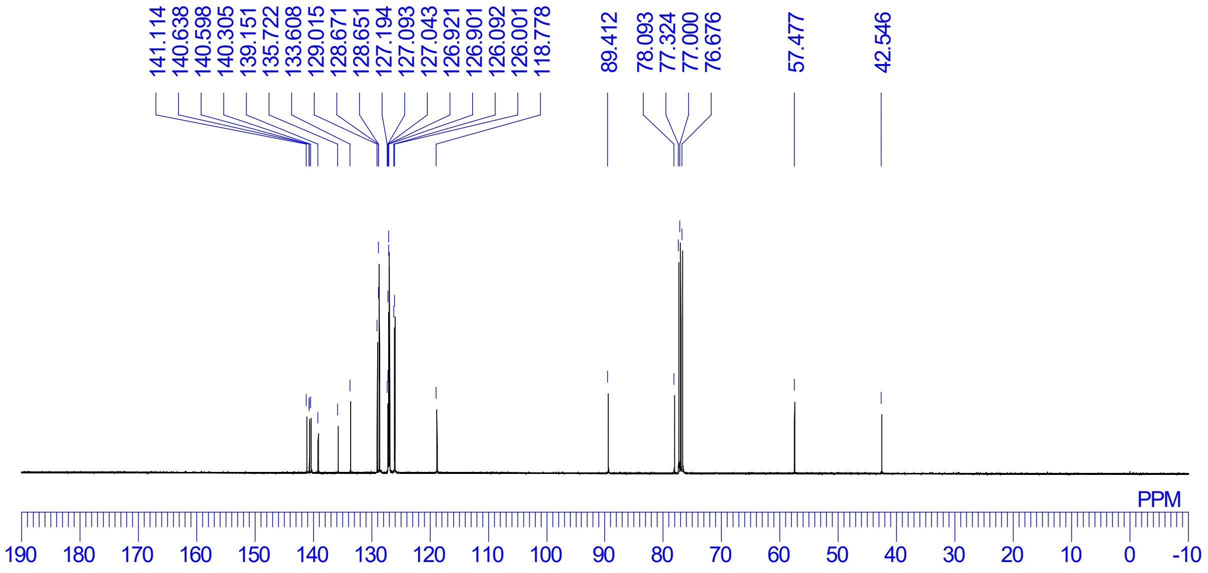

(1*S,2*S**)-1,2-Di{(1,1'-biphenyl)-4-yl}-1-methoxypent-4-en-2-ol *anti*-3h**

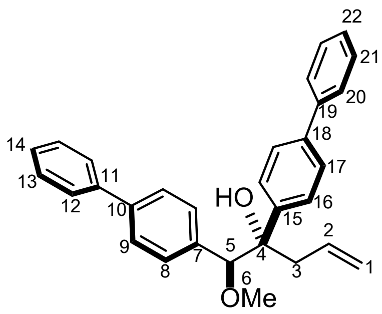

In a nitrogen-filled glovebox, to a mixture of $BF_3{\cdot}Et_2O$ (28.3 mg, 0.2 mmol) and 1,2-di{(1,1'-biphenyl)-4-yl}-2-

methoxyethan-1-one **2h** (75.7 mg, 0.2 mmol) in dichloromethane (2 mL) was added **1**Si(allyl) (106.1 mg, 0.3 mmol). After the reaction mixture was stirred for 20 h at room temperature, methanol (2 mL) was added to the mixture. The residue was evaporated to give a crude mixture, which was analyzed by ^{1}H NMR to obtain the yield and diastereoselectivity using 1,1,2,2-tetrachloroethane as an internal standard (NMR yield: 90%, *syn*/*anti* = 1/99). The obtained residue was purified by column chromatography (hexane/ethyl acetate = 70/30) on silica gel. Further purification was conducted by a recycle GPC to give product as a colorless solid (84.1 mg, 82%).
mp 134.2–134.6°C; IR (KBr) v = 3503 (br), 3055 (w), 3029 (w), 2819 (w), 1597 (w), 1486 (s), 1406 (m), 1195 (w), 1077 (s), 1005 (m), 916 (m), 849 (m), 751 (s) cm^{-1}; ^{1}H NMR (400 MHz, acetone-d_6) 7.67 (d, J = 8.0 Hz, 2H), 7.65 (d, J = 8.0 Hz, 2H), 7.56 (d, J = 8.4 Hz, 2H), 7.53 (d, J = 7.2 Hz, 2H), 7.48–7.42 (m, 6H), 7.33 (t, J = 7.4 Hz, 2H), 7.21 (d, J = 7.6 Hz, 2H), 5.79–5.68 (m, 1H, 2-H), 5.01 (d, J = 18.0 Hz, 1H, 1-H), 4.93 (d, J = 10.4 Hz, 1H, 1-H), 4.50 (s, 1H, 5-H), 4.08 (s, 1H, OH), 3.20 (s, 3H, 6-H), 2.82 (dd, J = 14.2, 7.0 Hz, 1H, 3-H), 2.68 (dd, J = 14.4, 6.8 Hz, 1H, 3-H); ^{13}C{^{1}H} NMR (100 MHz, acetone-d_6) 143.8 (s, C-15), 141.6 (s), 141.5 (s), 140.8 (s), 139.6 (s), 137.9 (s, C-7), 135.1 (d, C-2), 130.4 (d), 129.63 (d), 129.61 (d), 128.4 (d), 128.0 (d), 127.9 (d), 127.55 (d), 127.52 (d), 126.5 (d), 126.3 (d), 117.9 (t, C-1), 90.4 (d, C-5), 78.8 (s, C-4), 57.4 (q, C-6), 43.6 (t, C-3); HRMS (MALDI-TOF MS) Calculated ($C_{30}H_{28}O_2Na$): 443.1982 ([M+Na]$^{+}$), Found: 443.1979.

^{1}H NMR: (400 MHz, acetone-d_6)

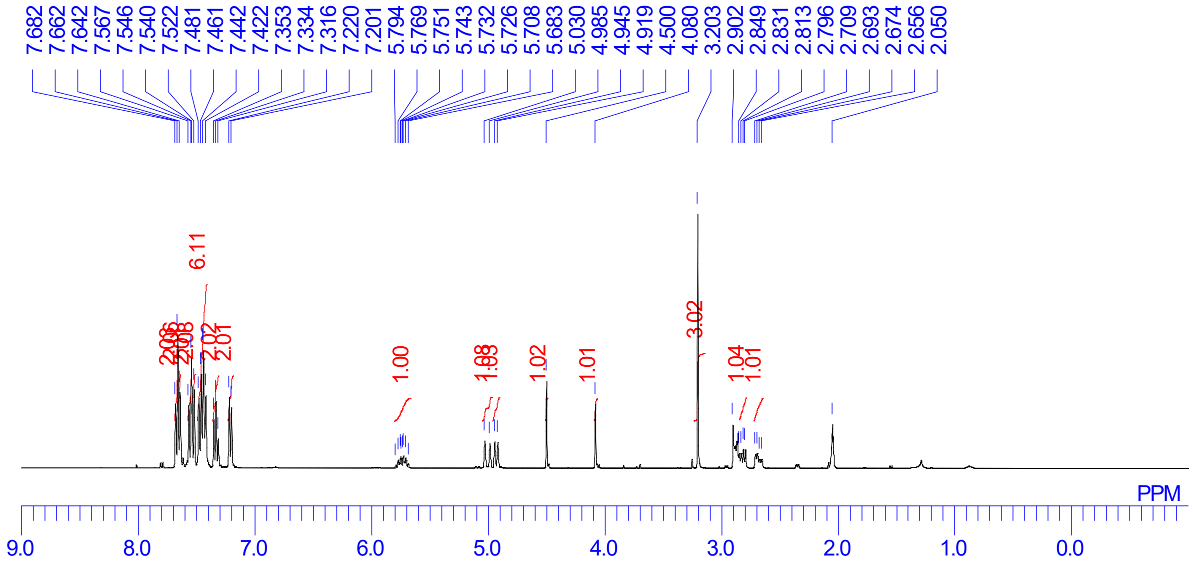

$^{13}C\{^1H\}$ NMR: (100 MHz, acetone-d_6)

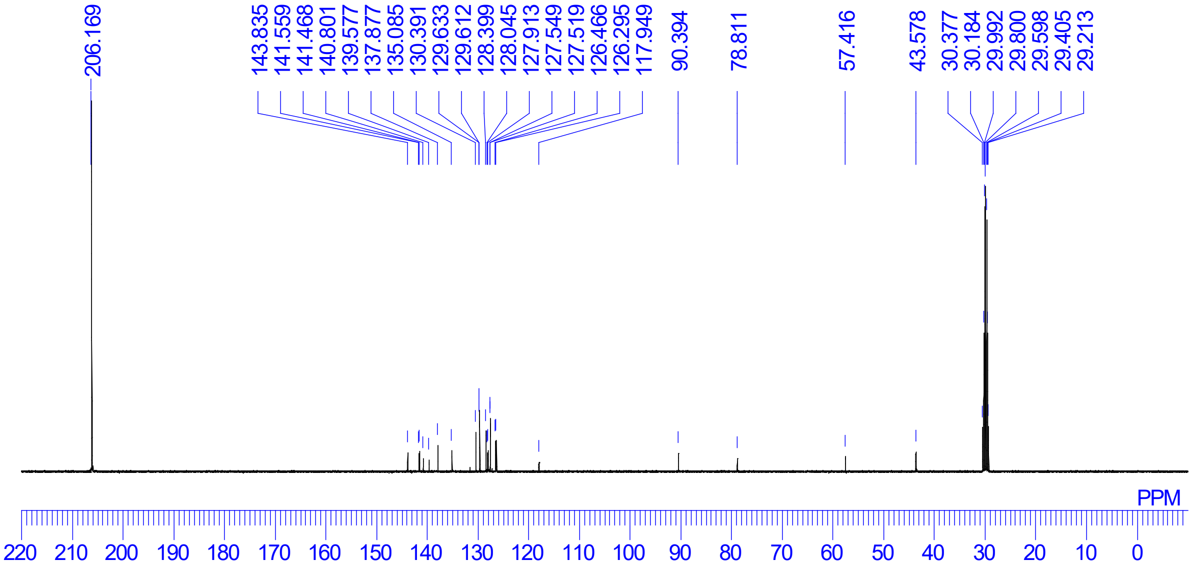

2-Methoxy-1,2-di(naphthalen-2-yl)ethan-1-one 2i

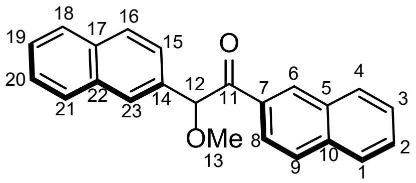

Methyl iodide (0.81 mL, 13.0 mmol) was added to a suspension of 2-hydroxy-1,2-di(naphthalen-2-yl)ethan-1-one (0.68 g, 2.17 mmol) and silver(I) oxide (1.01 g, 4.34 mmol) in chloroform (10 mL). The reaction mixture was heated to reflux for 20 h. After the reaction mixture was cooled to room temperature, the resulting suspension was filtered on celite. The filtrate was dried over Na_2SO_4 and the solvent was removed in vacuum. The obtained residue was purified by column chromatography (hexane/ethyl acetate = 40/60) on silicagel to give the product as a colorless oil (0.39 g, 54%).

IR (neat) ν = 3058 (s), 3010 (s), 2931 (s), 2825 (s), 1686 (s), 1597 (m), 1575 (w), 1508 (m), 1467 (m), 1361 (m), 1280 (m), 1186 (s), 1101 (s), 966 (w), 904 (w), 861 (m), 804 (s), 747 (s) cm^{-1}; ^1H NMR (400 MHz, $CDCl_3$) 8.61 (s, 1H), 8.05 (dd, J = 9.0, 1.4 Hz, 1H), 8.01 (s, 1H), 7.90 (d, J = 8.0 Hz, 1H), 7.84–7.78 (m, 5H), 7.61 (dd, J = 8.2, 1.4 Hz, 1H), 7.56 (t, J = 7.0 Hz, 1H), 7.52–7.45 (m, 3H), 5.83 (s, 1H), 3.55 (s, 3H); $^{13}C\{^1H\}$ NMR (100 MHz, $CDCl_3$) 197.0 (s), 135.5 (s), 133.5 (s), 133.24 (s), 133.22 (s), 132.3 (s), 132.2 (s), 131.0 (d), 129.7 (d), 128.8 (d), 128.6 (d), 128.3 (d), 128.1 (d), 127.7 (d), 127.20 (d), 127.18 (d), 126.7 (d), 126.5 (d), 126.3 (d), 124.8 (d), 124.5 (d), 86.7 (d), 57.5 (q); HRMS (MALDI-TOF MS) Calculated ($C_{23}H_{18}O_2Na$): 349.1199 ($[M+Na]^+$), Found: 349.1190.

^{1}H NMR: (400 MHz, $CDCl_3$)

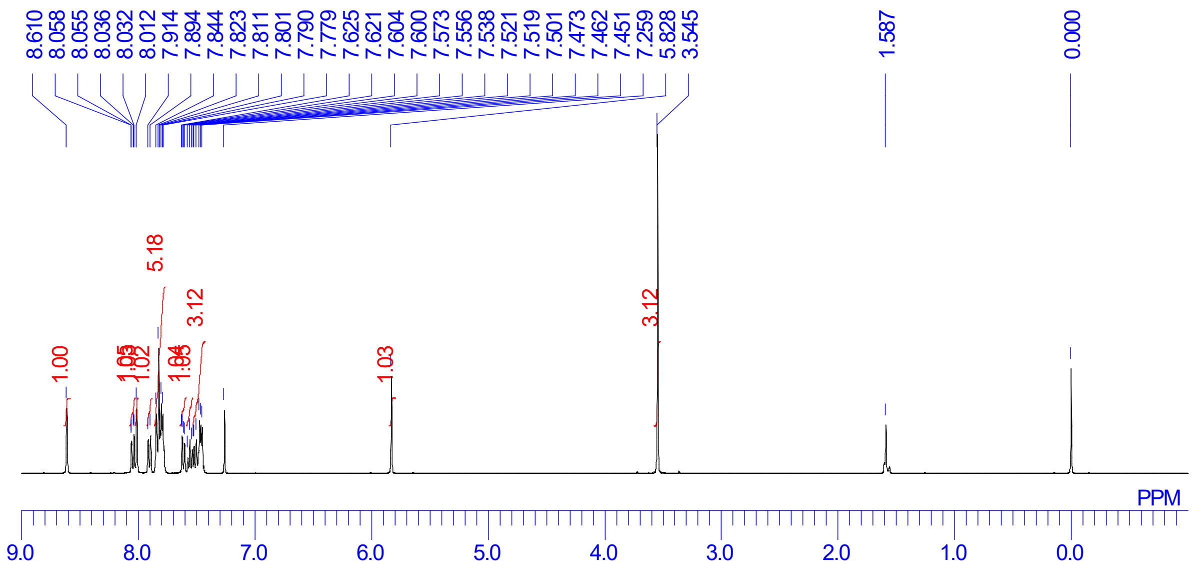

$^{13}C\{^{1}H\}$ NMR: (100 MHz, $CDCl_3$)

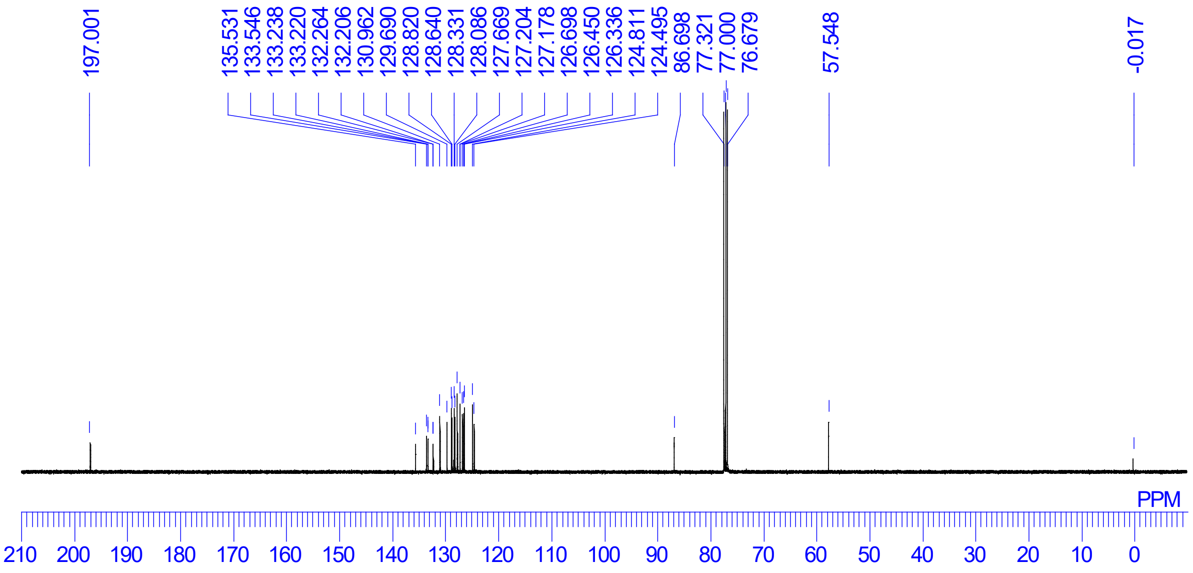

(1*S,2*R**)-1-Methoxy-1,2-di(naphthalen-2-yl)pent-4-en-2-ol *syn*-3i**

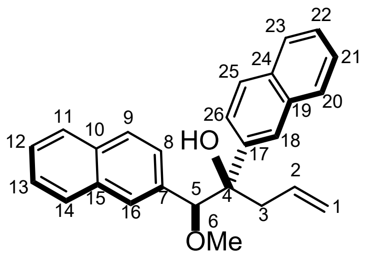

To a mixture of $SnCl_2$ (45.5 mg, 0.24 mmol) and 2-methoxy-1,2-di(naphthalen-2-yl)ethan-1-one **2i** (65.3 mg, 0.2 mmol) in acetonitrile (2 mL) was added tributylallylstannane (79.5 mg, 0.24 mmol). After the reaction mixture was

stirred for 12 h at room temperature, methanol (2 mL) was added to the mixture. The residue was evaporated to give a crude mixture, which was analyzed by ^{1}H NMR to obtain the yield and diastereoselectivity using 1,1,2,2-tetrachloroethane as an internal standard (NMR yield: 100%, *syn*/*anti* = >99/1). The obtained residue was purified by column chromatography (10% w/w anhydrous K_2CO_3-silica, hexane/ethyl acetate = 75/25) to give the product as a colorless oil (70.0 mg, 95%).

IR (neat) v = 3551 (br), 3057 (s), 3015 (s), 2979 (s), 2931 (s), 2825 (s), 1731 (w), 1637 (w), 1601 (m), 1507 (m), 1444 (m), 1370 (m), 1271 (m), 1165 (m), 1122 (m), 1092 (s), 1046 (w), 912 (m), 820 (m), 750 (m) cm^{-1}; ^{1}H NMR (400 MHz, $CDCl_3$) 7.77–7.73 (m, 2H), 7.694–7.686 (m, 3H), 7.66 (d, J = 8.8 Hz, 1H), 7.59–7.57 (m, 2H), 7.44–7.38 (m, 4H), 7.29 (dd, J = 8.6, 1.8 Hz, 1H), 7.06 (dd, J = 8.4, 1.6 Hz, 1H), 5.67–5.57 (m, 1H), 5.12 (d, J = 17.2 Hz, 1H), 5.01 (d, J = 10.4 Hz, 1H), 4.59 (s, 1H), 3.29 (s, 3H), 3.07–3.02 (m, 2H), 2.93 (dd, J = 14.4, 8.4 Hz, 1H); ^{13}C{^{1}H} NMR (100 MHz, $CDCl_3$) 139.7 (s), 134.4 (s), 133.5 (d), 133.0 (s), 132.8 (s), 132.6 (s), 132.2 (s), 128.2 (d), 128.0 (d), 127.9 (d), 127.5 (d), 127.3 (d), 127.0 (d), 126.8 (d), 126.4 (d), 125.9 (d), 125.8 (d), 125.7 (d, Two signals were overlapped.), 125.6 (d), 124.9 (d), 118.8 (t), 89.7 (d), 78.4 (s), 57.5 (q), 42.7 (t); HRMS (MALDI-TOF MS) Calculated ($C_{26}H_{24}O_2Na$): 391.1669 ([M+Na]$^{+}$), Found: 391.1651.

^{1}H NMR: (400 MHz, $CDCl_3$)

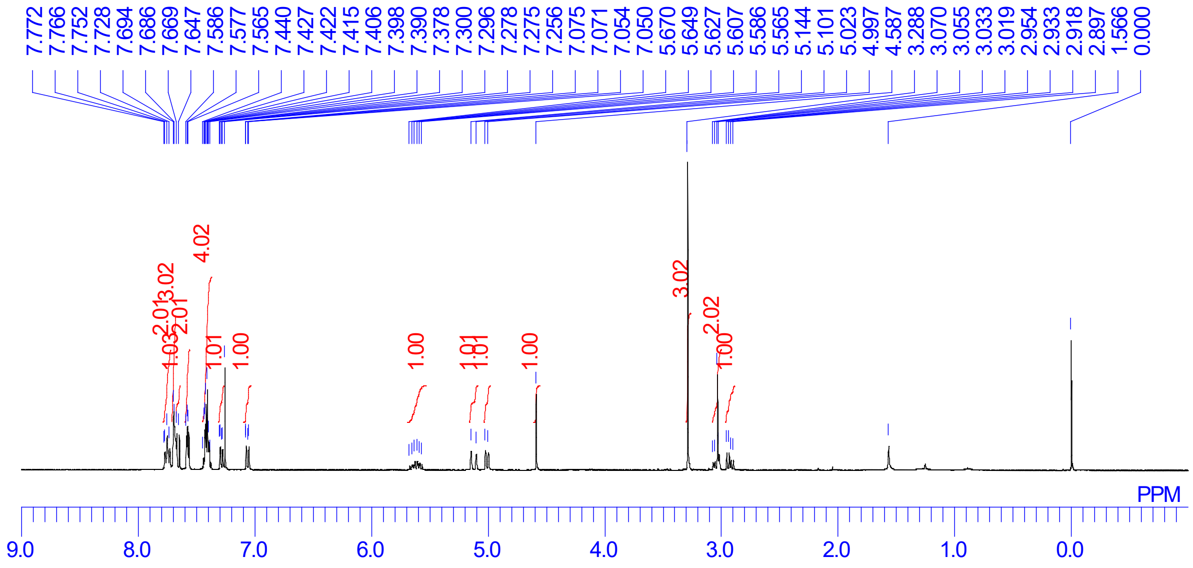

$^{13}C\{^1H\}$ NMR: (100 MHz, $CDCl_3$)

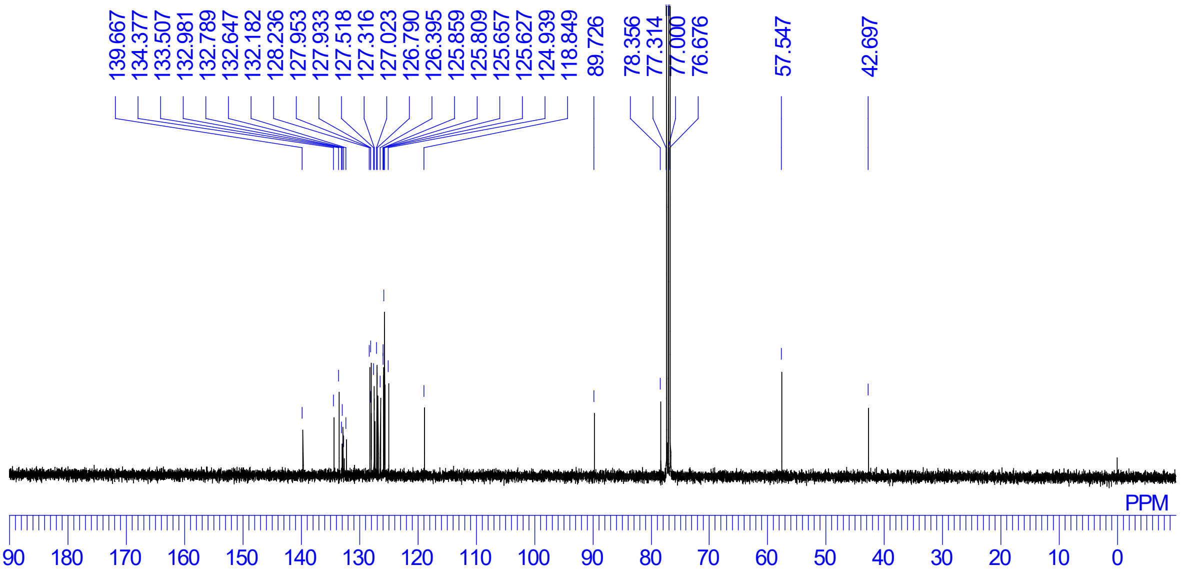

(1*S,2*S**)-1-Methoxy-1,2-di(naphthalen-2-yl)pent-4-en-2-ol *anti*-3i**

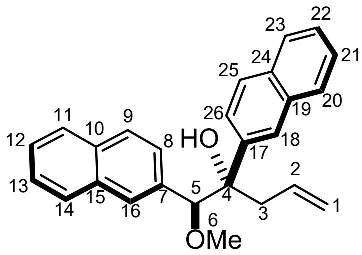

In a nitrogen-filled glovebox, to a mixture of $BF_3{\cdot}Et_2O$ (28.3 mg, 0.2 mmol) and 2-methoxy-1,2-di(naphthalen-2-yl)ethan-1-one **2i** (65.3 mg, 0.2 mmol) in dichloromethane (2 mL) was added **1**Si(allyl) (70.7 mg, 0.2 mmol). After the reaction mixture was stirred for 6 h at room temperature, methanol (2 mL) was added to the mixture. The residue was evaporated to give a crude mixture, which was analyzed by ^{1}H NMR to obtain the yield and diastereoselectivity using 1,1,2,2-tetrachloroethane as an internal standard (NMR yield: 92%, *syn*/*anti* = 4/96). The obtained residue was purified by column chromatography (hexane/ethyl acetate = 20/80) on silica gel to give product as a colorless solid (59.5 mg, 81%).

mp 102.8–103.7 °C; IR (KBr) v = 3554 (br), 3056 (m), 2928 (m), 2822 (w), 1600 (w), 1507 (w), 1433 (w), 1166 (w), 1087 (s), 917 (m), 822 (m), 756 (s) cm^{-1}; ^{1}H NMR (400 MHz, $CDCl_3$) 7.85 (s, 1H, 18-H), 7.83–7.81 (m, 2H), 7.78–7.75 (m, 2H), 7.73 (d, J = 8.8 Hz, 1H, 25-H), 7.68 (d, J = 8.4 Hz, 1H, 9-H), 7.59 (s, 1H, 16-H), 7.48–7.45 (m, 4H), 7.35 (d, J = 9.2 Hz, 1H, 26-H), 7.12 (d, J = 8.4 Hz, 1H, 8-H), 5.61–5.50 (m, 1H, 2-H), 5.01 (d, J = 17.2 Hz, 1H, 1-H), 4.95 (d, J = 10.4 Hz, 1H, 1-H), 4.56 (s, 1H, 5-H), 3.25 (s, 3H, 6-H), 3.11 (s, 3H, OH), 2.84 (dd, J = 14.6, 6.2 Hz, 1H, 3-H), 2.64 (dd, J = 14.2, 7.8 Hz, 1H, 3-H); $^{13}C\{^1H\}$ NMR (100 MHz, $CDCl_3$) 140.2 (s, C-17), 134.3 (s, C-7), 133.4 (d, C-2), 133.2 (s), 132.9 (s), 132.7 (s), 132.4 (s), 128.3 (d), 128.2 (d), 127.9 (d), 127.6 (d), 127.4 (d), 127.3 (d), 127.1 (d), 126.3 (d), 126.01 (d), 125.98 (d), 125.83 (d), 125.76 (d), 125.73 (d), 124.9 (d, C-26), 118.7 (t, C-1), 90.5 (d, C-5), 78.5 (s, C-4), 57.5 (q, C-6), 41.3 (t, C-3); HRMS (MALDI-TOF MS) Calculated ($C_{26}H_{24}O_2Na$): 391.1669 ([M+Na]$^+$), Found: 391.1656.

^{1}H NMR: (400 MHz, $CDCl_3$)

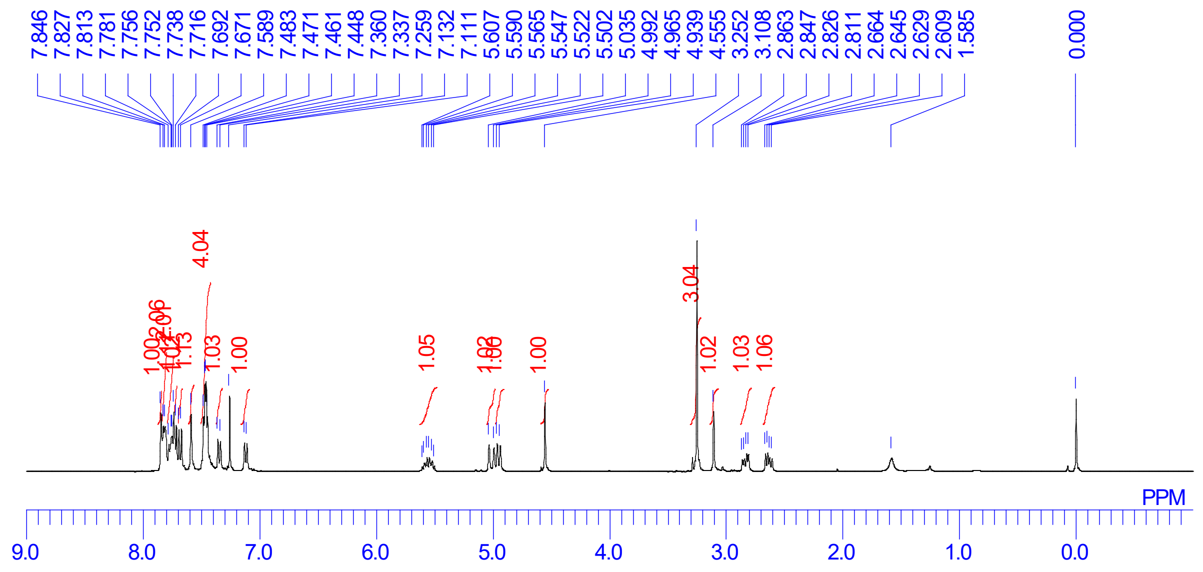

^{13}C{^{1}H} NMR: (100 MHz, $CDCl_3$)

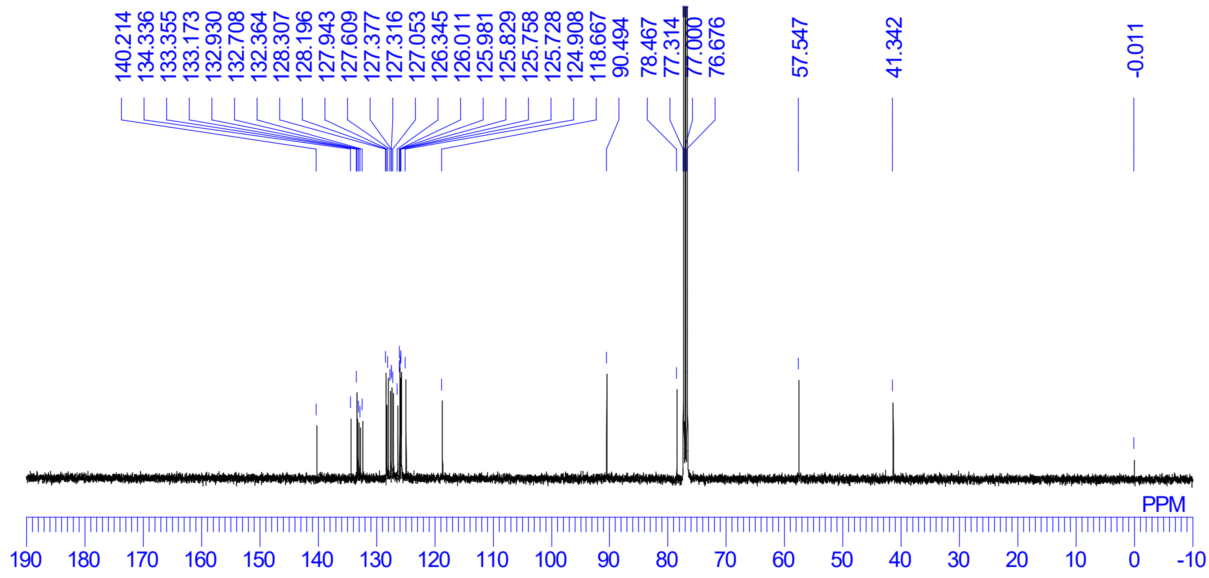

(*S)-1-phenyl-1-[(2*S**,5*S**)-5-{(triisopropylsilyl)methyl}tetrahydrofuran-2-yl]but-3-en-1-ol *anti*-3k**

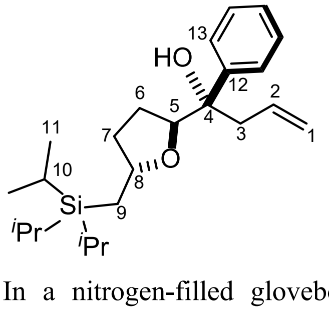

In a nitrogen-filled glovebox, to a mixture of $BF_3 \cdot Et_2O$ (14.2 mg, 0.10 mmol) and phenyl[(2*S**,5*S**)-5-{(triisopropylsilyl)methyl}tetrahydrofuran-2-yl]methanone **2k** (34.7 mg, 0.10 mmol) in dichloromethane (1 mL)

was added **1**Si(allyl) (53.0 mg, 0.15 mmol) at −20 °C. After the reaction mixture was stirred for 24 h, methanol (1 mL) was added to the mixture. The residue was evaporated to give a crude mixture, which was analyzed by ^{1}H NMR to obtain the yield and diastereoselectivity using 1,1,2,2-tetrachloroethane as an internal standard (NMR yield: 88%, *syn*/*anti* = 2/98). The obtained residue was purified by column chromatography (hexane/ethyl acetate = 90/10) on silica gel. Further purification was conducted by a recycle GPC to give product as a colorless oil (33.8 mg, 87%). The NMR data were consistent with the data previously reported.[8]

^{1}H NMR (400 MHz, $CDCl_3$) 7.47 (dd, *J* = 8.6, 1.4 Hz, 2H), 7.31 (t, *J* = 7.6 Hz, 2H), 7.22 (t, *J* = 7.4 Hz, 1H), 5.59–5.49 (m, 1H), 5.08 (dd, *J* = 17.2, 2.0 Hz, 1H), 5.02 (dd, *J* = 10.2, 2.2 Hz, 1H), 4.23 (dd, *J* = 9.2, 6.0 Hz, 1H), 4.02–3.95 (m, 1H), 2.80 (dd, *J* = 13.8, 6.6 Hz, 1H), 2.54 (dd, *J* = 14.2, 7.8 Hz, 1H), 2.45 (s, 1H), 1.98–1.90 (m, 2H), 1.87–1.77 (m, 1H), 1.49–1.40 (m, 1H), 1.07–0.94 (m, 22H), 0.83 (dd, *J* = 14.2, 6.6 Hz, 1H); $^{13}C\{^1H\}$ NMR (100 MHz, $CDCl_3$) 143.8 (s), 133.4 (d), 127.7 (d), 126.6 (d), 126.2 (d), 118.7 (t), 84.3 (d, 78.2 (d), 76.8 (s), 43.1 (t), 36.2 (t), 27.5 (t), 18.8 (q), 17.3 (t), 11.3 (d); $^{29}Si\{^1H\}$ NMR (78.7 MHz, $CDCl_3$, Me_4Si in $CDCl_3$ as an external standard) 5.36.

^{1}H NMR: (400 MHz, $CDCl_3$)

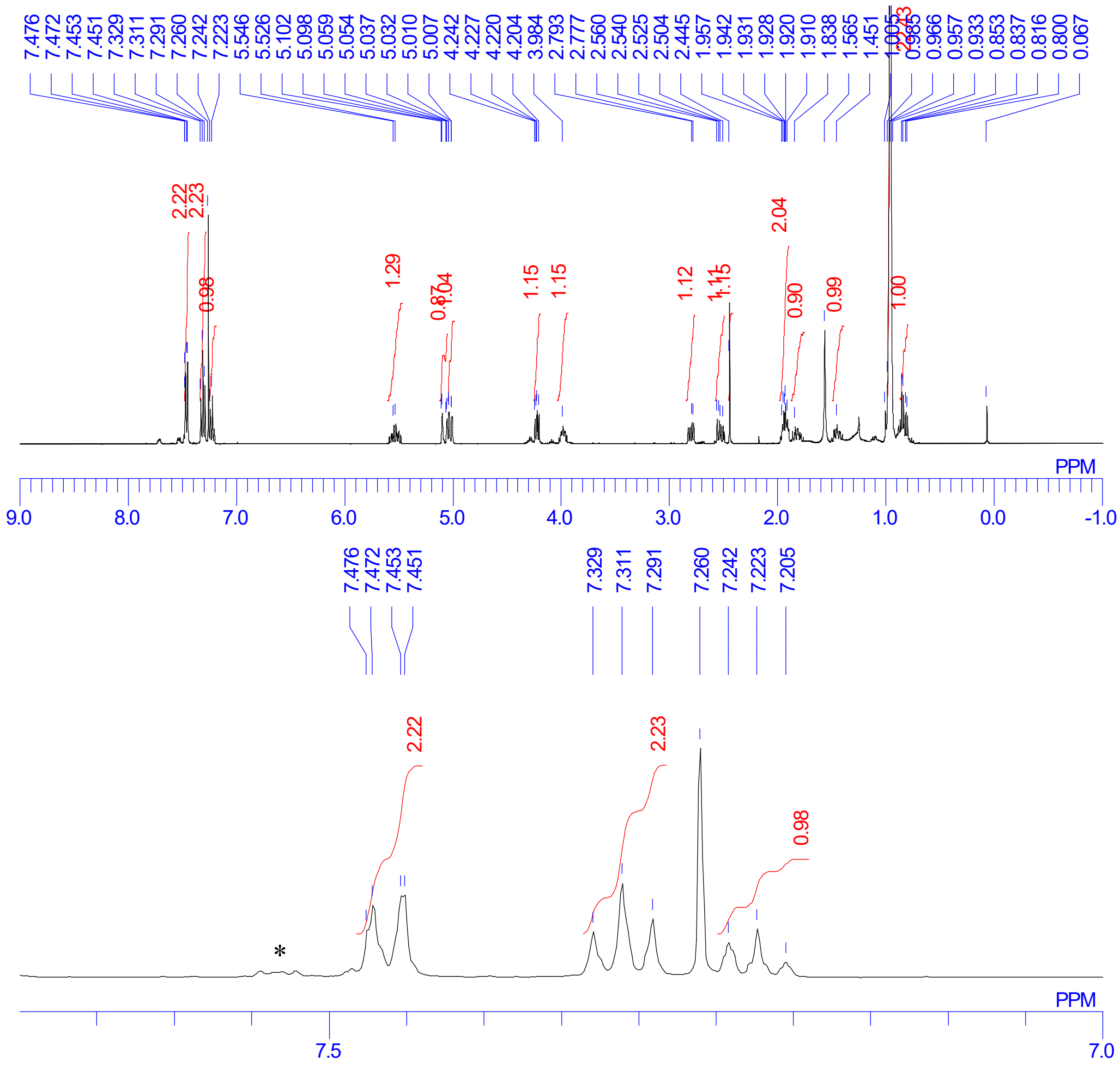

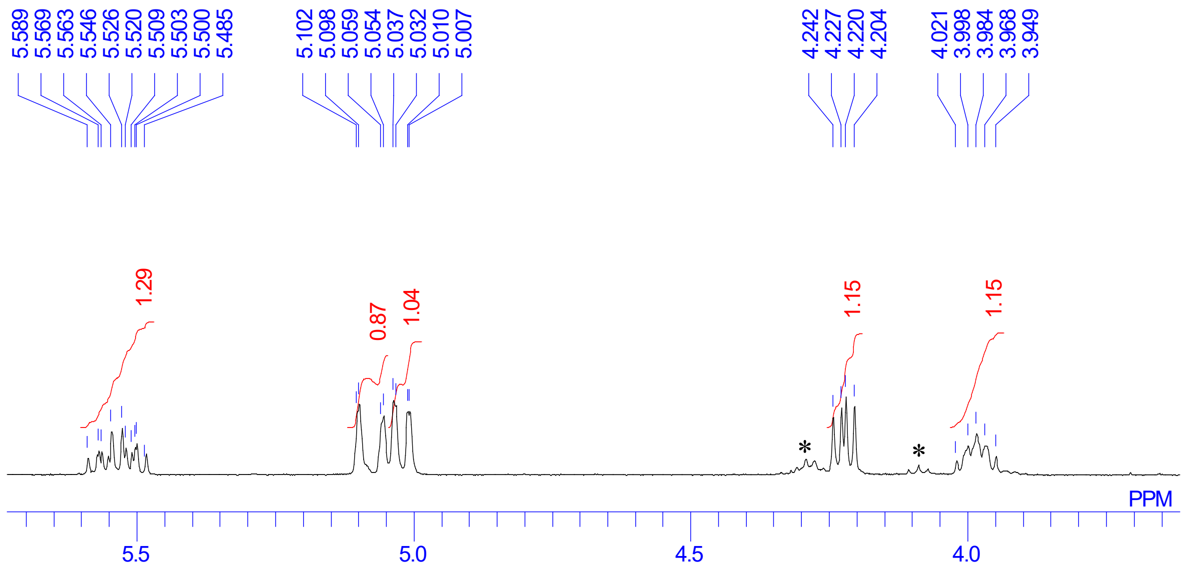

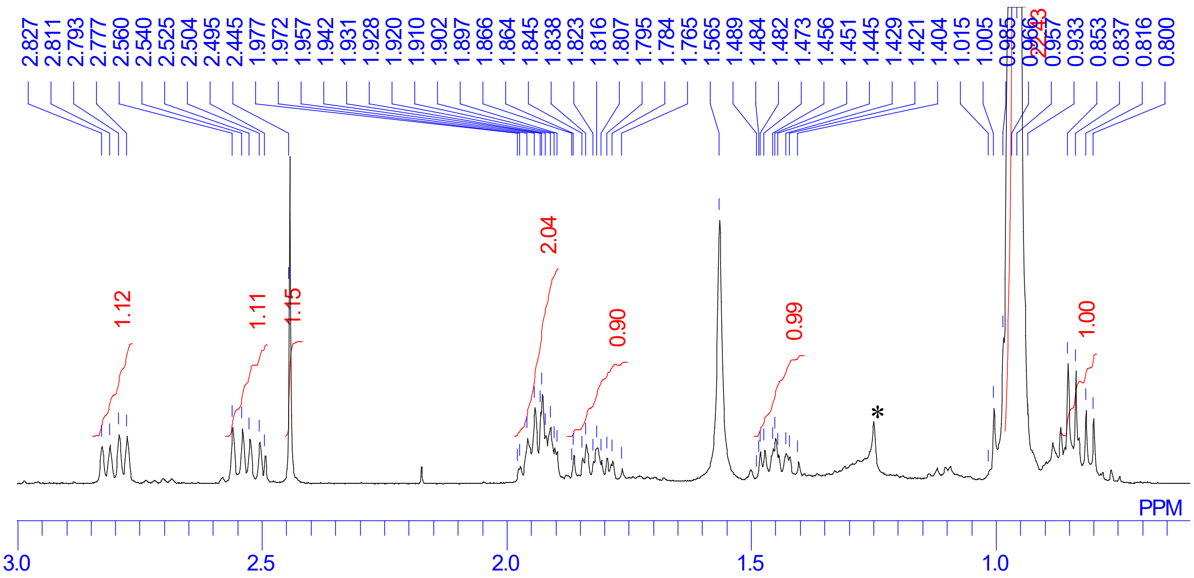

Asterisks represent inseparable impurities and residual solvents.

^{13}C NMR: (100 MHz, $CDCl_3$)

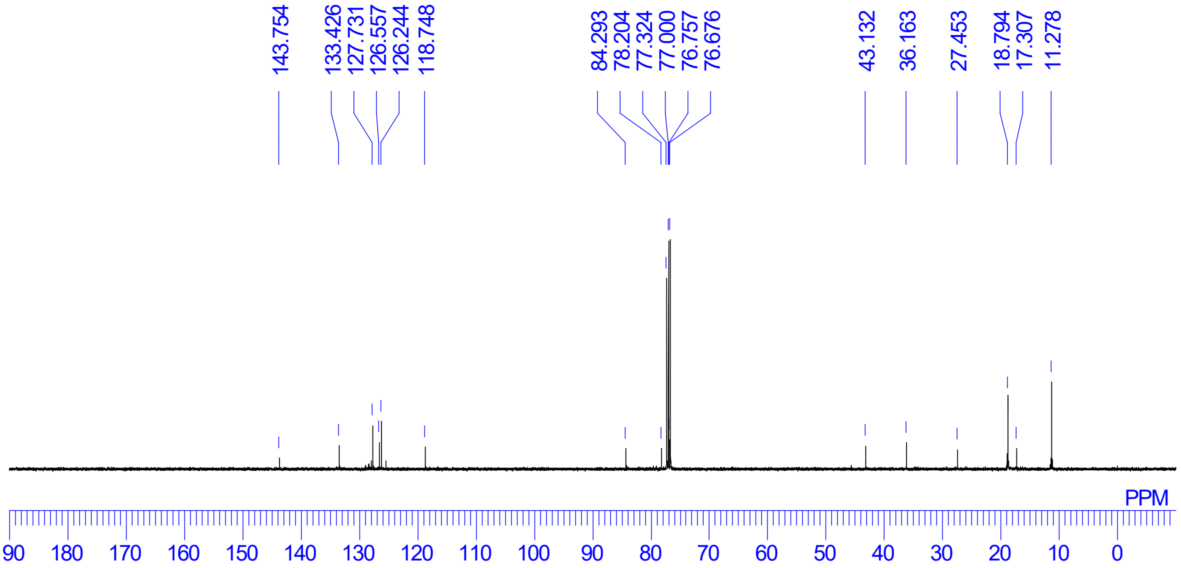

^{29}Si{^{1}H} NMR: (78.7 MHz, $CDCl_3$, Me_4Si in $CDCl_3$ as an external standard)

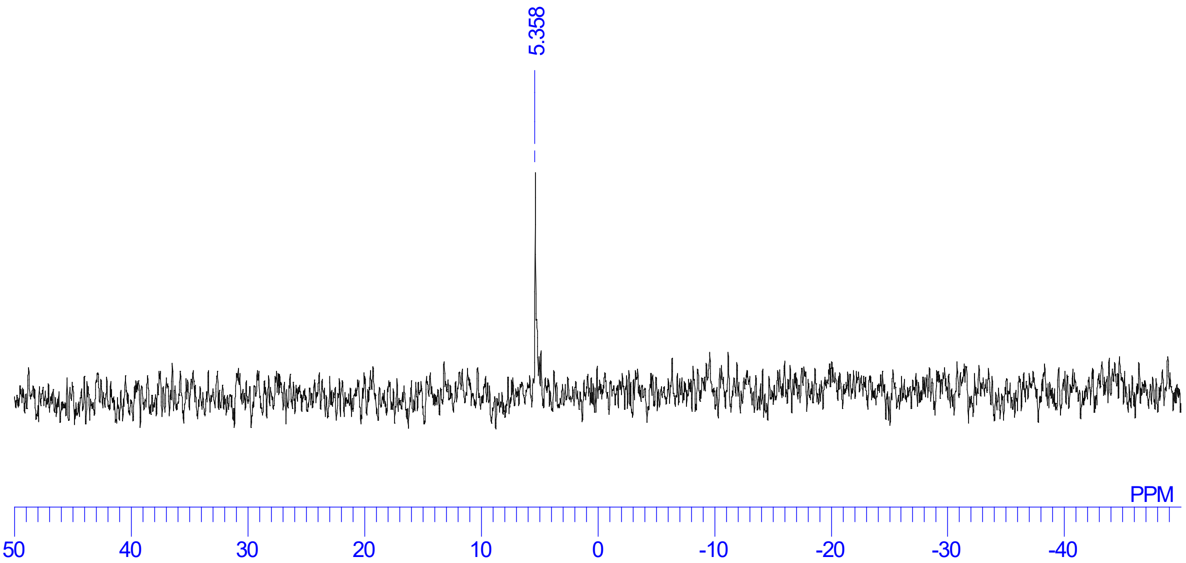

(1*S,2*S**,4*S**)-1-allyl-4-(*tert*-butyl)-2-methoxycyclohexan-1-ol *cis*-3o**

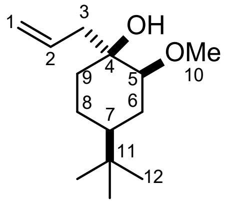

In a nitrogen-filled glovebox, to a mixture of $BF_3{\cdot}Et_2O$ (14.2 mg, 0.1 mmol) and (2*S**,4*S**)-4-(*tert*-butyl)-2-methoxycyclohexan-1-one **2o** (18.4 mg, 0.1 mmol) in dichloromethane (1 mL) was added **1**Si(allyl) (35.4 mg, 0.1 mmol). After the reaction mixture was stirred for 3 h at room temperature, methanol (1 mL) was added to the mixture.

The residue was evaporated to give a crude mixture, which was analyzed by ^{1}H NMR to obtain the yield and diastereoselectivity using 1,1,2,2-tetrachloroethane as an internal standard (NMR yield: 79%, *cis*/*trans* = 96/4). The obtained residue was purified by column chromatography (hexane/ethyl acetate = 75/25) on silica gel to give product as a colorless oil (16.6 mg, 73%). The NMR data were consistent with the data previously reported.[14]

^{1}H NMR (400 MHz, benzene-d_6) 5.98–5.87 (m, 1H), 5.11–5.06 (m, 2H), 3.07 (s, 3H), 2.74 (dd, J = 11.2, 4.8 Hz, 1H), 2.46 (dd, J = 13.6, 6.4 Hz, 1H), 2.26 (dd, J = 14.0, 8.4 Hz, 1H), 1.98 (d, J = 2.4 Hz, 1H), 1.88–1.82 (m, 2H), 1.40–1.31 (m, 1H), 1.39–1.30 (m, 2H), 1.06 (td, J = 13.8, 4.3 Hz, 1H), 0.86 (s, 9H), 0.79 (tt, J = 12.6, 3.0 Hz, 1H); ^{13}C{^{1}H} NMR (100 MHz, benzene-d_6) 135.3 (d), 117.3 (t), 82.8 (d), 72.5 (s), 56.2 (q), 46.4 (d), 45.3 (t), 34.3 (t), 32.4 (s), 27.7 (q), 26.8 (t), 22.0 (t).

^{1}H NMR: (400 MHz, benzene-d_6)

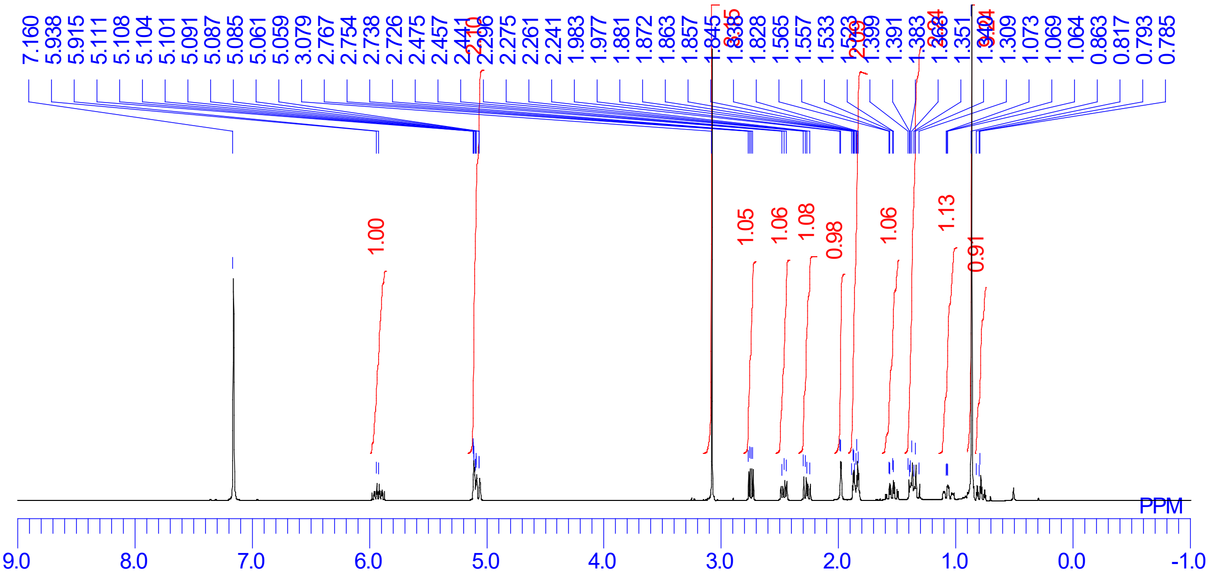

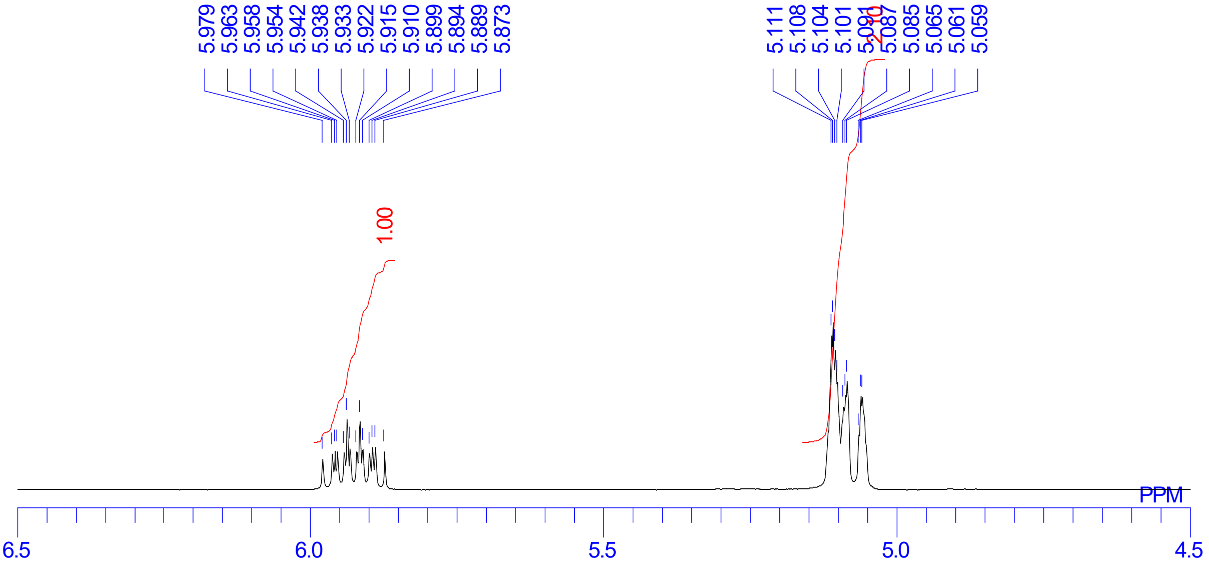

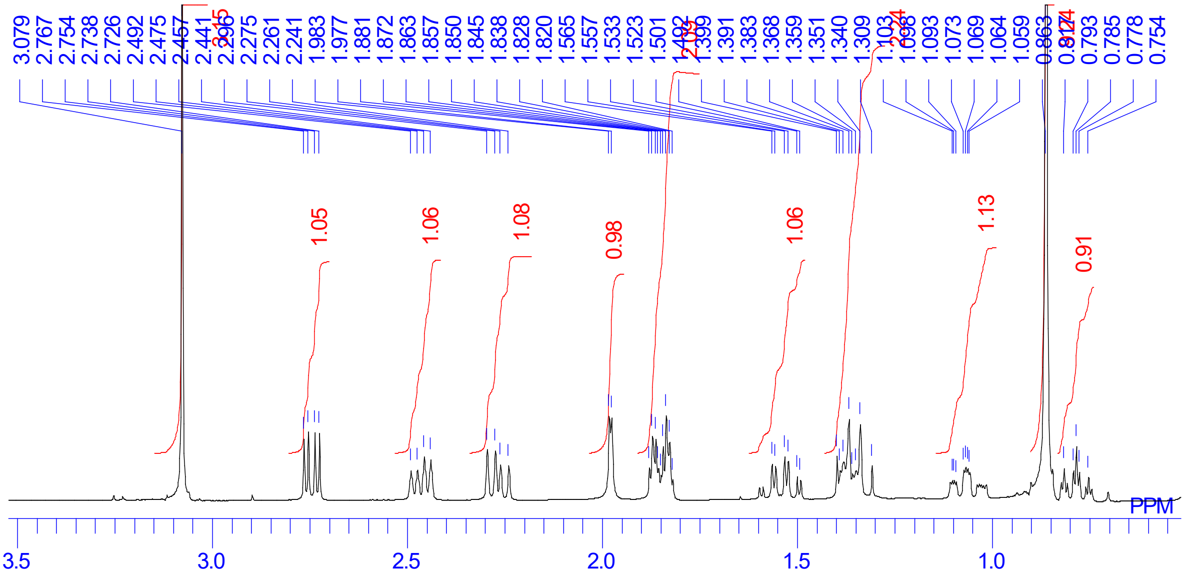

$^{13}C\{^1H\}$ NMR: (100 MHz, benzene-d_6)

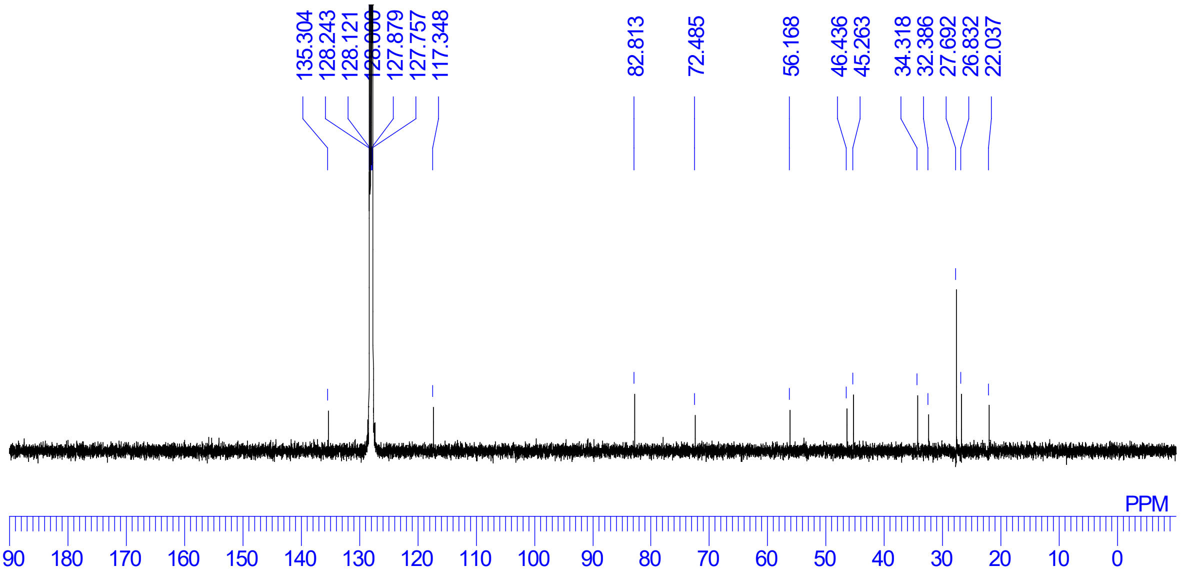

(1*S,2*R**,4*S**)-1-allyl-4-(*tert*-butyl)-2-methoxycyclohexan-1-ol *trans*-3p**

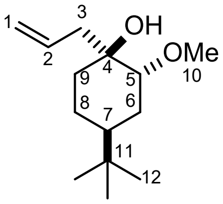

In a nitrogen-filled glove box, to a mixture of $BF_3{\cdot}Et_2O$ (14.2 mg, 0.1 mmol) and (2*R**,4*S**)-4-(*tert*-butyl)-2-methoxycyclohexan-1-one **2p** (18.4 mg, 0.1 mmol) in dichloromethane (1 mL) was added **1**Si(allyl) (35.4 mg, 0.1 mmol). After the reaction mixture was stirred for 3 h at room temperature, methanol (1 mL) was added to the mixture. The residue was evaporated to give a crude mixture, which was analyzed by ^{1}H NMR to obtain the yield and diastereoselectivity using 1,1,2,2-tetrachloroethane as an internal standard (NMR yield: 97%, *cis*/*trans* = 1/>99). The obtained residue was purified by a recycle GPC to give product as a colorless oil (20.8 mg, 92%). The compound was previously reported.[14]

^{1}H NMR (400 MHz, $CDCl_3$) 5.93–5.82 (m, 1H), 5.20–5.13 (m, 2H), 3.30 (s, 3H), 3.10 (brs, 1H), 2.42 (dd, *J* = 13.4, 7.4 Hz, 1H), 2.17 (dd, *J* = 13.8, 7.8 Hz, 1H), 1.91 (dd, *J* = 13.8, 3.0 Hz, 1H), 1.63–1.49 (m, 3H), 1.44–1.26 (m, 3H), 0.86 (s, 9H); ^{13}C{^{1}H} NMR (100 MHz, $CDCl_3$) 133.7 (d), 119.3 (t), 81.2 (d), 71.6 (s), 56.2 (q), 43.7 (t), 40.2 (d), 33.5 (t), 32.0 (s), 27.4 (q), 24.1 (t), 21.9 (t).

^{1}H NMR: (400 MHz, $CDCl_3$)

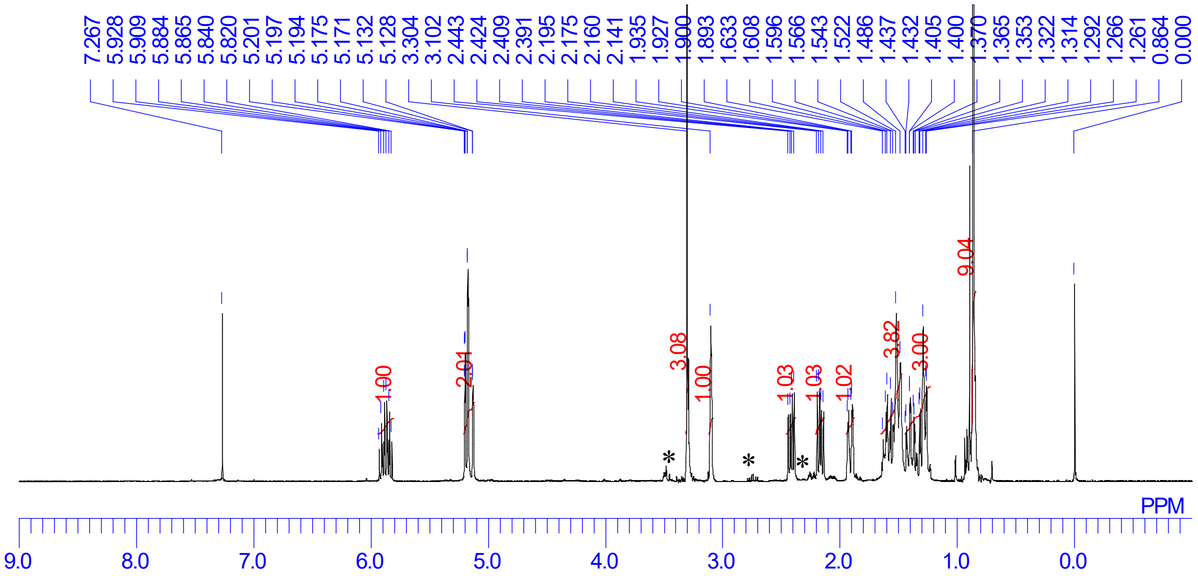

Asterisks represent inseparable impurities and residual solvents.

$^{13}C\{^1H\}$ NMR: (100 MHz, $CDCl_3$)

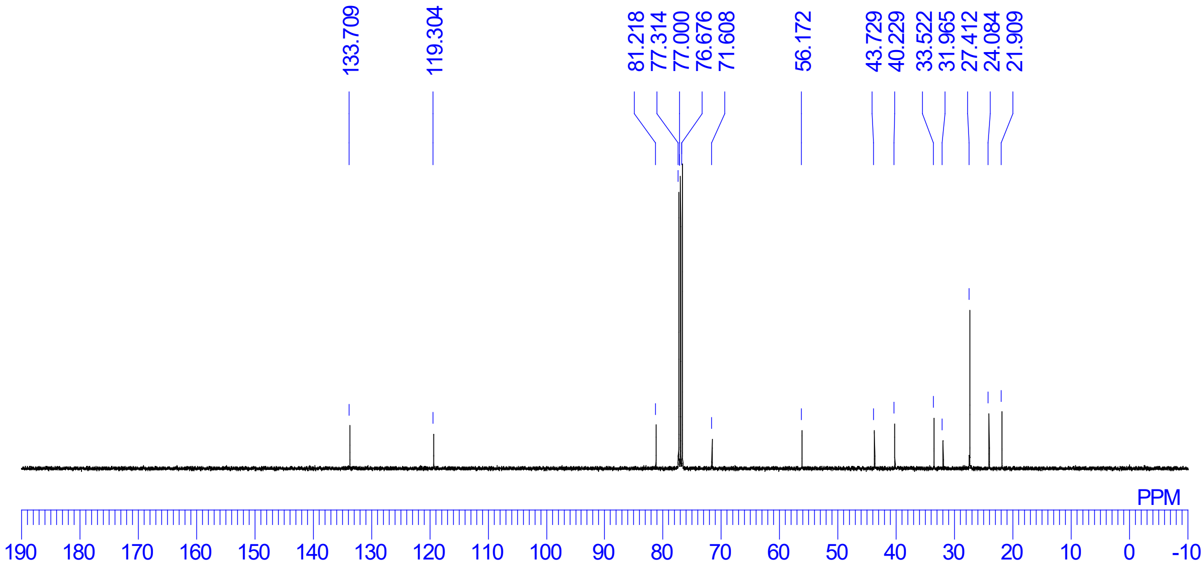

(1*S,2*S**)-1-Allyl-2-(benzyloxy)cyclobutan-1-ol *cis*-3q**

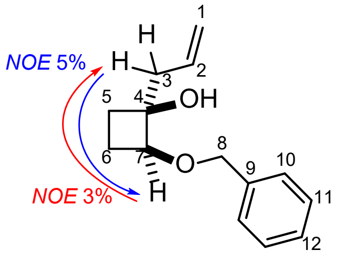

To a solution of 2-(benzyloxy)cyclobutan-1-one **2q** (17.6 mg, 0.1 mmol) in THF (1 mL) was added allylmagnesium bromide in Et_2O (0.7 M, 0.3 mL, 0.2 mmol) at 0 °C. After the reaction mixture was stirred for 3 h to room temperature, methanol (1 mL) was added to the mixture. The residue was evaporated to give a crude mixture, which was analyzed by ^1H NMR to obtain the yield and diastereoselectivity using 1,1,1,2-tetrachloroethane as an internal standard (NMR yield: 82%, *cis*/*trans* = 66/34). The obtained residue was purified by column chromatography (hexane/ethyl acetate = 50/50) on silica gel to give product as a colorless oil (10.8 mg, 50%).

IR (neat) v = 3551 (br), 3070 (m), 2980 (s), 2943 (s), 1496 (w), 1454 (s), 1433 (m), 1333 (m), 1208 (m), 1123 (s), 998 (s), 961 (w), 914 (s), 738 (m) cm^{-1}; ^1H NMR (400 MHz, $CDCl_3$) 7.38–7.28 (m, 5H), 5.89 (ddt, J = 17.2, 10.4, 7.8 Hz, 1H), 5.13–5.08 (m, 2H), 4.54 (d, J = 12.4 Hz, 1H), 4.51 (d, J = 12.4 Hz, 1H), 3.84 (t, J = 5.8 Hz, 1H), 3.07 (s, 1H), 2.32 (ddd, J = 12.5, 12.5, 6.9 Hz, 1H), 2.29 (ddd, J = 12.2, 12.2, 7.2 Hz, 1H), 2.07–1.96 (m, 2H), 1.94–1.87 (m, 1H), 1.79–1.72 (m, 1H); $^{13}C\{^1H\}$ NMR (100 MHz, $CDCl_3$) 137.9 (s), 133.5 (d), 128.4 (d), 127.80 (d), 127.75 (d), 117.9 (t), 77.3 (d), 76.8 (s), 71.1 (t), 44.0 (t), 29.6 (t), 23.6 (t); HRMS (DART$^+$) Calculated ($C_{14}H_{19}O_2$): 219.1380 ($[M+H]^+$), Found: 219.1384.

^{1}H NMR: (400 MHz, $CDCl_3$)

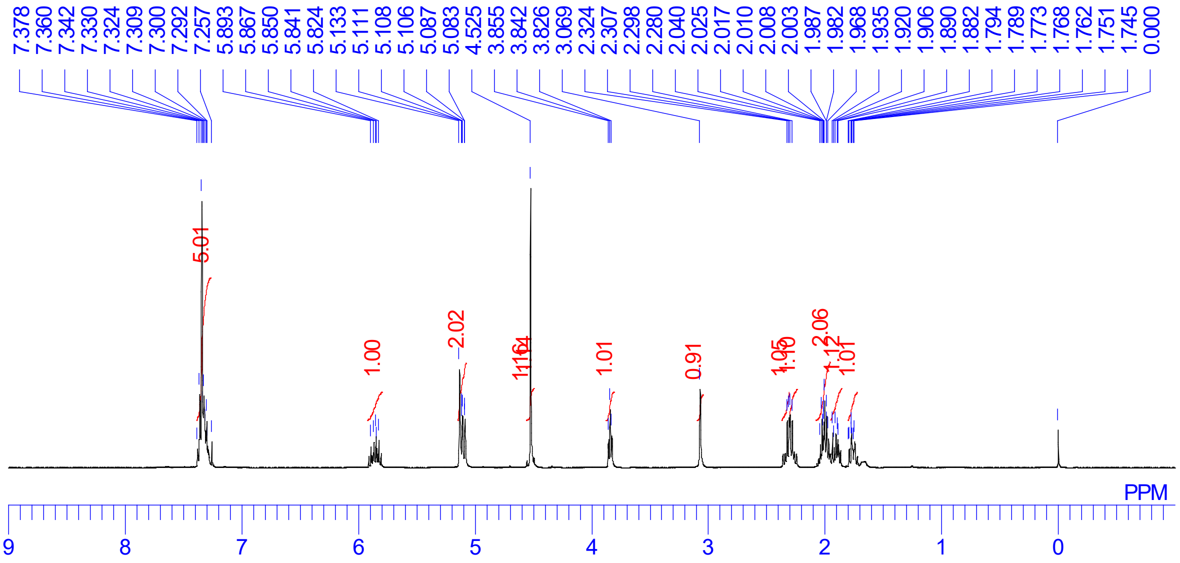

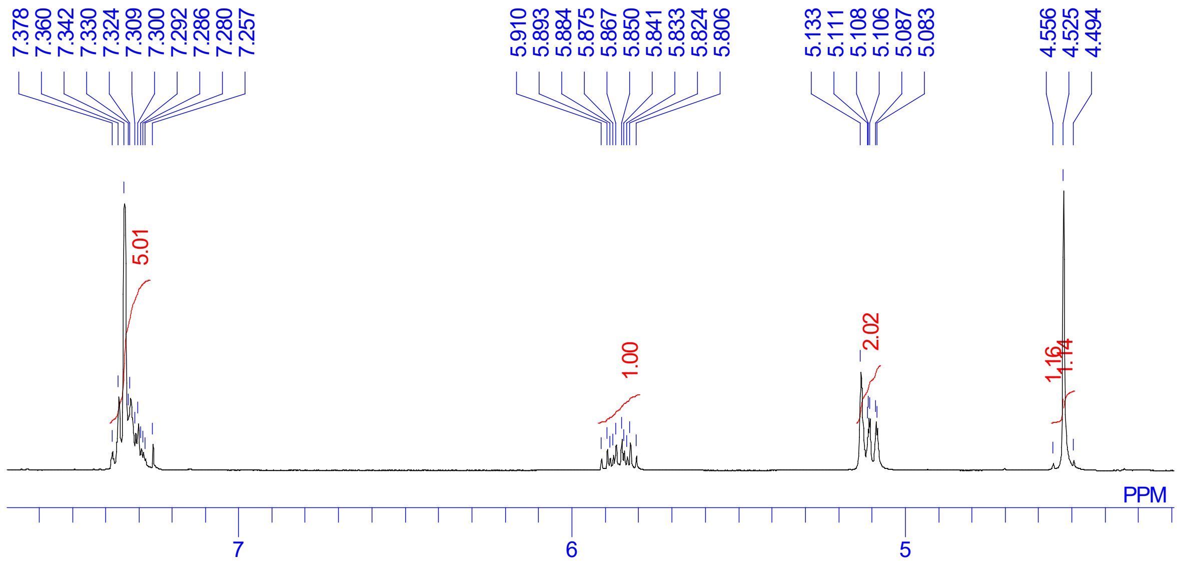

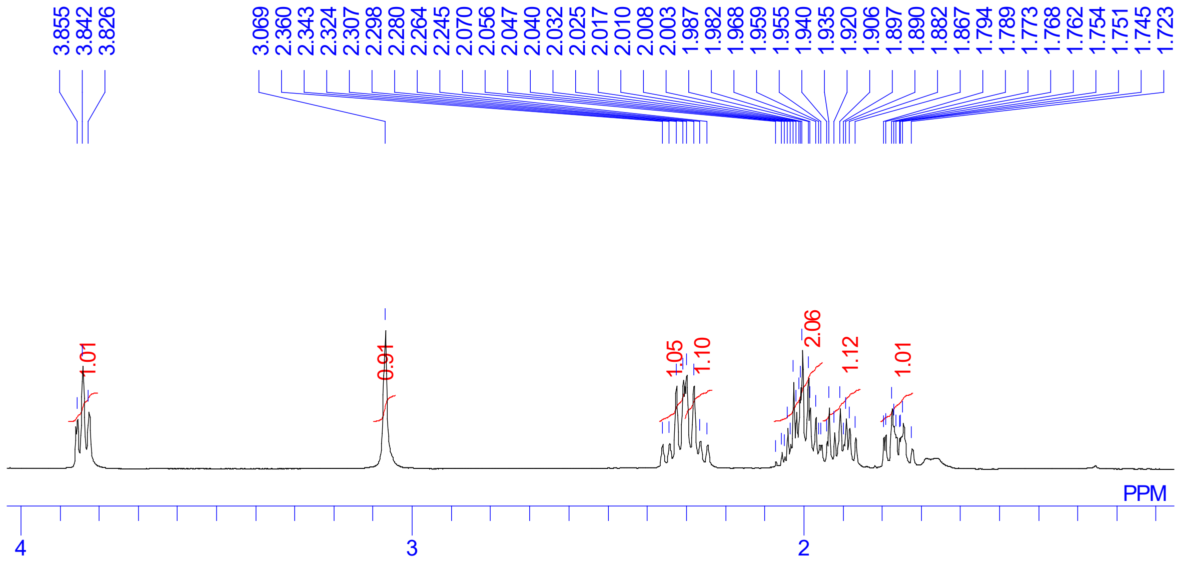

$^{13}C\{^{1}H\}$ NMR: (100 MHz, $CDCl_3$)

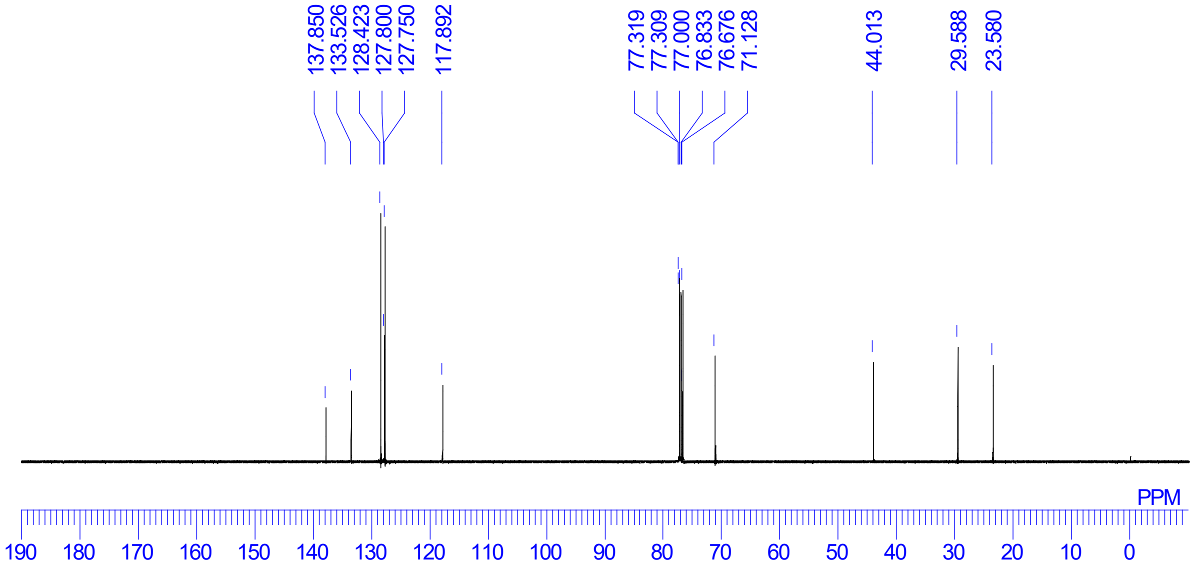

NOE differential spectrum (^{1}H NMR, 400 MHz, $CDCl_3$) irradiated at 3-H (2.30 ppm)

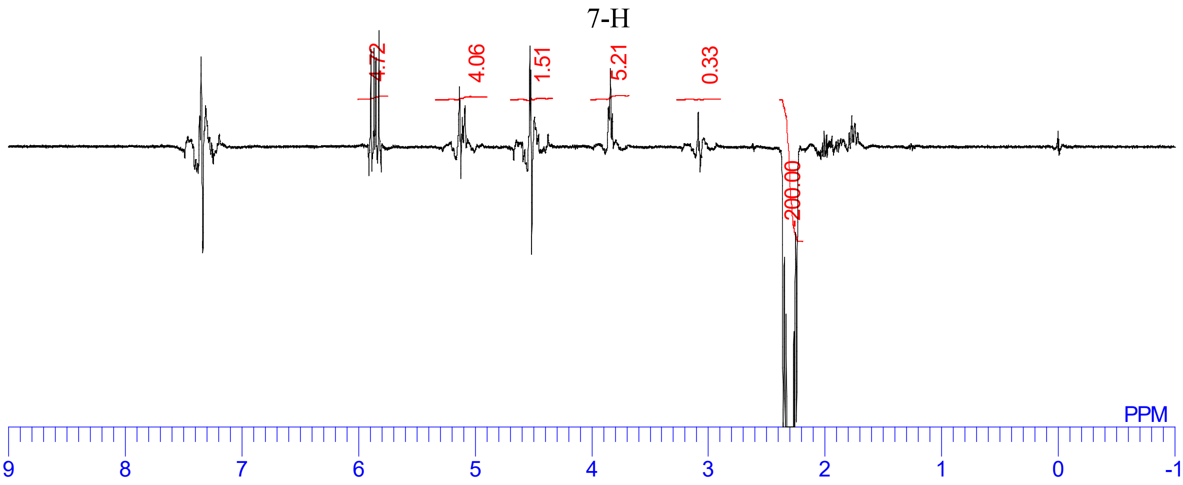

NOE differential spectrum (^{1}H NMR, 400 MHz, $CDCl_3$) irradiated at 7-H (3.84 ppm)

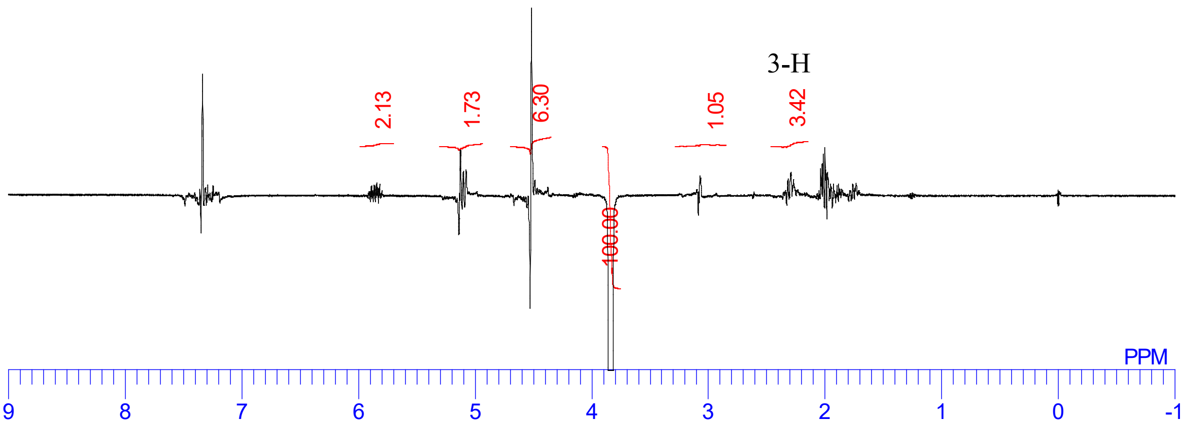

(1*R,2*S**)-1-Allyl-2-(benzyloxy)cyclobutan-1-ol *trans*-3q**

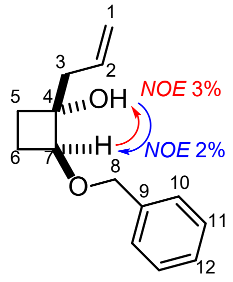

In a nitrogen-filled glovebox, to a mixture of $BF_3 \cdot Et_2O$ (28.3 mg, 0.1 mmol) and 2-(benzyloxy)cyclobutan-1-one **2q** (17.6 mg, 0.1 mmol) in dichloromethane (1 mL) was added **1**Si(allyl) (53.0 mg, 0.15 mmol). After the reaction

mixture was stirred for 3 h at room temperature, methanol (1 mL) was added to the mixture. The residue was evaporated to give a crude mixture, which was analyzed by ^{1}H NMR to obtain the yield and diastereoselectivity using 1,1,1,2-tetrachloroethane as an internal standard (NMR yield: 98%, *cis*/*trans* = 5/95). The obtained residue was purified by column chromatography (hexane/ethyl acetate = 50/50) on silica gel to give product as a colorless oil (18.6 mg, 85%).

IR (neat) ν = 3358 (br), 3069 (w), 2948 (m), 2868 (m), 1496 (w), 1454 (m), 1350 (m), 1282 (m), 1210 (m), 1123 (s), 999 (s), 915 (m), 736 (m) cm^{-1}; ^{1}H NMR (400 MHz, $CDCl_3$) 7.36–7.27 (m, 5H), 5.98 (ddt, J = 16.8, 10.8, 7.6 Hz, 1H, 2-H), 5.24–5.19 (m, 2H, 1-H), 4.55 (d, J = 11.6 Hz, 1H, 8-H), 4.52 (d, J = 12.0 Hz, 1H, 8-H), 3.99 (t, J = 8.2 Hz, 1H, 7-H), 2.49 (ddd, J = 10.6, 10.6, 7.1 Hz, 1H, 3-H), 2.46 (ddd, J = 10.7, 10.7, 7.1 Hz, 1H, 3-H), 2.17–2.05 (brs, 1H, OH), 2.07–1.97 (m, 1H, 6-H), 1.91–1.84 (m, 1H, 5-H), 1.55–1.43 (m, 2H, 5-H, 6-H); ^{13}C{^{1}H} NMR (100 MHz, $CDCl_3$) 138.3 (s, C-9), 133.4 (d, C-2), 128.3 (d, C-11), 127.59 (d, C-10), 127.57 (d, C-12), 119.6 (t, C-1), 81.5 (d, C-7), 77.9 (s, C-4), 71.4 (t, C-8), 38.1 (t, C-3), 26.5 (t, C-5), 20.8 (t, C-6); HRMS (DART^{+}) Calculated ($C_{14}H_{19}O_2$): 219.1380 ([M+H]$^{+}$), Found: 219.1376.

^{1}H NMR: (400 MHz, $CDCl_3$)

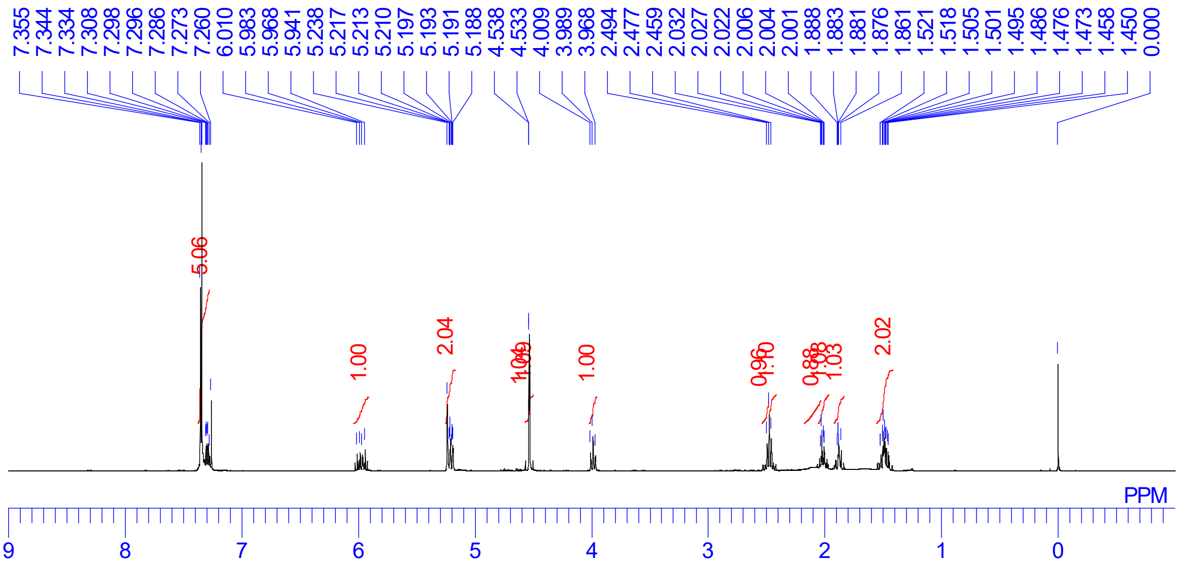

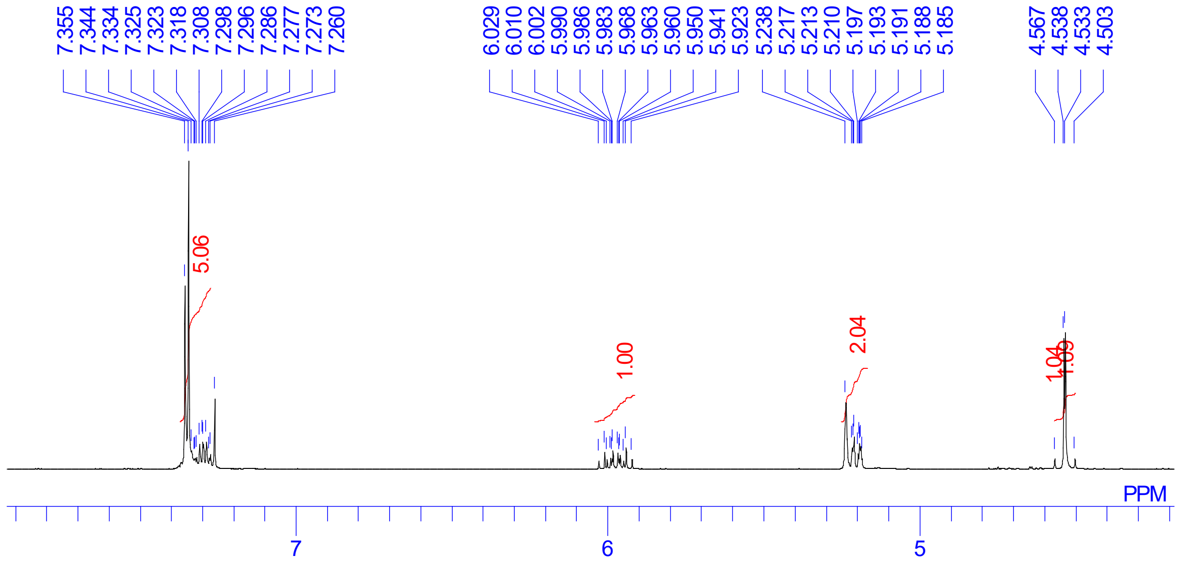
7.355
7.344
7.334
7.325
7.323
7.318
7.308
7.298
7.296
7.286
7.277
7.273
7.260
6.029
6.010
6.002
5.990
5.986
5.983
5.968
5.963
5.960
5.950
5.941
5.923
5.238
5.217
5.213
5.210
5.197
5.193
5.191
5.188
5.185
4.567
4.538
4.533
4.503
5.06
1.00
2.04
1.04
1.00
PPM
7
6
5

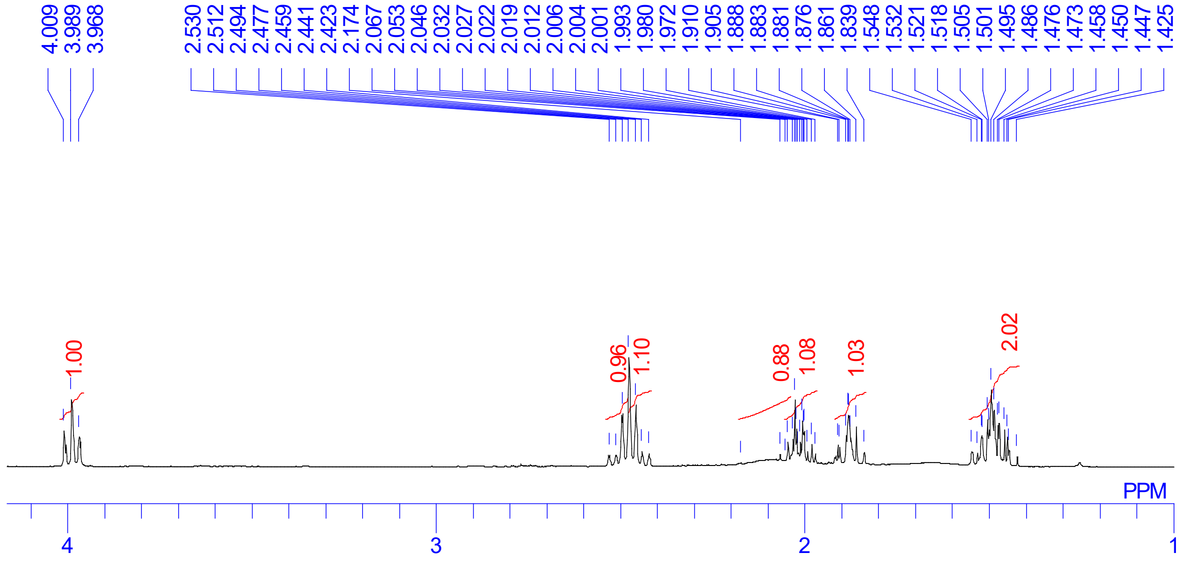
4.009
3.989
3.968
2.530
2.512
2.494
2.477
2.459
2.441
2.423
2.174
2.067
2.053
2.046
2.032
2.027
2.022
2.019
2.012
2.006
2.004
2.001
1.993
1.980
1.972
1.910
1.905
1.888
1.883
1.881
1.876
1.861
1.839
1.548
1.532
1.521
1.518
1.505
1.501
1.495
1.486
1.476
1.473
1.458
1.450
1.447
1.425
1.00
0.96
1.10
0.88
1.08
1.03
2.02
PPM
4
3
2
1

$^{13}C\{^{1}H\}$ NMR: (100 MHz, $CDCl_3$)

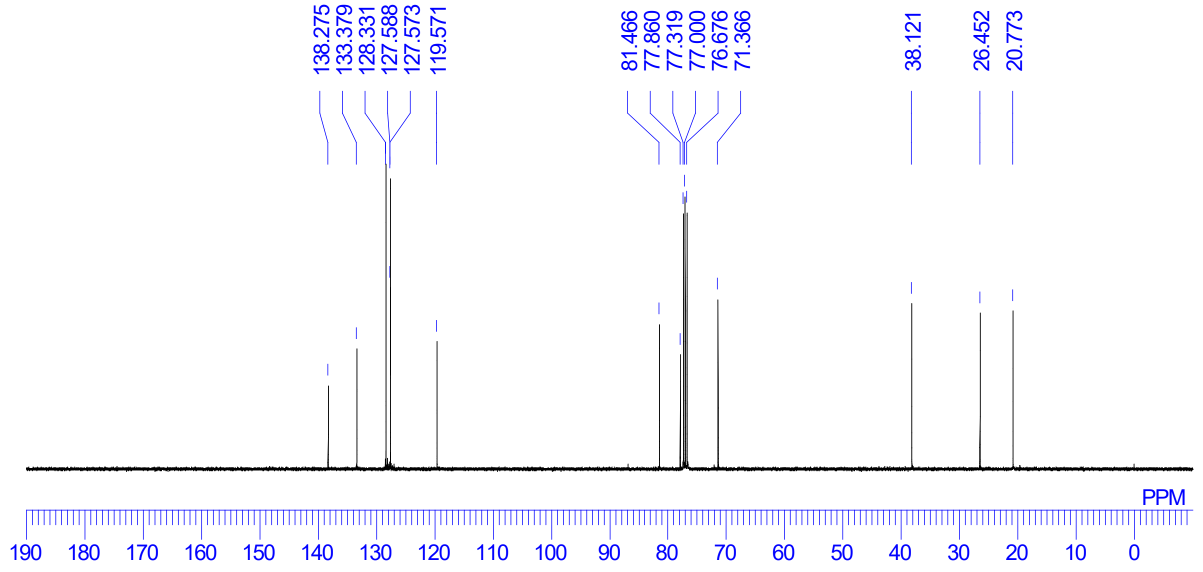

NOE differential spectrum (^{1}H NMR, 400 MHz, $CDCl_3$) irradiated at OH (2.12 ppm)

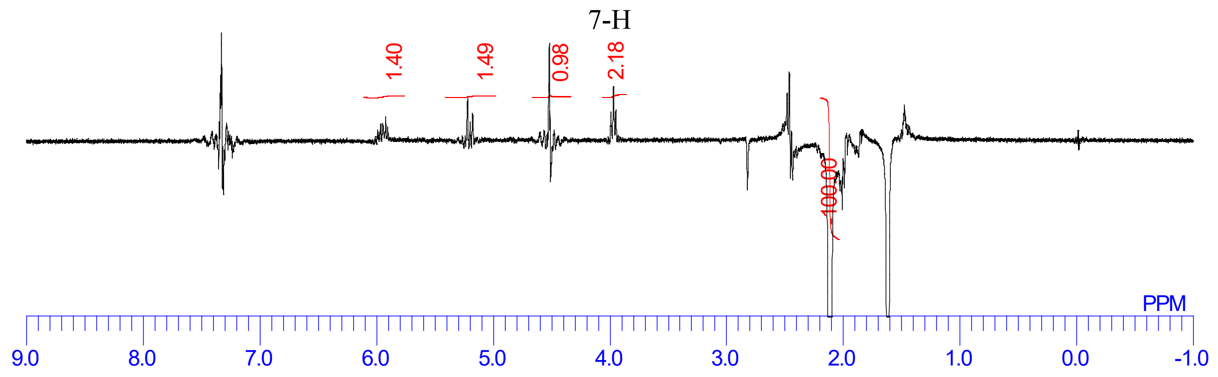

NOE differential spectrum (^{1}H NMR, 400 MHz, $CDCl_3$) irradiated at 7-H (3.98 ppm)

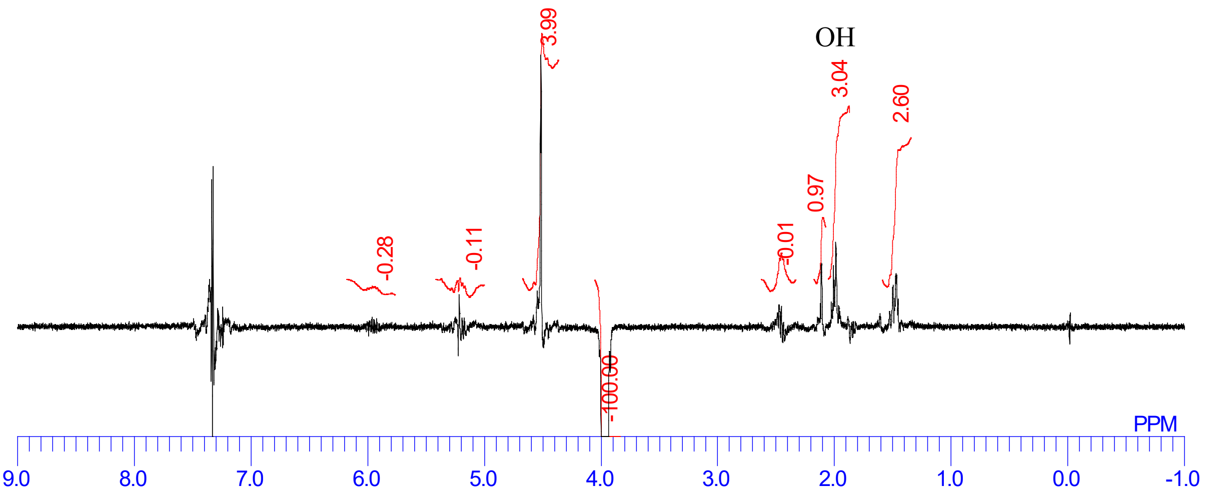

(1*S,2*R**)-1-Methoxy-4-methyl-1,2-diphenylpent-4-en-2-ol *syn*-3aa**

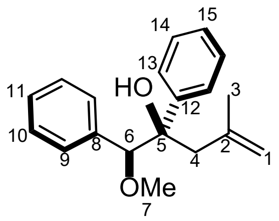

To a mixture of $SnCl_2$ (113 mg, 0.5 mmol) and benzoin methyl ether **2a** (113 mg, 0.5 mmol) in acetonitrile (2 mL) was added tributylmetallylstannane (173 mg, 0.5 mmol). After the reaction mixture was stirred for 14 h at room temperature, methanol (2 mL) was added to the mixture. The residue was evaporated to give a crude mixture, which was analyzed by ^{1}H NMR to obtain the yield and diastereoselectivity using 1,1,2,2-tetrachloroethane as an internal standard (NMR yield: 73%, *syn*/*anti* = 97/3). The obtained residue was purified by a recycle GPC to give the product as a colorless oil (96 mg, 68%).

IR (neat) v = 3537 (br), 3063 (m), 2932 (s), 2824 (m), 1643 (m), 1602 (w), 1493 (m), 1448 (s), 1375 (m), 1200 (s), 1157 (w), 1094 (s), 1030 (m), 948 (m), 886 (m), 747 (m) cm^{-1}; ^{1}H NMR (400 MHz, $CDCl_3$) 7.17–7.12 (m, 8H), 6.98 (dd, J = 7.4, 1.4 Hz, 2H), 4.75 (d, J = 1.2 Hz, 1H), 4.67 (d, J = 1.6 Hz, 1H), 4.28 (s, 1H), 3.27 (s, 3H), 3.00 (d, J = 14.0 Hz, 2H), 2.84–2.81 (m, 2H), 1.41 (s, 3H); $^{13}C\{^1H\}$ NMR (100 MHz, $CDCl_3$) 142.3 (s), 142.2 (s), 136.7 (s), 128.6 (d), 127.4 (d), 127.3 (d), 127.2 (d), 126.4 (d), 126.3 (d), 115.2 (t), 90.0 (d), 78.0 (s), 57.4 (q), 45.9 (t), 24.3 (q); HRMS (MALDI-TOF MS) Calculated ($C_{19}H_{22}O_2Na$): 305.1512 ($[M+Na]^+$), Found: 305.1517.

^{1}H NMR: (400 MHz, $CDCl_3$)

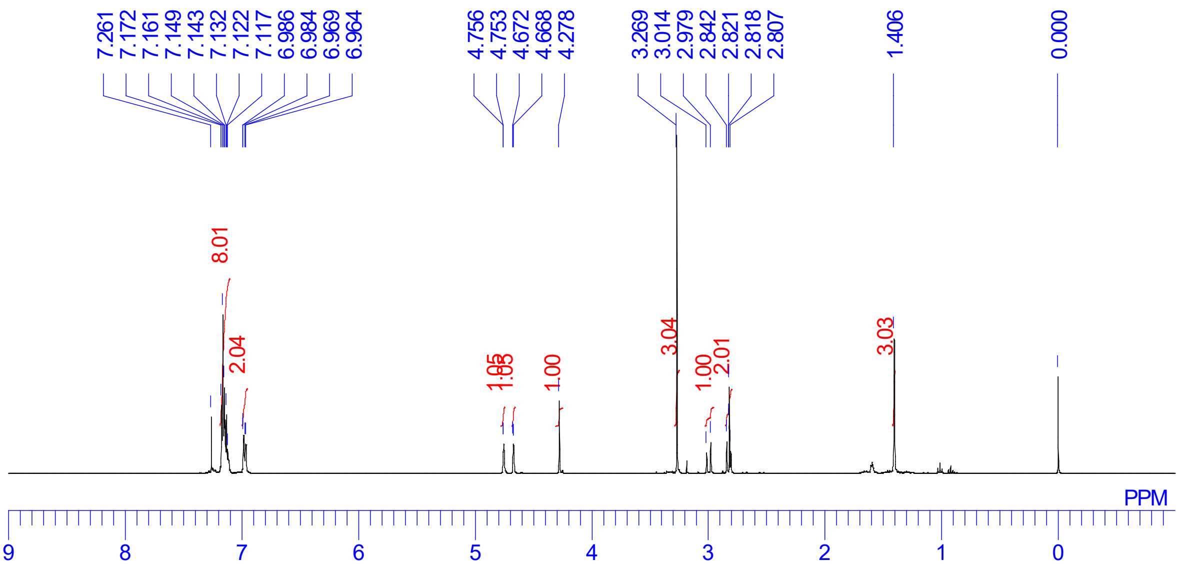

$^{13}C\{^{1}H\}$ NMR: (100 MHz, $CDCl_3$)

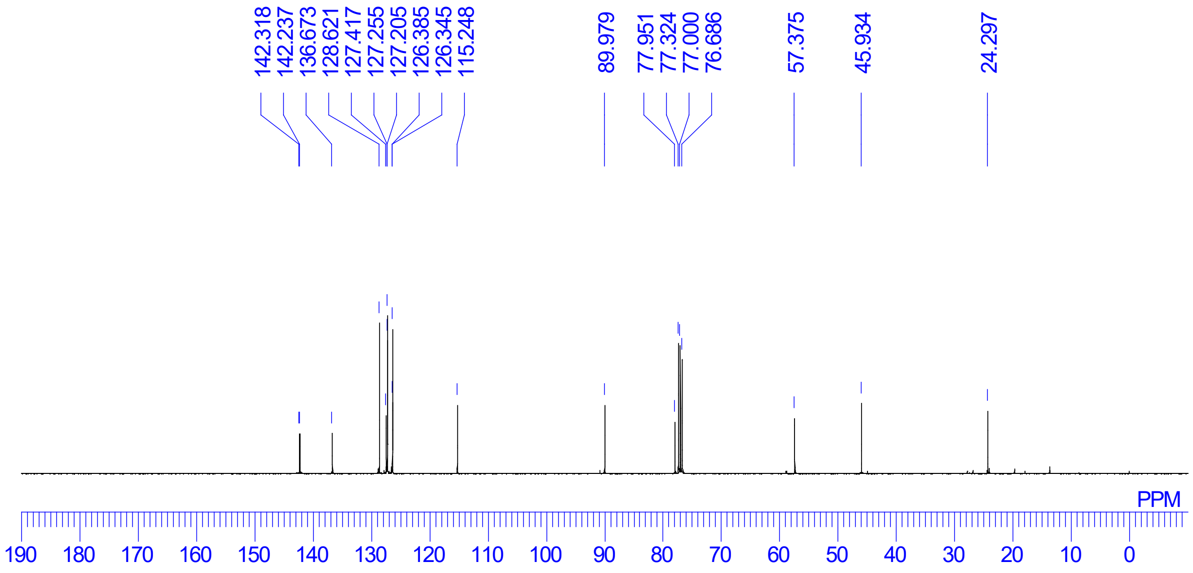

(1*S,2*S**)-1-Methoxy-4-methyl-1,2-diphenylpent-4-en-2-ol *anti*-3aa**

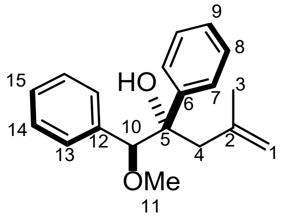

In a nitrogen-filled glovebox, to a mixture of $BF_3 \cdot Et_2O$ (14.2 mg, 0.1 mmol) and benzoin methyl ether **2a** (22.6 mg, 0.1 mmol) in dichloromethane (1 mL) was added **1**Si(metallyl) (36.8 mg, 0.1 mmol) at 0 °C. After the reaction mixture was stirred for 6 h at 0 °C, methanol (1 mL) was added to the mixture. The residue was evaporated to give

a crude mixture, which was analyzed by ^{1}H NMR to obtain the yield and diastereoselectivity using 1,1,2,2-tetrachloroethane as an internal standard (NMR yield: 78%, *syn*/*anti* = 7/93). The obtained residue was purified by column chromatography (hexane/ethyl acetate = 90/10) on silica gel to give product as a colorless oil (19.1 mg, 68%).

IR (neat) v = 3541 (br), 3061 (m), 2926 (s), 2825 (m), 1726 (m), 1643 (w), 1494 (m), 1446 (s), 1264 (m), 1096 (s), 892 (m), 836 (w), 701 (s) cm^{-1}; ^{1}H NMR (400 MHz, $CDCl_3$) 7.29 (dd, J = 8.2, 1.8 Hz, 2H), 7.25–7.21 (m, 6H), 7.03 (dd, J = 8.0, 2.0 Hz, 2H, 13-H), 4.74 (dd, J = 2.8, 1.2 Hz, 1H, 1-H), 4.60 (d, J = 1.6 Hz, 1H, 1-H), 4.25 (s, 1H, 10-H), 3.19 (s, 3H, 11-H), 2.87 (s, 1H, OH), 2.69 (d, J = 14.0 Hz, 1H, 4-H), 2.54 (d, J = 14.4 Hz, 1H, 4-H), 1.42 (s, 3H, 3-H); $^{13}C\{^1H\}$ NMR (100 MHz, $CDCl_3$) 142.9 (s, C-6), 142.1 (s, C-2), 136.9 (s, C-12), 128.9 (d, C-13), 127.8 (d), 127.5 (d), 127.3 (d), 126.7 (d), 126.6 (d), 115.3 (t, C-1), 90.9 (d, C-10), 77.8 (s, C-5), 57.3 (q, C-11), 45.0 (t, C-4), 24.4 (q, C-3); HRMS (MALDI-TOF MS) Calculated ($C_{19}H_{22}O_2Na$): 305.1512 ([M+Na]$^+$), Found: 305.1508.

^{1}H NMR: (400 MHz, $CDCl_3$)

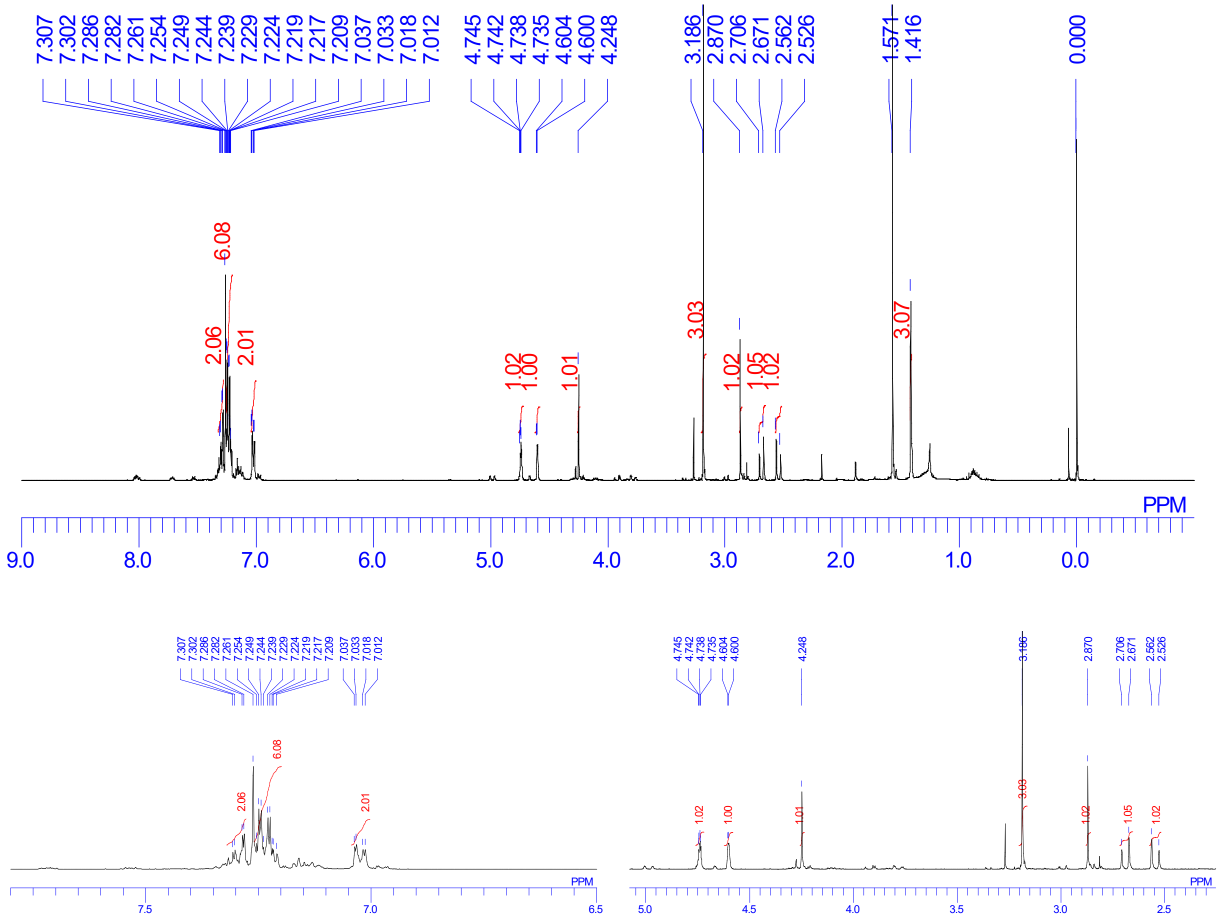

$^{13}C\{^{1}H\}$ NMR: (100 MHz, $CDCl_3$)

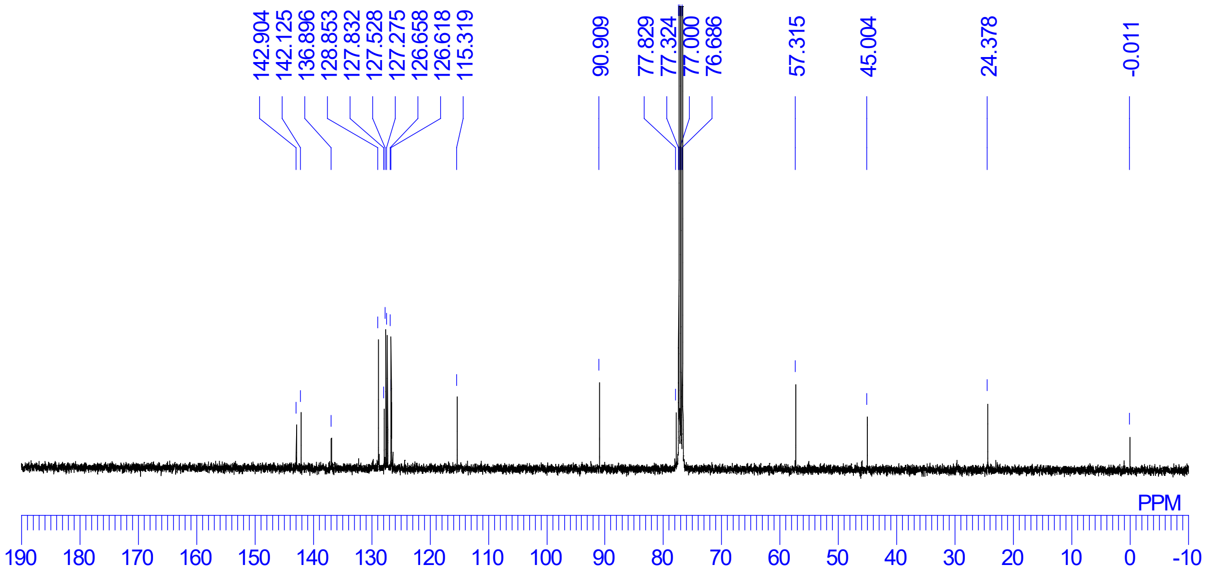

(1*S,2*R**)-1-Methoxy-1,2,4-triphenylpent-4-en-2-ol *syn*-3ab**

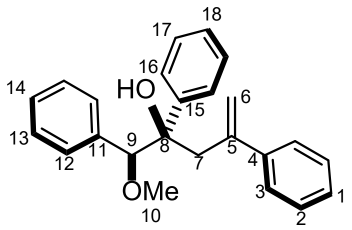

To a mixture of $SnCl_2$ (113 mg, 0.5 mmol) and benzoin methyl ether **2a** (113 mg, 0.5 mmol) in acetonitrile (2 mL) was added tributyl(2-phenyl-2-propen-1-yl)stannane (204 mg, 0.5 mmol). After the reaction mixture was stirred for 14 h at room temperature, methanol (5 mL) was added to the mixture. The residue was evaporated to give a crude mixture, which was analyzed by ^{1}H NMR to obtain the yield and diastereoselectivity using 1,1,2,2-tetrachloroethane as an internal standard (NMR yield: 62%, *syn*/*anti* = 98/2). The obtained residue was purified by column chromatography (hexane/ethyl acetate = 40:60) on silica gel. Further purification was conducted by a recycle GPC to give the product as a colorless oil (81 mg, 47%).

IR (neat) ν = 3564 (br), 3082 (w), 3059 (m), 2932 (m), 2824 (w), 1626 (w), 1494 (s), 1447 (s), 1316 (w), 1201 (m), 1098 (s), 1030 (w), 957 (w), 905 (m), 777 (m) cm^{-1}; ^{1}H NMR (400 MHz, $CDCl_3$) 7.21–7.17 (m, 5H), 7.13–7.04 (m, 8H), 6.97 (dd, J = 7.4, 1.8 Hz, 2H), 5.12 (d, J = 1.6 Hz, 1H), 4.92 (d, J = 1.2 Hz, 1H), 4.30 (s, 1H), 3.46 (dd, J = 14.8, 1.2 Hz, 1H), 3.25 (dd, J = 14.8, 1.2 Hz, 1H), 3.18 (s, 3H), 2.65 (s, 1H); $^{13}C\{^{1}H\}$ NMR (100 MHz, $CDCl_3$) 144.7 (s), 143.3 (s), 142.0 (s), 136.7 (s), 128.7 (d), 128.0 (d), 127.4 (d), 127.3 (d), 127.1 (d), 126.9 (d), 126.4 (d), 126.33 (d), 126.25 (d), 117.5 (t), 89.4 (d), 79.1 (s), 57.2 (q), 44.0 (t); HRMS (MALDI-TOF MS) Calculated ($C_{24}H_{24}NaO_2$): 367.1669 ($[M+Na]^+$), Found: 367.1661.

^{1}H NMR: (400 MHz, $CDCl_3$)

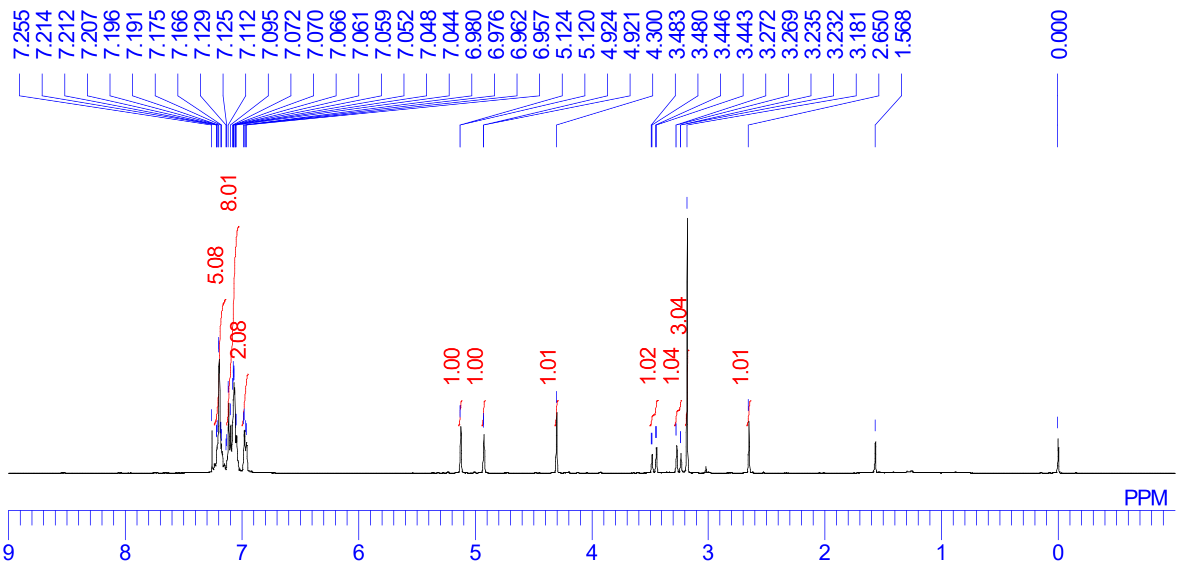

$^{13}C\{^1H\}$ NMR: (100 MHz, $CDCl_3$)

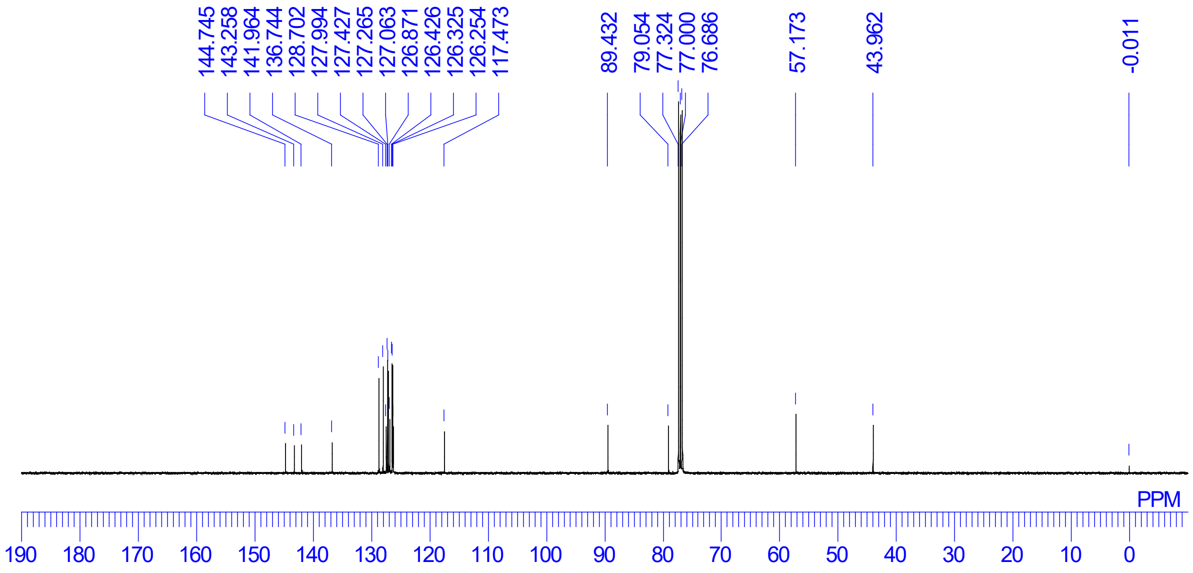

(1*S,2*S**)-1-Methoxy-1,2,4-triphenylpent-4-en-2-ol *anti*-3ab**

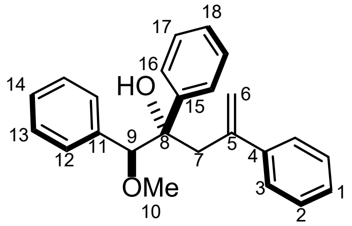

In a nitrogen-filled glovebox, to a mixture of $BF_3 \cdot Et_2O$ (14.2 mg, 0.1 mmol) and benzoin methyl ether **2a** (22.6 mg, 0.1 mmol) in dichloromethane (1 mL) was added **1**Si(2-phenylallyl) (43.0 mg, 0.1 mmol). After the reaction mixture was stirred for 6 h at room temperature, methanol (1 mL) was added to the mixture. The residue was evaporated to

give a crude mixture, which was analyzed by ^{1}H NMR to obtain the yield and diastereoselectivity using 1,1,2,2-tetrachloroethane as an internal standard (NMR yield: 69%, *syn*/*anti* = 1/99). The obtained residue was purified by column chromatography (hexane/ethyl acetate = 85/15) on silica gel. Further purification was conducted by a recycle GPC to give product as a colorless oil (22.3 mg, 65%).

IR (neat) v = 3545 (br), 3058 (s), 3028 (s), 2931 (s), 1725 (w), 1624 (m), 1600 (m), 1446 (s), 1354 (m), 1178 (m), 1104 (s), 1002 (w), 978 (m), 907 (m), 754 (m) cm^{-1}; ^{1}H NMR (400 MHz, $CDCl_3$) 7.25–7.15 (m, 13H), 6.92 (d, J = 6.8 Hz, 2H, 12-H), 5.23 (d, J = 1.6 Hz, 1H, 6-H), 5.00 (s, 1H, 6-H), 4.20 (s, 1H, 9-H), 3.30 (d, J = 14.8 Hz, 1H, 7-H), 3.02 (s, 3H, 10-H), 3.00 (d, J = 14.0 Hz, 1H, 7-H), 2.62 (s, 1H, OH); $^{13}C\{^{1}H\}$ NMR (100 MHz, $CDCl_3$) 144.5 (s, C-4), 143.1 (s, C-5), 142.5 (s, C-15), 136.8 (s, C-11), 128.8 (d, C-12), 128.0 (d), 127.7 (d), 127.4 (d), 127.1 (d), 127.0 (d), 126.7 (d), 126.55 (d), 126.47 (d), 117.9 (t, C-6), 89.6 (d, C-9), 78.3 (s, C-8), 56.9 (q, C-10), 42.3 (t, C-7); HRMS (MALDI-TOF MS) Calculated ($C_{24}H_{24}NaO_2$): 367.1669 ([M+Na]$^{+}$), Found: 367.1664.

^{1}H NMR: (400 MHz, $CDCl_3$)

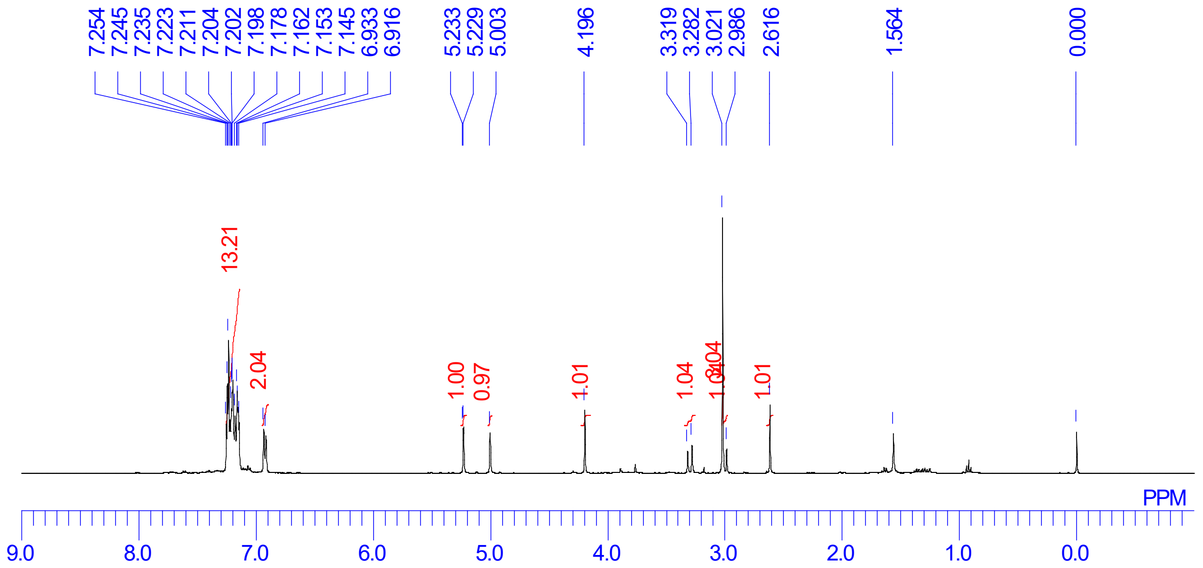

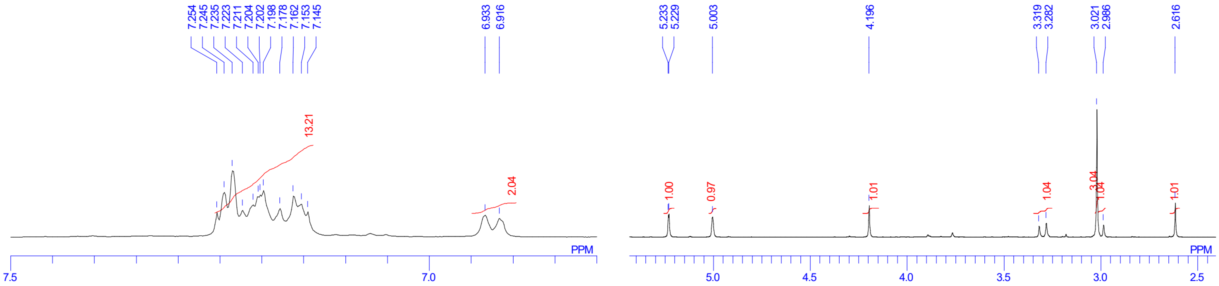

$^{13}C\{^1H\}$ NMR: (100 MHz, $CDCl_3$)

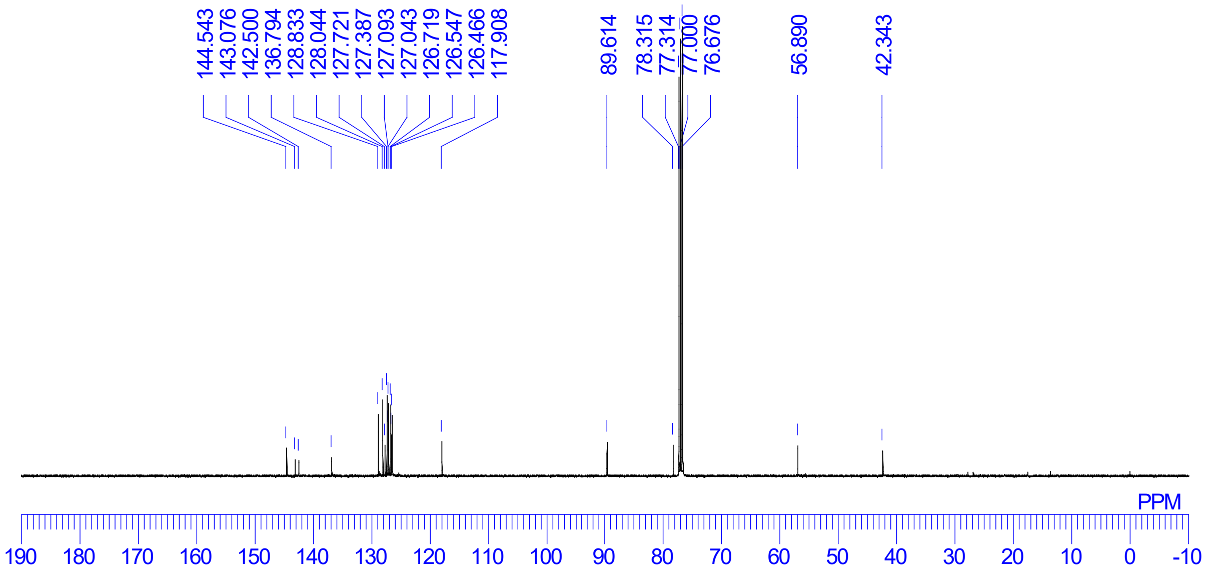

5. X-ray crystallographic data

5-1. Allylsilatrane 1Si(allyl)

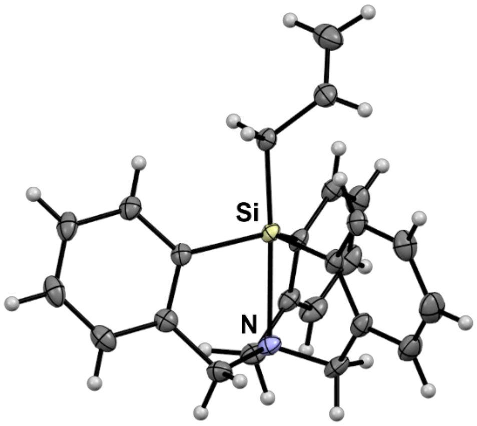

Figure S1. ORTEP drawings of **1**Si(allyl) at the 50% probability level.

No. CCDC	2443722	Space Group	$P2_1/c$ (#14)
Empirical Formula	$C_{24}H_{23}NSi$	Z value	4
Formula Weight	353.52	D_{calc}	1.255 g/cm^3
Crystal Color	colorless	F_{000}	752.0
Crystal Dimensions	0.25 X 0.18 X 0.13 mm	μ(CuK_α)	1.136 mm^{-1}
Crystal System	monoclinic	Temperature	123 K
Lattice Type	Primitive	Data/restraints/parameters	3733/0/235
Lattice Parameters	a = 12.3584(4) Å	Residuals: R_1 ($I > 2.00\sigma(I)$)	0.0453
	b = 13.5426(3) Å	Residuals: wR_2 (all data)	0.1251
	c = 12.5163(4) Å	Goodness of Fit Indicator	1.075
	β = 116.714(4) °	Crystal growth	Slow evaporation of a acetone/hexane solution
	V = 1871.19(11) Å^3		

```
Alert level C
PLAT230_ALERT_2_C Hirshfeld Test Diff for    Si1      --C22      .        6.6 s.u.
PLAT911_ALERT_3_C Missing FCF Refl Between Thmin & STh/L=    0.600          5 Report
              -13  5  1,   6  7  5,   6 10  6, -10 10 10,  -6  9 12,

Alert level G
PLAT912_ALERT_4_G Missing # of FCF Reflections Above STh/L=  0.600        111 Note
PLAT941_ALERT_3_G Average HKL Measurement Multiplicity ..........         3.2 Low
PLAT969_ALERT_5_G The 'Henn et al.' R-Factor-gap value ..........        2.09 Note
              Predicted wR2: Based on SigI**2  5.99 or SHELX Weight 12.03
PLAT978_ALERT_2_G Number C-C Bonds with Positive Residual Density.         11 Info

   0 ALERT level A = Most likely a serious problem - resolve or explain
   0 ALERT level B = A potentially serious problem, consider carefully
   2 ALERT level C = Check. Ensure it is not caused by an omission or oversight
   4 ALERT level G = General information/check it is not something unexpected

   0 ALERT type 1 CIF construction/syntax error, inconsistent or missing data
   2 ALERT type 2 Indicator that the structure model may be wrong or deficient
   2 ALERT type 3 Indicator that the structure quality may be low
   1 ALERT type 4 Improvement, methodology, query or suggestion
   1 ALERT type 5 Informative message, check
```

5-2. Allylgermatrane 1Ge(allyl)

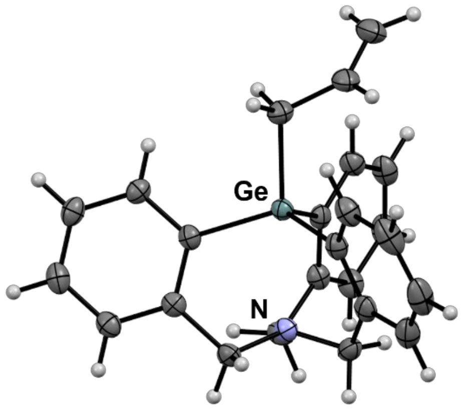

Figure S2. ORTEP drawings of **1**Ge(allyl) at the 50% probability level.

No. CCDC	2443723	Space Group	$P2_1$/n (#14)
Empirical Formula	$C_{24}H_{23}NGe$	*Z* value	8
Formula Weight	398.02	D_{calc}	1.362 g/cm^3
Crystal Color	colorless	F_{000}	1648.0
Crystal Dimensions	0.12 X 0.09 X 0.05 mm	μ(CuK_α)	2.171 mm^{-1}
Crystal System	monoclinic	Temperature	123 K
Lattice Type	Primitive	Data/restraints/parameters	7779/0/469
Lattice Parameters	a = 11.33740(10) Å	Residuals: R_1 ($I > 2.00\sigma(I)$)	0.0320
	b = 13.5784(2) Å	Residuals: wR_2 (all data)	0.0859
	c = 25.4964(3) Å	Goodness of Fit Indicator	1.034
	β = 98.4140(10)°	Crystal growth	Slow evaporation of a ether/hexane solution
	V = 3882.76(8) Å^3		

The following ALERTS were generated. Each ALERT has the format
test-name_ALERT_alert-type_alert-level.
Click on the hyperlinks for more details of the test.

Alert level C

PLAT911_ALERT_3_C Missing FCF Refl Between Thmin & STh/L= 0.600 6 Report
-12 5 13, -12 4 16, 2 13 17, 8 5 20, 2 1 29, 1 3 29,

Alert level G

PLAT720_ALERT_4_G Number of Unusual/Non-Standard Labels .... 8 Note
H8BA H8BB H1BA H1BB H1AA H1AB H8AA H8AB
PLAT912_ALERT_4_G Missing # of FCF Reflections Above STh/L= 0.600 222 Note
PLAT941_ALERT_3_G Average HKL Measurement Multiplicity .... 3.7 Low
PLAT969_ALERT_5_G The 'Henn et al.' R-Factor-gap value .... 1.81 Note
Predicted wR2: Based on SigI**2 4.74 or SHELX Weight 8.57
PLAT978_ALERT_2_G Number C-C Bonds with Positive Residual Density. 8 Info

---

0 **ALERT level A** = Most likely a serious problem - resolve or explain
0 **ALERT level B** = A potentially serious problem, consider carefully
1 **ALERT level C** = Check. Ensure it is not caused by an omission or oversight
5 **ALERT level G** = General information/check it is not something unexpected

0 ALERT type 1 CIF construction/syntax error, inconsistent or missing data
1 ALERT type 2 Indicator that the structure model may be wrong or deficient
2 ALERT type 3 Indicator that the structure quality may be low
2 ALERT type 4 Improvement, methodology, query or suggestion
1 ALERT type 5 Informative message, check

5-3. Allylstannatrane 1Sn(allyl)

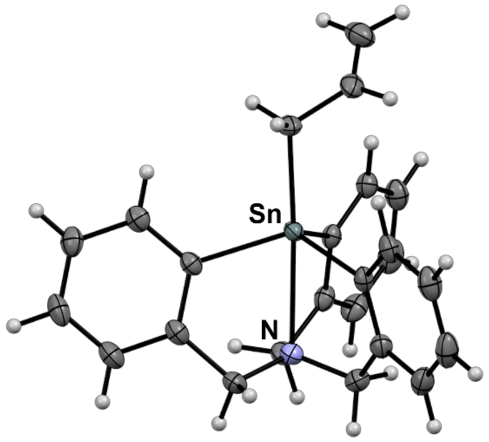

Figure S3. ORTEP drawings of **1**Sn(allyl) at the 50% probability level.

No. CCDC	2443724	Space Group	$P2_1/c$ (#14)
Empirical Formula	$C_{24}H_{23}NSn$	Z value	8
Formula Weight	444.12	D_{calc}	1.506 g/cm^3
Crystal Color	colorless	F_{000}	1792.0
Crystal Dimensions	0.19 X 0.11 X 0.07 mm	μ(CuK$_\alpha$)	10.404 mm^{-1}
Crystal System	monoclinic	Temperature	123 K
Lattice Type	Primitive	Data/restraints/parameters	7803/0/469
Lattice Parameters	a = 18.3443(3) Å	Residuals: R_1 ($I > 2.00\sigma(I)$)	0.0319
	b = 11.9752(2) Å	Residuals: wR_2 (all data)	0.0845
	c = 18.0701(3) Å	Goodness of Fit Indicator	1.050
	β = 99.3160(10)°	Crystal growth	Slow evaporation of a ether/hexane solution
	V = 3917.22(11) Å^3		

```
Alert level C
PLAT230_ALERT_2_C Hirshfeld Test Diff for    C22A     --C23A     .        6.7 s.u.
PLAT368_ALERT_2_C Short  C(sp2)-C(sp2) Bond  C23A     - C24A     .       1.22 Ang.
PLAT911_ALERT_3_C Missing FCF Refl Between Thmin & STh/L=    0.600          3 Report
                0 13  9,    3  8 17,    5  6 18,
PLAT971_ALERT_2_C Check Calcd Resid. Dens.  1.24Ang From C23A           1.56 eA-3
PLAT977_ALERT_2_C Check Negative Difference Density on H24C      .      -0.37 eA-3

Alert level G
PLAT720_ALERT_4_G Number of Unusual/Non-Standard Labels ..........          8 Note
              H8AA    H8AB    H1BA    H1BB    H8BA    H8BB    H1AA    H1AB
PLAT910_ALERT_3_G Missing # of FCF Reflection(s) Below Theta(Min).          1 Note
                1  0  0,
PLAT912_ALERT_4_G Missing # of FCF Reflections Above STh/L=  0.600        273 Note
PLAT941_ALERT_3_G Average HKL Measurement Multiplicity ...........        3.4 Low
PLAT969_ALERT_5_G The 'Henn et al.' R-Factor-gap value ...........       1.62 Note
              Predicted wR2: Based on SigI**2  5.23 or SHELX Weight  8.30
PLAT978_ALERT_2_G Number C-C Bonds with Positive Residual Density.          3 Info

   0 ALERT level A = Most likely a serious problem - resolve or explain
   0 ALERT level B = A potentially serious problem, consider carefully
   5 ALERT level C = Check. Ensure it is not caused by an omission or oversight
   6 ALERT level G = General information/check it is not something unexpected

   0 ALERT type 1 CIF construction/syntax error, inconsistent or missing data
   5 ALERT type 2 Indicator that the structure model may be wrong or deficient
   3 ALERT type 3 Indicator that the structure quality may be low
   2 ALERT type 4 Improvement, methodology, query or suggestion
   1 ALERT type 5 Informative message, check
```

5-4. Methallylsilatrane 1Si(methallyl)

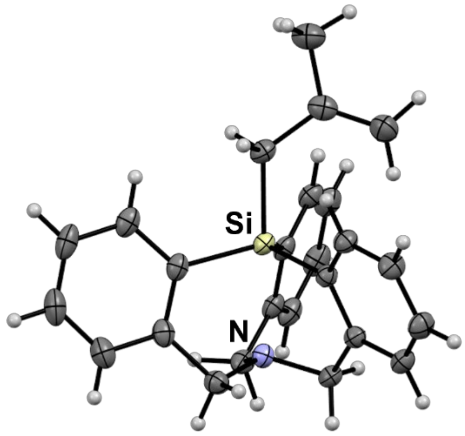

Figure S4. ORTEP drawings of **1**Si(methallyl) at the 50% probability level.

No. CCDC	2443725	Space Group	$P2_1$/n (#14)
Empirical Formula	$C_{25}H_{25}NSi$	Z value	8
Formula Weight	367.55	D_{calc}	1.242 g/cm^3
Crystal Color	colorless	F_{000}	1568.0
Crystal Dimensions	0.15 X 0.13 X 0.08 mm	μ(CuK_α)	1.101 mm^{-1}
Crystal System	monoclinic	Temperature	123 K
Lattice Type	Primitive	Data/restraints/parameters	7840/0/489
Lattice Parameters	a = 10.56040(10) Å	Residuals: R_1 ($I > 2.00\sigma(I)$)	0.0384
	b = 26.4754(3) Å	Residuals: wR_2 (all data)	0.1060
	c = 14.1395(2) Å	Goodness of Fit Indicator	1.034
	β = 96.1810(10)°	Crystal growth	Slow evaporation of a acetone/hexane solution
	V = 3930.29(8) Å^3		

Alert level C

```
PLAT230_ALERT_2_C Hirshfeld Test Diff for    Si1A     --C3A      .         6.0 s.u.
PLAT230_ALERT_2_C Hirshfeld Test Diff for    Si1A     --C17A     .         5.4 s.u.
PLAT230_ALERT_2_C Hirshfeld Test Diff for    Si1A     --C22A     .         5.6 s.u.
PLAT230_ALERT_2_C Hirshfeld Test Diff for    Si1B     --C3B      .         5.1 s.u.
PLAT911_ALERT_3_C Missing FCF Refl Between Thmin & STh/L=    0.600         10 Report
              -12 10  1,  -2 31  1, -11 11  3, -11 12  7,  10  8  8, -10 15  8,
                1 16 14,   2 16 14,   1 17 14,  -1 14 15,
```

Alert level G

```
PLAT720_ALERT_4_G Number of Unusual/Non-Standard Labels ..........          8 Note
              H8BA    H8BB    H1AA    H1AB    H1BA    H1BB    H8AA    H8AB
PLAT912_ALERT_4_G Missing # of FCF Reflections Above STh/L=  0.600        278 Note
PLAT941_ALERT_3_G Average HKL Measurement Multiplicity ...........        3.1 Low
PLAT969_ALERT_5_G The 'Henn et al.' R-Factor-gap value ...........       2.06 Note
              Predicted wR2: Based on SigI**2  5.15 or SHELX Weight 10.58
PLAT978_ALERT_2_G Number C-C Bonds with Positive Residual Density.         12 Info
```

5-5. 2-Phenylallylsilatrane 1Si(2-phenylallyl)

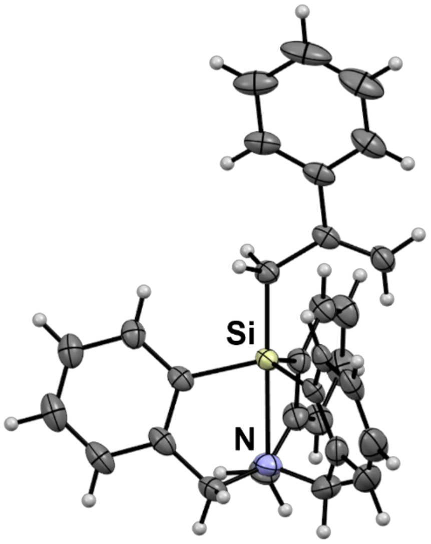

Figure S5. ORTEP drawings of **1**Si(2-phenylallyl) at the 50% probability level.

No. CCDC	2443726	Space Group	*P*1 (#1)
Empirical Formula	$C_{30}H_{27}NSi$	*Z* value	2
Formula Weight	429.61	D_{calc}	1.227 g/cm^3
Crystal Color	colorless	F_{000}	456.0
Crystal Dimensions	0.12 X 0.1 X 0.09 mm	μ(CuK$_\alpha$)	1.007 mm^{-1}
Crystal System	triclinic	Temperature	123 K
Lattice Type	Primitive	Data/restraints/parameters	5755/3/577
Lattice Parameters	a = 9.2936(5) Å	Residuals: R_1 ($I > 2.00\sigma(I)$)	0.0524
	b = 9.4122(5) Å	Residuals: wR_2 (all data)	0.1533
	c = 13.5766(6) Å	Goodness of Fit Indicator	1.057
	α = 101.016(4)°	Crystal growth	Slow evaporation of a $CHCl_3$/hexane solution
	β = 90.072(4)°		
	γ = 93.727(5)°		
	V = 1163.13(10) Å^3		

```
The following ALERTS were generated. Each ALERT has the format
       test-name_ALERT_alert-type_alert-level.
Click on the hyperlinks for more details of the test.
```

Alert level B

```
PLAT915_ALERT_3_B No Flack x Check Done: Low Friedel Pair Coverage         25 %
```

Author Response: In space group P1, it was not possible to cover more of reciprocal space.

Alert level C

```
PLAT094_ALERT_2_C Ratio of Maximum / Minimum Residual Density ....       3.12 Report
PLAT230_ALERT_2_C Hirshfeld Test Diff for    Si1A     --C17A     .        6.0 s.u.
PLAT230_ALERT_2_C Hirshfeld Test Diff for    Si1B     --C17B     .        6.0 s.u.
PLAT340_ALERT_3_C Low Bond Precision on  C-C Bonds ...............     0.0077 Ang.
PLAT411_ALERT_2_C Short Inter H...H Contact  H13A     ..H22A     .       2.12 Ang.
                                                    1+x,y,z  =      1_655 Check
PLAT411_ALERT_2_C Short Inter H...H Contact  H20B     ..H26B     .       2.14 Ang.
                                                    x,1+y,z  =      1_565 Check
PLAT767_ALERT_4_C INS Embedded LIST 6 Instruction Should be LIST 4     Please Check
PLAT911_ALERT_3_C Missing FCF Refl Between Thmin & STh/L=    0.600         42 Report
              -11  1  0, -10  1  0,  -9  1  0, -10 -3  1,  -6 -2  1, -10 -4  2,
              -10 -3  2,   6  5  3, -10  2  4,   8  6  4,   4  8  4,  -2  9  4,
              -10  2  5,   5  3  5,  -9 -5  6,  10 -4  6,  -6  7  6,  -2 -2  7,
              -10  0  7,  -6  7  7,   8 -7  8,  -8 -6  8,   9 -4  8,   2  8  8,
               -7 -6  9,  -8 -5  9,   9 -4  9,  -8  4  9,  -7 -6 10,  -8 -4 10,
                   (   12 More Missing: see the .ckf listing file)
```

Alert level G

```
PLAT720_ALERT_4_G Number of Unusual/Non-Standard Labels ..........          8 Note
              H1BA    H1BB    H8AA    H8AB    H8BA    H8BB    H1AA    H1AB
PLAT912_ALERT_4_G Missing # of FCF Reflections Above STh/L=  0.600        189 Note
PLAT941_ALERT_3_G Average HKL Measurement Multiplicity ..........         2.6 Low
PLAT969_ALERT_5_G The 'Henn et al.' R-Factor-gap value ..........       2.566 Note
              Predicted wR2: Based on SigI**2  5.97 or SHELX Weight 14.50
PLAT978_ALERT_2_G Number C-C Bonds with Positive Residual Density.          2 Info
```

5-6. *syn*-3a

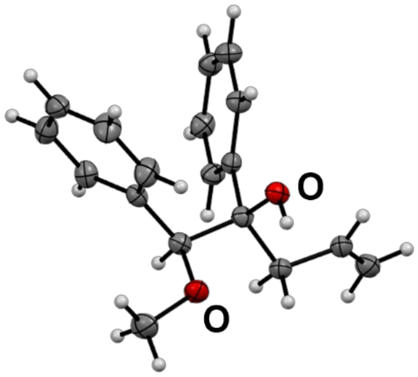

Figure S6. ORTEP drawings of *syn*-**3a** at the 50% probability level.

No. CCDC	2443727	Space Group	$P2_1/c$ (#14)
Empirical Formula	$C_{18}H_{20}O_2$	Z value	4
Formula Weight	268.34	D_{calc}	1.230 g/cm^3
Crystal Color	colorless	F_{000}	576.0
Crystal Dimensions	0.18 X 0.13 X 0.11 mm	μ(CuK$_\alpha$)	0.618 mm^{-1}
Crystal System	monoclinic	Temperature	123 K
Lattice Type	Primitive	Data/restraints/parameters	2870/0/183
Lattice Parameters	a = 12.1836(4) Å	Residuals: R_1 ($I > 2.00\sigma(I)$)	0.0402
	b = 5.7926(2) Å	Residuals: wR_2 (all data)	0.1063
	c = 20.5818(7) Å	Goodness of Fit Indicator	1.066
	β = 93.760(3) °	Crystal growth	Slow evaporation of a hexane solution
	V = 1449.43(8) Å^3		

Alert level C

PLAT911_ALERT_3_C Missing FCF Refl Between Thmin & STh/L= 0.600 3 Report
4 6 10, 3 6 11, -9 0 20,

Alert level G

PLAT007_ALERT_5_G Number of Unrefined Donor-H Atoms .... 1 Report
H1
PLAT912_ALERT_4_G Missing # of FCF Reflections Above STh/L= 0.600 116 Note
PLAT941_ALERT_3_G Average HKL Measurement Multiplicity .... 3.2 Low
PLAT969_ALERT_5_G The 'Henn et al.' R-Factor-gap value .... 1.91 Note
Predicted wR2: Based on SigI**2 5.57 or SHELX Weight 10.31
PLAT978_ALERT_2_G Number C-C Bonds with Positive Residual Density. 9 Info

0 **ALERT level A** = Most likely a serious problem - resolve or explain
0 **ALERT level B** = A potentially serious problem, consider carefully
1 **ALERT level C** = Check. Ensure it is not caused by an omission or oversight
5 **ALERT level G** = General information/check it is not something unexpected

0 ALERT type 1 CIF construction/syntax error, inconsistent or missing data
1 ALERT type 2 Indicator that the structure model may be wrong or deficient
2 ALERT type 3 Indicator that the structure quality may be low
1 ALERT type 4 Improvement, methodology, query or suggestion
2 ALERT type 5 Informative message, check

5-7. *anti*-3a′

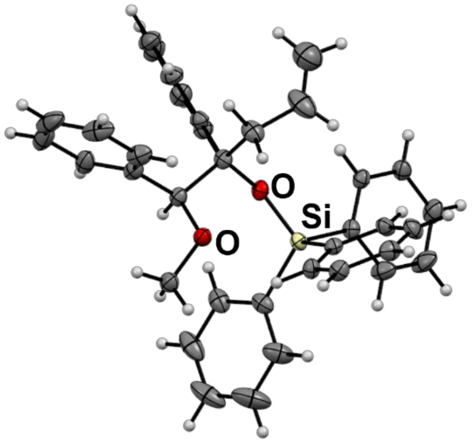

Figure S7. ORTEP drawings of *anti*-**3a′** at the 50% probability level.

No. CCDC	2443728	Space Group	$P2_1$/n (#14)
Empirical Formula	$C_{36}H_{34}O_2Si$	Z value	8
Formula Weight	526.72	D_{calc}	1.195 g/cm^3
Crystal Color	colorless	F_{000}	2240.0
Crystal Dimensions	0.14 X 0.13 X 0.04 mm	μ(CuK$_\alpha$)	0.934 mm^{-1}
Crystal System	monoclinic	Temperature	123 K
Lattice Type	Primitive	Data/restraints/parameters	11710/0/724
Lattice Parameters	a = 15.0576(2) Å	Residuals: R_1 ($I > 2.00\sigma(I)$)	0.0494
	b = 17.9231(3) Å	Residuals: wR_2 (all data)	0.1428
	c = 22.7246(3) Å	Goodness of Fit Indicator	1.057
	β = 107.265(2) °	Crystal growth	Slow evaporation of a AcOEt/hexane solution
	V = 5856.56(16) Å^3		

Alert level B

```
PLAT097_ALERT_2_B Large Reported Max.  (Positive) Residual Density       1.51 eA-3
```

Author Response: Because there is a minor unresolved disorders in the allyl group.

Alert level C

```
DIFMX02_ALERT_1_C  The maximum difference density is > 0.1*ZMAX*0.75
            The relevant atom site should be identified.
PLAT094_ALERT_2_C Ratio of Maximum / Minimum Residual Density ....       2.88 Report
PLAT329_ALERT_4_C Carbon Atom Hybridisation Unclear for .........        C1AA Check
PLAT911_ALERT_3_C Missing FCF Refl Between Thmin & STh/L=    0.600         25 Report
               16  0  0,   1 18  0,   2 18  0,   2 17  1,  16  0  2,   2 16  2,
               14  8  3,  16  0  4,  14  8  4,  13  7  5,  -5 20  8,   0  4 13,
              -10 16 14, -15  9 16,   5  9 16, -14  8 17, -14  9 17,   3 10 17,
                3 11 17, -16  3 18, -16  4 18, -16  1 19, -16  2 19,   4  7 22,
              -11  6 24,
PLAT971_ALERT_2_C Check Calcd Resid. Dens.  0.97Ang From C2              1.57 eA-3
PLAT977_ALERT_2_C Check Negative Difference Density on H0AC      .      -0.51 eA-3
PLAT977_ALERT_2_C Check Negative Difference Density on H38       .      -0.42 eA-3
```

Alert level G

```
PLAT301_ALERT_3_G Main Residue  Disorder ..............(Resd    1)          5% Note
PLAT395_ALERT_2_G Deviating  X-O-Y   Angle From 120 for O3       .      138.4 Degree
PLAT395_ALERT_2_G Deviating  X-O-Y   Angle From 120 for O1       .      139.8 Degree
PLAT410_ALERT_2_G Short Intra H...H Contact  H53      ..H39A     .       2.09 Ang.
                                                   x,y,z  =      1_555 Check
PLAT720_ALERT_4_G Number of Unusual/Non-Standard Labels .........           5 Note
              C0AA    H0AA    H0AB    C1AA    H0AC
PLAT793_ALERT_4_G Model has Chirality at C4          (Centro SpGr)          S Verify
PLAT793_ALERT_4_G Model has Chirality at C5          (Centro SpGr)          S Verify
PLAT793_ALERT_4_G Model has Chirality at C40         (Centro SpGr)          S Verify
PLAT793_ALERT_4_G Model has Chirality at C41         (Centro SpGr)          S Verify
PLAT910_ALERT_3_G Missing FCF Reflection(s) Below Theta(Min)[Deg]=       3.94 Note
               -1  0  1,   0  1  1,
PLAT912_ALERT_4_G Missing # of FCF Reflections Above STh/L=  0.600        361 Note
PLAT941_ALERT_3_G Average HKL Measurement Multiplicity ..........         3.6 Low
PLAT969_ALERT_5_G The 'Henn et al.' R-Factor-gap value ..........       3.090 Note
              Predicted wR2: Based on SigI**2  4.62 or SHELX Weight 13.52
PLAT978_ALERT_2_G Number C-C Bonds with Positive Residual Density.          6 Info
```

5-8. *anti*-3d

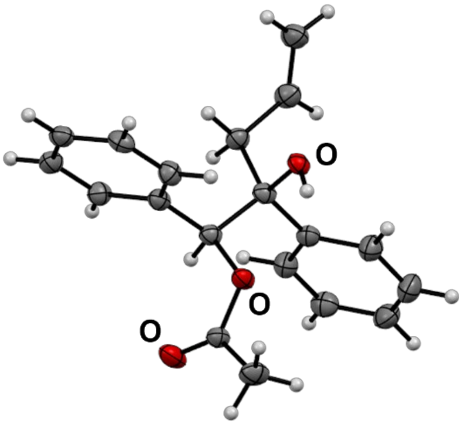

Figure S8. ORTEP drawings of *anti*-**3d** at the 50% probability level.

No. CCDC	2443729	Space Group	*R*-3 (#148)
Empirical Formula	$C_{19}H_{20}O_3$	*Z* value	18
Formula Weight	296.35	D_{calc}	1.175 g/cm^3
Crystal Color	colorless	F_{000}	2844.0
Crystal Dimensions	0.15 X 0.08 X 0.06 mm	μ(CuK_α)	0.629 mm^{-1}
Crystal System	trigonal	Temperature	123 K
Lattice Type	Primitive	Data/restraints/parameters	3358/0/201
Lattice Parameters	a = 39.3874(5) Å	Residuals: R_1 ($I > 2.00\sigma(I)$)	0.0646
	b = 39.3874(5) Å	Residuals: wR_2 (all data)	0.2008
	c = 5.60870(10) Å	Goodness of Fit Indicator	1.152
	V = 7535.4(2) Å^3	Crystal growth	Slow evaporation of a AcOEt/CH_3CN solution

Alert level A

PLAT097_ALERT_2_A Large Reported Max. (Positive) Residual Density   1.62 eA-3

Author Response: Due to the severe disorders of the lattice solvents.

Alert level B

PLAT094_ALERT_2_B Ratio of Maximum / Minimum Residual Density ....   6.00 Report

Author Response: Due to the severe disorders of the lattice solvents.

PLAT601_ALERT_2_B Unit Cell Contains Solvent Accessible VOIDS   <=   164 Ang**3

Author Response: Due to the severe disorders of the lattice solvents.

PLAT934_ALERT_3_B Number of (Iobs-Icalc)/Sigma(W) > 10 Outliers ..   3 Check
-2 4 0, -3 6 0, -7 8 0,

Author Response: Due to the severe disorders of the lattice solvents.

Alert level C

```
DIFMX02_ALERT_1_C  The maximum difference density is > 0.1*ZMAX*0.75
            The relevant atom site should be identified.
PLAT906_ALERT_3_C Large K Value in the Analysis of Variance ......      4.042 Check
PLAT911_ALERT_3_C Missing FCF Refl Between Thmin & STh/L=    0.600         14 Report
               -1  1  1, -15 16  2, -31 42  2, -30 43  2, -18 39  3, -20 40  3,
              -11 40  3, -22 41  3, -13 41  3, -24 42  3, -13 37  4, -23 26  5,
              -25 27  5,  -3 28  5,
PLAT918_ALERT_3_C Reflection(s) with I(obs) much Smaller I(calc) .          5 Check
PLAT939_ALERT_3_C Large Value of Not (SHELXL) Weight Optimized S .      15.67 Check
```

Alert level G

PLAT007_ALERT_5_G Number of Unrefined Donor-H Atoms ....   1 Report

5-9. Summary of structural data of allylatranes

Geometric parameters of allylatranes are summarized in Table S1. The mean values are shown in the case of two crystallographically independent molecules. The relatively short bond length of the N–E bond (**1**Si(allyl), 2.306 Å; **1**Ge(allyl), 2.480 Å; **1**Sn(allyl), 2.503 Å) indicates the efficient transannular interaction at the apical position. The bond length of the E–C_α increases in order from **1**Si(allyl) to **1**Ge(allyl) to **1**Sn(allyl) (**1**Si(allyl), 1.921 Å; **1**Ge(allyl), 2.011 Å; **1**Sn(allyl), 2.206 Å), which is induced by the difference in the elemental size. The bond alternation between C_α–C_β and C_β–C_γ in **1**E(allyl) was approximately 0.17 Å, which is nearly identical regardless of elemental center. The dihedral angles E–C_α–C_β–C_γ (E = Si, 46.0°; E = Ge, 56.1°; E = Sn, 61.2°) were considerably smaller than 90°, suggesting the weak σ–π conjugations.

Table S1. Summary of bond lengths, bond angles and torsion angles of allylatranes.

		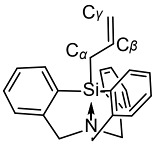	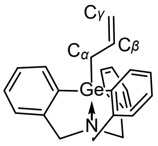	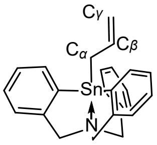	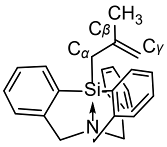	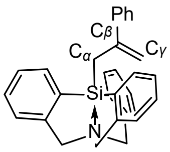
		**1**Si(allyl)	**1**Ge(allyl)	**1**Sn(allyl)	**1**Si(methallyl)	**1**Si(2-phenylallyl)
bond lengths /Å	N–E	2.306(1)	2.480(1)	2.503(2)	2.339(1)	2.303(3)
	E–C_α	1.921(2)	2.011(1)	2.206(3)	1.923(1)	1.925(4)
	C_α–C_β	1.494(2)	1.495(2)	1.490(4)	1.517(1)	1.517(4)
	C_β–C_γ	1.327(3)	1.322(3)	1.269(4)	1.328(1)	1.336(5)
torsion angles /°	E–C_α–C_β–C_γ	46.0(2)	56.1(1)	61.2(4)	9.1(1)	17.3(5)

6. Diastereoselective allylation reactions

6-1. Screening reaction conditions

6-1-1. Screening allyl nucleophiles

In a nitrogen-filled glovebox, to a mixture of $BF_3{\cdot}Et_2O$ (0.1 mmol) and **2a** (0.1 mmol) in dichloromethane (1 mL) was added allyl nucleophile (0.1 mmol). After the reaction mixture was stirred for 3 h at room temperature, methanol (1 mL) was added to the mixture. The solvents were removed in vacuum to give a crude product. The ^{1}H NMR measurement afforded the yield and diastereoselectivity using 1,1,2,2-tetrachloroethane as an internal standard.

Table S2. Screening allyl nucleophile in allylation of **2a**.

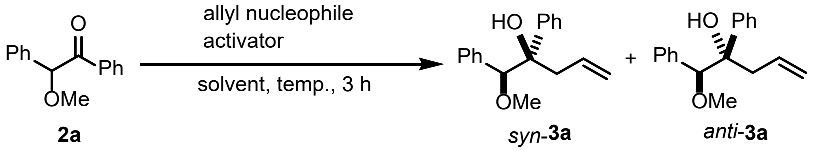

entry	allyl nucleophile	activator	solvent	temp.	yield/%	*syn*/*anti*
1	**1**Si(allyl) (1.0 equiv.)	$BF_3{\cdot}Et_2O$ (1.0 equiv.)	CH_2Cl_2	rt	97%	5/95
2	**1**Ge(allyl) (1.0 equiv.)	$BF_3{\cdot}Et_2O$ (1.0 equiv.)	CH_2Cl_2	rt	98%	5/95
3	**1**Sn(allyl) (1.0 equiv.)	$BF_3{\cdot}Et_2O$ (1.0 equiv.)	CH_2Cl_2	rt	60%	14/86
4	allylSi(OC_2H_4)$_3$N (1.0 equiv.)	$BF_3{\cdot}Et_2O$ (1.0 equiv.)	CH_2Cl_2	rt	0%	–
5	allylSi(cat)$_2$ (1.0 equiv.)	$BF_3{\cdot}Et_2O$ (1.0 equiv.)	CH_2Cl_2	rt	0%	–
6	allylSiMe_3 (1.0 equiv.)	$BF_3{\cdot}Et_2O$ (1.0 equiv.)	CH_2Cl_2	rt	6%	>99/1
7	allylSiPh_3 (1.0 equiv.)	$BF_3{\cdot}Et_2O$ (1.0 equiv.)	CH_2Cl_2	rt	5%	>99/1
8	allylSnBu_3 (1.0 equiv.)	$BF_3{\cdot}Et_2O$ (1.0 equiv.)	CH_2Cl_2	rt	99%	>99/1
9	allylSnPh_3 (1.0 equiv.)	$BF_3{\cdot}Et_2O$ (1.0 equiv.)	CH_2Cl_2	rt	94%	98/2
10	allylSnBu_3 (1.0 equiv.)	$SnCl_2$ (1.0 equiv.)	CH_3CN	rt	100%	>99/1
11	allylLi (1.5 equiv.)	–	THF	−78 °C to rt	99%	84/16
12	allylMgBr (1.5 equiv.)	–	THF	−78 °C to rt	94%	31/69
13	allylCl + Zn (1.5 equiv.)	–	THF	rt	97%	89/11
14	allylBr + In (1.5 equiv.)	–	THF	rt	94%	>99/1
15	allylBF_3K (1.0 equiv.)	$BF_3{\cdot}Et_2O$ (1.0 equiv.)	CH_2Cl_2	rt	0%[a]	–
16	allylBF_3K (1.0 equiv.)	$BF_3{\cdot}Et_2O$ (5 mol%)	CH_2Cl_2	rt	19%	12/88
17	allylBpin (1.0 equiv.)	$BF_3{\cdot}Et_2O$ (1.0 equiv.)	CH_2Cl_2	rt	4%	1/>99
18	allylSiMe_3 (1.0 equiv.)	TBAF (10 mol%)	THF	60 °C	39%	58/42

[a] rearrangement product was observed (53%).

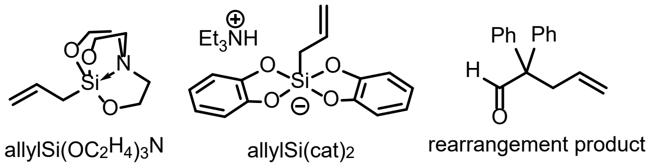

6-1-2. Screening activators and solvents with 1Si(allyl)

In a nitrogen-filled glovebox, to a mixture of activator (0.1 mmol) and **2a** (0.1 mmol) in solvent (1 mL) was added **1**Si(allyl) (0.1 mmol). After the reaction mixture was stirred for 3 h at room temperature, methanol (1 mL) was added to the mixture. The solvents were removed in vacuum to give a crude product. The ^{1}H NMR measurement afforded the yield and diastereoselectivity using 1,1,2,2-tetrachloroethane as an internal standard.

Table S3. Screening activator and solvent in allylation of **2a** with **1**Si(allyl).

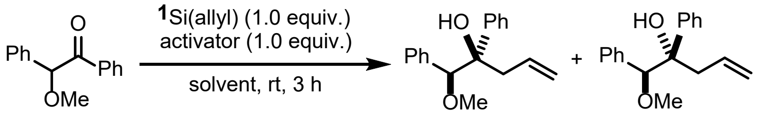

2a *syn*-**3a** *anti*-**3a**

entry	Lewis acid	solvent	yield/%	*syn*/*anti*
1	$BF_3 \cdot Et_2O$	CH_2Cl_2	97%	5/95
2	$TiCl_4$	CH_2Cl_2	93%	28/72
3	$SnCl_4$	CH_2Cl_2	96%	25/75
4	$AlCl_3$	CH_2Cl_2	85%	98/2
5	$InCl_3/Me_3SiCl$[a]	CH_2Cl_2	80%	98/2
6	$B(C_6F_5)_3$	CH_2Cl_2	0%[c]	–
7	$Sc(OTf)_3$	CH_2Cl_2	0%[c]	–
8	Me_3SiOTf	CH_2Cl_2	0%[c, d]	–
9	$BF_3 \cdot Et_2O$[b]	CH_2Cl_2	11%	10/90
10	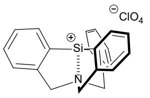 [**1**Si][ClO_4]	CH_2Cl_2	0%[c]	–
11	none	CH_2Cl_2	0%[c]	–
12	$BF_3 \cdot Et_2O$	$CHCl_3$	78%	13/87
13	$BF_3 \cdot Et_2O$	1,2-DCE	92%	6/94
14	$BF_3 \cdot Et_2O$	Et_2O	14%	>99/1
15	$BF_3 \cdot Et_2O$	CH_3CN	24%	13/87
16	$BF_3 \cdot Et_2O$	hexane	32%	31/69
17	$BF_3 \cdot Et_2O$	toluene	87%	88/12
18	$BF_3 \cdot Et_2O$	THF	0%[c]	–
19	$BF_3 \cdot Et_2O$	DMF	0%[c]	–

[a] 5 mol%, [b] 10 mol%, [c] Quantitative recovery of **2a** and the absence of any by-products were verified by means of NMR measurements of the reaction mixture. [d] An NMR analysis showed the rapid decomposition of **1**Si(allyl).

6-2. *anti*-Selective allylation using 1Si(allyl)

General procedure

In a nitrogen-filled glovebox, to a mixture of $BF_3{\cdot}Et_2O$ (0.2 mmol) and ketone **2** (0.2 mmol) in dichloromethane (2 mL) was added **1**Si(allyl) (0.2 mmol). After the reaction mixture was stirred for 3 h at room temperature, methanol (2 mL) was added to the mixture. The solvents were removed in vacuum to give a crude product. The ^{1}H NMR measurement afforded the yield and diastereoselectivity using 1,1,2,2-tetrachloroethane as an internal standard.

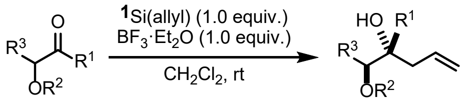

2 *anti*-**3**

6-3. *syn*-Selective allylation using Sn(II) salts

General procedure

To a mixture of $SnCl_2$ (0.24 mmol) and ketone **2** (0.2 mmol) in acetonitrile (2 mL) was added tributylallylstannane (0.24 mmol). After the reaction mixture was stirred for 12 h at room temperature, methanol (2 mL) was added to the mixture. The solvents were removed in vacuum to give a crude product. The ^{1}H NMR measurement afforded the yield and diastereoselectivity using 1,1,2,2-tetrachloroethane as an internal standard.

Table S4. *syn*-Selective allylation of α-oxy ketones using allylstannane and $SnCl_2$.

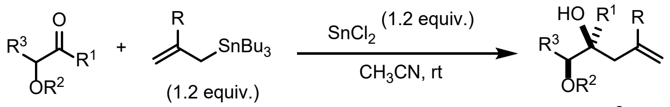

2 *syn*-**3**

substrate	yield/%	*syn*/*anti*	*syn*-**3**
**2b**	91	>99/1	*syn*-**3b**
**2c**	93	>99/1	*syn*-**3c**
**2d**	97	>99/1	*syn*-**3d**
**2e**	99	87/13	*syn*-**3e**
**2f**	89	>99/1	*syn*-**3f**
**2a**	73	97/3	*syn*-**3aa**
**2a**	62	98/2	*syn*-**3ab**

substrate	yield/%	*syn*/*anti*	*syn*-**3**
**2g**	91	>99/1	*syn*-**3g**
**2h**	97	>99/1	*syn*-**3h**
**2i**	100	>99/1	*syn*-**3i**
**2m**	37	52/48	*syn*-**3m**

6-4. Screening allyl nucleophiles in allylation of 2q

In a nitrogen-filled glovebox, to a mixture of activator (0.1 mmol) and **2q** (0.1 mmol) in dichloromethane (1 mL) was added allyl nucleophile (0.1 mmol). After the reaction mixture was stirred for 3 h at room temperature, methanol (1 mL) was added to the mixture. The solvents were removed in vacuum to give a crude product. The ^{1}H NMR measurement afforded the yield and diastereoselectivity using 1,1,2,2-tetrachloroethane as an internal standard.

Table S5. Screening reaction conditions in allylation of **2q**.

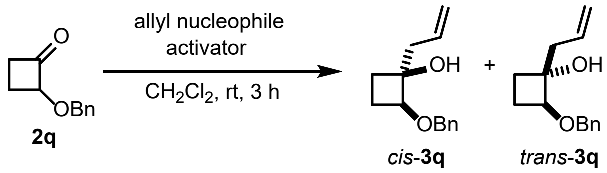

entry	allyl nucleophile	activator	temp.	yield/%	*cis*/*trans*
1	**1**Si(allyl) (1.0 equiv.)	$BF_3 \cdot Et_2O$ (1.0 equiv.)	rt	65%	7/93
2	**1**Si(allyl) (1.5 equiv.)	$BF_3 \cdot Et_2O$ (1.0 equiv.)	rt	98%	5/95
3	allyl$SiMe_3$ (1.0 equiv.)	$BF_3 \cdot Et_2O$ (1.0 equiv.)	rt	9%	1/>99
4	allylMgBr (2.0 equiv.)	–	0 °C to rt	82%	66/34
5	allylBF_3K (1.0 equiv.)	$BF_3 \cdot Et_2O$ (1.0 equiv.)	rt	35%	28/72
6	allyl$SnBu_3$ (1.0 equiv.)	$BF_3 \cdot Et_2O$ (1.0 equiv.)	rt	88%	27/73
7[a]	allyl$SnBu_3$ (1.2 equiv.)	$SnCl_2$ (1.2 equiv.)	rt	99%	52/48

[a] CH_3CN solvent.

6-5. Stereochemical determination of cyclobutane product 3q

For the stereochemical determination of **3q**, we performed independent acetalization[15] with 1,2-diols *cis*-**3q′** and *trans*-**3q′**, which were obtained by debenzylation of *cis*-**3q** and *trans*-**3q** with lithium naphthalenide. The corresponding 1,2-diol (12.8 mg, 0.1 mmol) was treated with 2,2-dimethoxypropane (20.8 mg, 0.2 mmol) and pyridinium *p*-toluenesulfonate (5.0 mg, 0.02 mmol) in THF-d_8. The reaction mixture was stirred at 60 °C for 1 h. The resulting mixture was analysed by ^{1}H NMR measurements. The signals of *cis*-**3q′** completely disappeared and the signals of the acetalized *cis*-**3q″** were observed (NMR a). In contrast, *trans*-**3q′** did not give the corresponding acetal with the same condition (NMR b). Therefore, we determined that the product **3q** obtained using **1**Si(allyl) is *trans*-form.

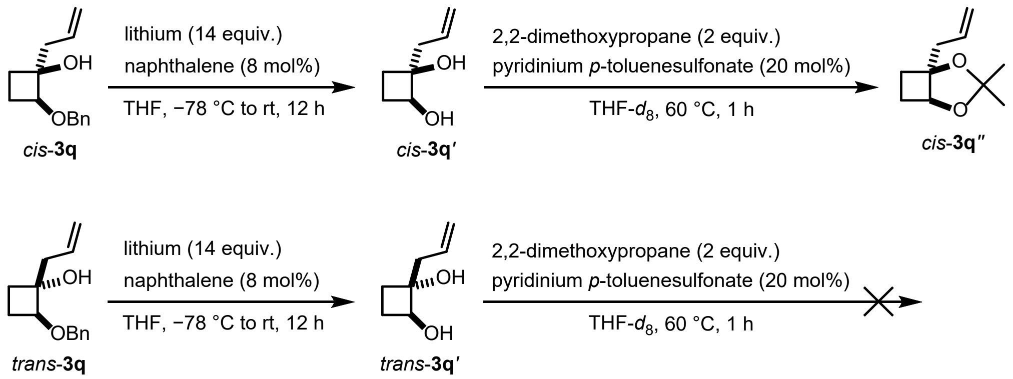

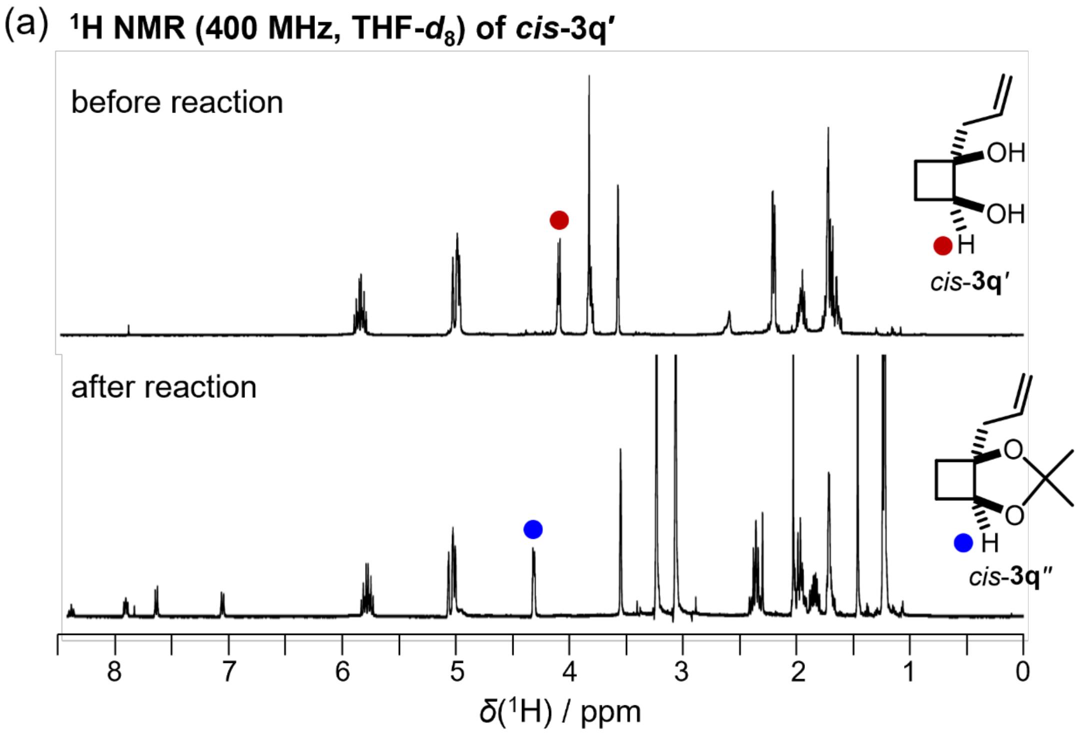

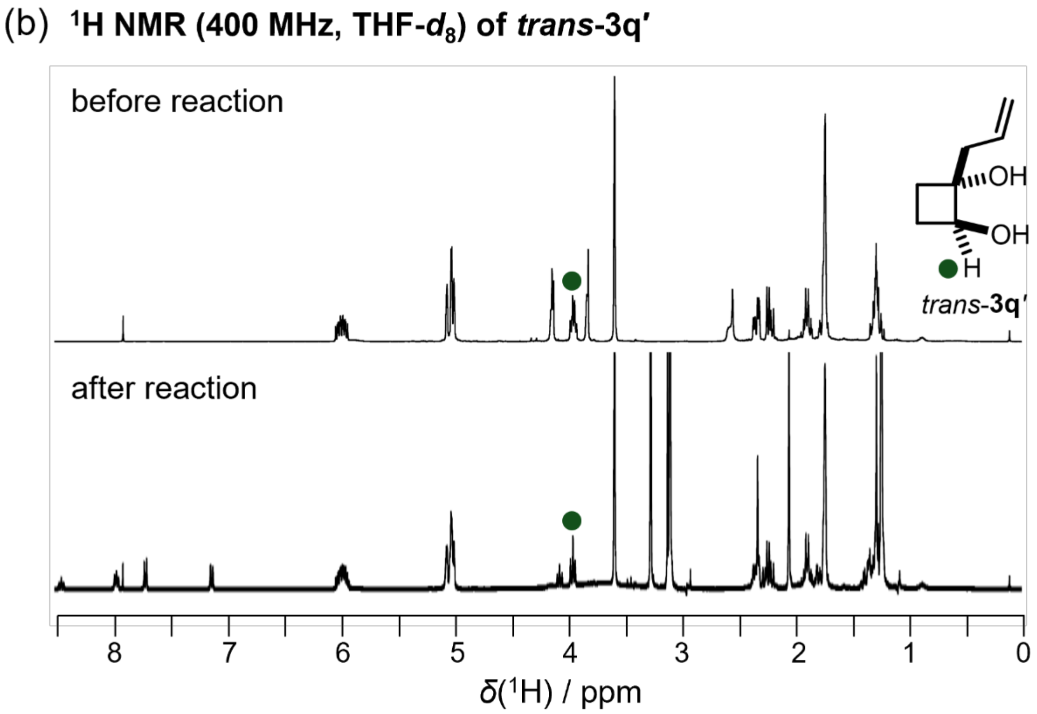

Figure S9. ^{1}H NMR spectra chenges after acetalization reaction. (a) *cis*-**3q′** (b) *trans*-**3q′**.

(1*S,2*S**)-1-Allylcyclobutane-1,2-diol *cis*-3q′**

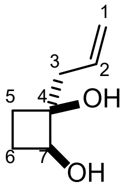

To a solution of lithium wires (54 mg, 7.8 mmol) and naphthalene (5.6 mg, 0.04 mmol) in THF (5 ml) were added (1*S**,2*S**)-1-allyl-2-(benzyloxy)cyclobutan-1-ol (121 mg, 0.55 mmol) at −78 °C. The reaction mixture was stirred to room temperature for 12 h. Water (10 mL) was added to quench the reaction and the mixture was extracted with ethyl acetate (3×20 mL). The obtained organic layer was dried over Na_2SO_4 and the solvent was removed in vacuum. The obtained residue was purified by column chromatography (hexane/ethyl acetate = 20/80) on silicagel to give the product as a colorless oil (49 mg, 69%).

^{1}H NMR (400 MHz, $CDCl_3$) 5.90–5.79 (m, 1H), 5.18–5.14 (m, 2H), 4.01 (t, J = 5.4 Hz, 1H), 2.55 (brs, 2H), 2.34 (d, J = 7.2 Hz, 2H), 2.20–2.13 (m, 1H), 1.94–1.84 (m, 2H), 1.81–1.73 (m, 1H); ^{13}C{^{1}H} NMR (100 MHz, $CDCl_3$) 132.9, 118.6, 77.2, 71.1, 43.6, 28.6, 26.7; HRMS (MALDI-TOF MS) Calculated ($C_7H_{12}O_2Na$): 151.0730 ([M+Na]$^+$), Found: 151.0731.

^{1}H NMR: (400 MHz, $CDCl_3$)

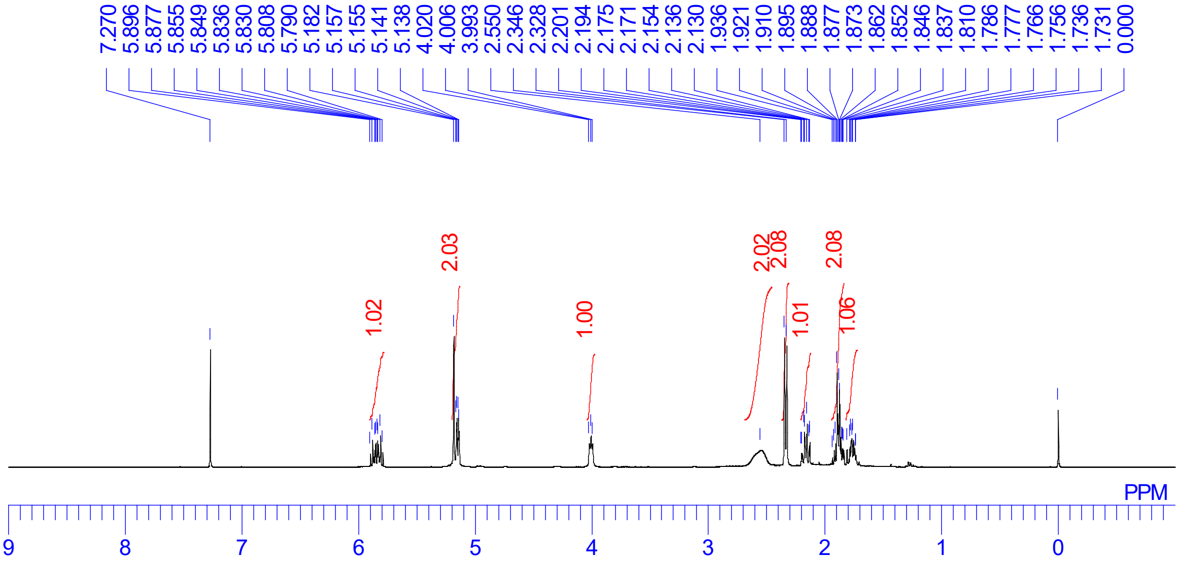

$^{13}C\{^1H\}$ NMR: (100 MHz, $CDCl_3$)

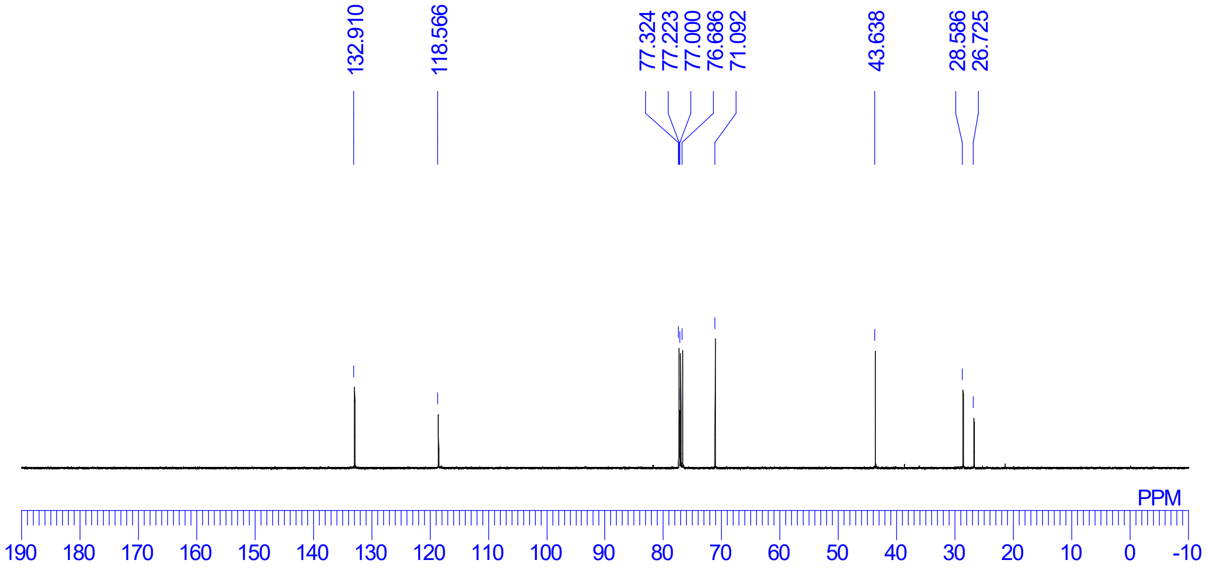

(1*R,2*S**)-1-Allylcyclobutane-1,2-diol *trans*-3q′**

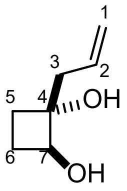

To a solution of lithium wires (26 mg, 3.7 mmol) and naphthalene (2.7 mg, 0.02 mmol) in THF (5 ml) were added (1*R**,2*S**)-1-allyl-2-(benzyloxy)cyclobutan-1-ol (57 mg, 0.26 mmol) at −78 °C. The reaction mixture was stirred to room temperature for 12 h. Water (10 mL) was added to quench the reaction and the mixture was extracted with ethyl acetate (3×20 mL). The obtained organic layer was dried over Na_2SO_4 and the solvent was removed in vacuum. The obtained residue was purified by column chromatography (hexane/ethyl acetate = 10/90) on silicagel to give the product as a colorless oil (28 mg, 85%).

^1H NMR (400 MHz, $CDCl_3$) 6.04–5.93 (m, 1H), 5.26–5.22 (m, 2H), 4.22 (t, J = 7.8 Hz, 1H), 2.48 (dd, J = 14.2, 7.8 Hz, 1H), 2.39 (dd, J = 14.0, 7.2 Hz, 1H), 2.14–2.05 (m, 2H), 1.96 (brs, 1H), 1.92–1.86 (m, 1H), 1.57–1.40 (m, 2H); $^{13}C\{^1H\}$ NMR (100 MHz, $CDCl_3$) 133.1, 119.9, 78.0, 75.6, 37.8, 26.6, 22.8; HRMS (MALDI-TOF MS) Calculated ($C_7H_{12}O_2Na$): 151.0730 ($[M+Na]^+$), Found: 151.0735.

^{1}H NMR: (400 MHz, $CDCl_3$)

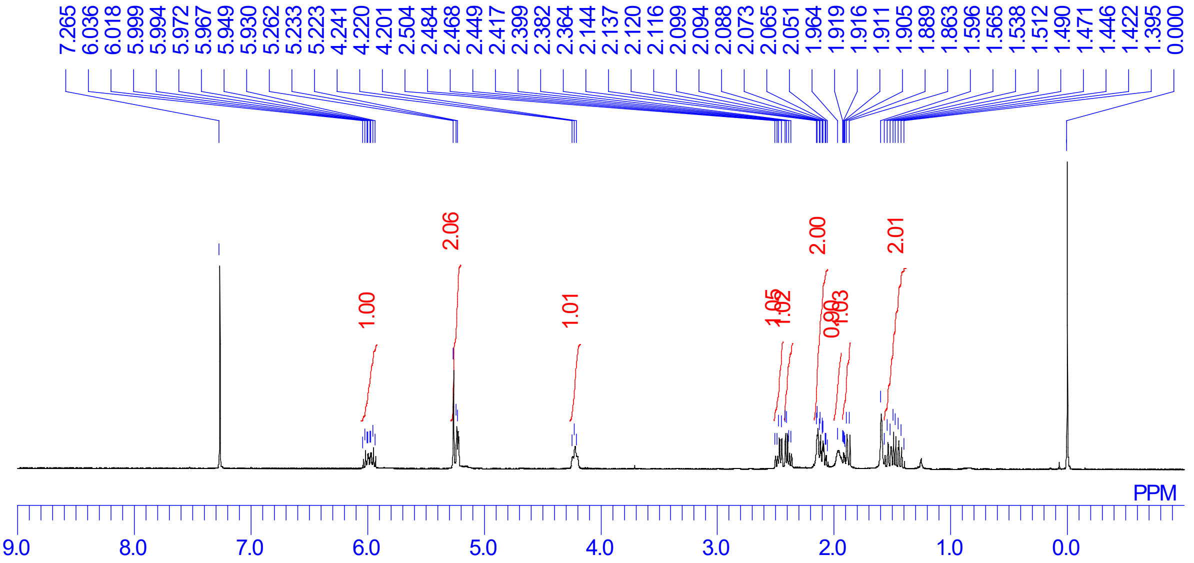

$^{13}C\{^{1}H\}$ NMR: (100 MHz, $CDCl_3$)

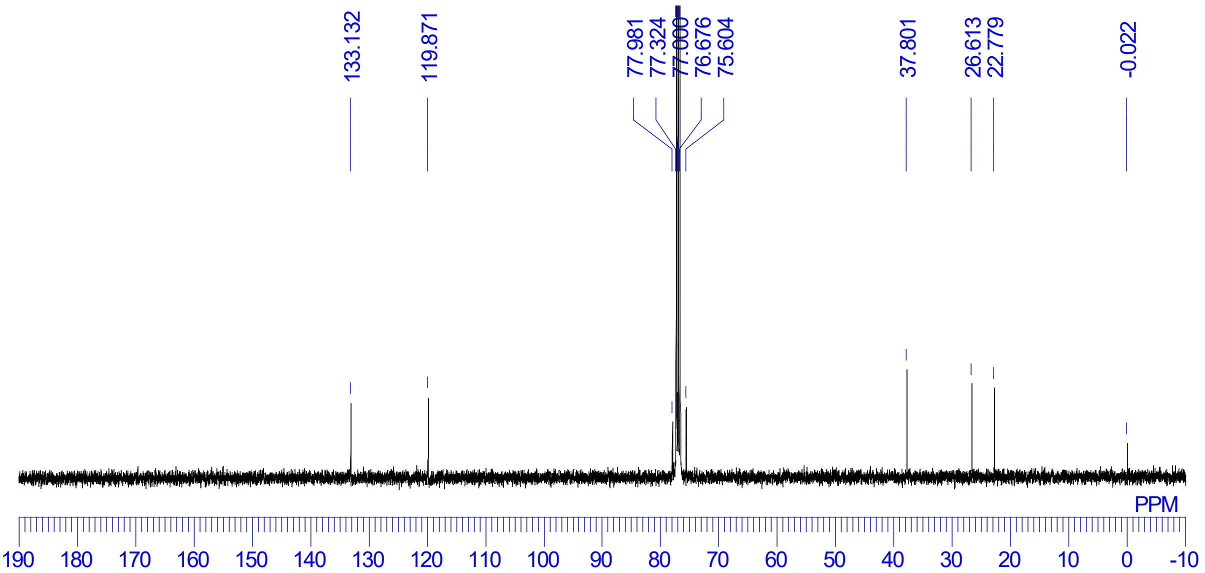

7. Mechanistic study

7-1. Stability of allylsilatranes

The stability of allylsilatranes against $BF_3 \cdot Et_2O$ was evaluated in ^1H NMR measurements. The mixture of allylsilatrane (allylSi$(OC_2H_4)_3N$ or **1**Si(allyl), 0.05 mmol) and $BF_3 \cdot Et_2O$ (0.05 mmol) in CD_2Cl_2 (0.6 mL) was monitored by ^1H NMR measurements. AllylSi$(OC_2H_4)_3N$ was immediately decomposed in the presence of $BF_3 \cdot Et_2O$. In contrast, the signals of **1**Si(allyl) were still observed after 5 min mixed with $BF_3 \cdot Et_2O$.

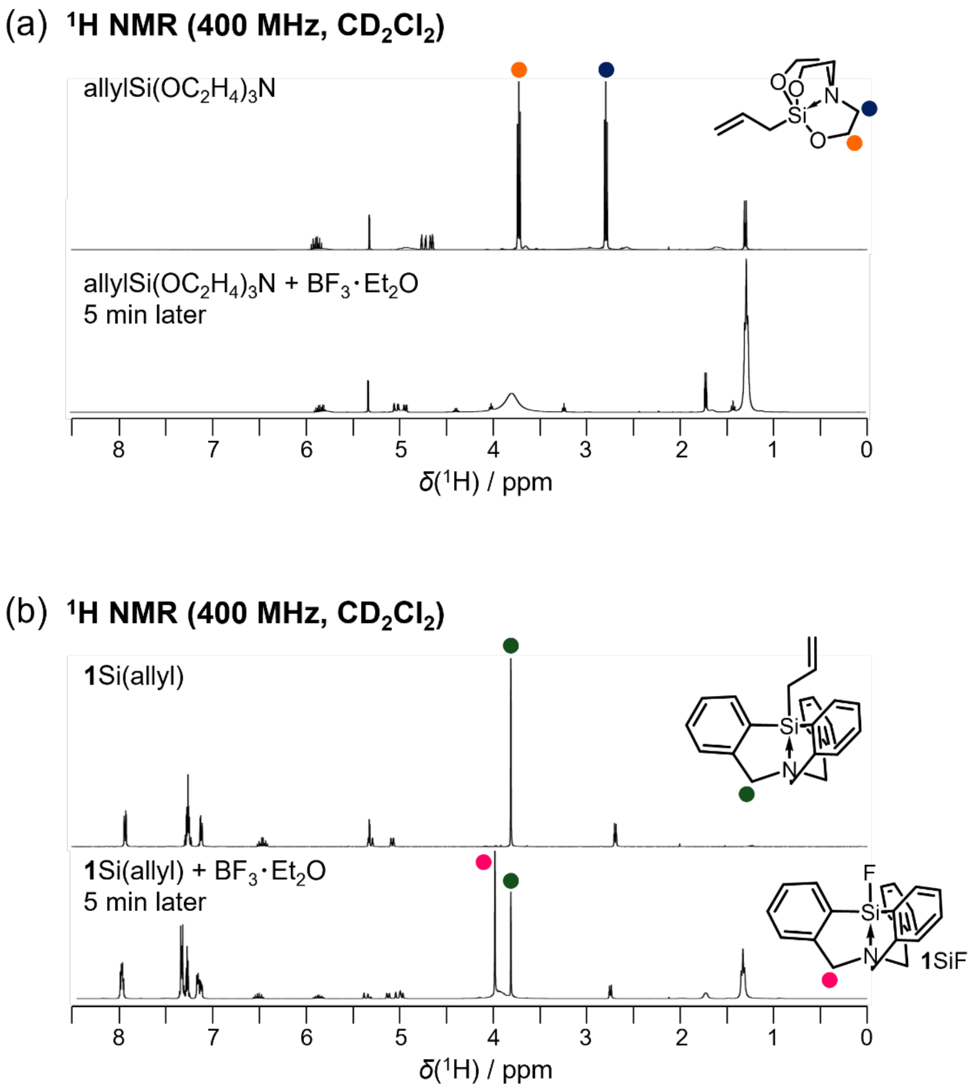

Figure S10. ^1H NMR spectra chenges of allylsilatrane after adding $BF_3 \cdot Et_2O$. (a) allylSi$(OC_2H_4)_3N$ (b) **1**Si(allyl).

7-2. Evaluation of nucleophilicity

The nucleophilicity of **1**E(allyl) and other allylsilanes was evaluated. HOMO orbitals, NBO atomic charges, and second order perturbation analysis was calculated by the B3PW91/6-31G** for C, H and N and DGDZVP for Si. The Δ*δ* values (^{13}C NMR, 100 MHz, $CDCl_3$) between the allylic carbons (C_β and C_γ) are also shown.

The HOMO orbital of **1**Si(allyl) was calculated as −6.18 eV, which suggested the higher nucleophilicity than that of allyltriphenylsilane (−6.60 eV). High nucleophilicity of **1**Si(allyl) is supported by ^{13}C NMR and NBO analysis. Meanwhile, the second order perturbation analysis indicated that the hyperconjugation from the σ(Si–C_α) bond to the π*(C_β=C_γ) bond was less effective in **1**Si(allyl) (Δ*E*=3.84 kcal/mol) than allyltriphenylsilane (Δ*E*=5.61 kcal/mol), illustrating the weak electronic communication between the allylic moiety and the elemental center of **1**Si(allyl). Thus, the nucleophilic character of **1**Si(allyl) is considered to reflect the charge localization rather than the stereoelectronic effects (Figure S11). The stabilization of the silyl cation by transannular interactions would enhance the allyl anion character. The allylatrane **1**E(allyl) was expected to act as a nucleophile with low Lewis acidity of the central element and sufficient nucleophilicity for *α*-oxy ketones.

Table S6. Summary of parameters related to nucleophilicity.

	**1**Si(allyl)	**1**Ge(allyl)	**1**Sn(allyl)	allylSiPh$_3$	allylSi(OC$_2$H$_4$)$_3$N	allylSnPh$_3$
	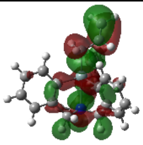	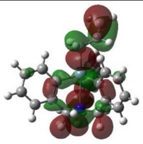	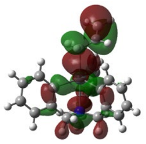	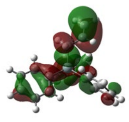	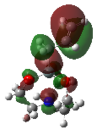	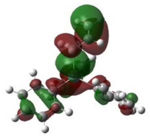
HOMO /eV	−6.18	−6.02	−5.91	−6.60	−6.17	−6.37
NBO charge						
q(E)	+1.891	+1.697	+1.898	+1.872	+2.379	+1.852
q(C_α)	−1.038	−0.970	−0.986	−1.009	−1.061	−0.958
q(C_β)	−0.215	−0.222	−0.230	−0.227	−0.217	−0.244
q(C_γ)	−0.492	−0.491	−0.506	−0.481	−0.491	−0.491
σ(E–C_α) →π*(C_β–C_γ) /kcal/mol	3.84	5.29	9.37	5.61	5.90	9.53
Δ$\delta_{C\beta\text{-}C\gamma}$ /ppm	25.3	24.4	28.4	18.7	27.8	23.5

B3PW91/DGDZVP (for Si, Ge, and Sn), 6–31+G**(for C, H, and N) level.

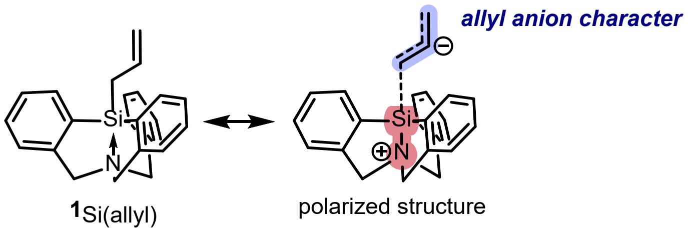

Figure S11. Resonance structure of **1**Si(allyl).

7-3. In-situ observation of allylation

We monitored the allylation reaction of **2a** with **1**Si(allyl) in the presence of $BF_3{\cdot}Et_2O$ by NMR measurement. Two different orders of reagent addition (i and ii) were examined to investigate the possibility of in-situ exchange of allyl moiety between **1**Si(allyl) and $BF_3{\cdot}Et_2O$. The order (i) is consistent with the standard procedure for reagent addition.

(i) $BF_3{\cdot}Et_2O$ in CD_2Cl_2 → ketone 2a → 1Si(allyl) → methanol-*d*$_4$

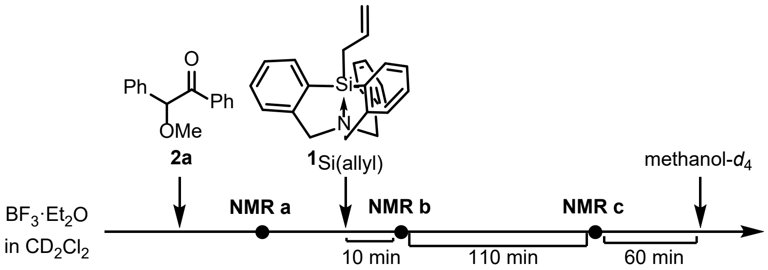

In a nitrogen-filled glovebox, to a solution of $BF_3{\cdot}Et_2O$ (0.2 mmol) in CD_2Cl_2 (0.6 mL) was added ketone **2a** (0.2 mmol) at room temperature. At that point, the signals of **2a** and $BF_3{\cdot}Et_2O$ were not changed in ^1H NMR spectra and ^{11}B NMR spectra (NMR a). After that, **1**Si(allyl) (0.2 mmol) was added to the mixture. After 10 minutes, the signals of an allyl product **Int** (δ_{11B} = 4.7 ppm) and fluorine-substituted silatrane **1**SiF were observed (NMR b). Further 110 minutes later, the signals of **1**Si(allyl) and **2a** have almost completely disappeared (NMR c). Further 60 minutes later, addition of methanol-*d*$_4$ (0.1 mL) facilitated protonation of **Int**, resulting in the formation of the desired product **3a** in 98% yield (*syn*/*anti* = 3/97). The selectivity were consistent with the results obtained under batch conditions (*syn*/*anti* = 5/95, entry 1 in Table S2). In conclusion, the allyl exchange between **1**Si(allyl) and $BF_3{\cdot}Et_2O$ was not observed in order (i).

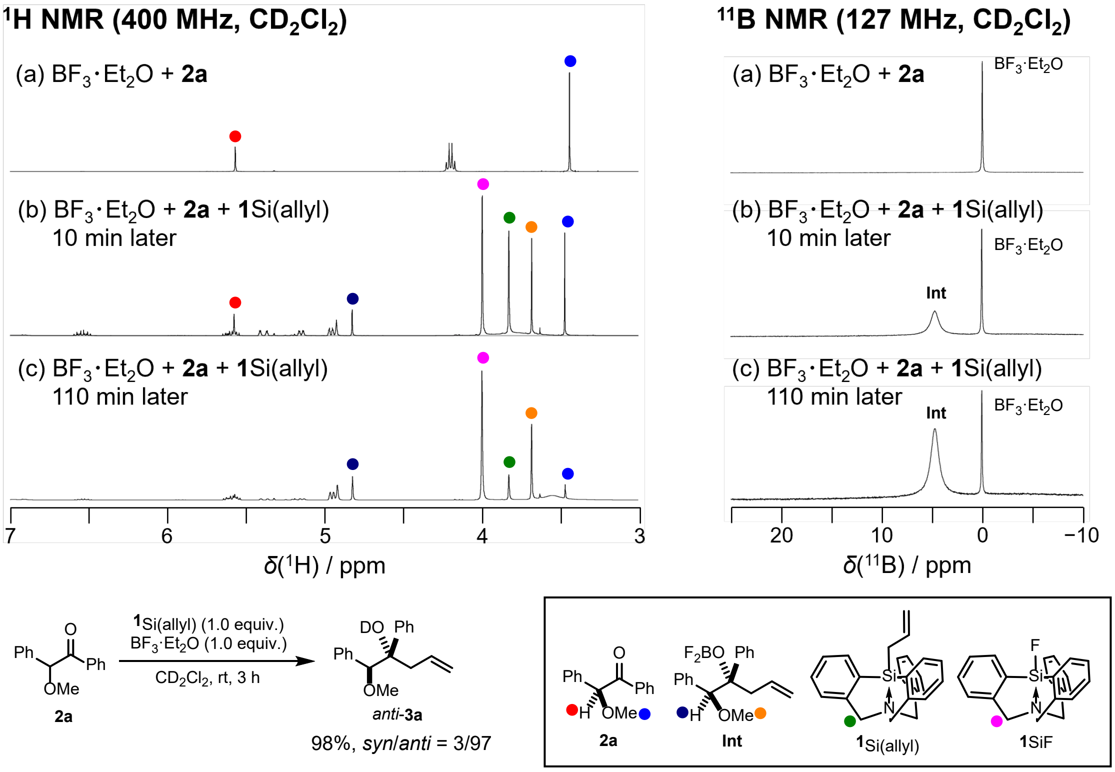

Figure S12. Monitoring of the allylation reaction by ^1H NMR and ^{11}B NMR analysis.

(ii) 1Si(allyl) in CD_2Cl_2 → BF_3·Et_2O → ketone 2a → methanol-*d_4*

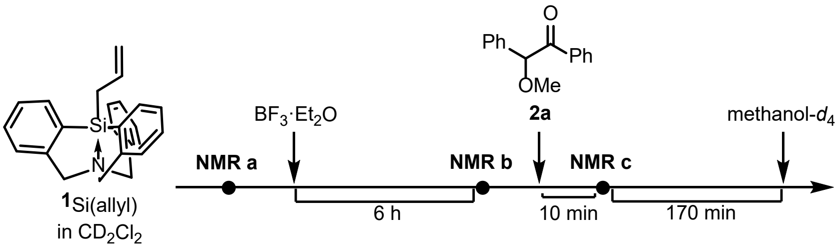

In a nitrogen-filled glovebox, **1**Si(allyl) (0.2 mmol) was added to CD_2Cl_2 solvent (0.6 mL) at room temperature (NMR a). After that, BF_3·Et_2O (0.2 mmol) was added to the mixture. After 6 h, the signal of **1**Si(allyl) have disappeared, and the signals corresponding to **1**SiF and allylBF_2·Et_2O (δ_{11B} = 17.8 ppm) were observed (NMR b). The chemical shift in ^{11}B NMR is comparable to that of the reported allylBF_2·Et_2O (δ_{11B} = 15.6 ppm in $CDCl_3$).[16] After that, the ketone **2a** was added to the mixture. After 10 minutes, the signal of **Int** (δ_{11B} = 4.7 ppm) was observed (NMR c). Further 170 minutes later, addition of methanol-*d_4* (0.1 mL) facilitated protonation of **Int**, resulting in the formation of the desired product **3a** in 69% yield (*syn*/*anti* = 17/83). The obtained diastereo ratio is closed to that observed under the condition using allylBF_3K and BF_3·Et_2O (*syn*/*anti* = 12/88, entry 15 in Table S2). It was found that allylBF_2·Et_2O generated by allyl exchange exhibited reduced diastereoselectivity compared to **1**Si(allyl).

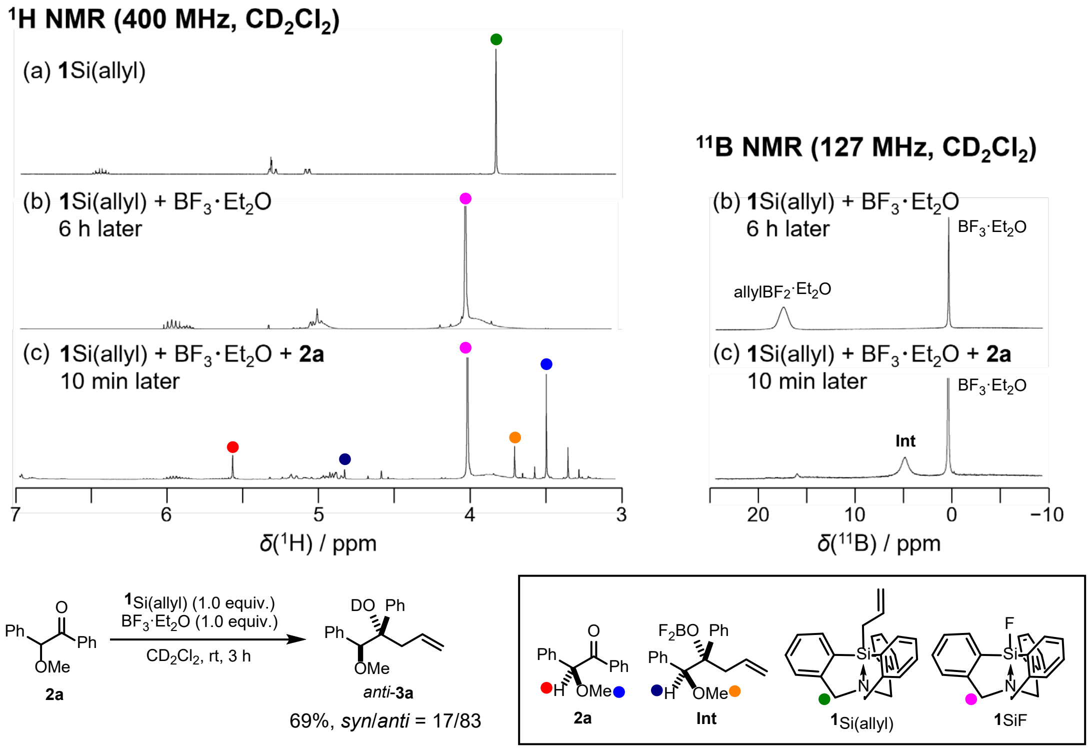

Figure S13. Monitoring of the allylation reaction by ^{1}H NMR and ^{11}B NMR analysis.

Based on the results of (i) and (ii), the possibility that allylBF_2·Et_2O, which is generated by in-situ allyl exchange, behaves as the allyl reagent can be ruled out. Thus, BF_3·Et_2O solely works as a Lewis acid activator of carbonyl group of the ketone.

8. Computational method

8-1. General

All calculations were conducted using the Gaussian 16 Rev. C. 01 program.[17] The optimizations of **1**E(allyl) were performed with the B3PW91/DGDZVP (for Si, Ge, and Sn), 6–31+G**(for C, H, and N) level. The obtained optimized structures are local minimum structures with all positive vibrational frequencies. Natural bond orbital analyses[18,19] were performed under the optimized geometries using the NBO version 3.1[20] program as including in Gaussian.

8-2. HOMO energies of 1E(allyl)

(a)

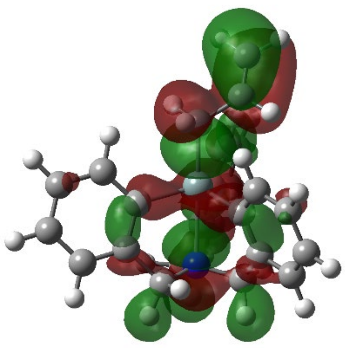

1Si(allyl)
–6.18 eV

(b)

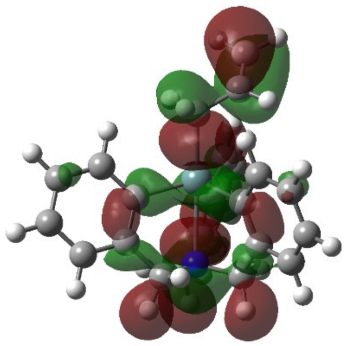

1Ge(allyl)
–6.02 eV

(c)

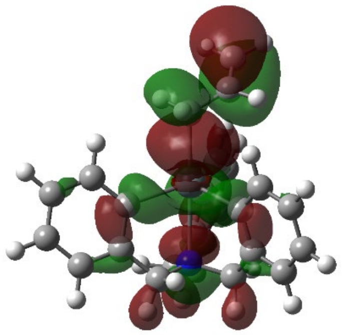

1Sn(allyl)
–5.91 eV

(d)

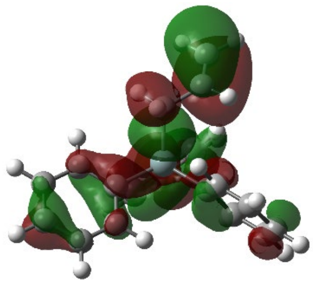

allylSiPh$_3$
–6.60 eV

(e)

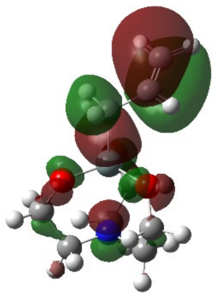

allylSi(OC$_2$H$_4$)$_3$N
–6.17 eV

(f)

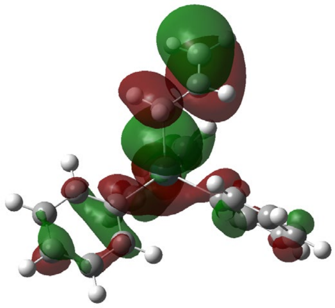

allylSnPh$_3$
–6.37 eV

Figure S14. HOMO of (a) **1**Si(allyl), (b) **1**Ge(allyl), (c) **1**Sn(allyl), (d) allylSiPh$_3$, (e) allylSi(OC$_2$H$_4$)$_3$N, and (f) allylSnPh$_3$ calculated at the B3PW91/DGDZVP (for Si, Ge, and Sn), 6–31+G**(for C, H, and N) level.

8-3. Second order perturbation analysis of allylic moieties of 1E(allyl)

(a) σ(Si–C_{α}) → π*(C_{β}–C_{γ}) 3.84 kcal/mol

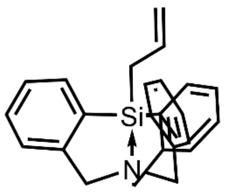

1Si(allyl)

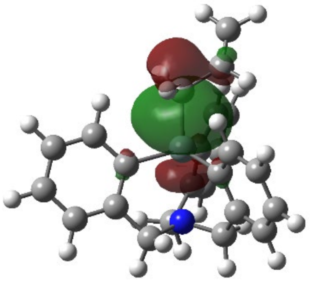

σ(Si–C_{α})

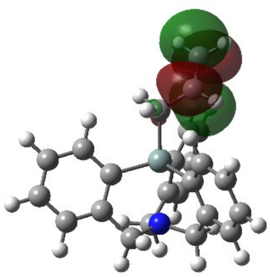

π*(C_{β}–C_{γ})

(b) σ(Ge–C_{α}) → π*(C_{β}–C_{γ}) 5.29 kcal/mol

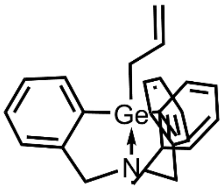

1Ge(allyl)

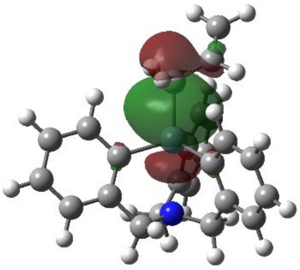

σ(Ge–C_{α})

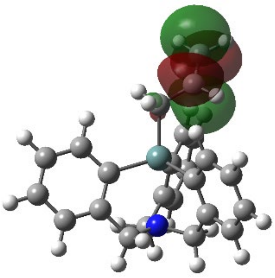

π*(C_{β}–C_{γ})

(c) σ(Sn–C_{α}) → π*(C_{β}–C_{γ}) 9.37 kcal/mol

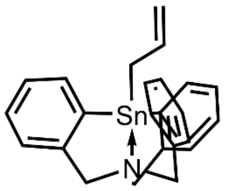

1Sn(allyl)

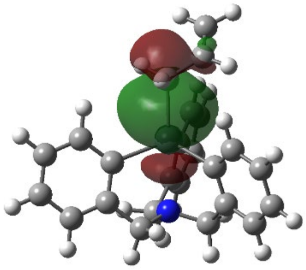

σ(Sn–C_{α})

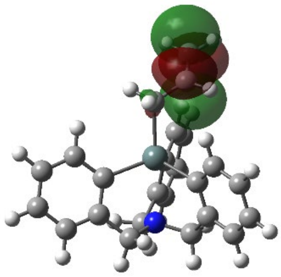

π*(C_{β}–C_{γ})

(d) σ(Si–C_{α}) → π*(C_{β}–C_{γ}) 5.61 kcal/mol

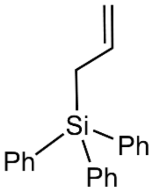

allylSiPh_3

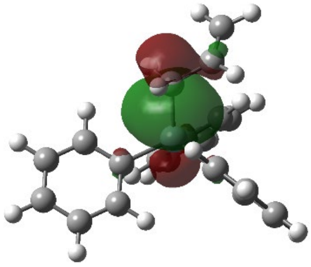

σ(Si–C_{α})

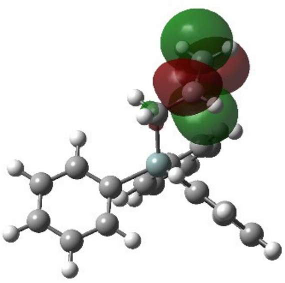

π*(C_{β}–C_{γ})

(e) σ(Si–C_α) → π*(C_β–C_γ) 5.90 kcal/mol

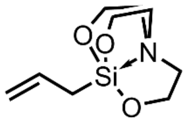

$allylSi(OC_2H_4)_3N$

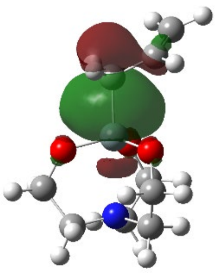

σ(Si–C_α)

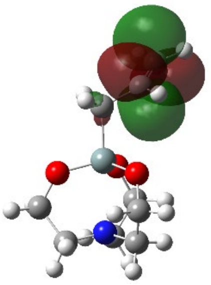

π*(C_β–C_γ)

(f) σ(Sn–C_α) → π*(C_β–C_γ) 9.53 kcal/mol

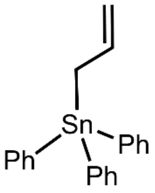

$allylSnPh_3$

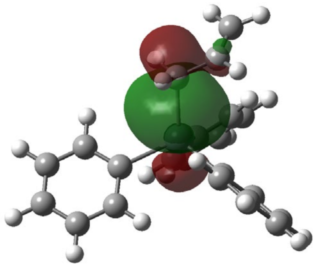

σ(Sn–C_α)

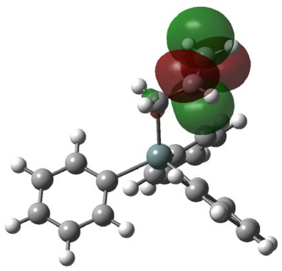

π*(C_β–C_γ)

Figure S15. Natural bond orbitals of (a) **1**Si(allyl), (b) **1**Ge(allyl), (c) **1**Sn(allyl), (d) $allylSiPh_3$, (e) $allylSi(OC_2H_4)_3N$, and (f) $allylSnPh_3$ calculated at the B3PW91/DGDZVP (for Si, Ge, and Sn), 6–31+G**(for C, H, and N) level.

[SI]

9. Computational estimation of the reaction profile

9-1. General

All calculations were conducted using the Gaussian 16 Rev. C. 01 program.[17] For the reaction profiles, the B3LYP-D3 functional was used in combination with the 6-31G* basis set. All molecular geometries were fully optimized at the singlet state and confirmed to have all positive vibrational frequencies for local minima or an imaginary vibrational frequency for transition states at the same theoretical level. By using the obtained optimized structures, Gibbs free energies including contribution of vibrational entropy at an appropriate temperature were described at the same theoretical level, in which solvation effect was introduced using the SMD model (dichloromethane).

9-2. Summary for the reaction profiles for the allylations of 2a with 1Si(allyl) and BF_3

Table S7. Summary for the energy in the *anti*-allylation of **2a**.

state	total energy / Hartree [a]	Zero-point energy / Hartree [b]	Thermal correlation for Gibbs energy / Hartree [b]	Total energy +ZPE / Hartree	Gibus energy (298 K) / Hartree	imaginary frequency / cm^{-1} [b]
**2a**·BF_3	−1055.0808175	0.2669856	0.215846	−1054.8138319	−1054.8649715	–
**1**Si(allyl)	−1272.7421729	0.4078174	0.3593160	−1272.3343555	−1272.3828569	–
sum	−2327.8229904	0.6748030	0.5751620	−2327.1481874	−2327.2478284	–
**TS**(*anti*)	−2327.8368437	0.6789008	0.6055910	−2327.1579429	−2327.2312527	348.1523*i*
**Int′**(*anti*)	−2327.8543954	0.6803922	0.6064130	−2327.1740032	−2327.2479824	–
**Int**(*anti*-**3a**)	−1072.5440874	0.3389021	0.2871920	−1072.2051853	−1072.2568954	–
**1**SiF	−1255.3459658	0.3396299	0.2944420	−1255.0063359	−1255.0515238	–
sum	−2327.8900532	0.6785320	0.5816340	−2327.2115212	−2327.3084192	–

[a] B3LYP-D3/6-31G*/SMD (dichloromethane)//B3LYP-D3/6-31G*

[b] B3LYP-D3/6-31G*

Table S8. Summary for the energy in the *syn*-allylation of **2a**.

state	total energy / Hartree [a]	Zero-point energy / Hartree [b]	Thermal correlation for Gibbs energy / Hartree [b]	Total energy +ZPE / Hartree	Gibus energy (298 K) / Hartree	imaginary frequency / cm^{-1} [b]
**2a**·BF_3	−1055.0808175	0.2669856	0.215846	−1054.8138319	−1054.8649715	–
**1**Si(allyl)	−1272.7421729	0.4078174	0.3593160	−1272.3343555	−1272.3828569	–
sum	−2327.8229904	0.6748030	0.5751620	−2327.1481874	−2327.2478284	–
**TS**(*syn*)	−2327.8276692	0.6790139	0.6066700	−2327.1486553	−2327.2209992	350.2415*i*
**Int′**(*syn*)	−2327.8509799	0.6806515	0.6082270	−2327.1703284	−2327.2427529	–
**Int**(*syn*-**3a**)	−1072.5369396	0.3396299	0.2944420	−1072.1973097	−1072.2424976	–
**1**SiF	−1255.3459658	0.3396299	0.2944420	−1255.0063359	−1255.0515238	–
sum	−2327.8829054	0.6792598	0.5888840	−2327.2036456	−2327.2940214	–

[a] B3LYP-D3/6-31G*/SMD (dichloromethane)//B3LYP-D3/6-31G*

[b] B3LYP-D3/6-31G*

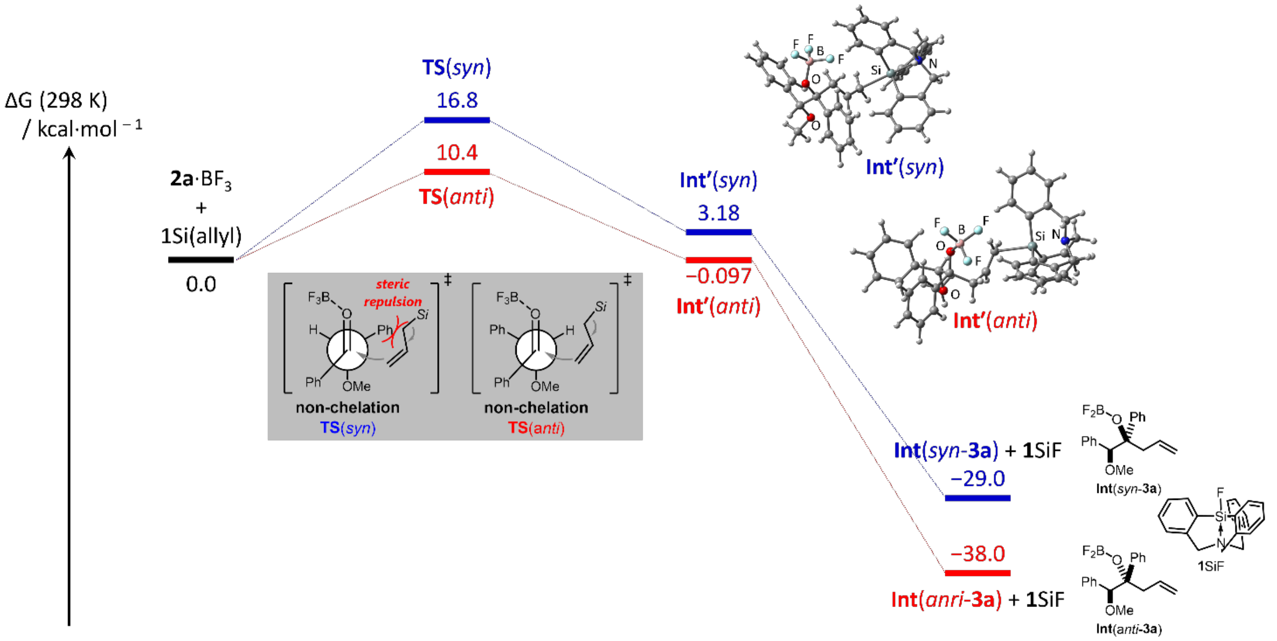

Figure S16. Energy diagram for the allylations of **2a** with **1**Si(allyl) and BF_3. The Gibbs-free-energy values relative to **2a**·BF_3 **and 1**Si(allyl) were calculated at the B3LYP-D3/6-31G*/SMD (dichloromethane)//B3LYP-D3/6-31G* level.

10. References

1. Dolomanov, O. V., Bourhis, L. J., Gildea, R. J., Howard, J. a. K. & Puschmann, H. OLEX2: a complete structure solution, refinement and analysis program. *J Appl Cryst* **42**, 339–341 (2009).
2. Tanaka, D., Konishi, A. & Yasuda, M. Synthesis and Catalytic Activity of Atrane-type Hard and Soft Lewis Superacids with a Silyl, Germyl, or Stannyl Cationic Center. *Chemistry – An Asian Journal* **16**, 3118–3123 (2021).
3. Gao, K. *et al.* Cobalt-Catalyzed Reductive C–O Bond Cleavage of Lignin β-O-4 Ketone Models via In Situ Generation of the Cobalt–Boryl Species. *Org. Lett.* **22**, 6055–6060 (2020).
4. Cutulic, S. P. Y., Findlay, N. J., Zhou, S.-Z., Chrystal, E. J. T. & Murphy, J. A. Metal-Free Reductive Cleavage of C−O σ-bonds in Acyloin Derivatives by an Organic Neutral Super-Electron-Donor. *J. Org. Chem.* **74**, 8713–8718 (2009).
5. Bartolo, N. D. *et al.* Conformationally Biased Ketones React Diastereoselectively with Allylmagnesium Halides. *J. Org. Chem.* **87**, 3042–3065 (2022).
6. Zhang, M.-Z. *et al.* Transition-Metal-Free Oxidative Aminooxyarylation of Alkenes: Annulations toward Aminooxylated Oxindoles. *J. Org. Chem.* **83**, 2369–2375 (2018).
7. Read, J. A., Yang, Y. & Woerpel, K. A. Additions of Organomagnesium Halides to α-Alkoxy Ketones: Revision of the Chelation-Control Model. *Org. Lett.* **19**, 3346–3349 (2017).
8. Dunn, J. & Dobbs, A. P. Synthesis and reactions of donor cyclopropanes: efficient routes to *cis*- and *trans*-tetrahydrofurans. *Tetrahedron* **71**, 7386–7414 (2015).
9. Matsumoto, K., Okamoto, T. & Otsuka, K. Optical Resolution of Acyclic α-Hydroxy Ketone Derivatives by Inclusion Complexation. *Bulletin of the Chemical Society of Japan* **77**, 2051–2056 (2004).
10. Lipp, A., Badir, S. O., Dykstra, R., Gutierrez, O. & Molander, G. A. Catalyst-Free Decarbonylative Trifluoromethylthiolation Enabled by Electron Donor-Acceptor Complex Photoactivation. *Advanced Synthesis & Catalysis* **363**, 3507–3520 (2021).
11. Wender, P. A. & Rawlins, D. B. Toward the synthesis of the taxol C,D, ring system: Photolysis of α-methoxy ketones. *Tetrahedron* **48**, 7033–7048 (1992).
12. Yu, W., Williams, L., Camp, V. M., Olson, J. J. & Goodman, M. M. Synthesis and biological evaluation of *anti*-1-amino-2-[18F]fluoro-cyclobutyl-1-carboxylic acid (*anti*-2-[18F]FACBC) in rat 9L gliosarcoma. *Bioorganic & Medicinal Chemistry Letters* **20**, 2140–2143 (2010).
13. Yasuda, M., Fujibayashi, T. & Baba, A. Allylation of Carbonyl Compounds Bearing a Hydroxyl Group by Tetraallyltin: Highly Stereoselective Allylation in a Chelation-Controlled Manner. *J. Org. Chem.* **63**, 6401–6404 (1998).
14. Paquette, L. A. & Lobben, P. C. π-Facial Diastereoselection in the 1,2-Addition of Allylmetal Reagents to 2-Methoxycyclohexanone and Tetrahydrofuranspiro-(2-cyclohexanone). *J. Am. Chem. Soc.* **118**, 1917–1930 (1996).
15. Gómez-Gil, S. *et al.* Synthesis of 1,4-ketoaldehydes and 1,4-diketones by Mo-catalyzed oxidative cleavage of cyclobutane-1,2-diols. *Org. Biomol. Chem.* **21**, 4185–4190 (2023).
16. Le, S. S. & Guillemin, J.-C. Synthesis and Characterization of Allylic Dihaloboranes. *Organometallics* **16**, 5844–5848 (1997).

17. Frisch, M. J.; Trucks, G. W.; Schlegel, H. B.; Scuseria, G. E.; Robb, M. A.; Cheeseman, J. R.; Scalmani, G.; Barone, V.; Petersson, G. A.; Nakatsuji, H.; Li, X.; Caricato, M.; Marenich, A. V.; Bloino, J.; Janesko, B. G.; Gomperts, R.; Mennucci, B.; Hratchian, H. P.; Ortiz, J. V.; Izmaylov, A. F.; Sonnenberg, J. L.; Williams-Young, D.; Ding, F.; Lipparini, F.; Egidi, F.; Goings, J.; Peng, B.; Petrone, A.; Henderson, T.; Ranasinghe, D.; Zakrzewski, V. G.; Gao, J.; Rega, N.; Zheng, G.; Liang, W.; Hada, M.; Ehara, M.; Toyota, K.; Fukuda, R.; Hasegawa, J.; Ishida, M.; Nakajima, T.; Honda, Y.; Kitao, O.; Nakai, H.; Vreven, T.; Throssell, K.; Montgomery, J. A., Jr.; Peralta, J. E.; Ogliaro, F.; Bearpark, M. J.; Heyd, J. J.; Brothers, E. N.; Kudin, K. N.; Staroverov, V. N.; Keith, T. A.; Kobayashi, R.; Normand, J.; Raghavachari, K.; Rendell, A. P.; Burant, J. C.; Iyengar, S. S.; Tomasi, J.; Cossi, M.; Millam, J. M.; Klene, M.; Adamo, C.; Cammi, R.; Ochterski, J. W.; Martin, R. L.; Morokuma, K.; Farkas, O.; Foresman, J. B.; Fox, D. J. Gaussian 16, Revision C.01,. *Gaussian, Inc., Wallingford CT,* (2016).
18. Neese, F. The ORCA program system. *WIREs Computational Molecular Science* **2**, 73–78 (2012).
19. Reed, A. E., Curtiss, L. A. & Weinhold, F. Intermolecular interactions from a natural bond orbital, donor-acceptor viewpoint. *Chem. Rev.* **88**, 899–926 (1988).
20. Glendening, E. D., Reed, A. E., Carpenter, J. E. & Weinhold, F. NBO Version 3.1.
